# A graph theory approach to chart the architecture of dream and mind-wandering reports

**DOI:** 10.1038/s44271-026-00509-5

**Published:** 2026-10-08

**Authors:** Peter Coppola, Pilleriin Sikka, Katja Valli, Jarno Tuominen, Antti Revonsuo, Ville Loukola, Ryan Bernstein, Nanna Strid, Manuela Kirberg, Alejandro Ezquerro-Nassar, Jennifer Windt, Valdas Noreika, Natália Bezerra Mota, Tristan Andres Bekinschtein

**Affiliations:** 1 Consciousness and Cognition lab, Department of Psychology, University of Cambridge, Cambridge, UK; 2 Neuropsychology Service, Lincolnshire Partnership NHS Foundation Trust, Lincoln, UK; 3 Department of Anesthesiology, Perioperative and Pain Medicine, Stanford University, Stanford, CA USA; 4 Department of Psychology, Stanford University, Stanford, CA USA; 5 Department of Psychology and Speech-Language Pathology, and Turku Brain and Mind Center, University of Turku, Turku, Finland; 6 Department of Cognitive Neuroscience and Philosophy, University of Skövde, Skövde, Sweden; 7 Department of Philosophy, Monash University, Melbourne, VIC Australia; 8 Monash Centre for Consciousness and Contemplative Studies, Monash University, Melbourne, VIC Australia; 9 Centre for Brain and Behaviour, Department of Psychology, School of Biological and Behavioural Sciences, Queen Mary University of London, London, UK; 10 Institute of Psychiatry at Federal University of Rio de Janeiro – IPUB/UFRJ, Rio de Janeiro, BR Brazil; 11 Research Department at Mobile Brain, Rio de Janeiro, BR Brazil; 12 Human Experience Dynamics Ltd, London, UK

**Keywords:** Human behaviour, Complex networks, Language and linguistics

## Abstract

The language people use to describe their inner experiences arising during sleep and wakefulness provides a window into the mind. Dreams and unguided waking thought, here referred to as mind wandering, are thought to reflect unique individual differences and mental health. Here we use graph theory to analyse a large dataset of dream (*n* = 1434) and mind wandering (*n* = 1619) reports from 176 individuals. We found that dream reports have a more complex structure, while mind-wandering reports have fewer word repetitions and are more verbose, with essential transition points in the structure of the texts. Dream reports, which rely on recall over longer periods of time, tend to have more word repetitions, local cliques, and global integration, comparatively indicating a small world structure. Capitalising on a repeated measures design, we found that the structure of dream and mind-wandering reports contains individual-specific information. Finally, we observed that word centrality (i.e., lower lexical diversity) in dream and mind-wandering reports is predictive of depression and anxiety symptoms, respectively. Thus, this approach is sensitive to individual differences, quantitatively differentiates reports of two different states of consciousness, and seems promising for cost-effective analyses of large naturalistically occurring qualitative datasets.

## Abstract (web summary)

This study applies graph theory analysis to a large dataset of dream and mind wandering reports. It repoerts distinct narrative structures, stable individual patterns and relationships between lower lexical diversity and depression and anxiety symptoms.

## Introduction

The way we organize our thoughts is reflected in how we structure words, themes, and arguments when we speak and write. By examining the spontaneous associations that emerge in narratives, we can observe the ways in which individuals construct their interpretations of specific situations, thereby revealing aspects of their memories and life experiences. This notion underpins the concept of mental organization, which refers to the arrangement of declarative information into complex combinations that convey broader concepts beyond isolated parts.

Classic ideas such as William James’s stream of consciousness^1^ or the word-association methods explored in psychoanalysis have been given an objective methodological framework through natural language processing (NLP) approaches developed over the past 13 years^2,3^.

Conversely, in the context of psychopathological conditions that impact memory and social cognition—such as dementia or psychosis—there is evidence of a loss of these topological properties in word recurrence graphs^2–8^. These findings suggest that graph-based analyses can provide an objective means to access features of mental organization.

Investigating mental organization and event memory involves considering different physiological states of consciousness, such as dreaming and mind wandering. Both states involve introspective narratives that are uniquely experienced and reported by the individual. Here, we investigate the required mental organization to convey these highly personal, witness-free memories in written form.

## Dreaming and mind wandering as continuous and divergent

In the present study, we operationalize mind wandering as unguided waking thought occurring in the absence of a focal external task with prespecified goals, what Li and Hills term unrestricted mind wandering^9^. The literature reviewed draws on a range of operationalizations, including stimulus-independent thought, task-unrelated thought, spontaneous thought, each emphasizing different defining features. The relationship between dreams and the waking mind has been of interest to scholars for centuries. Increasing research efforts have also been directed to mind wandering, which is recognised as a fundamental feature of naturalistic cognition^10^. Of interest is that recently, researchers have proposed that dreaming and mind wandering lie on a continuous spectrum, as both are spontaneous forms of thought^11^. Another approach is the continuity theory, which claims that the content of dreams relates to and reflects waking thought^12,13^.

Both dreaming and mind wandering are most commonly reported as visual and auditory experiences, yet can also be “thought-like”^14^. They both tend to arise spontaneously and often reflect one’s current concerns^15^ and mentation, and rely on mnemonic information (although to different degrees) or provide a simulation of reality^11,14,16,17^. Their neural similarity is supported by the observation that mind wandering and REM sleep broadly recruit default mode network regions. However, dorsal prefrontal regions involved in executive functions tend to be less activated in REM sleep^17^, although there are inconsistencies between studies^18^. Thus, dreaming has been described as an “intensified” form of mind wandering^15,17,19^.

However, whereas dream experiences arise during sleep, mind wandering consists of internally generated, lived experiences during wakefulness. Thus, the two phenomena occur in different physiological and behavioural states and are different states of consciousness^20,21^. Dreams are characterised by a lack of reality testing^22^, greater bizarreness and unstructured content^23,24^, and are often considered a hallucinatory phenomenon^25^. In contrast, mind wandering may involve varying degrees of intentionality and executive control^10,11,19,25,26^. Researchers have even challenged the notion that mind wandering is a unitary phenomenon^26,27^, noting variations in attentional neural systems, intentionality^26,28,29^, affective outcomes^30^, and restrictive contexts^9^.

Similarly, dream experiences are not necessarily a unitary phenomenon. Dreams differ in richness and detail, in lucidity, in the extent they are remembered, in how mundane or bizarre they are, in their frequency and content during different stages of sleep and in how continuous they may be with waking life^14,16,18,31^. Furthermore, although there is a degree of similarity in cortical activation patterns during REM sleep and mind wandering during wakefulness^17^, there are important and somewhat overlooked subcortical differences between REM sleep and wakefulness states^32^. Moreover, the neural correlates of REM sleep cannot be considered to be the neural correlates of dreaming, and thus not directly comparable to the neural correlates of waking mind wandering, given that dreaming occurs also in other sleep stages than REM sleep and not all REM sleep awakenings are accompanied by dream recall^33^.

In the present study, we investigated the organisation of narratives when recalling introspective and personal memories from two different physiological states of consciousness, by characterising the continuity and divergence of dreams and mind-wandering reports.

In this study, we define mind wandering as self-generated, intentional or unintentional unguided thought that can be either stimulus-dependent or stimulus-independent, and comprises all inner experiences (e.g., thoughts, images, memories, plans). Participants were instructed to let their minds wander^26,27^ and write a report of the content of their mind wandering immediately afterwards^26,27^. Similarly to Li and Hills^9^, we focused on unrestricted forms of mind wandering. Participants were instructed to find a distraction-free environment, allow their minds to wander freely from thought to thought without any specific goal, and write a detailed report of whatever had passed through their minds immediately afterwards. This paradigm was chosen to allow longer, more naturalistic reports and maximize comparability with dream reports. It is also analogous to paradigms using resting-state neuroimaging where participants are similarly instructed to their minds wander freely^26,27^.

## Individual differences related to dreaming and mind wandering

An important question is to what extent individual differences (stable characteristics that distinguish individuals from one another) underlie experiences of dreams and mind wandering. In relation to dreams, there is mounting evidence that certain traits and experiences are related to dream content. For example, neuroticism and anxiety show consistent associations with negative dream content^34–37^. This may be driven by waking preoccupations, as individuals who tended to worry more about COVID-19 during the coronavirus pandemic also experienced more negative dreams^38^. Similarly, pandemic dreams were characterised by a higher proportion of negative and semantically relevant content^39–41^. While seemingly less consistent, there are also studies showing that extraversion and openness to experience may relate to dream content^34,35^.

Although waking thoughts about dreams may differ from those had within the dreams themselves^18^, emotional reactions to dream situations are similar between the waking and dreaming states, indicating a degree of continuity^42–44^. Waking reactions to dreams may be thus trait-mediated and an important additional aspect of dream-related experiences. In fact, neurobiological differences are likely to be driving such individual differences in dream experiences^31,34^.

Studies show that mind wandering can be related to both positive^45,46^ as well as negative affect^47,48^. There have been suggestions that mind wandering may be related to rumination^49,50^. In fact, neuroticism has been related to higher frequency and more negative mind wandering^51–53^, although the direction and magnitude of these relationships depend on how mind wandering is operationalized. For example, Kam and colleagues showed that, while unprompted thought is overall negatively associated with affective well-being, this relationship varies depending on whether thought is intentional or unintentional, task-unrelated or freely moving, and how it is measured^30^. Conversely, schizotypal personality traits have been found to be more related to bizarre content^53^. Similarly to above, participants who reported worrying more during the COVID-19 pandemic also had more negative mind wandering^38^. Given a theoretical focus on cognitive control and its failure in some mind wandering research^11,54^, it is perhaps unsurprising that lower executive functioning can predict mind wandering frequency and content^52,53,55^ and may relate to individual differences in intentionality during mind wandering^26–28^. In fact, research has shown that mind wandering occurrences are complex and diverse and can be differentially explained by cognitive abilities and emotional states^55^.

In this study, we investigated whether the organisations of narratives of dreaming and mind wandering are consistent within individuals and dissociable between individuals, via the use of word recurrence graphs^2,3^.

## Dream and mind-wandering reports reflect mental health

Verbal descriptions of one’s experiences may convey information about mental health not captured by other methods^31,56–58^. For example, Sikka et al.^37^ have shown that the content of dreams (as rated by external judges) is more strongly associated with anxiety and peace of mind than self-ratings of dream experiences. In fact, a large number of studies show discrepancies regarding results depending on whether the content of dream or mind-wandering reports or self-ratings of these experiences has been analysed^59–61^. Beyond the aforementioned relationships with trait anxiety^25,34–36,51,53,55,62^, studies have demonstrated that self-reported anxiety and general psychopathology are associated with more negative dream content^37,63,64^.

An important consideration in the development of the word recurrence graph methodology used in this study is the observation that dream reports reveal differences between the narratives of individuals diagnosed with schizophrenia compared to those without such symptoms, while everyday narratives do not show these differences^4,5^. Individuals affective psychotic symptoms, tend to produce more complex and connected narratives when describing dreams compared with reports of waking experiences reports, unlike those with schizophrenia diagnoses^4^.

Interestingly, acute psychological distress—such as that caused by social isolation during the COVID-19 pandemic—can profoundly alter dream content (e.g., increased expressions of sadness and anger, contamination and cleaning themes) compared to pre-pandemic dreams by the same individuals, yet does not change the topological structure of dream narratives^65^. This suggests that such structural features may reflect stable individual characteristics.

Mind wandering, although sometimes associated with positive affect^45,46^ has also been related to negative mood, and has been proposed to be potentially be related to anxiety and depression^11,17,38,47,48^. Due to difficulties in sustained attention, Attention Deficit Hyperactivity Disorder has been hypothesised to be characterised by increased mind wandering^11^. However, individuals in this group showed no differences in mind wandering frequency compared to a control group^51^. Nonetheless, as suggested above, there are consistent reports that suggest a relationship between mind wandering and trait anxiety^51–53^.

Differences in writing style have also been shown to hold information that is predictive of depression and anxiety^66–71^. Thus, all the above suggest that reports of dreams and mind wandering may not only reflect, but possibly also predict, mental health.

In this study, we thus investigated whether the organisation of mind wandering and dream report architecture^3^ may relate to self-reported symptoms of anxiety and depression.

## The present study

In the present study, we applied graph theory to investigate the thought organisation structure of dream and mind-wandering reports. We employed a unique and large dataset featuring multiple repeated measures of dreaming and mind wandering (1619 and 1434 reports, respectively, from a total of 176 individuals).

The data we use are written reports derived from delayed recall of the experiences in question (dreams and mind wandering). Thus, our analyses pertain to the reports of dreaming and mind wandering rather than the experiences themselves.

Using graph theory analysis, our aim was to (1) compare the structure of dream and mind-wandering reports; (2) explore the within- and between-person variability of these reports; and (3) investigate whether the structure of these reports can predict self-reported clinically relevant mental health symptoms (i.e., symptoms of anxiety and depression). With this technique we aim to explore word recurrence patterns in natural writing with little preprocessing of the data, in accordance with the framework described above (see Mota et al. 2023 for further info.)

**Fig. 1:**
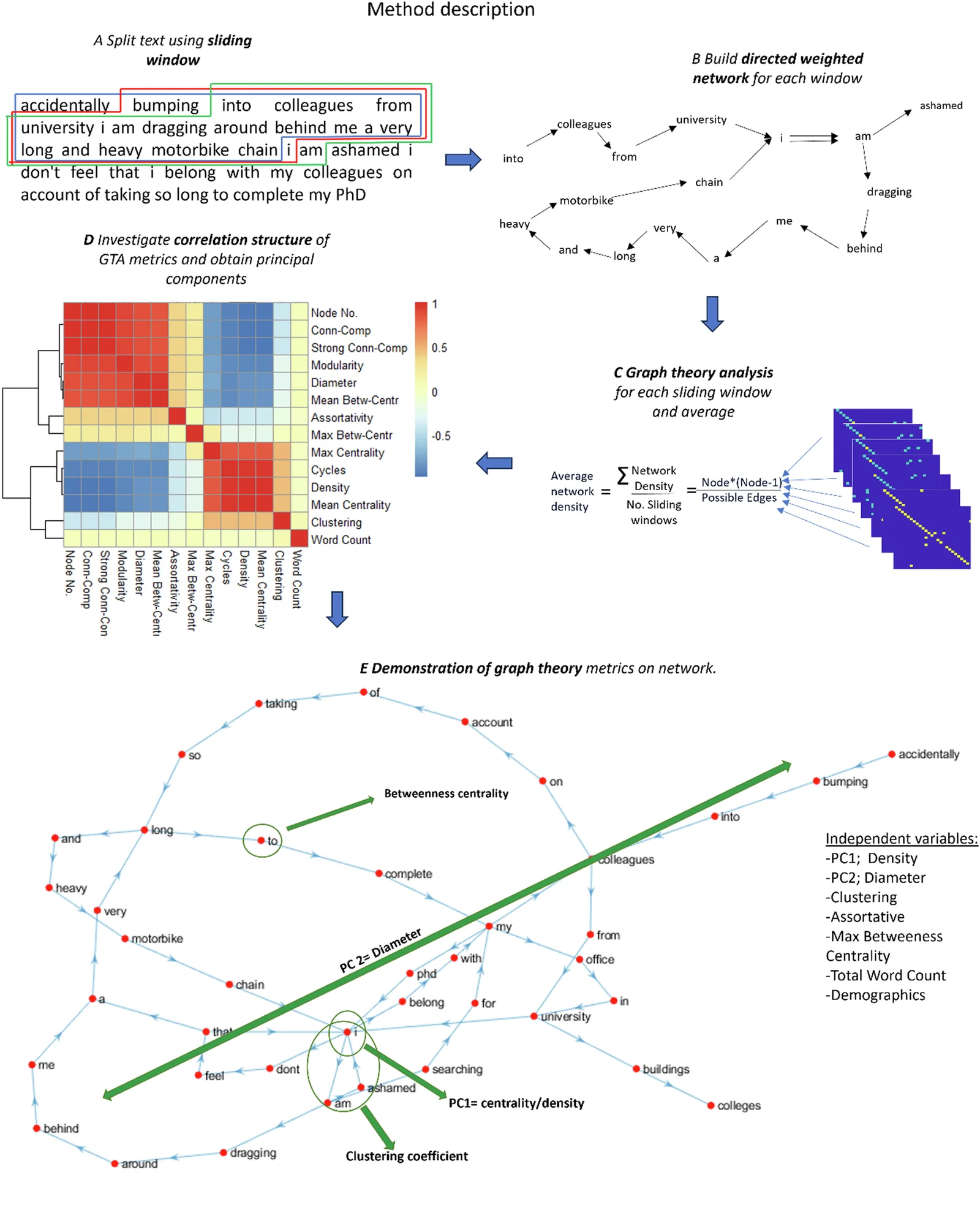
Description of network methodology. Panel **A** After having pre-processed each dream and mind wandering report (e.g., removing punctuation and capital letters), we applied a sliding window algorithm where we selected 30 words and slided the window by one word until the entire report was exhausted (the figure is for illustration purposes only). Panel **B** For each of the sliding windows, we built a weighted directed network. This was done by connecting words that were adjacent to each other (in left to right direction). Repeated words were considered the same node in this network. Panel **C** For each of these networks (shown as stacked matrices), we calculated relevant graph theory properties (shown in matrix in the following panel; here only density is displayed). Panel **D** We investigated the correlation structure and reduced multicollinearity by extracting principal components based on the highly correlated variables (Pearson’s r > 0.5, Supplementary Material 1). Word count was not associated with other variables, indicating that the sliding window method successfully controlled for network size in calculating graph theory metrics. Panel **E** Illustration of a speech graph network and different graph metrics. Shown are the network diameter feature (captured by principal component 2) indicating the “ease of travel” or distance between the farthest points in the network, centrality, (i.e., highly repeated words, captured by principal component 1), betweenness centrality (words that connect different parts of the network) and clustering (neighbouring words tend to also be neighbours, forming cliques). Not shown are assortativity (nodes tend to connect to similarly connected nodes) and word count (total number of words in original report). Final independent variables for the hypotheses are shown to the right of panel (**E**). Betw-Centr betweenness centrality, Conn-Comp connected component, Node no. Node number after sliding window, PC Principal Component. See Table 2 for further descriptions.

In the pre-registration (https://osf.io/arx2h), we put forward the following hypotheses. In accordance with previous literature^16,23,24,72^, for the first hypothesis (H1), we predicted that dream reports would be more unstructured than mind-wandering reports as they may be more bizarre and lack continuity in narrative structure and be less amenable to cognitive control. We therefore predicted dream reports would have a more modular/segmented and hierarchical structure. Furthermore, in the pre-registration, we also predicted that there would be higher verbosity in mind-wandering reports due to recency effects and ease of recall^73^, but also a difference in word repetition and related metrics. However, we specified that, despite the a-priori predictions above, the specific network properties tested would depend on data reduction results (Fig. 1).

For the second hypothesis (H2), in accordance with previous^4,7,10,34,38,40,42,45,58,74^, we expected that within-participant similarity of text network structures will be higher than between-participant similarity. This would suggest that, indeed, this approach is sensitive to individual differences and therefore may display predictive power.

For the third hypothesis (H3), given the links between dream and mind-wandering reports and mental health, we expected that graph theory structures of text would predict self-reported anxiety and depression symptoms.

We expected a high level of multicollinearity between different graph theory measures^75^. As such, we specified in the preregistration (https://osf.io/arx2h) that we would use a data reduction technique to find metrics that had unique explanatory power (i.e., did not display collinearity). This would ensure that statistical models were tractable and robust.

## Methods

### Participants

We collected the data in the framework of a larger study^38^ from Australia and the United Kingdom between July and November 2020. We used social media advertisements and press releases to recruit the participants. In total, 294 participants provided 2604 dream reports, whilst 261 individuals provided 1767 mind-wandering reports. Data were excluded if participants did not declare to be above 18 years of age, if they did not reside in the UK or Australia, did not give consent to participate or did not answer within the above stated timeframe. If multiple entries were given within the same day, only the evening mind-wandering reports were included, whilst duplicate entries were deleted. After excluding the above, 1781 dream reports from 172 participants (25 men, 139 women, eight “other”; mean age 46.99 ± 14.80 years, range 19–78) and 1496 mind-wandering reports from 152 individuals (23 men, 123 women, 6 “other”, mean age 48.42 ± 14.71 years, range 19–78) remained. In accordance with the graph theory method used (see below), reports that did not result in a minimum of 30 words were excluded. Additionally, an outlier observation was removed to ensure that statistical assumptions were met. As a result, in the present study we analysed 1619 dream reports (172 participants) and 1434 mind-wandering reports (152 participants) from a total of 176 individuals. Of these, 109 were from the UK, 66 from Australia, whilst one reported to reside in both countries. No other demographic variables were collected. The study was carried out in accordance with the Declaration of Helsinki and approved by the Cambridge Psychology Research Ethics Committee and the Monash University Human Research Ethics Committee.

As expected with such naturalistic data, there were large variations in the amount of data each individual provided (max dream reports = 28, max mind-wandering reports = 40, minimum for both types of reports = 1). Further information is available in https://osf.io/arx2h and Sikka et al. 2024.

### Procedure

Participants completed the study on a webpage (https://sites.utu.fi/mind/en). They were required to read through the information regarding the study and were provided with a randomly generated participant ID. They were required to be above 18 years of age and provide informed consent. After completing relevant self-reported measures, they would provide dream and mind-wandering reports over a two-week period. Specifically, they were asked to complete daily dream logs every morning immediately after waking up. They were asked to report their dreams with as much accuracy and detail as possible. They were also asked to write a mind wandering log every day for two weeks. They were asked to find a distraction-free environment and let their minds wander from thought to thought without any specific goal. After a 10-min period, they were instructed to write down everything that went through their minds (thoughts, feelings, images, scenarios) as clearly and accurately as possible. We thus used an unrestricted form of mind wandering allowing longer and more naturalistic reports and greater comparability with the dream reports^38^ (https://osf.io/su7mn/). Participants received no compensation for their participation.

### Network construction and correlational structure of graph theory metrics

The present work was preregistered (https://osf.io/arx2h), which contains a brief literature review as well as proposed methods and hypotheses.

The networks were created by connecting adjacent words (Fig. 1B). Where, in the report, there were two or more instances of word sequences (i.e., in the relevant text as per sliding window, the same words were adjacent two or more times), the corresponding cell in the matrix (representing the graph theory network) was filled with the total instances of the sequence, thus creating a weighted network.

Following Mota et al.^2–4,74,76^, we processed the dream and mind-wandering reports to create graphs (i.e., networks). We created a MATLAB script that would reproduce the output of the speech graphs software that was used in previous research^3,4^. To do this, all words were converted to lower case and punctuation (including apostrophes) was removed. The script returns matrices that represent graphs/networks and is *available at* https://github.com/Peter6789/graph-theory-analysis-of-text/tree/main. Of note is that the implementations nonetheless differ in some ways, in that in the speech graphs software paragraphs would provoke disconnected components, whilst this is not the case in the present implementation. Furthermore, measures of Largest Connected Component and Strongest Largest Connected Component in the NetworkX implementation (https://networkx.org/) yielded near perfect collinearity with this type of graph, whilst this is not the case in the speech graph software (Mota et al. 2012, 2014, see Supplementary Material 1 for further information).

Importantly, to ensure that the word count (report length) would not influence graph theory properties^3,4,75^, we applied a sliding window approach where 30 words (as sequentially found in the report) were selected and a network created out of those. The window was slided by one step (i.e., one word) and the process repeated until that instance of text was exhausted. Thus, no windows are perfectly the same but are overlapping with all adjacent windows. The averaging ensures the ensuring metric is representative of the report whilst controlling overall verbosity. Given the lack of correlation between the total word count of the report and all network properties (*average r* = *0.025*), this approach was successful in controlling for network size (which has a substantial effect on most graph theory measures).

One of the most fundamental sources of variation in these types of graphs (primarily measured by PC1 in the results) is the repetition of words in the sliding window, which would result in one node (i.e., word) having multiple connections in different parts of the report (see Mota et al.^4^; https://neuro.ufrn.br/softwares/speechgraphs, as well as Supplementary Material 2 and Github). Thus, repetition of words strongly drives density, number of connected components, centrality, modularity, numbers of cycles etc., (Fig. 1D), whilst the second principal component tends to represent how extended, overall, the network is (how easy is it to get from one side of the network to the other), somewhat indicating the “integration” of the report with itself rather than a non-recurrent linearity.

We thus obtained several networks for each report by using the sliding window approach explained above. Accordingly, the number of sliding windows depended on the total number of words for each text and were subsequently averaged to ensure that the word count did not have undue influence on the graph theory metrics. These networks, as obtained via the sliding window methodology, were then transferred into python for graph theory analyses as the package NetworkX (https://networkx.org/) provides exhaustive and robust analyses. Information with all relevant details of graph theory analyses can be viewed in Supplementary Material 1. Given the inherently directional nature of the graphs constructed, (i.e., one word sequentially leading to another), where the specific graph theory metric allowed, the directed (asymmetrical graph) network was used as an input. Otherwise, a non-directed network was used (Supplementary Material 1).

In the preregistration (https://osf.io/arx2h) we attributed interpretations to such measures (post hoc decisions are noted in Supplementary Material 1). Although there has been previous work on such text derived graphs^3,4,75^, as per preregistration, the initial analyses were exploratory as the explicit extent of correlations between all the measures in this dataset was unknown. This exploration was necessary to learn what measures may have independent predictive power in the following hypotheses (see preregistration) and avoid collinearity.

Briefly, the metrics obtained were diameter, density, node number, assortativity, cluster coefficient, mean degree centrality, max centrality, characteristic path length, modularity, number of cycles, number of bridges, largest connected component and strongly connected component, mean and maximum betweenness centrality (see Supplementary Material 1 for more details). These data were imported into R for further statistical analyses. We found a high level of skewness in word count, clustering coefficient, bridges, mean betweenness centrality, characteristic path length. Log transformation of these metrics improved the skewness to acceptable levels (values from “skewness” function in R, measuring of the extent of deviation from symmetry of a frequency curve from X > 1 to X < 1, Supplementary Material 2). Subsequently, a correlation matrix revealed that maximum-centrality, number of cycles, density, mean degree centrality, diameter, mean betweenness centrality, modularity, node number, largest connected component and strongly connected component were all highly correlated (R ~ 0.7–1) (Fig. 1D*)*. Thus, these measures were inserted in a principal component analysis and three principal components extracted. First, however, we removed characteristic path length, it being highly related mathematically to the diameter and mean betweenness centrality measure. The first principal component captured 91% of the variation in the inserted data, the second 3.9%., whilst the third 2.8% (details in Supplementary Material 2). In the first PC, the variables loaded more-or-less equally, as implied by the high explained variation. Mean degree centrality and density had the highest loadings indicating the repetition of words was the main contributor to this principal component (Table 1). In the second component, diameter and mean betweenness centrality (also based on path length, i.e., distance between two nodes) had the highest loadings. We decided to interpret this primarily in terms of diameter as this is more intuitive and avoids confusion with another metric which is maximum betweenness centrality. The final PC was primarily driven by max centrality, however, as explained below, this component could not improve inferential statistical models and therefore was not included in the final analyses. This analysis was repeated averaging all within participant repeated measures. No major differences in the data reduction results were found.

**Table 1:** Variable inserted in the PCA with loadings onto the principal components (sign also displayed)

	PC1	PC2
Diameter	0.306	−0.546
Density	−0.326	−0.239
Node number	0.324	0.264
Mean centrality	−0.326	−0.239
Maximum centrality	−0.285	0.045
Modularity	0.313	−0.114
Cycles	−0.320	0.045
Largest connected component	0.324	0.264
Largest strongly connected component	0.324	0.264
Mean betweenness centrality	0.305	−0.600

Highlighted in BOLD are the highest loadings onto PC1 and PC2.

**Fig. 2:**
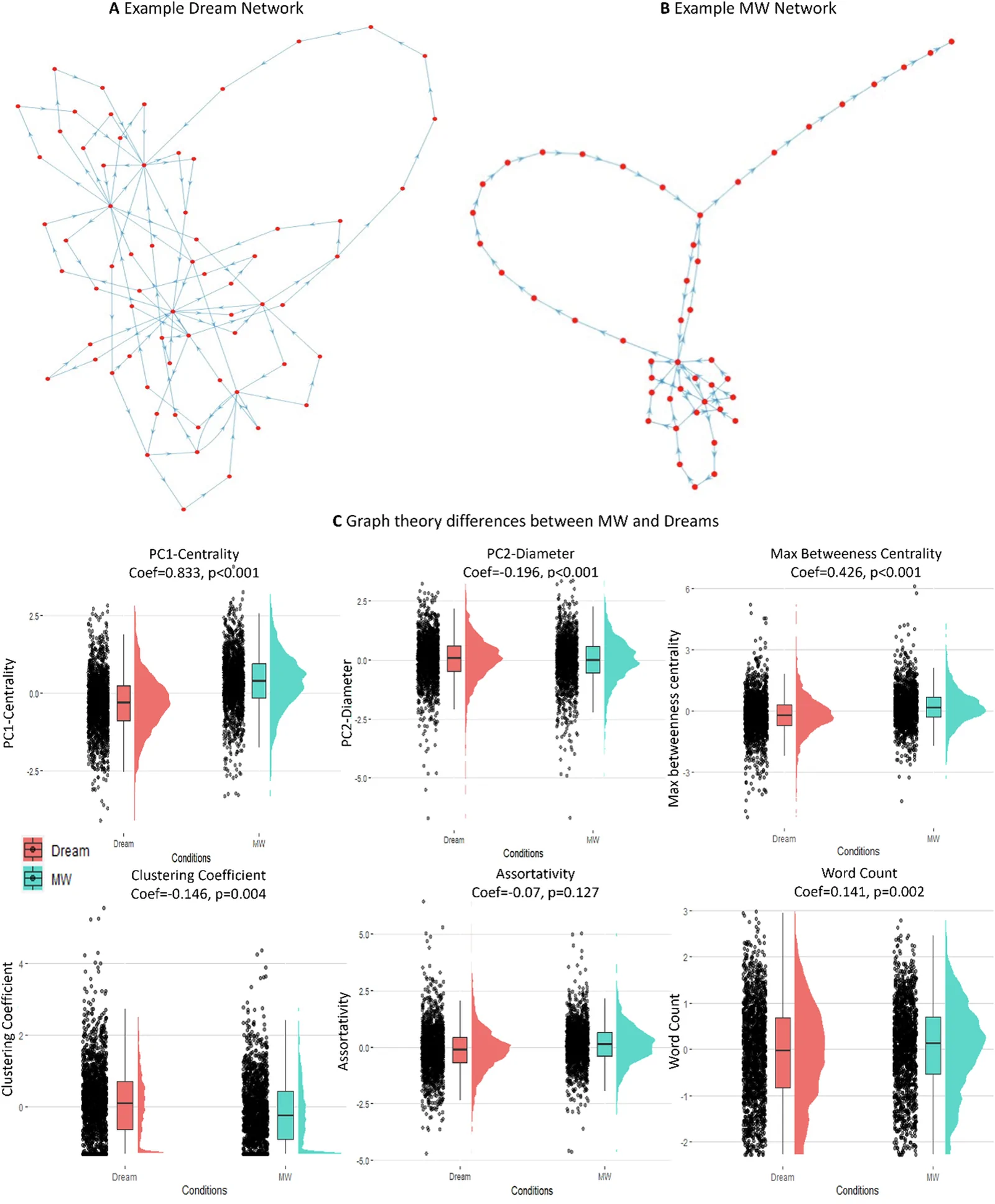
Dream and mind-wandering reports have different network structures. Panel **A** shows an example network structure of a dream report. Panel **B** shows an example of network structure of a mind wandering report. Panel **C** displays significant graph theory metrics (regression coefficients and non-corrected *p*-values) in the form of rain cloud plots. Bars represent interquartile range. Shown are values for centrality (PC1), diameter (PC2), max betweenness centrality, clustering coefficient and word count. Note that the dream report network (panel **A**), as compared to the mind wandering report network (panel **B**), tends to have lower lexical diversity (PC1), more ease of communication (PC2), less essential nodes to connect different parts of the network (Betweenness centrality), and more connections between neighbours (clustering coefficient). Overall, the structure of dream reports tended to be more complex. In panel (**C**), violin plot of differences between metrics. Please note centrality and diameter had negative loadings onto PC1 and 2, respectively, see S2 or methods section). PC1: coef. = 0.83, 95% C.I. = 0.72:0.94, *p* < 0.001. PC2: coef. = −0.20, 95% C.I. = −0.29: −0.10 *p* < 0.001). Verbosity: coef. = 0.14, 95% C.I. = 0.051:0.23, *p* = 0.002. Maximum betweenness centrality: coef. = 0.43, 95% C.I. = 0.33:0.52, *p* < 0.001. Clustering: coef. = −0.146, 95% C.I.= −0.25: −0.05, *p* = 0.005. 1619 dream reports from 172 participants and 1434 mind-wandering reports from 152 participants where analysed. Total participants = 176.

Loadings of the relevant variables were negative onto PC1 and PC2 (Table 1). The clustering coefficient had a floor effect, by which a certain number of reports (14.3%) had zero in this metric. We nonetheless kept this metric as it still might have displayed independent variance, which was of relevance to the hypotheses (distributions and results available in Fig. 2C).

The first PC was therefore interpreted as representing centrality, repetition of words, whilst the second PC denoted network diameter (i.e., distance between one side of the network to the other). To improve adherence to statistical assumptions in the logistic mixed model regression, one outlier in PC1 was removed.

**Table 2:** Descriptions of graph theory metrics that are relatively independent from one another

Metrics	Description
PC1-Centrality	This is sensitive to lexical diversity. How dense are the graph connections? Are there more nodes (words) connected with fewer possible pathways, i.e. higher lexical diversity connected through fewer possibilities of pathways, fewer repetitions of central words? (Note: this aspect of networks explains other graph theory measures (see methods section and S2 for further details)).
PC2-Diameter	How far away are the two extremes of the network? Does it take many “steps” to get from one side of the graph to another? How linear are word trajectories? Reveals lower connectedness between distant words, fewer repetitions that do not connect large amounts of words (probably repeating locally in the sentences).
Clustering Coefficient	Are there “cliques” or clusters in the report? The extent to which neighbours of one node are also neighbours amongst themselves. How the words connected to another word, are also connected to each other.
Maximum betweenness centrality	Are the points in the network through which lots of paths tend to go through? Are there essential “bridges” in the report? Are there important words that connect a variety of other words in the narrative?
Assortativity	Are highly connected words also connected to highly connected words? Are minimally connected nodes connected to minimally connected nodes? Are repetitive words linked in sequence to each other?
Report length/verbosity	How many words are in the report? What would be the total number of edges if the sliding window approach were not used?

### Statistical analyses of the network architecture difference between dream and mind-wandering reports

We reduced the data via the above described principal component analysis to ensure that predictor variables had sufficient independent variance to avoid multicollinearity issues (NB: inclusion of both individual graph theory metrics and reduced graph theory metrics was not explicitly stated in the pre-registration). We subsequently constructed a mixed effect logistic regression (glmer from the R package lme4) with subject ID defining the random intercepts. As per preregistered hypotheses, we investigated which variables would predict whether the text denoted a mind wandering or a dream report. Following preprocessing described above, we included PC1-centrality, age, country, gender, word count, assortativity, maximum betweenness centrality, and clustering coefficient in the statistical model. We also constructed two additional models, one adding PC2-diameter, and another adding the third principal component in addition to the previously mentioned predictors. Through model comparison using the R “anova()” function we found that the model with PC1-centrality and PC2-diameter provided the better fit (considering predictive power and parsimony, Supplementary Material 3). In fact, the Akaike information criterion was 3716.2 for the model without PC2 and 3700.9 for the model with PC2 (note the procedure for including variables of interest was not specified in the preregistration). We thus report the results that have PC2 as a predictor variable. We used the DHARMa to test assumptions and found these were not violated (when removing an outlier in PC1). We found that variance inflation factors (using the R function VIF()) of the independent variables were all below 1.5, indicating low correlation between these.

Of note is that we predicted that graph theory analyses would show that dreams are more unstructured. However, due to a lack of a straightforward operationalisation, beyond the high level of covariance found between the metrics, this hypothesis could not be tested in a way that was conducive to univocal interpretations. Due to high collinearity, we could not test differences in modularity and hierarchy (these having high collinearity with other measures and being reduced in the principal component analysis. This possibility was specified in the pre-registration).

In sum, we used network properties from 3053 texts from 176 participants. All variables were scaled (mean centred and divided by standard deviation) before they were entered into statistical models. Further descriptive statistics of the variables of interest are presented in Supplementary Material 7 (distributions are presented in Fig. 2C). Analyses were two-sided. Data met statistical assumptions.

### Individual-specific information in network structure

As per preregistered hypotheses (https://osf.io/arx2h, first proposed method used), we tested whether the report structure contained individual-specific information. These analyses capitalised on the repeated design structure of the data.

For each report, we created a vector containing the scaled network properties that had a sufficient independent variance (r < 0.5, *see methods and* Supplementary Material 2). In the vector we inserted clustering, word count, maximum betweenness centrality, assortativity, PC1-Density, PC2-Diameter. This vector thus gave a 6-dimensional description of the network structure of each report.

We then used city block distance metric (a.k.a. Manhattan distance) to investigate the difference (or distance) between each vector describing the topology/architecture of the report. We compared the distance (i.e., inverse of similarity) between each report and categorised them according to whether we compared the same of different types of report (dream and mind-wandering reports), and whether the comparisons were within or between individuals.

In total, there were 9654 within individual comparison for dreams and 8250 for mind-wandering reports. There were 16,888 within-individual comparisons between dream and mind-wandering reports. As for between individual comparisons there were 1,300,117 for dreams reports and 1,019,211 for mind-wandering reports and 2,304,758 between dreams and mind-wandering reports (Supplementary Material 7). We investigated if the reports written by the same person were more similar compared to the reports from other individuals using a non-parametric permutation approach. This involved randomly shuffling condition labels (e.g., within vs between person similarity) 1000 times to create a null distribution from the data. This null distribution was then compared to the test statistic (difference between medians). Due to the highly unbalanced sample size, each permutation involved a random sampling of the condition with the highest number of observations so as to match to the number of observations in the lowest condition. This ensured that the null distribution was appropriately defined. We present the median differences of robust z-scored values (using median absolute deviation and median) to enable ready interpretation of the effect size. We also present the robust Cohen’s d; the Cohen’s d and 95% confidence intervals statistics calculated using the Matlab meanEffectSize function.

The temporal distance between dream and mind-wandering reports was calculated via the distance in days between instances of reports. This distance was then correlated with the network similarity of the report, testing the continuity hypothesis.

Analyses of the relationship between graph theory structures of reports and depression and anxiety symptoms.

Participants completed a well-being questionnaire containing the Patient Health Questionnaire-9 (PHQ-9) and the Generalised Anxiety Disorder Assessment (GAD-7). They were then asked to provide dream reports every morning upon awakening and then engage in a mind wandering task and provide mind-wandering reports every evening for a two week period. See Sikka et al.^38^ for more details.

**Fig. 3:**
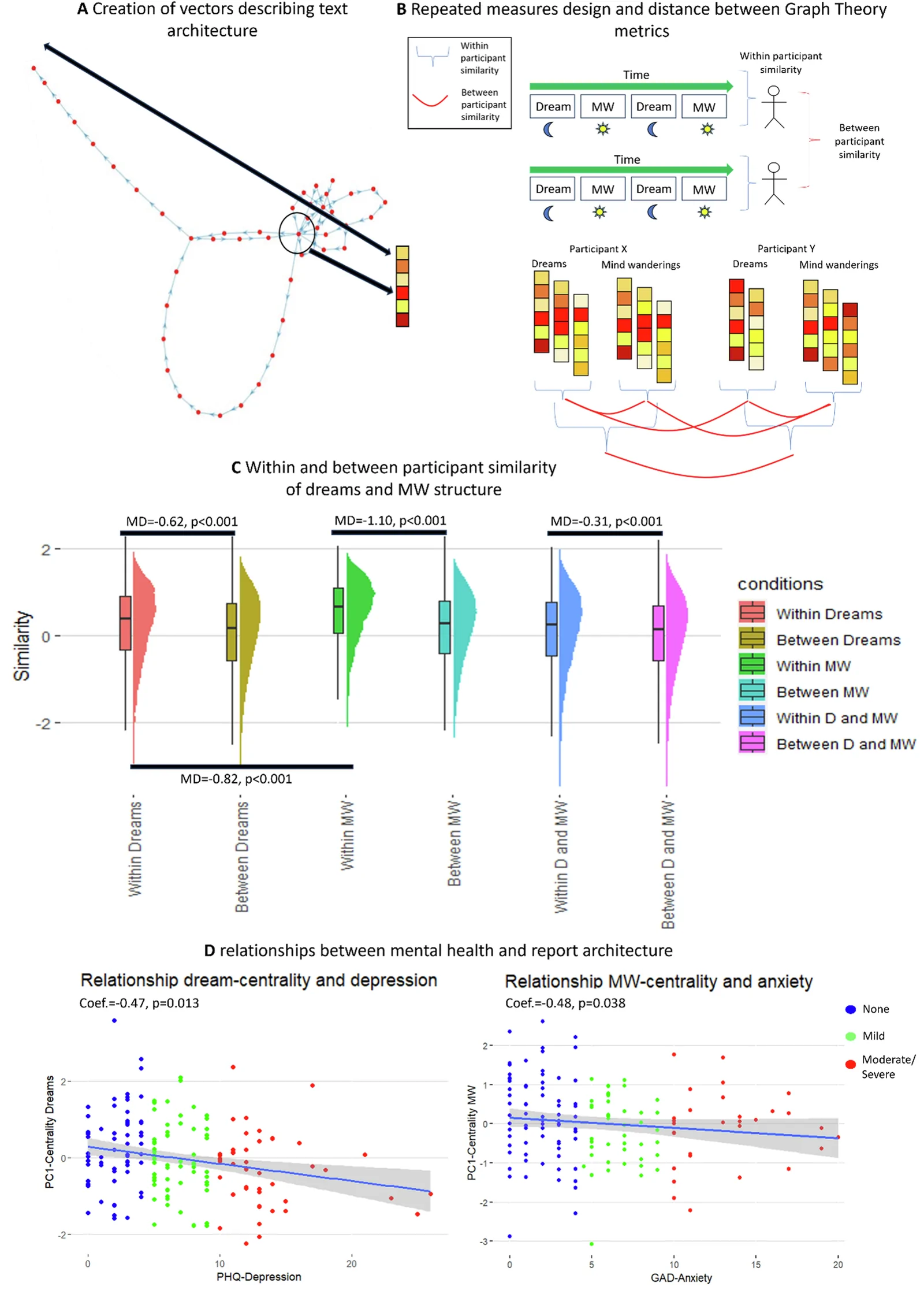
Individual differences in the structure of dream and mind-wandering reports. Panel **A** Demonstration of how different graph theory metrics (shown are centrality and diameter), are inserted into a 6-element vector which characterises the structure of the network (these are composed of the variables found to explain independent variance as shown in Fig. 1D, see Table 1). The vector is comprised of PC1-centrality, PC2-diameter, maximum betweenness centrality, assortativity, clustering coefficient, and word count. Panel **B** shows how the different texts are compared within and between individuals as well as within and between network type in accordance with the hypotheses. Panel **C** shows the within-and between-participant similarities of dream and mind wandering report structures (statistics shown are from Mann–Whitney U tests). Bars represent interquartile range. Horizontal black bars indicate which conditions were compared with the relevant statistic above. Within-participant similarities between dream and mind-wandering reports are greater than between-participant similarities. Within-participant similarities in mind-wandering reports were greater than within-participant similarities in dream reports. Within individual dream and mind wandering comparisons, *n* = 9654 and 8250, respectively. Within individual between dream and mind wandering comparison *n* = 16,888. Between individual dream and mind wandering comparisons, *n* = 1,300,117 and 1,019,211, respectively. Between individuals, between dream and mind wandering comparisons *n* = 2,304,758. Panel **D**. Relationship between graph theory measures and GAD-7 and PHQ-9 (anxiety and depression, respectively) scales. Note, measures were averaged in a participant-specific manner. Line of best fit and standard error, for illustration purposes only, was fitted with a robust linear model, thus is less influenced by outliers. For both GAD-7 and PHQ-9, blue dots indicate individuals with no symptoms (0–5), green mild symptoms (4–9) and red moderate to severe symptoms (10 or more). MD = median difference. For GAD-7 score (coef. = −0.48, [95% CI = −0.94, −0.02], *p* = 0.038, Odds Ratio = 0.62, df = 11). For PHQ-9 score (coef. = −0.47, [95% CI = −0.84, −0.10], *p* = 0.013, Odds Ratio = 0.62, df = 11). *N* = 176.

We sought to investigate the predictive power of the graph theory metrics using self-reported depression and anxiety symptoms, measured using the Patient Health Questionnaire 9 (PHQ-9) and Generalised Anxiety Disorder Assessment (GAD-7) scales (Kroenke et al. 2001; Spitzer et al. 2006) as outcome variables. Given these data are ordinal and there was not a good representation on the more severe end of the scale, we split the data in different levels to be able have a more comparable number of datapoints at each level. Therefore, we used the cut-off values (to define ordinal levels of anxiety-depression) that would minimise the difference between ordinal categories. For both the PHQ-9 and GAD-7, scores from 0 to 4 represented “no depression/anxiety”, from 5 to 9 “ mild depression/anxiety”, from 10 to the highest score “moderate to severe depression/anxiety”^77,78^ (see horizontal axes in Fig. 3E for distribution of the data).

We ran ordinal logistic multiple regressions, as multi-level models were not feasible due to violated assumptions (tested with DARHA r package as above). To this effect, we averaged data in a participant specific manner. This reduced data points from 3053 to 176. Given the reduced power, we ran separate ordinal logistic multiple regressions for the dream and mind-wandering reports with the two outcome variables separately (GAD7 and PHQ9). The variables included were word count, max betweenness centrality, assortativity, PC1-centrality, PC2-diameter, clustering coefficient, country, gender and age. Analyses were two-sided. Variance inflation factors indicated low collinearity (all below the value of 2.1)

Contrary to our predictions (https://osf.io/arx2h), we did not find evidence of verbosity, betweenness centrality and path length predicting the relevant outcome variables. Modularity, similarly to above, was found to have collinearity to other independent variables (Fig. 1D, Supplementary Material 2) and thus we could not test whether this would be related to self-reported mental health symptoms. Data met statistical assumptions.

Full results are presented in the Supplementary Materials.

## Results

### Description of graph theory of natural language approach

To analyse the flow of thoughts during the narration of a dream or mind wandering within a word recurrence graph, we move beyond simply considering verbosity or the frequency of repetitions. Instead, we investigate the complex features of the trajectories used to connect words that collectively express these experiences. Drawing an analogy to a map in which each point represents a city, we can quantify the number of cities in each map—analogous to verbosity—and the number of visits to each city—comparable to word repetitions. However, when applying word recurrence graphs, we characterize the complexity of the routes taken to travel from one city to another, revealing whether the paths are more linear or more interconnected. Certain measures describe how well-connected specific cities are to others, the density of routes linking all cities, and the distances between the most distant cities given the possible pathways. These topological metrics capture the complexity of different ways in which memories of dreams or mind wandering are expressed, organizing the sequence or flow of words to reflect underlying cognitive processes. Using this method, we tested whether dream and mind-wandering reports differed in network structure^4^.

### Hypothesis 1: Network architecture differences between dream and mind-wandering reports

A mixed-effect logistic regression showed that higher PC1 or lower density/centrality (i.e., higher lexical diversity); coef. = 0.83, 95% C.I. = 0.72:0.94, *p* < 0.001), lower PC2 or higher diameter (more difficult to get from one side of the text to the other, or longer structures) (coef. = −0.20, 95% C.I. = −0.29:−0.10, *p* < 0.001), and higher verbosity (coef. = 0.14, 95% C.I. = 0.051:0.23, *p* = 0.002) had a significantly higher probability of characterising mind-wandering reports. Maximum betweenness centrality, indicating the presence of words that connect different parts of the text (via shortest paths), was also more likely to characterise mind wandering over dream reports (coef. = 0.43, 95% C.I. = 0.33:0.52, *p* < 0.001). Dream reports, on the other hand, tended to have more clustering (i.e., words forming triangles, thus forming a “clique” or cluster of words) (coef. = −0.146, 95% C.I. = −0.25:−0.05, *p* = 0.005). Statistical details are presented in Table 3 and Supplementary Material 3.

**Table 3:** Difference between dream and mind-wandering reports as per mixed model logistic regression (mind wandering> dreams)

	Coeff	95% C.I.	Odds ratio	Std. error	*z* value	*P*-value
Intercept	−0.12	−0.45, 0.21	0.89	0.17	−0.70	0.483
PC1-Centrality	0.83	0.72, 0.95	2.30	0.06	13.98	<0.001
PC2-Diameter	−0.20	−0.29, −0,10	0.82	0.05	−4.15	<0.001
Age	−0.05	−0.18, 0.08	0.95	0.07	−0.74	0.460
Country	−0.15	−0.40, 0.10	0.86	0.13	−1.19	0.233
Gender	0.03	−0.27, 0.32	1.03	0.15	0.17	0.861
Word Count	0.14	0.05, 0.23	1.15	0.05	3.08	0.002
Assortativity	−0.07	−0.17, 0.02	0.93	0.05	−1.53	0.127
Max betweenness centrality	0.43	0.33, 0.52	1.53	0.05	9.06	<0.001
Clustering coefficient	−0.15	−0.25, −0.05	0.86	0.05	−2.91	0.004

*C.I.* Confidence Intervals.

Thus, mind-wandering reports were less dense with higher lexical diversity and presented longer trajectories/structures. They were verbose (had more words), with essential transition points (higher max betweenness centrality) in the text structure (e.g., words connecting different parts of the report). Conversely, dream reports tended to have a more complex structure with small world characteristics (i.e., high clustering and short path length^71^). As such they were characterised by local cliques/clusters, global integration as well as high word repetitions. It seems that in dream recall, a higher number of repeated words connects the reports over long and short distances, whilst the more verbose mind-wandering reports develop into longer structures. There seems to be two diverse patterns of mental organization expressed in the narrative when memory encoding occurred during waking or sleep stages (Fig. 2).

### Hypothesis 2: Individual differences in dream and mind-wandering reports

To test whether text-derived network properties contain individual-specific information, we built a vector with six elements summarising the network structure of a specific report (including density/centrality (PC1), diameter (PC2), clustering coefficient, betweenness centrality, assortativity, and word count, Fig. 3A). We exploited the repeated-measures design of this study and investigated whether the vectors (describing report structure) displayed more similarity within individuals than between individuals; thus, indicating that these measures are sensitive to individual specific variation (Fig. 3B). We quantified similarities via the Manhattan distance (i.e., inverse of similarity) metric between each vector encapsulating the network structure of each report and tested differences via a permutation test.

We found that the structure of dream (median difference = −0.62, *p* < 0.001, standardized median difference = −0.33; robust Cohen’s d = −21; Cohen’s d = −21, C.I. = −0.23:−0.19) and mind wandering (median difference = −1.10, *p* < 0.001, standardized median difference = −0.65; robust Cohen’s D = −0.42; Cohen’s d = −0.38, C.I. = −0.41:−0.36) reports were more similar within individuals than between them. Thus, the network structure of dream and mind-wandering reports does contain individual-specific information (Fig. 3C). Similarly, we found that dream reports were more similar to mind-wandering reports when written by the same individual than when written by different individuals (median difference = −0.31, *p* < 0.001, standardized median difference = −0.17; robust Cohen’s d = −0.11, Cohen’s d = −0.10, C.I. = −0.11:−0.08).

Finally, within-individual similarities of mind-wandering reports were higher than within-individual similarities of dream reports (median difference = −0.82, *p* < 0.001, standardized median difference = −0.51; robust Cohen’s d = −0.34; Cohen’s d = −0.30, C.I. = −0.33:−0.27). This indicates higher within-individual variation in dream reports (i.e., higher complexity) (Fig. 3C) and indicates a diverse pattern of mental organization to narrate memories encoded during sleep. Although not specified in the preregistration, we also investigated whether the similarity between an individual’s dream and mind-wandering reports was higher than similarities between dream reports from different people. We indeed found that the within-person similarity across dream and mind-wandering reports was higher than between-person dream similarity (median difference = −0.20, *p* < 0.001, standardized median difference = −0.11; robust Cohen’s d = −0.08, Cohen’s d = −0.07, C.I. = −0.09:−0.06), further indicating an individual specific stability of mental organization pattern expressed in writing style and phenomenology compared to that of between person dreams.

We investigated whether there was a correlation between the similarity between dream and mind-wandering reports and how close they were in time. For this hypothesis, we did not find statistically significant evidence (Kendall’s Tau = 0.003, *p* = 0.51).

### Hypothesis 3: Relationship between graph theory structures of dream and mind-wandering reports and depression and anxiety symptoms

We subsequently explored how the architecture of dream and mind-wandering reports predicts mental health symptoms, specifically, self-reported measures of anxiety (GAD-7) and depression (PHQ-9). We averaged across repeated measures to yield one value per individual and conducted analyses for dream and mind-wandering reports separately.

**Fig. 4:**
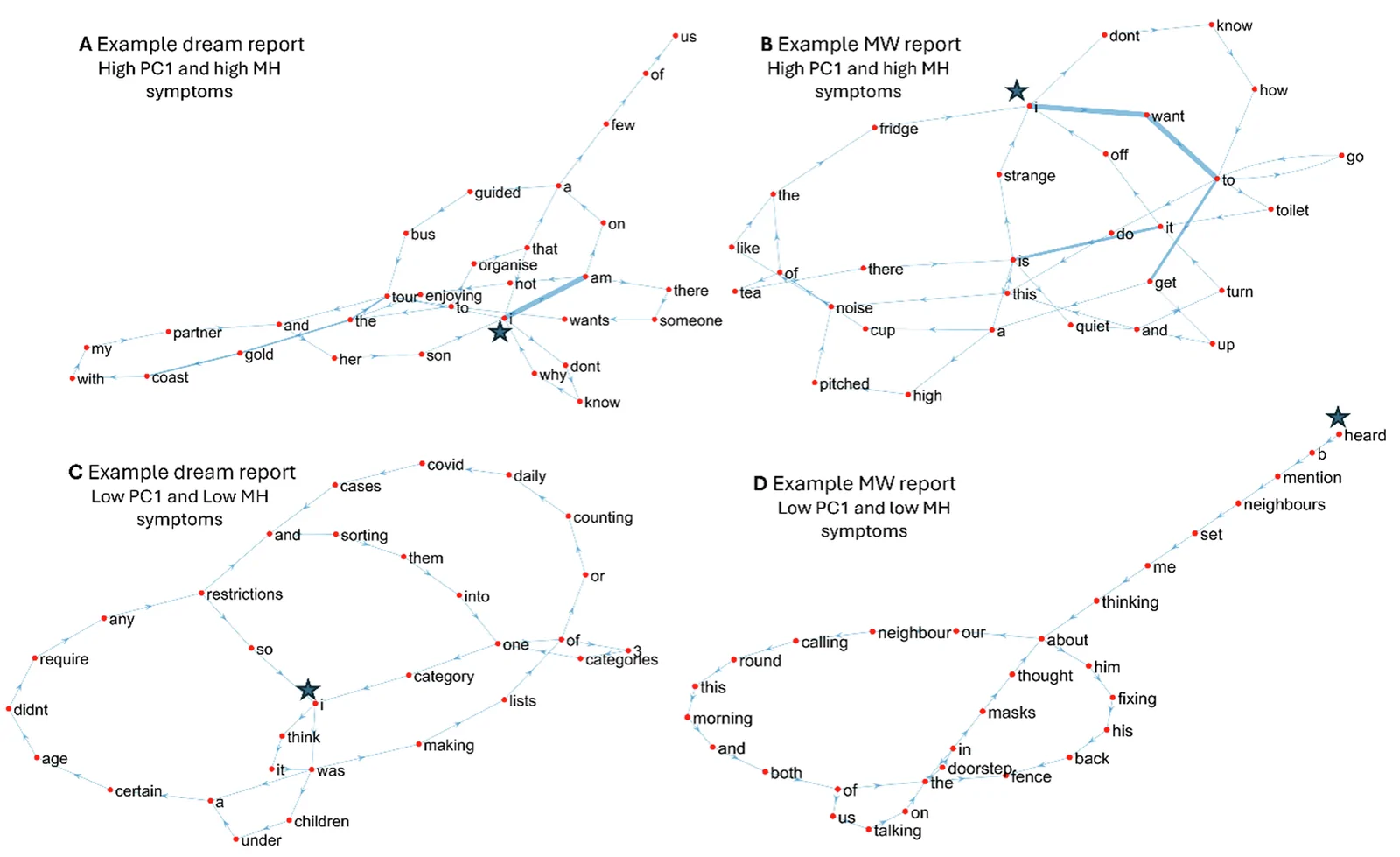
Examples of dream and mind wandering graphs with high and low PC1 and self-reported symptoms. The top left quadrant **A** shows an example of a dream report with high PC1 and high self-reported symptoms (PC1 = −3.01, PHQ-9 = 17, GAD-7 = 11). From the same participant, the top right quadrant **B** shows an example of a mind wandering report with high PC1 and high self-reported symptoms (PC1 = −2.47, PHQ-9 = 17, GAD-7 = 11). From a different participant, **C** the bottom left quadrant shows an example of a dream report with low PC1 and low self-reported symptoms (PC1 = 2.58, PHQ-9 = 4, GAD-7 = 2), whilst the bottom right quadrant **D** shows an example of a mind wandering report with low PC1 and low self-reported symptoms (PC1 = 3.07, PHQ-9 = 4, GAD-7 = 2; same participant as (**C**). Please note this is for illustration purposes only. In this figure, all networks have 30 nodes plotted. Please see Supplementary Material 5 for the full text used in this figure, with all the relevant graph theory metrics that are specific to those dream and mind-wandering reports, as well as a pictorial representation of the graph. Thicker lines indicate repeated sequences of words. The star indicates the beginning of the report. MH symptoms = self-reported mental health symptoms. PC1 = PC1-centrality.

Lower PC1 (i.e., lower lexical diversity, higher density/centrality) was likely to be associated with a higher PHQ-9 score in dream reports (in ordinal logistic regression coef. = −0.47, [95% CI = −0.84, −0.10], *p* = 0.013, Odds Ratio = 0.62, df = 11; see Fig. 3D). The centrality of mind-wandering reports also showed a relationship with the GAD-7 score (in ordinal logistic regression coef. = −0.48, [95% CI = −0.94, −0.02], *p* = 0.038, Odds Ratio = 0.62, df = 11). This means that lower lexical diversity was associated with more severe depressive and anxiety symptoms scored by the participant (see Fig. 4 for examples).

There was a negative relationship between age and PHQ-9 (ordinal logistic regression, coef = −0.031, [95% CI = −0.05, −0.009], Odds Ratio = 0.96, *p* = 0.005, df = 11), as well as with GAD-7 (iordinal logistic regression, coef = −0.031, [95% CI = −0.06, −0.005], Odds Ratio = 0.97, *p* = 0.016, df = 11). This result might be related to greater psychological burden of pandemic-induced isolation to younger individuals or education level.

Contrary to our predictions (https://osf.io/arx2h), we did not find evidence of verbosity, path length and betweenness centrality predicting the relevant outcome variables. Finally, in accordance with the pre-registration, we investigated whether the similarity between reports of the same individual would predict self-reported symptoms. We did not find any statistical evidence for this.

## Discussion

In this study, we sought to investigate the thought organization pattern while reporting inner experiences encoded during waking or sleep. Using word-recurrence graphs^3^, we characterised network structure differences between dream and mind wandering written reports, explored individual differences in these, and examined whether such features predict mental health symptoms.

We discovered six network features of dream and mind-wandering reports that have unique explanatory variance. These are centrality/connectedness, diameter, maximum betweenness centrality, assortativity, word count, and clustering coefficient. Using these metrics, we found that this method can distinguish between individual differences, that both types of report present different and complex patterns of mental organization in which dream reports tend to have a more “small-word” structure and higher intra-individual variation, probably related with diverse mechanisms to express memories encoded during waking or sleep. To conclude, we showed that dream and mind-wandering reports could be used to predict clinically relevant variables.

### Differences between dream and mind-wandering reports

There seem to be two diverse patterns of mental organization expressed in the mind wandering versus dream narratives, reflecting whether the memory is encoded during waking or sleep.

Dreams have been described as an intensified form of mind wandering^17–19^. On one hand, dreaming and mind wandering are considered continuous^11–14^. On the other hand, there seem to be consistent differences between these experiences^19,25,32,61,79^. We observed differences in the structure of the written reports of these experiences^25,46,80^.

Although we found differences, we do not outright reject the proposal that dreams and mind wandering are continuous^11^, given that distributions were overlapping (Fig. 2), the method and design of this study are not appropriate to make such claims, and only some variables differed between the conditions.

Specifically, dream reports were characterised by a more densely connected architecture and higher word repetition (i.e., with smaller lexical diversity, node centrality, in graph theory terminology). This effect was captured by the first principal component and is an important feature of the data, explaining high amounts of variance (in principal component analysis of highly correlated variables) and displaying the strongest effects (Figs. 1C and 3D). On the other hand, mind-wandering reports engaged higher lexical diversity and developed into longer structures.

We found that dream reports have a more complex structure, exhibiting the computational elements of a small-world architecture^81–83^. This network structure is characterised by clustered, interconnected neighbours (higher clustering coefficient) and a simultaneous ease in long-distance communication (lower path length). Although the effect sizes were small, this suggests that dream reports had more local clusters (neighbouring words also tended to be neighbours amongst themselves), but were, overall, also more interconnected (there were words that created shortcuts, that facilitated efficient transitions across the network).

By analogy, there may be difficulties in accurately recalling experiences from one state of consciousness (dreaming) when in another state of consciousness (wakefulness)^20,21^. The cognitive divergences between such states may therefore somewhat explain the convoluted and complex structures of these types of text. This pattern may reflect difficulties in organizing the narrative of a memory encoded during sleep, potentially due to reduced engagement of the default mode network (DMN) compared to wakefulness. In a recent study that presented affective images prior to sleep to investigate how emotional and mnemonic information is encoded during sleep onset, ~50% of the DMN structures were identified as sources of electrophysiological activity associated with the content and emotional residues of memories in dream reports collected during a nap^84^.

The multifaceted and somewhat unpredictable nature of experiences that may be had in dreams may also somewhat explain their higher structural complexity^16,25^. In fact, variably, dreams can be rather bizarre^18,85^, and may be considered comparable to psychosis in terms of a lack of reality testing and bizarreness^25^, and analogous alterations in prefrontal cortex functions^17,22^. Furthermore, analogies have been drawn between dreaming and psychosis, as both may relate to a breakdown of higher-order predictions^20,86,87^. In fact, texts produced by schizophrenic individuals^4,88^ and individuals under the influence of LSD^89^ show similar characteristics to dream reports (i.e., a small world structure, lower diameter and clustering coefficient). In schizophrenia, these elements may be partially explained by the cognitive difficulties and negative symptoms associated with such disorders^3,4^.

In the present study, we cannot dissociate to what extent these results relate to differences in working memory^40^ and long-term recall, rather than to the actual structure of the experiences. Nonetheless, the complex structure of dream reports, may intuitively, empirically, and theoretically^17,22,24,90^ be somewhat explained by the rather peculiar nature of such experiences.

Whilst dream reports were more recursive and complex, mind-wandering reports had a more linear, extended structure with essential transition points in the report structure (higher max betweenness centrality). These are connecting words that create “shortcuts” that connect the network in the more linear and extended structure that characterised mind-wandering reports. Importantly, mind-wandering reports also had less repetition of words (i.e., centrality), which may be of a syntactical or semantic nature^3^. The encoding and recall during wakeful consciousness may facilitate the mental organization of inner experiences such as mind wandering, enabling participants to construct narratives with greater lexical diversity and a more linear sequence of events. This typically involves the repetition of linking words that connect the beginning and end of narrative topics. In contrast, dream reports often exhibit repetitions within narrower thematic segments, possibly reflecting an effort to recall fragmented details as well as in structuring the experience into a coherent and logical account.

Participants were also more verbose in reporting mind wandering experiences (a feature which is independent from other graph theory metrics). This may indicate that mind wandering may just engender more content (e.g., words, ideas) than dreams. In fact, texts describing dreams imply longer-term recall (from sleep to wakefulness), whilst mind-wandering reports were characterised by a shorter-term “recording” after the mind wandering experience. Thus, this verbosity effect may be due to recency effects on recall (i.e., recall is easier with more recent events^73^). Furthermore, differences in working memory load during delayed dream free recall may have reduced verbal fluency in comparison to immediate mind wandering recall (although one may wander about the past). Similar questions arise with respect to differences in dream report length from different sleep stages.

Neuroimaging studies describe differences between dreams and mind wandering^17^ in regions that are fundamental to consciousness^91^. In fact, dream reports, compared to mind-wandering reports, are characterised by recall of information between starkly different states of consciousness^20^. This may make dream recall more susceptible to interferences (e.g., recalling irrelevant notions, confabulation^92^). To elucidate the implications of this intriguing characteristic, future studies may look at recall of present and past and future oriented text, and recall between different states of consciousness (e.g., inebriation and sobriety) and how this may affect the structure of reports^89^. Although we intended to keep the instructions and reporting style as similar as possible in these two conditions, participants were instructed to mind-wander for 10 min. Alterations in these instructions may have resulted in different amounts of verbosity, and it is unclear how one could consistently control verbosity in such naturalistic data. Either way, via the sliding window approach, the principal component analyses, and the inclusion of verbosity as a covariate in statistical models, we ensured that verbosity did not influence other graph theory metrics.

### Individual differences in dream and mind-wandering reports

Importantly, we found that within-individual similarity between dream and mind-wandering reports was higher than between-individual similarity. This suggests that this method is sensitive to stable characteristics that distinguish individuals in writing dream and mind-wandering reports. These findings may imply that writing style and the way people express their experiences is similar within individuals. In fact, we found consistency between an individual’s mind wandering and dream reports. This may be driven by underlying personality traits, particular affective styles and forms of emotion regulation^31,37,38,61^. However, an aspect to consider is the extent to which part of the individual-specific variation captured by this approach is driven by writing style, vocabulary, education, ethnicity, social economic status, engagement, and other such variables^3^. Although, we do include some of these relevant variables in statistical models, future research may try to elucidate how other affective, trait and demographic factors may differentially relate to the structure of naturalistic texts. In fact, in a recent review, the relationships between word recurrence structure and cognitive development, social cognition, and verbal memory in a typical population are elucidated^3^.

Furthermore, in harmony with the first hypothesis, dream reports had a greater amount of intra-individual variation. Higher variation is often interpreted as indicating higher complexity^93,94^ and information^95^. This may be explained by the various features discussed above, such as differences in recall length, the bizarreness, lack of reality testing of these experiences, variations in cognitive load, and difficulties in the free recall of information across different states of consciousness. The analyses exploring differences between dream and mind-wandering reports, and the individual differences therein, converge to indicate a notable degree of complexity in dream reports.

### Dream and mind-wandering reports predict mental health symptoms

Capitalising on this unique and extensive dataset, we found that graph theory analysis of written reports of dreams and mind wandering may relate to mental health symptoms, in accordance with previous literature using this method^3–5^. These results pave the way for the potential use of such techniques in clinically relevant screenings^68^, in additional analyses of qualitative data, or with substantial amounts of freely available online data for a variety of psychologically relevant questions.

We found that patterns of word repetition in dream and mind-wandering reports were related to self-reported symptoms of depression and anxiety, respectively. Lower density/centrality and, thus, a higher diversity of words in dream and mind-wandering reports was correlated with reduced negative symptomatology. Similarly, previous research has shown higher recurrence of words in dream recall in individuals with obsessive compulsive disorder^96^, which would translate to higher node centrality (lower lexical diversity) in a graph theory context. Furthermore, in a 2-year follow-up sample of individuals expressing attenuated symptoms, graph theory features were predictive of a conversion to anxiety and depression^97^. Both trait and state anxiety have been related to dream recall frequency^96^, which likely correlates with higher word repetition (i.e., node centrality).

Importantly, higher word repetition may be driven by symptoms that characterise depression. Lower cognitive flexibility, focus on the self, and higher rumination (which has been related to mind wandering and negative affect^49^) may be driving higher word recurrence that impacts lexical diversity.

Although cognitive symptoms are documented in anxiety and depression, we control for the verbosity of the reports in our analyses, and this variable did not show significant effects, in contrast to our predictions. Of interest is the difference between dream and mind-wandering reports in that they relate to depression and anxiety symptoms, respectively. Having reduced repeated measures by averaging metrics within participants, this discrepancy may be partly due to reduced statistical power. However, lexical diversity in mind wandering may be related to repetitive thoughts and worries, which may be particularly prominent during mind wandering and less noticeable in dream recall. In fact, tendentially, anxiety is particularly characterised by worry-driven repetitive thoughts about future situations^98^, which may have been preferentially expressed in mind-wandering reports. On the other hand, potential cognitive symptoms in depression may be more prominent during dream recall, which is cognitively complex (recall across states of consciousness), as noted above. Furthermore, higher word recurrence in dream reports may reflect self-centred processing (e.g., repetition of “I”, see in Fig. 4A for an example) or a ruminative focus on specific themes. Recurring dreams, in fact, are often characterised by negative content^99^. Post-traumatic clinical presentations may offer a stark example of these phenomena. In future research, parsing out the influence that different symptoms of depression may have on naturalistic reports may provide a thought-provoking elaboration on our findings. Beyond cognitive symptoms, another explanation is that there is higher negative (or apathetic) emotional content in the highly central words. However, in the present study, we plotted in word clouds the most repeated words according to level of depression (Supplementary Material 6) but did not find meaningful differences in themes.

Efforts in natural language processing have found that written semantic content can be used to predict depressive symptoms^66,67,70,71,100–102^. Our results provide an addition to previous such methodologies, which have been applied to compare dreams and mind wandering data^46^ in that we analyse the minimally pre-processed syntactic structure of these reports. In accordance to previous results focusing on phenomenological dimensions of such experiences, we found differences in terms of the structure of reports^46^.

Nonetheless, the integration of semantic information in the construction of text-based graphs may yield even more sensitivity and predictive validity for a wide variety of purposes across languages^3,103^. This might provide an interesting future direction with further additional benefit to previous studies^103^ and the present. For example, the use of questionnaires^80^ may complement the present large scale naturalistic approach to further understand how different aspects of the complex multidimensional nature of mind wandering relate to report structure and semantics^103^. Whereas phenomenological comparisons rely on the content of the experiences, semantic approaches capture their meaning, graph analyses capture a third dimension: the organizational architecture of how experiences are narrated. Graph analysis reveals structural properties underlying that difference (e.g., “small-world” structure) which semantic distance measures do not capture. A full characterization of waking and dream cognition requires analysis at all these three levels. Other experiencing sampling methodologies might be used in connection with the present method which allowed for direct analysis of the data, rather than further measures using post hoc evaluations of experiences of participants^103^.

Furthermore, anxiety and depression tend to be correlated, and thus, future studies may attempt to further dissociate unique variation. Interestingly, different types of reports from the same individual may yield incremental predictive power for mental health symptoms.

### Limitations

The writing of a verbal report of an experience (such as dreaming and mind wandering) poses the problem that it requires recall and then a verbal description of a subjective experience. As described above, it becomes difficult to disentangle the effects driven by verbal reporting style, recall type, time elapsed since the experience, various elaborations of the experience, etc. Nonetheless, the study was designed to render the two experiences in question as comparable as possible^38^. The qualitative differences in recall may have had an effect on the data (e.g., from different states of consciousness^104^). These may be considered a relevant difference between dream and mind-wandering reports, and perhaps telling on the debates as to extent to which mind wandering and dreams are continuous. Of note is that the time of day may have had an effect on the reports. Beyond recency effects on recall, some participants may have been more alert whilst completing the mind wandering tasks, thus perhaps explaining the higher verbosity in such naturalistic data. Furthermore, individual differences in circadian preferences may have had additional effects. Rather than the present natural language, big data approach, future studies may consider a more laboratory-based approach, where mind wandering experience is sampled via a think-aloud paradigm, and a wake-up paradigm is utilised for dreams. However, such an approach would still suffer from a difference in the reporting of an experience (engendering a second “recall” and “verbalization” experience) versus the experience itself. Different timings of reports might be manipulated in future studies to further explore how various features of recall might influence reports.

Of note is that this study instructed participants to engage in mind wandering during a set period, providing somewhat of a contrast to some definitions that highlight task-unrelated aspects of mind wandering^29^.

The operationalization used here is broader than that used by others. Nonetheless, as Seli et al.^27^, note “Specific and exclusive definitions of mind-wandering do not—and perhaps cannot—adequately capture the rich variety of this experience”, and “mind-wandering” is a useful umbrella term just as “cognition,” and “creativity” are. However, this might engender the limitation that “essentially any thought” might be considered mind wandering. However, the instruction to let mind wander, allows an intuitive naturalistic operationalisation that participants could easily interpret at face value. Such an operationalisation excluded clear performance-based goal-directed tasks (e.g., such as those that might be used in an executive function neuropsychological assessment^105^). The present operationalisation^27^ is analogous to those used in resting state functional magnetic imaging studies where participants are instructed to let their minds wander^106^. Furthermore, such a definition was deemed to allow a greater comparability between the two conditions of interest in the present study (i.e., dream and mind-wandering reports)^11^, whilst preserving the naturalistic aspects of the data and allowing extensive data collection.

The various degrees and multidimensionality of intentionality and executive control in mind wandering are variably discussed in the literature^26,27,29^ and above, there may plausibly be considerable differences between an unintentional and intentionally initiated form of mind wandering (e.g., differences in attentional and executive functions and affective content^26,28^). Future studies may investigate differences between different operationalisations of mind wandering^30^.

Although it is possible (although not in such a large and naturalistic study) in principle to wake participants up and obtain an immediate recall of dreams as well as obtain live mind wandering reports, this would hardly make for naturalistic conditions, and beyond this, immediate reporting is likely to change the nature of the experience itself (a well known, difficult and arguably unresolved issue in consciousness science^107–109^).

As per the preregistration (https://osf.io/su7mn/files/arx2h), we focused on exploring whether (i) dreams and mw have different structures as measured by graph theory, (ii) that they have individual specific information and that (iii) they relate to clinically relevant information. As such, the discussion is largely focused on interpreting results within this context. Relevant theoretical contributions relate to the notion that MW and dreams are continuous, although this is appropriately interpreted in accordance with the limitations of the method. The operationalisation that mind wandering “undirected thoughts during wakefulness”^17^ might be, arguably, considered sufficiently similar to the present operationalisation to provide a contribution to the theoretical proposal that dreams and mind wandering are continuous. However, if this were not to be considered sufficiently similar, the methodological contribution of this paper might be considered in further research.

Although we find that the present method is sensitive to individual differences, we could not exhaustively elucidate what might be driving such differences. For example, differences in aspects of executive function (e.g., inhibition vs verbal fluency), ethnicity, and socioeconomic status may have influenced the structure of dream and mind-wandering reports.

Whilst the large sample size is a strength, this may have allowed to find small effects that would not be found with smaller sample sizes (e.g., PC2, Clustering coefficient and word count in Fig. 2, individual differences analysis). Furthermore, although the participants were instructed to find an environment free from distractions, we did not employ post-hoc measures evaluating distractions. Such measures may be a consideration for future studies.

The use of sliding window may have highlighted different words in each window. We implemented a method with several precedents (e.g., refs. ^2,3,5,40,97,110^). An interested reader may consult Mota et al.^3^ for an overview of the methodology. In this approach, stop words (i.e., common, grammatically functional words such as “I”, “and”, “in”, “the”) are not removed. These words may contain important information (e.g., the use of “I” as a subject rather than “me” as an object of a verb, the repetition of “and” in speech/thought organisation or narrative planning). This method is sensitive to the structure of verbal data rather than its content. Nonetheless, future studies may focus on exploring the importance of stop words in this method, across different conditions. This may be united with an analysis of the grammatical category of important words in these speech graphs. Thus, future research may also look for salient words after having removed stop words and explore whether other variables may explain sentence word repetition (e.g., education, sentence length). Furthermore, this method may be united with other natural language processing methods to further characterise the features this approach is sensitive to.

## Conclusions

Here, we sought to characterise different reports of spontaneous thoughts and experiences, to further validate a graph theory approach to natural language processing and investigate the validity of this methodology. Our findings indicate that dream reports are more complex than mind-wandering reports. We show that the present method can distinguish between individual differences. We demonstrate that certain features of text can predict self-reported mental health symptoms. Thus, this method may have implications for characterising individual differences and symptoms in widely available and naturally occurring written language, as well as cost-effective mental health screenings. Graph analysis of verbal reports may offer a promising complement to content-based and phenomenological approaches.

## Supplementary information

Transparent Peer Review file

SUPPLEMENTAL MATERIAL

## Data Availability

Preprocessed data is available from https://github.com/Peter6789/graph-theory-analysis-of-text.

## Code availability

A MATLAB script to create networks from text following the procedure above is freely available at https://github.com/Peter6789/graph-theory-analysis-of-text/tree/main.
