# Supplementary material for: A graph theory approach to chart the architecture of dream and mind-wandering reports
Source: Commun Psychol. 2026 Oct 8;4:135. doi: 10.1038/s44271-026-00509-5 (PMC13650135; doi:10.1038/s44271-026-00509-5)
Supplement: Supplementary file 1 [file 44271_2026_509_MOESM1_ESM.pdf]

nature portfolio

Peer Review File

The architecture of spontaneous thoughts and experiences: a graph theory approach

Corresponding Author: Dr Peter Coppola

This file contains all editorial decision letters in order by version, followed by all author rebuttals in order by version.

Version 0:

Decision Letter:

** Please ensure you delete the link to your author homepage in this e-mail if you wish to forward it to your coauthors **

Dear Peter,

Thank you for your patience during the peer-review process. I am sincerely sorry for the unconscionable delay in returning to you with a decision.

Your manuscript titled "The architecture of spontaneous thoughts and experiences: a graph theory approach" has now been seen by 3 reviewers, whose comments are appended below. You will see that they find your work of some interest. However, they have raised quite substantial concerns that must be addressed. In light of these comments, we cannot accept the manuscript for publication, but would be interested in considering a revised version that fully addresses these serious concerns.

We hope you will find the Reviewers' comments useful as you decide how to proceed. Should additional work allow you to address these criticisms, we would be happy to look at a substantially revised manuscript. If you choose to take up this option, please highlight all changes in the manuscript text file, and provide a detailed point-by-point reply to the reviewers.

Editorially, we consider it particularly important that the methods are judged to be a sensitive measure of the conceptual framework, so that the results offer a strong test of the hypotheses. Please address all methodological and conceptual concerns in detail. As you do so, please ensure that you follow journal guidelines on the reporting of preregistered and post-hoc analyses (as detailed in the checklist). Please also ensure that the reporting complies with our requirements for statistics reporting and interpretation (also detailed in the checklist).

Please attend to each item in the checklist and ensure your manuscript is fully compliant. We are requesting that your manuscript aligns with these requirements as this facilitates the evaluation of your revised manuscript, reducing delays in re-review and potential future acceptance. If your revised manuscript is not aligned with these requests on major issues, such as those concerning statistics, it may be returned to you for further revisions without re-review. Additional information can be found in our style and formatting guide Communications Psychology formatting guide.

If the revision process takes significantly longer than five months, we will be happy to reconsider your paper at a later date, provided it still presents a significant contribution to the literature at that stage.

We would appreciate it if you could keep us informed about an estimated timescale for resubmission, to facilitate our planning.

We are committed to providing a fair and constructive peer-review process. Please do not hesitate to contact us if you wish to discuss the revision in more detail.

Please use the following link to submit your
- revised manuscript,
- point-by-point response to the referees' comments,
- cover letter (as a separate document),

- the Editorial Policy Checklist (see below),
- the Reporting Summary (see below), and
- the completed Editorial Request Table (attached):

Link Redacted

** This url links to your confidential home page and associated information about manuscripts you may have submitted or be reviewing for us. If you wish to forward this email to co-authors, please delete the link to your homepage first **

Thank you for the opportunity to review your work.

Best regards,

Marike

Marike Schiffer, PhD
Chief Editor
Communications Psychology

REVIEWER EXPERTISE:
Reviewer #1 task states, mind-wandering, cognitive neuroscience
Reviewer #2 task states, mind-wandering, cognitive neuroscience
Reviewer #3 graph theory, semantic analysis, CSS

REVIEWER REPORTS:

Reviewer #1 (Remarks to the Author):

Thank you for the opportunity to review this manuscript. I really enjoyed the topic and the methods used here. The results are potentially of interest to researchers as well. At the same time, I do not recommend publication of this paper due to some issues that I think are quite serious including some of the analyses (PCAs, no removal of stop words, lemmatization, etc.), conceptualizations of the key constructs, and methods. Some of these may be addressed, but some are difficult to overcome and I will of course defer to the Editor and other reviewers on this.

There is a strong assumption that thought can be accurately represented through language and that the paradigm used here is actually representative of the mind wandering a dreaming state. This issue goes back to William James, etc. who questioned whether language actually changes thought by simply trying to engage in this interpretation. I think the authors may be able to make a case that language is the best proxy we have, but I am still concerned that their particular methods are difficult to tie directly to the states in question. In this case, it is a memory of what that state is like expressed through language. With dreams, I understand you can't really track it in real time, but for thoughts you have much better access in real time as evidenced by others conducting similar work with think aloud etc. Some convincing that these memory of a thought stream is akin to the stream in real time is important to establish.

Some of the claims that follow from the results make me wonder if the method is driving these results. For example, "participants were more verbose in reporting mind-wandering experiences. This may indicate that mind-wandering may just engender more content (e.g., words, ideas) than dreams." This claim could entirely be explained by the memory access when folks are awake. The authors point out that verbosity does not affect the graph theoretical measures, but I believe that the memory access and descriptiveness (i.e. concreteness) could have played a role. One way to show that this is not the case is to look at the concreteness/vividness etc. in descriptions across dream and mind wandering states. The linearity of mind wandering, similarly, could simply be because of the recent access to the actual structure and how it unfolded temporally.

I am somewhat unclear about the inclusion of the idea that mind wandering can be intentional. This view does not align with the other one that is cited that suggest that mind wandering is spontaneous. I would ask the authors take a stronger stance on what they mean by mind wandering (in their view) because different conceptualizations are perhaps being conflated here. From my read, the intentional / unintentional distinction is purely related to studies of task-unrelated thought, whereas another camp of researchers argue that such intentional off task task is not mind wandering at all — it is merely directed thought that was intentionally not related to a task (see Christoff et al. and Seli et al. articles from TiCS, 2018). When making a comparison here, it is important to describe the phenomenon in ways that align with the data that was collected as well as help craft a strong and cohesive theoretical argument that is based on a clear definition of what mind wandering actually is. I do not think it is important for the authors to necessarily agree with either the Christoff or Seli approach, but I do think having clarity is important. In some ways the same thing could be said for dreams, where the authors suggest that dreams can take many forms; what form is studied here and why is that representative of "dreams"?

A lack of theoretical clarity is also related to how he authors have discussed and framed their individual differences discussion in the Intro. For example, it is suggested to be related to executive function, yet that intentionality might matter. If

going this route, this would need to be unpacked more in terms of how it relates to the current study. As well, I am a bit nervous by the perpetuation of the narrative that mind wandering is overall “negatively” related to anxiety and mood. In fact, recent studies suggest that it can even be the opposite when considering either content of the self-report or which “dimension” of mind wandering is being measured (for a review, see Kam et al., 2023, Psych Bulletin). This is again where I believe the methods could be influencing the results. Perhaps people were intentionally directing their thoughts during the "mind wandering" period. This is very different based on how they were engaging in the task.

The hypotheses, in my opinion (and especially related to H3), could be both more strongly motivated with clearer conceptualizations. I also think it would be beneficial to integrate the hypotheses into the actual narrative rather than saving them as a list at the end. This list made it difficult to map to the prior work.

Related to one of my points above, one of the major issues with this study is the way that mind wandering reports were recorded as retrospective memories. There are several key issues on this methodology, but one of them being that we have no reassurance that the memory for dreams after waking up and the memory for thoughts immediately are not actually driving the differences in the results— rather than the different states themselves. The claims being made in the paper are ultimately difficult to trust given the methodologies used.

I also understand that this makes it akin to the dream recalls, but I would like to see the authors make a stronger case for why this is a valid way to study mind wandering. I think this includes being able to compare to other actual think aloud or type aloud studies as well.

I would really like to see some descriptive statistics that lets the reader see how similar the reports were in terms of word count, sentence count, etc. across the states. The lack of descriptives really makes the paper difficult to evaluate. This is for the key variables but also perhaps a histogram of how many reports and length of report per person (with SDs).

Why were three components extracted? Explaining 91% of the variance with a the first PC seems extremely high. Did the authors look at and elbow plot, etc. or follow some convention to make these decisions? Please provide more information on this with clear justifications based on prior literature. The loadings of the PCA features are also important to discuss.

As well, how did the repeated measures aspect of the PCA come into play? The fact that people had different reports means that they were likely “weighting” the PCA. Please address this issue with more detail or updated analyses.

One issue is that the paper does not connect to prior work on using graph theory in language more generally. This work has been going for quite some time and seems like it would relate to the hypothesis — especially in relation to narratives etc.

I noticed in the graph that some words I would expect to be removed were actually in the graph. In NLP analyses, we often remove “stop words” in order to address non-content words biasing results. I noticed in the Discussion this was pointed out as a limitation, but I would argue it is not a minor limitation that should be address in future work. Stop word removal is a pretty basic part of language processing that is almost always the first step along with lemmatizing, etc. The way this was done essentially values meaningless words and also does not allow for words like run, running, ran to be considered the “same” which is also a problem in my view.

I understand the authors are saying that the anxiety scores are ordinal. However, they are very frequently used as continuous measures. I would like to see if the results replicate regardless of how the data is split, and I would also like to see the descriptives per category that was created based on the scoring.

I am still not entirely sure how the mental health models were constructed. There is no way to estimate a multi-level model with a random effect when a person has only one value for the predicted value; the authors do acknowledge this. The authors should likely take an “average” of the graph theory metrics and then see how the aggregate measure relates to the scores. I was not clear if this was the approach taken (but I think it was); how the models were exactly constructed was not clear though.

Reviewer #2 (Remarks to the Author):

This study compared and contrasted the reports of dreaming and mind wandering using graph theory applied to natural language. Participants were asked to report their dreams in the morning and report their mind wandering thoughts during the evening every day for up to two weeks. The authors found that dream reports have a more complex structure (i.e., small world architecture), whereas mind wandering reports have less word repetitions and more verbose structure compared to the other condition. These reports are more similar within subjects compared to between subjects. Finally, one of the measures, the word centrality component, in dream reports positively predict depressive symptoms.
This study addressed a longstanding question about similarities and differences between dreaming and mind wandering using sophisticated measures of graph theory. I think the results are novel and will appeal to both fields. The analyses of choice are generally valid and appropriate for addressing the research questions at hand and I appreciate the pre-registered analyses. I am impressed with the size of the dataset and that it was collected across two countries. Most of the results are convincing while others appear less robust (which I elaborate below). I raise some questions about the methods and statistical analyses, and the interpretations about some of the results, with the aim to help improve the clarity and comprehensiveness of the manuscript. Please don’t take the number of comments to reflect my enthusiasm for this paper, as

I believe the paper has good potential.

GENERAL

Succinctness of Introduction/Discussion
Although I find the summaries of dreaming and mind wandering interesting, I did ponder several times throughout the introduction, the relevance of some the background literature. To that end, I have a few suggestions.
• I think a more focused introduction would help prepare the readers for the research question when they reach the "Present Study" section. As an example, I suggest making the third paragraph (Lines 68-84) more succinct. Having read the Discussion, I see why the authors wish to introduce concepts of reality testing etc here, but without knowing the results, this does not seem directly related to the 'architecture' of dreaming. The same applies to the section on individual differences. This section primarily summarizes findings involving personality traits, even though the manuscript does not implement measures of personality traits. I understand authors use this as a measure of individual differences, but it gives the illusion that personality traits will be examined. I also note it's not clear how some of the findings tap into individual differences so stating the link more explicitly would be helpful.
• Similar to the Introduction, I think there may be some ways to make the Discussion more concise. For example, the more complex structure of dream reports was already discussed in the previous page, so it would make more sense to include the discussion of this finding in the paragraph above, versus interpreting the results twice across separate non-contiguous paragraphs.

Terminology
This paper appears to use mind wandering throughout the manuscript in a way that may be inconsistent with the general definition of mind wandering in the field. Specifically, the methods indicate that subjects were asked to think about whatever comes to mind without specific goals in a distraction free environment (reminiscent of a resting state instruction) and then to report their thoughts. This would fit better with the definition of spontaneous thought than mind wandering, in particular based on the papers cited in this manuscript (Smallwood & Schooler, 2015; Seli et al., 2016), all of which tend to define mind wandering as "task-unrelated thought". Given the subject's task is to let their thoughts wander, this would not be consistent with this common definition of mind wandering. I suggest the authors choose a different term, or explicitly define mind wandering in their own way that aligns with what the task captures early on in the introduction.

Depth of Result Interpretation in the Discussion
In the interpretation of results, I think the manuscript will benefit greatly from interpreting the results in a concrete way. For example, starting from Line 365, authors found a small world architecture in dream reports. Instead of (or in addition to) summarizing what small world architecture reflects in the context of graph theory, I think it would be much more meaningful to describe what that means in terms of dream experience. For example, what does it mean for a dream to show more clustered, interconnected neighbours? If possible, sharing an example or an actual excerpt from the study would be helpful. The same applies to interpretation of the mind wandering result (Line 376 onwards).

Consideration of Alternative Explanations
I'd like to suggest a few points of consideration to be discussed in a limitation section. First, could time of day of the report play a role in some of the differences observed between mind wandering and dreaming? For at least some individuals, they may be more alert while completing the mind wandering task and providing a report on their thoughts in the evening whereas they may be less alert when providing the dream report in the morning. I'm not sure the authors can address this, unless they have measure of hours and quality of sleep, as well as individuals' work hours (9-5pm vs graveyard shifts). But it would be important to discuss.
A related point concerns whether control measures or post-report question were implemented to ensure that 1) the environment in which participants allowed their minds to wander was in fact free of distraction and 2) the thoughts were not restricted by any specific goal (either through self-report or experimenter coding)? If so, please report and if not, this would be important to discuss as a limitation (as this impacts the 'spontaneity' of their thoughts). Consider an example in which the participant was in a coffee shop and their thoughts were selectively drawn to the conversation between two friends sitting next to the participant.

SPECIFIC

Line 214. The decision that additional features of text explained 'sufficient unique variance' based on correlations with other metrics <25% seems somewhat arbitrary. I'm not familiar with past literature that included both principal components and individual features in the same model. Can authors verify that this is appropriate mathematically and there are previous literature suggesting <25% is considered sufficiently unique to include in the same model with principal components derived from the same data? Related to this, can authors provide some past literature (on statistics and literature adopting this approach) that suggests $r < 0.5$ indicates independent variance (Line 231)? Some clarification on how these values were decided on would be helpful, as I did not see any information about this in the pre-registration.
Line 244. The reporting of the logistic regression seems to be inconsistent with the nature of these analyses. Specifically, logistic regression compares the odds of something happening. In this case, if I understand correctly, it is whether a report predicts mind wandering or dreaming. So the precise language I believe is that a certain network architecture feature has a higher probability (or betters odds) of characterizing a mind wandering than a dreaming report. It's a nuanced difference in reporting but I think this more precisely and accurately captures the analyses implemented.
Figure 2. The group level mean of PC2 for dreaming and mind wandering seem very similar. Yet the result is highly significant (despite a small coefficient). Although a large data set such as this one is valuable, it also does render very small effects significant by nature of the sample size. Can authors discuss the conceptual significance of their results for PC2,

taking into consideration this issue? It's not clear the effect would be replicated in a smaller sample. This is particularly concerning given PC2 accounted for <5% of variance in the data.
Line 277. Can authors report the number of data points of participants with both dream and mind wandering reports? I'm specifically wondering about whether the data is balanced in the analyses addressing H2.
Line 327. That multilevel mixed effects models did not yield robust results but repeated measures did is noteworthy. Typically, in accounting for within subject variance, multilevel mixed effects are more powerful. This contrasts with obtaining a large amount of data and narrowing it down to a single value per individual. Can authors address this in the Discussion, and speculate why this may be the case, and what this means in the context of predicting mental health status?
Line 374. Given the importance of the notion of node centrality (or word repetition), can authors elaborate on the last sentence of this paragraph? This seems crucial for understanding what this means for dreams by linking to other literature. Although the links are reported here, the actual relationship between node centrality and cognitive development/social cognition/etc are not described. This may help the authors more fully interpret and contextualize the findings.
Line 405. Have unpredictable or bizarre or unrealistic experiences been linked to more complex network structures? If so, this would be helpful to cite here. Otherwise, it would be helpful if authors elaborated on this speculation as to why more bizarre or unpredictable experiences have more complex structures.
Line 453. Given the results were not significant using multilevel mixed effects models and the coefficient is not large (or the p-value is not as highly significant as other effects), I would tone down this interpretation. In addition to the relatively unreliable effects, the study also does not address the mechanism by which higher word repetition in dream (but not mind wandering) reports was related to depression. Based on the speculated explanation, why may this be only observed in dream but not mind wandering reports?
Is the repeated measures analysis implemented post hoc and not in the preregistration? if so, this needs to be explicitly stated.
Line 488. I think it would be important to add a limitations section. For example, how well might these results be generalizable beyond the current sample. The authors reported age, gender and years of education. What about ethnicity?
Line 518. My understanding is that anything that is not included in the pre-registered analysis needs to be made clear. Can authors please ensure that? I recognize the analysis plan was written in a more of a high-level way, but some decisions (for example to include individual graph theory measures in addition to the principal components seems to be a post hoc decision). I think it would be appropriate to explicitly state which aspects were pre-registered (which is a valuable feature of this manuscript) and which aspects were exploratory/post-hoc.
Line 587. Based on my understanding of principal components as well as common practice, it is rare to see principal components that account for less than 5% of the data to be used for subsequent analysis. It's helpful to see the inclusion of PC2 accounts for significant unique variance based on model comparisons with the anova function. But it would be difficult to justify testing it in the first place. Can authors provide justification for examining this component in the first place? If not, this would seem to be a post-hoc decision – I did not find information in the pre-registration about how to select the number of components. If the authors feel strongly about keeping, please include a rationale and supporting literature in the Methods and report the variation explained in the Results, as this stark contrast between the variance accounted for in PC1 and PC2 is important for interpretation. This also needs to be addressed as a limitation. Line 625.

MINOR

Figure 2. Can authors plot out assortativity as well, despite the variable was non-significant?
Line 506. The authors state that a participant was excluded if they did not have at least three entries per type of report. Then they report that 1781 dream reports were recorded from 172 participants and 1496 mind wandering reports were recorded from 152 participants. So how is it possible to have a final sample of 176 participants (given that participants must have at least 3 entries of dreaming and mind wandering reports)?
Line 550. Can authors explain what word sequences are, for those not familiar with this approach?
Line 560. Can authors clarify what is meant by "several networks" were obtained for each report?
Line 604. When authors stated they reduced the data, are they referring to implementing the principal component analysis? If so this needs to be clarified as it is unclear written as is.
Line 617. Please clarify whether variables were scaled within subjects. I think that would be the most appropriate given the repeated measures nature of the data.

Reviewer #3 (Remarks to the Author):

This is an interesting piece of work, with a creative use of a graph-theoretic measure to look at how words are repeated. I think there's a lot of interesting material here.

However, I also think that there are major gaps in the analysis and presentation that would require at least major revisions and a change in presentation. There are some very basic problems (e.g., material in the abstract that directly contradicts material in the main paper, sentences that seem to contradict the ones just before) that mean that revisions would need to be significant and wholesale.

(1) The preregistration that the authors present is rather incoherent. In particular, they claim that "dream reports will be unstructured". However, they also claim that "dreams would have a more modular and hierarchical structure". This doesn't seem to make sense: if I write a paper (or a referee report) with distinct modules in a hierarchical arrangement, this seems to indicate a very structured presentation.

(2) It is very unclear if the authors have correctly translated the claim "modular and hierarchical" into graph theoretic

language. They find, for example, that dreams have higher "density/centrality". I'm not sure what that means — "network density" is usually something like "edges per node-pair", i.e., (# edges)/(N*(N-1)/2).) So a higher density is just the amount of word repetition — they should find that word repetition and density covary (almost) perfectly. The fact that dream reports have more word repetition does not seem to indicate "lack of structure" or (conversely) "modularity and hierarchy"

(3) There's a claim that PC1 is also "centrality"; it's unclear what this means, because centrality is usually a property of nodes, rather than edges. There's no definition of what they mean in the text. There's a great deal left out about the details of the analysis, and quite a bit of material is missing altogether (e.g., I'm referred to a MATLAB package for basic questions like what kinds of measures are being used.)

(4) It's also claimed that diameter is smaller in the dream graphs; it's not clear if this is max diameter, or average diameter (again, we don't have details). It's unclear if "unstructured" implies "small diameter" — indeed, you generally need quite a bit of structure to make diameter small; it's also unclear if "small diameter" implies "modular" (in general, if you have modules, the diameter will get larger).

(5) It would be good to show coefficients on the PCs, and how they decompose into the different basic measures.

(6) There seems to be an error in either the table or the text — it's claimed that dream reports have more clustering, but the coefficient is negative. (The negative coefficient for Diameter implies that mind wandering has larger diameter, so why doesn't a negative coefficient for Clustering imply mind wandering has larger clustering?)

(7) There are some strange claims that I don't quite understand — e.g., the claim that mindwandering has "essential nodal points". What does this mean? How is this determined by graph metrics? (I can think of ways it might be done, but I'm left to guess, and I'm left without any significance or effect size for this claim).

(8) I'm unclear why a larger diameter means a more "linear" narrative. Here are two narratives:

(A) I went to the store. At the store, I bought some oranges. I paid the cashier, who complimented me, and I left the store.

(B) I went to the store. A giant giraffe attacked me. Then some monkeys came out. My spaceship landed safely.

It seems clear that (A) is a very linear narrative, and (B) very random and non-linear. But (B) has a much larger diameter than (A), if I understand the method correctly — (A) repeats "I" and "store" a lot, but (B) has no repetitions. If by "linear" they mean something more specific, it needs to be made clear what this is.

(9) The results on mental health and texts seem so vague as to be basically null results. I couldn't understand, for example, why they couldn't do very simple logistic regressions, in the same way they did for the graph properties. This section needs to be either cut, or completely redone.

(10) The result that "people are more similar to themselves than to others" doesn't seem to make sense to me. How could the opposite be true?

(11) I don't see how the results claimed in the abstract are supported by the analysis. For example: thematic repetition in dreams? But how do we know it's thematic repetition, rather than just repeating function words like "I"? Above I've mentioned other issues: the claim that dream reports are "less structured" (this is H1, that they claim is consistent with their data) seems contrary to the claim in the abstract that dream reports have "more complex structure".

Summary: I don't want to seem too negative here. The basic story is that the authors came up with a cool method, and are clearly getting distinctions between the dreams and mind-wandering. There is great data, and the basic methods are strong. But there are big gaps: in both the framing of the analysis, the operationalization of the concepts, the construct validity of the claims, and the analysis of the the mental health material. I think that something approaching a complete re-write would be necessary here to fix things.

EDITORIAL POLICIES

We ask that you ensure your manuscript complies with our editorial policies and reporting requirements.

To that end, we require revised manuscripts to be accompanied by two completed items: a reporting summary that collects information on study design and procedure, and an editorial policy checklist that verifies compliance with all required editorial policies

- Nature Research Reporting Summary
- Editorial Policy Checklist

All points on the policy checklist must be addressed. Your revised manuscript can only be sent back to the referees if these checklists are completed and uploaded with the revision.

Notes: If you have submitted a Stage 1 Registered Report, Review, Primer, Comment, or Perspective you do not need to submit these forms. If you have already submitted these forms, you may disregard this request.

* TRANSPARENT PEER REVIEW: Communications Psychology uses a transparent peer review system. This means that we publish the editorial decision letters including Reviewers' comments to the authors and the author rebuttal letters online as a supplementary peer review file. However, on author request, confidential information and data can be removed from the published reviewer reports and rebuttal letters prior to publication. If your manuscript has been previously reviewed at another journal, those Reviewers' comments would not form part of the published peer review file.

** Visit Nature Research's author and referees' website at www.nature.com/authors for information about policies, services and author benefits**

Communications Psychology is committed to improving transparency in authorship. As part of our efforts in this direction, we are now requesting that all authors identified as 'corresponding author' create and link their Open Researcher and Contributor Identifier (ORCID) with their account on the Manuscript Tracking System prior to acceptance. ORCID helps the scientific community achieve unambiguous attribution of all scholarly contributions. You can create and link your ORCID from the home page of the Manuscript Tracking System by clicking on 'Modify my Springer Nature account' and following the instructions in the link below. Please also inform all co-authors that they can add their ORCIDs to their accounts and that they must do so prior to acceptance.
https://www.springernature.com/gp/researchers/orcid/orcid-for-nature-research

For more information please visit http://www.springernature.com/orcid

If you experience problems in linking your ORCID, please contact the Platform Support Helpdesk.

Version 1:

Decision Letter:

** Please ensure you delete the link to your author homepage in this e-mail if you wish to forward it to your coauthors **

Dear Peter,

Thank you for your patience during the peer-review process.

Your manuscript was vetted by two of the original three reviewers. The reviewers find your work improved, but also voice persistent concerns. In light of their advice, we invite you undertake further thorough revisions to the work. It is an editorial principle at the journal to avoid multiple rounds of re-review. We are making an exception in the present case, in the expectation that the revised version will address all of the reviewers' concerns comprehensively.

As you address the reviewers' comments, please bear in mind that the journal guidelines set a framework for the reporting of preregistered work (https://www.nature.com/commspsychol/editorial-policies/preregistration-policy). I highlight in particular the following:

-Deviations from the preregistration need to be transparently reported, with a justification for the deviation.
-In general, the preregistered analysis needs to be reported alongside the deviation.
-However, if the preregistered analysis is considered fundamentally flawed, this should be noted without reporting the results (as fundamentally flawed results should not be reported).

Please also review your statistics reporting (using the attached checklist and the information on our website as reference material for our reporting requirements and guidelines on interpretability).

We hope you will find the reviewers' comments useful as you decide how to proceed. Should additional work allow you to address these criticisms, we would be happy to look at a substantially revised manuscript. If you choose to take up this option, please highlight all changes in the manuscript text file, and provide a detailed point-by-point reply to the reviewers.

Please bear in mind that we will be reluctant to approach the reviewers again in the absence of substantial revisions.

I am attaching a checklist that details critical reporting requirements for the revised manuscript. Please attend to each item and ensure your manuscript is fully compliant. We are requesting that your manuscript aligns with these requirements as this facilitates the evaluation of your manuscript, reducing delays in re-review and potential future acceptance. If your revised

manuscript is not aligned with these requests on major issues, such as those concerning statistics, it may be returned to you for further revisions without re-review. Additional information can be found in our style and formatting guide Communications Psychology formatting guide.

If the revision process takes significantly longer than five months, we will be happy to reconsider your paper at a later date, provided it still presents a significant contribution to the literature at that stage.

We would appreciate it if you could keep us informed about an estimated timescale for resubmission, to facilitate our planning.

We are committed to providing a fair and constructive peer-review process. Please do not hesitate to contact us if you wish to discuss the revision in more detail.

Please use the following link to submit your
- revised manuscript,
- point-by-point response to the referees' comments,
- cover letter (as a separate document),
- the Editorial Policy Checklist (see below),
- the Reporting Summary (see below), and
- the completed Editorial Request Table (attached):

Link Redacted

** This url links to your confidential home page and associated information about manuscripts you may have submitted or be reviewing for us. If you wish to forward this email to co-authors, please delete the link to your homepage first **

Thank you for the opportunity to review your work.

Best regards,

Marike

Marike Schiffer, PhD
Chief Editor
Communications Psychology

REVIEWER REPORTS:

Reviewer #1 (Remarks to the Author):

In general, I found that the revisions did improve the paper. At the same time, I do not think some of the deeper issues were substantially attended to — mostly the comments by myself and Reviewer 3, which point out the submission tends to focus on their methods without linking them in very deep ways to the literature and existing theories. I also think some of the methods were applied in ways that make it difficult to understand the mechanistic contribution.

Looking at the changes made to the submission, the authors have added some additional explanations and clarifications - but that is not exactly what the reviewers were requesting in the major revisions (in my opinion). I think the comments were asking for substantial changes to the framing, hypotheses, and connections to the literature - both in spontaneous thought and graph theory. Some of the responses instead simply pointed to previous publications to justify choices — which in my view is not sufficient. In some cases, the responses themselves were more sufficient compared to what actually made it into the paper.

Some of the updated language is useful but raises some additional questions. For example, the authors have updated the text to say they attempted to fit multilevel models for the mental health analyses. I guess I would argue that acknowledging that attempt is likely (and the several significant effects) are potentially misleading given that it is simply not an accurate modeling strategy (which is seemingly acknowledged). Saying that there were any significant effects is not really a good idea in my opinion, unless there is a way to suggest this is in fact the correct way to analyze the data.

Related, I guess I am a bit uncomfortable with some of the responses, such as "across many different permutations of the analyses, PC1 showed convergent effects." In a hypothesis driven, null hypothesis testing framework, why are there multiple permutations? This seems to be directly at odds with other places saying the study was pre-registered. Some of this was updated, but I think the issues raised by the other reviewers (confusing preregistration hypotheses and changes made) makes me wonder how valuable the preregistration actually was.

I am still not entirely convinced by the argument that no stop words should be removed. The recurrent/repetitive nature that is

mentioned in the manuscript could simply be just that (which would not require graph theory to determine) and may be related to the issues raised in my previous review — which is that it is product of the method not the phenomenon. At the same time, I understand the authors' argument that these words may be important to include. What I would suggest is that they remove a stop word list that contains highly commonly removed words, such as articles, prepositions, and pronouns — if the results are completely different after this removal, I would think that is an important point to note and would be useful for interpretation. This is related to the change that says the results indicate word level repetitions - but it'd be nice to know which words or categories of words, e.g.?

Can the authors also justify further why age, country, and gender were included in the analyses? If these are removed, how much variance does PC1 explain?

I am also still confused how the Manny Whitney U test were conducted. The Man Whitney test allows for within subjects data when compared as a paired comparison but it does NOT allow for multiple observations per person in a single group. It seems like the authors are still taking multiple reports per person, rather than averaging to participant level. Further, there are uneven samples collected from people, so not including some sort of random effect is also problematic because it is effectively weighted — but the more important point is that it is likely not the appropriate test here. This may be an issue with clarity but I'm struggling to follow how this analyses was not done at the report level.

The addition of the r values is useful, but also highly concerning in terms of practical effects. I realize this is mentioned, but again it seems like the sample size of reports might be "revealing" significant p values but extremely small effects overall.

Some of the graph theory metrics that are showing differences are still not well explained. I think it would be useful to explain these theoretically more, as well as perhaps comparing them against more simply measures that theoretically do not capture the same as what the graph theoretic measures do - i.e. verbosity, proportion of word repetition, etc.

How is each sliding window represented in the data? (i.e. does that create more instances)

I'm still not sure what to make of the 91% variance. I understand the authors response logically, but I guess I'm trying to understand how to make sense of this theoretically still and what the contribution of this is then. For example, the authors say the first component captured density/centrality in their summary of the PC, but it actually had way more variables in it and the loadings are not clearly presented in the manuscript.

I suggest using three significant digits throughout the paper.

Finally, the authors have added a limitations section which is great. However, in my first review, I pointed out some serious concerns with the the methods and if they are really representative of the phenomenon of interest. The issue is that the methods are also a bit of the "lead" here, which may be a problem without really grappling deeply with how this relates to the theories.

Reviewer #2 (Remarks to the Author):

I appreciate the authors' efforts in addressing my comments, which have certainly helped clarify some aspects of the manuscript. However, some of my original questions remain and I raised a couple of new comments based on the newly added sections.

Depth of Result Interpretation in the Discussion
I understand the authors believe that they cannot provide interpretation beyond a literal interpretation of the structure, because they cannot be sure that interpretation applies to all reports. Though I'm not entirely sure why an example cannot be used to explain the findings, even if this example doesn't apply to all the reports. Although I appreciate the data is variable and complex, I found this rather dissatisfying, especially for a psychology journal. If we apply sophisticated analysis tools on a dataset but cannot make sense of the data in terms of the phenomenology (which I believe is core to this type of naturalistic data), then it raises the question of how useful is that tool. I think it's important for the authors to interpret the results in a concrete way. Please refer to my original review comment for more details.

Alternative Explanations
I understand that the authors have instructed participants to report contents that enter their mind when they are not engaged in or focused on any specific activity or task, which was clearly stated in the Methods section. My original question pertains to whether authors verified that. Since the entire study is grounded in this definition of mind wandering (which the authors now specified is based on dynamic thoughts (Christoff et al., 2016)), it would be important to verify that participants actually did not "engage in or focused on any specific activity or task". In behavioral tasks, we can use performance to determine whether participants were performing the task as instructed. In these types of tasks, it's more difficult as there are no clear 'behavioral measures', but that shouldn't prevent one from implementing post-hoc measures, such as questions to verify. A more concrete way to address this is to actually look at their reports to see if the content was focused on a specific thought. As participants are instructed to "allow your mind to wander freely", if the thoughts were mostly focused on a topic, then this would mean the participant did not follow the instructions and the state that was captured likely does not qualify as "mind wandering".

PCA and Individual Features
Regarding the inclusion of principal components and individual features in the same model, the authors' response did not actually address my concern. The authors pointed to the pre-registration where they anticipated multi collinearity would be an issue, which based on the manuscript, is the rationale for implementing PCA. However, it does not address the concern that there are essentially overlapping data in the same model. As the authors pointed out, it may not be an issue "unless the assumptions are violated (e.g., multicollinearity)". I can't easily find anywhere in the manuscript whether this assumption was tested. This would need to be tested and reported.

New Analyses
Since the authors are reporting the relationship between word repetition in dream reports and the PHQ9 to be 'particularly stable', I once again suggest the authors try to explain this result in the Discussion; this seems to have been missed in the response document. Specifically, I think it would be important to explain, or at least attempt at an explanation for, why higher word repetition in dream (but not mind wandering) was related to depression.

Limitation Section
Minor point: "The writing of a verbal report of an experience (such as mind-wandering and dreaming) poses the problem that it requires the recall and then a verbal description of a qualitative experience. Such an issue relates to any verbal report." I believe this last statement is not correct. Verbal report in the context of a think aloud would circumvent the issue of recall, which may not be accurate.

EDITORIAL POLICIES

We ask that you ensure your manuscript complies with our editorial policies and reporting requirements.

To that end, we require revised manuscripts to be accompanied by two completed items: a reporting summary that collects information on study design and procedure, and an editorial policy checklist that verifies compliance with all required editorial policies

- Nature Research Reporting Summary
- Editorial Policy Checklist

All points on the policy checklist must be addressed. Your revised manuscript can only be sent back to the referees if these checklists are completed and uploaded with the revision.

Notes: If you have submitted a Stage 1 Registered Report, Review, Primer, Comment, or Perspective you do not need to submit these forms. If you have already submitted these forms, you may disregard this request.

* TRANSPARENT PEER REVIEW: Communications Psychology uses a transparent peer review system. This means that we publish the editorial decision letters including Reviewers' comments to the authors and the author rebuttal letters online as a supplementary peer review file. However, on author request, confidential information and data can be removed from the published reviewer reports and rebuttal letters prior to publication. If your manuscript has been previously reviewed at another journal, those Reviewers' comments would not form part of the published peer review file.

** Visit Nature Research's author and referees' website at www.nature.com/authors for information about policies, services and author benefits**

Communications Psychology is committed to improving transparency in authorship. As part of our efforts in this direction, we are now requesting that all authors identified as 'corresponding author' create and link their Open Researcher and Contributor Identifier (ORCID) with their account on the Manuscript Tracking System prior to acceptance. ORCID helps the scientific community achieve unambiguous attribution of all scholarly contributions. You can create and link your ORCID from the home page of the Manuscript Tracking System by clicking on 'Modify my Springer Nature account' and following the instructions in the link below. Please also inform all co-authors that they can add their ORCIDs to their accounts and that they must do so prior to acceptance.
https://www.springernature.com/gp/researchers/orcid/orcid-for-nature-research

For more information please visit http://www.springernature.com/orcid

If you experience problems in linking your ORCID, please contact the Platform

Support Helpdesk.

Version 2:

Decision Letter:

** Please ensure you delete the link to your author homepage in this e-mail if you wish to forward it to your coauthors **

Dear Peter,

Thank you for your patience during the peer-review process. Your manuscript titled "The architecture of spontaneous thoughts and experiences: a graph theory approach" has now been seen by the 2 reviewers, and I include their comments at the end of this message.

The reviewers do confirm that the revisions improved some aspects of the work, however, they both highlight significant shortcomings in the revision of the presentation that must be addressed in a final round of revisions.

If you do not consider these revisions feasible, it would be in your interest to seek publication elsewhere, as we cannot consider the work for publication unless it fully resolves these issues.

Commendably, your work was preregistered, however, the reviewers highlight that the preregistered hypotheses do not directly emerge from the relevant literature. This means that despite the preregistration, your work presents a degree of exploration that must be spelled out in the Introduction.

At the same time, Reviewer #1 highlights that the work does not sufficiently credit similar research in the literature and includes some references they found particularly lacking. It is critical that the revision explains exactly how the work confirms, extends, or contradicts previous studies and approaches.

Finally, both reviewers highlight that the work in essence compares immediate recall of mind-wandering with delayed recall of dreams. You do reflect on that in the Discussion. Nonetheless, it is important that this distinction, and consequently the potential role of memory in differences between the reports, is clearly highlighted throughout (Abstract, Introduction, Discussion). We also strongly encourage you to present suitable control analyses if possible (see Reviewer #1).

As you revise the manuscript in response to these issues, please also implement all requests in the attached Mandatory Revision Requests document. All requirements listed in this document need to be fully met, or the work will be returned to you for further revisions without peer review. This workflow is in place to increase the likelihood that the paper will be accepted for publication. It reduces the number of rounds of revision (and review) and ensures that the reviewers vet a version of the article that is compliant with journal policies. If you have any questions regarding the required revisions, please contact the journal prior to resubmission to avoid a negative outcome.

We are committed to providing a fair and constructive peer-review process. Please don't hesitate to contact us if you wish to discuss the revision in more detail.

Please submit the following items:

- Revised manuscript
- Point-by-point response to the referees’ comments
- Mandatory Revision Requests Table (attached).
- Cover letter (as a separate document)

via this link: Link Redacted .

** This url links to your confidential home page and associated information about manuscripts you may have submitted or are reviewing for us. If you wish to forward this email to co-authors, please delete the link to your homepage first **

We hope to receive your revised paper within 8 weeks; please let us know if you aren’t able to submit it within this time so that we can discuss how best to proceed. If we don’t hear from you, and the revision process takes significantly longer, we may close your file. In this event, we will still be happy to reconsider your paper at a later date, provided it still presents a significant contribution to the literature at that stage.

We look forward to seeing the revised manuscript and thank you for the opportunity to review your work.

Best regards,

Marike

Marike Schiffer, PhD
Chief Editor
Communications Psychology

REVIEWER REPORTS:

Reviewer #1 (Remarks to the Author):

I appreciate the chance to re-review this paper. The authors have once again made substantial changes that have improve the manuscript overall. I personally still have some concerns about the manuscript being published in its current form - partly due to some of the issues not being addressed in a deep and thoughtful way that I have already pointed out a few times. That said, there is still a lot of positive about the paper and so I will defer to the Editor and other reviewer's on the publication readiness of this manuscript.

In both of my previous reviews, I pointed out that the authors have not done a theoretical deep dive to formulate their key research questions and contributions. This is still problematic to me in a few ways. First, the authors would have a much stronger paper if they made the theoretical and/or methodological contributions very clear. There is some good interpretation in the discussion section, but it focuses on explaining individual findings. In my last review, I asked the authors to more deeply connect their work to the previous literature and theory. They authors indeed changed the framing of their Intro, but in once again a relatively "light touch way."

There are several example of this, some are pretty more shallow ones and others are deeper. I asked for a deeper examination without necessarily pointing out issues in great detail, but here I will point a few specifics to hopefully provide more clarity. One that I (and other reviewers) have already mentioned is the treatment and construct definition of mind wandering and spontaneous thought, as well as whether the task actually elicits this phenomenon. The authors still do not define this clearly and do not handle it in a nuanced way. There is an assumption that all thought that arises in this paradigm is mind wandering, which is not at all what the theory they cite (Christoff et al.) would suggest to be consistent with mind wandering. The Dynamic Framework would suggest it *could* arise in this context but by no means will. It seems to me the authors are comparing waking cognition and dreaming — this is actually a fair and interesting comparison, but to extrapolate this to "mind wandering" or spontaneity is a confusing leap regardless of what theory is implicated (even Seli et al's family resemblances theory would not agree, I don't think). I am not an expert in graph theory, but the deep contribution is still not clear in relation to this; what do we get by combining that literature with this question that we do not already know? The authors seems to still base their investigation on the idea that we need to be able to understand "the organisation of narratives when recalling introspective and personal memories from these different physiological states of consciousness, thus characterising the continuity and divergence of dreams and mind-wandering reports." Yet I am still struggling to see how the organization of these narratives will tell us more than we know from other NLP and phenomenological investigations — that they are different in key ways. Why does the organization tell us something specific and how does that relate to theory? I continue to believe this is an answer here, but I also think a stronger case should be made.

Another pretty clear issue is that the authors motivate parts of their individual differences section with the idea that "studies show that mind-wandering is often related to negative affect." This is, once again, relatively shallow treatment of the literature. The two papers cited on this claim do not even seem relate to the mind wandering literature directly, despite a rich literature that exists. The idea that mind wandering is related to negative affect is actually quite complex and nuanced (there is a recent review on this, as well as other work in this field). Some measures of mind wandering actually positively relate to affect. Again, the core theory cited here would suggest that mind wandering is actually different from rumination and anxiety, so this where the background and framing seems a bit off.

As for the shallow ones: One is that the paper opens talking about tracking psychological states on social media, which seems quite distant from the literature/constructs of interest here.

The authors have attempted to clarify the fact that one of the PCs accounted for over 90% of the variance. I guess this is a critical point where I'm not sure how to interpret the PC and how it relates to the core research questions and predictions. I am also not sure why: "the variables included were word count, max betweenness centrality, assortativity, PC-1 Centrality, PC2-Diameter, clustering coefficient, country, gender and age." I asked about why certain variables were included last time, but it's still a bit confusing to me. Why did the authors do the PC if the ultimate approach was to pick particular variables — why not just include those variables to begin with instead of their loadings? This is especially true since this part was not pre-registered.

I would have expected the authors to integrate their core hypothesis into their literature review. I agree with them that the review is useful, but it is still very descriptive in nature without being integrated with theory. For example, what does

hypothesis 2 telling us?

I am still concerned that the authors don’t account for the methods and how they contribute to the findings themselves. This is a retrospective account, which is essentially a recall. The mind wandering reports happened immediately after a 10 minute period, whereas the dreams are not necessarily immediate and may have more decay and less specificity as a result. The authors mention this in the verbosity section of the discussion, but I’m not entirely convinced that this is not actually a major factor. For example, if an only a subset of the mind wandering results were compared — specifically ones that are less verbose — were compared, do results totally change? Or perhaps after controlling for length and episodic specificity in the other models? I would love to see some action to address this rather than simply saying it is an issue that can't be addressed given that the claims from the paper rest on the methodological soundness.

I would think the authors might have also found some other relevant literature in their attempt to review related work, particularly comparisons of sleep and wake condition (or specifically mind wandering). For example, Kirberg et al. (2025), Mallet et al. (2025), and Gross et al. (2021), to name a few (some even including NLP, such as Mallet).

I appreciate some of the additional details on analyses. I’m still a bit confused about why sometimes a logistic regression was used when I assume that the PCs and PHq9 are not binary variables?

Again, I appreciate the authors work on this!

Reviewer #2 (Remarks to the Author):

I commend the authors for implementing sophisticated analyses on a complex dataset with the aim to address an interesting question. Unfortunately, I still see that they have not quite fully addressed some of the core issues raised by myself and the other reviewer, which mostly concerns the methods and theoretical/conceptual aspects. For example, I still struggle to interpret some of the results involving graph theory metrics in the context of the data. To address this comment, the authors provided examples of mind wandering and dream reports in Figure 4 and the actual verbalized data, which is helpful. However, it does not directly help with interpreting the results. Given that I have already made this comment twice, I'm not sure that an additional round of comment would lead to a different outcome.

I recognize the authors spent a huge amount of effort to addressing comments during both rounds and I appreciate those efforts. Perhaps this second submission is reflective of the most that can be offered in terms of providing theoretical grounds and offering meaningful interpretations."

* TRANSPARENT PEER REVIEW: Communications Psychology uses a transparent peer review system. This means that we publish the editorial decision letters including Reviewers' comments to the authors and the author rebuttal letters online as a supplementary peer review file. However, on author request, confidential information and data can be removed from the published reviewer reports and rebuttal letters prior to publication. If your manuscript has been previously reviewed at another journal, those Reviewers' comments would not form part of the published peer review file.

** Visit Nature Research's author and referees' website at www.nature.com/authors for information about policies, services and author benefits**

Communications Psychology is committed to improving transparency in authorship. As part of our efforts in this direction, we are now requesting that all authors identified as ‘corresponding author’ create and link their Open Researcher and Contributor Identifier (ORCID) with their account on the Manuscript Tracking System prior to acceptance. ORCID helps the scientific community achieve unambiguous attribution of all scholarly contributions. You can create and link your ORCID from the home page of the Manuscript Tracking System by clicking on ‘Modify my Springer Nature account’ and following the instructions in the link below. Please also inform all co-authors that they can add their ORCIDs to their accounts and that they must do so prior to acceptance.
https://www.springernature.com/gp/researchers/orcid/orcid-for-nature-research

For more information please visit http://www.springernature.com/orcid

If you experience problems in linking your ORCID, please contact the Platform Support Helpdesk.

Version 3:

Decision Letter:

** Please ensure you delete the link to your author homepage in this e-mail if you wish to forward it to your coauthors **

Dear Peter,

Your manuscript titled "The architecture of spontaneous thoughts and experiences: a graph theory approach" has now been seen by our reviewer, whose comments appear below.

Although the reviewer expresses persistent hesitations, they also clarify that these do not undermine the methodological soundness of your work, and emphasise their appreciation for the comprehensive edits you have undertaken.

Given the present feedback and on the basis of our own reading, although the work is not acceptable for publication in its present form, I am, in principle, to publish a suitably revised version in Communications Psychology in the next instance.

However, to be acceptable for publication in the journal, you will need to ensure that the remaining concerns voiced by the reviewer are - at least - responded to in the Discussion (as part of transparent peer-review, the disagreement will also be published in the associated record).

Moreover, from an editorial perspective, it is moreover necessary that you further clarify what ethical framework was in place to cover the work.

We therefore invite you to revise your paper one last time to address the remaining concerns of our reviewers and a list of editorial requests. At the same time we ask that you edit your manuscript to comply with our format requirements and to maximise the accessibility and therefore the impact of your work.

EDITORIAL REQUESTS:

Please review our specific editorial comments and requests regarding your manuscript in the attached "Editorial Requests Table". Please outline your response to each request in the right hand column. Please upload the completed table with your manuscript files as a Related Manuscript file.

If you have any questions or concerns about any of our requests, please do not hesitate to contact me.

SUBMISSION INFORMATION:

In order to accept your paper, we require the files listed here https://www.nature.com/documents/commsj-file-checklist.pdf .

OPEN ACCESS:

Communications Psychology is a fully open access journal. Articles are made freely accessible on publication. For further information about article processing charges, open access funding, and advice and support from Nature Research, please visit https://www.nature.com/commspsychol/open-access

At acceptance, you will be provided with instructions for completing the open access licence agreement on behalf of all authors. This grants us the necessary permissions to publish your paper. Additionally, you will be asked to declare that all required third party permissions have been obtained, and to provide billing information in order to pay the article-processing charge (APC).

Please use the following link to submit the above items:
Link Redacted
** This url links to your confidential home page and associated information about manuscripts you may have submitted or be reviewing for us. If you wish to forward this email to co-authors, please delete the link to your homepage first **

We hope to hear from you within two weeks; please let us know if you need more time.

Best wishes,

Marike

Marike Schiffer, PhD
Chief Editor
Communications Psychology

REVIEWERS' COMMENTS:

Reviewer #1 (Remarks to the Author):

I appreciate the opportunity to review this paper again. I think some changes have been made that have once again improved the paper. Although I still have issues with the theoretical framing and clarity of the overall contribution, I am in agree with Reviewer's 2 take from the last round of revisions— namely, that similar comments have been given several times and met with some improvements but perhaps not ones that fully address the core concerns about integration of theory and deeper engagement with prior work. From my point of view, I am not sure that an additional round of comments will be useful or lead to a different outcome. I will reiterate my remaining concerns so that the authors and Editor may still have them. In general, I really do think the paper has been significantly improved from where it started and I defer to the Editor about the final decision and next steps.

I appreciate the authors attempt to engage in defining mind wandering. I recognize that my take on this particular issue may be trivial at this point in the review stage, but I would once again press the authors to consider what their definition assumes and whether it really offers a definition. They have now defined mind wandering as essentially any thought - it can be intentional / unintentional and stimulus dependent or not (and so on). So, it is basically just thinking then — you can be ruminating, letting your imagine fantastical concepts, or planning your day and they'd all be considered mind wandering. In the authors distraction free paradigm, is planning their days with a very detailed to do list MW, or what about if they are trying to solve a problem for work? The literature that is cited, instead, takes pretty specific views of mind wandering (i.e. much of it is explicitly stimulus independent or task unrelated, even though the methods here assume no task at all - and the two operationalizations have actually shown has dissociable results).

If we are to generalize thinking in a distraction free environment to the specific term mind wandering, we may continue to let the field evolve in a very confusing way. Mind wandering must have something different from 'thought' to be a unique construct, even if it is one with fuzzy boundaries. I understand the Seli paper is used to argue for this definition, but even that paper does suggest there are some exemplar features of mind wandering and I do not think it was intended to imply literally *anything* that arises in a self-generated way is MW.

My suggestion is not to try to take a broad strokes approach to defining mind wandering and instead simply reflect what is collected in this study - waking thought in a distraction free environment. Then, situate and be clear about where the literature support is coming from (what constructs/operationalizations were actually measured) and how that relates to what is studied here (was it task unrelated thought, unconstrained thought, etc.). This is actually inherent in what is described in the Introduction (which is really strong!) — waking cognition versus dreams. It is confusing that the intro seems to talk about waking thought, but then it switches to mind wandering at the very end and defines it basically as thought.

For example, even the link to affect cited here is explicit about task unrelated thought when in fact folks like Kam et al. (just one example) have recently specific how different forms of thought (and different conceptualizations of MW) have very different relationships with affect. There is a caveat that indicates it's not universal, but it is also not very descriptive. I do not mean this takes away from the actual results; it is genuinely meaningful to find differences in between waking cognition and dreams, but the theoretical implications and predictions about what is different between those stages is important. Is it the wake/sleep or is it something specific about the idea of mind wandering and dreaming, which actually implicates something different if the theories that exist are taken seriously. This is a re-stating of my prior comments - to deeply engage with the theories and prior work.

This also has implications for the way that the authors state their contribution regarding dreams and MW. If dreams are intensified version of MW, then what feature exactly is intensified. This hypothesis is derived from a different conceptualization of MW, one that does not treat is a family resemblances but rather one that does defined the properties of MW that make it distinct from other forms of thought. In this way, I am sorry to report that I am still not satisfied with the logical arguments made to support the theoretical conclusion. The title of the paper alone is referring to However, I am still fine and endorse the methodological one and want to re-state that the findings themselves could be useful.

I would also say this relates to the limitations of the design as well. I understand this limitation has been mentioned It doesn't alleviate my concern that this is still describing the nature of the design rather than the actual constructs, but I understand this is not something that the authors are willing to deal with head on and with data, i.e. something like showing that a (smaller) set of more delayed waking cognition reports don't significantly different from the ones that are more time-locked.

Finally, I was a little disappointed that the authors rebuttal to my suggested literature additions that compared waking/dreams was not handled very deeply. The point was not to say that those papers have done the same thing, but rather to suggest their findings could inform the previous literature cited or to be used as converging evidence. Adding them as citations just for the sake of citing them did not do much to improve the paper. Instead, I would expect them to be an anchor point for discussing the results that the graph theory approach is adding over other methods.

Ultimately, I want to be clear that I think the authors have done a huge amount of work and I am appreciative of their responsiveness to our reviews. That said, my concerns remain and perhaps 'agreeing to disagree' is the best approach at this point.

** Visit Nature Research's author and referees' website at www.nature.com/authors for information about policies, services and author benefits**

Open Access This Peer Review File is licensed under a Creative Commons Attribution 4.0 International License, which permits use, sharing, adaptation, distribution and reproduction in any medium or format, as long as you give appropriate credit to the original author(s) and the source, provide a link to the Creative Commons license, and indicate if changes were made.
In cases where reviewers are anonymous, credit should be given to 'Anonymous Referee' and the source.
The images or other third party material in this Peer Review File are included in the article's Creative Commons license, unless indicated otherwise in a credit line to the material. If material is not included in the article's Creative Commons license and your intended use is not permitted by statutory regulation or exceeds the permitted use, you will need to obtain permission directly from the copyright holder.
To view a copy of this license, visit https://creativecommons.org/licenses/by/4.0/

Poin-by-point responses to reviewers

The architecture of spontaneous thoughts and experiences: a graph theory approach.

REVIEWER EXPERTISE:
Reviewer #1 task states, mind-wandering, cognitive neuroscience
Reviewer #2 task states, mind-wandering, cognitive neuroscience
Reviewer #3 graph theory, semantic analysis, CSS

We would like to thank the reviewers for their helpful comments. We have implemented many changes to the manuscript and have undertaken control analyses. We feel that the manuscript has improved greatly following their comments. Please see below a point by point response to their comments.

To facilitate the process we have highlighted the reviewer comments in BOLD.

We have indented quotations from the paper and highlighted changes in red.

Reference style is APA in the present document, whilst Nature style in the manuscript.

Reviewer #1 (Remarks to the Author):

Thank you for the opportunity to review this manuscript. I really enjoyed the topic and the methods used here. The results are potentially of interest to researchers as well. At the same time, I do not recommend publication of this paper due to some issues that I think are quite serious including some of the analyses (PCAs, no removal of stop words, lemmatization, etc.), conceptualizations of the key constructs, and methods. Some of these may be addressed, but some are difficult to overcome and I will of course defer to the Editor and other reviewers on this.

We would like to thank the reviewer for their comments. We have tried to do our best to answer the reviewer’s queries.

There is a strong assumption that thought can be accurately represented through language and that the paradigm used here is actually representative of the mind wandering a dreaming state. This issue goes back to William James, etc. who questioned whether language actually changes thought by simply trying to engage in this interpretation. I think the authors may be able to make a case that language is the best proxy we have, but I am still concerned that their particular methods are difficult to tie directly to the states in question. In this case, it is a memory of what that state is like expressed through language. With dreams, I understand you can’t really track it in real time, but for thoughts you have much better access in real time as evidenced by others conducting similar work with think aloud etc. Some convincing that these

memory of a thought stream is akin to the stream in real time is important to establish.

It is indeed true that subjective experience can never be directly accessed by a third person, and that language can be considered a feeble tool in describing complex experiences (such as mind wandering and dreaming). In the words of Williams James who is aptly quoted by the reviewer “irreducible pluralism, is the law” (James, 1890).

Furthermore, even with experience sampling methods, experiences are not reported in real time but require a transition into another state (from experiencing to reflecting and reporting). This issue has been a point of contention in the science of consciousness (i.e., science of subjective experience) where there has been an effort to create “no report” paradigms to try to obviate such issues (Block, 2019). Nonetheless, as the reviewer notes, language remains the best available proxy for conveying information about one’s experiences. First-person verbal reports, whether collected with the think-out-loud methods or retrospective recall, are the closest approximation of another person’s internal states and experiences, and it is a widely accepted method in consciousness studies.

However, a key methodological consideration in this study was ensuring that reports of dreaming and mind wandering were as comparable as possible. To this end, we designed the study so that mind wandering reports were collected retrospectively after a 10-minute mind wandering period, mirroring the collection of reports of dream experiences. Although collecting mind wandering reports closer to the moment of experience would have been ideal, doing so would have introduced an inconsistency in the time delay between the experience and its report different for mind wandering and dreaming, compromising comparability between the two states. While more frequent experience sampling across the day and night could have provided additional data points, such design was not possible in such a large dataset which was collected during the COVID-19 pandemic.

In naturalistic data collection, some degree of experimental control must be inevitably sacrificed. However, we tried our best to control for as many extraneous variables as we could in both the design and analysis stages of the study.

To further clarify this point, we have added the following statement to the Present Study subsection of the Introduction:

> The data we use are written reports derived from delayed recall of the experiences in question (mind wandering and dreams). Thus, our analyses pertain to the reports of mind wandering and dreaming rather than the experiences themselves.

In fact, after various discussions amongst the co-authors, we are careful throughout the manuscript to talk about mind wandering and dream **reports** rather than mind wandering or dream experiences themselves. For example, the abstract has been modified in the following way.

> Thus, this approach is sensitive to individual differences, quantitatively differentiates reports of two different states of consciousness and seems promising for cost-effective analyses of large naturalistically occurring qualitative datasets.

In various places throughout the manuscript, we acknowledge that reports are various degrees removed from the reported experience in various ways:

> e.g., “Participants were more verbose in reporting mind-wandering experiences. This may indicate that mind-wandering may just engender more content (e.g., words, ideas) than dreams. In fact, texts describing dreams imply longer-term recall (from sleep to wakefulness), whilst mind-wandering reports were characterised by a shorter-term “recording” after the mind wandering experience. Thus, this verbosity effect may be due to recency effects on recall (i.e., recall is easier with more recent events (Vandenbos, 2016). Furthermore, differences in working memory load during delayed dream free recall may have reduced verbal fluency in comparison to immediate mind-wandering recall (although one may wander about the past).”

We further summarise these issues in the newly included Limitations section:

> The writing of a verbal report of an experience (such as mind wandering and dreaming) poses the problem that it requires the recall and then a verbal description of a subjective experience. This challenge applies to any verbal reports. As described above, it becomes difficult to disentangle the effects driven by verbal reporting style, recall type, time elapse since the experience, various elaborations of the experience etc., Nonetheless, the study was designed to render the two experiences in question as comparable as possible (Sikka et al., 2023).

Some of the claims that follow from the results make me wonder if the method is driving these results. For example, “participants were more verbose in reporting mind-wandering experiences. This may indicate that mind-wandering may just engender more content (e.g., words, ideas) than dreams.” This claim could entirely be explained by the memory access when folks are awake. The authors point out that verbosity does not affect the graph theoretical measures, but I believe that the memory access and descriptiveness (i.e. concreteness) could have played a role. One way to show that this is not the case is to look at the concreteness/vividness etc. in descriptions across dream and mind wandering states. The linearity of mind wandering, similarly, could simply be because of the recent access to the actual structure and how it unfolded temporally.

We thank the reviewer for the perceptive and appropriate comment. This is an important consideration in this study. This indeed may have been an important factor indicating that these two states are not as comparable as may have been suggested in the past literature.

Of note is that, we can only collect data from the reports of these experiences (rather than the experiences themselves) and that we explicitly acknowledge that our outcome measures relate to the these reports throughout the manuscript. Therefore, such differences (e.g., delay length in free recall) may be considered a legitimate part of the object of study (e.g., mind wandering reports vs dream reports) rather than a confound variable.There is a trade

off between the use of naturalistic data and the ability to control for extraneous variables. Nonetheless, above we offer potential future directions for studying such variables in a controlled manner.

We discuss such issues explicitly in various parts of the manuscript. We also consider how such recency effects in delayed recall may have implicated other cognitive domains that may have had an influence in the writing style.

Please see an example of this below (also cited just above), cited directly from the manuscript, including discussions of recall.

> Participants were more verbose in reporting mind-wandering experiences. This may indicate that mind-wandering may just engender more content (e.g., words, ideas) than dreams. In fact, texts describing dreams imply longer-term recall (from REM sleep to wakefulness), whilst mind-wandering reports were characterised by a shorter-term "recording" after the mind wandering experience. Thus, this verbosity effect may be due to recency effects on recall (i.e., recall is easier with more recent events (Vandenbos, 2016). Furthermore, differences in working memory load during delayed dream free recall may have reduced verbal fluency in comparison to immediate mind-wandering recall (although one may wander about the past). In fact, dream reports are characterised by recall of information between starkly different states of consciousness (Carhart-Harris et al., 2014). This may make dream recall more susceptible to interferences (e.g., recalling irrelevant notions, confabulation (Rosen, 2013)). To elucidate the implications of this intriguing characteristic, future studies may look at texts of recall of present and past, and future oriented text, and recall between different states of consciousness (e.g., inebriation and sobriety) and how this may affect the structure of reports (Sanz et al., 2021). Although we intended to keep the instructions and reporting style as similar as possible in these two conditions, participants were instructed to mind wander for 10 minutes. Alterations in these instructions may have resulted in different amounts of verbosity, and it is unclear how one could consistently control verbosity in such naturalistic data. Either way, via the sliding window approach, the principal component analyses, and the inclusion of verbosity as a covariate in statistical models, we ensured that verbosity did not influence other graph theory metrics.

We also mention these difficulties in the newly inserted limitations section

> The writing of a verbal report of an experience (such as mind wandering and dreaming) poses the problem that it requires the recall and then a verbal description of a subjective experience. This challenge applies to any verbal reports. As described above, it becomes difficult to disentangle the effects driven by verbal reporting style, recall type, time elapse since the experience, various elaborations of the experience etc., Nonetheless, the study was designed to render the two experiences in question as comparable as possible (Sikka et al., 2023). The qualitative differences in recall may have had an effect on the data (e.g., from different states of consciousness, (Laureys et al., 2008)). These may be considered a relevant difference between dream and mind wandering reports, and perhaps telling on the debates as to extent to which mind wandering and dreams are continuous (Christoff et al., 2016). Of note is that the

time of day may have had an effect on the reports. Beyond recency effects on recall, some participants may have been more alert whilst completing the mind wandering tasks, thus perhaps explaining the higher verbosity in such naturalistic data. Furthermore, individual differences in circadian preferences may have had further effects. Rather than the present natural language, big data approach, futures studies may consider a more laboratory based approach, where mind wandering experience is sampled via a think aloud paradigm and a wake-up paradigm is utlised for dreams. However such an approach would still suffer from a difference in the reporting of an experience (engendering a second “recall” and “verbalization” experience) versus the experience itself.

Of note is that the word counts are well controlled for in various ways (sliding window, adding as a covariate in statistical models). Beyond this, we confirmed a lack of correlation between the graph theory variables of interest and word count following the sliding window procedure. We know that word differences may have an effect on Graph Theory Analyses and we made sure this was not the case. The extent to which the different nature of the recall (that extends beyond delay length) for these different experiences influence the graph theory analyses is not know, and potentially multifarious. Nonetheless, as stated above, such differences are naturally part of differences in written dream reports and mind wandering reports.

I am somewhat unclear about the inclusion of the idea that mind wandering can be intentional. This view does not align with the other one that is cited that suggest that mind wandering is spontaneous. I would ask the authors take a stronger stance on what they mean by mind wandering (in their view) because different conceptualizations are perhaps being conflated here. From my read, the intentional / unintentional distinction is purely related to studies of task-unrelated thought, whereas another camp of researchers argue that such intentional off task task is not mind wandering at all — it is merely directed thought that was intentionally not related to a task (see Christoff et al. and Seli et al. articles from TiCS, 2018). When making a comparison here, it is important to describe the phenomenon in ways that align with the data that was collected as well as help craft a strong and cohesive theoretical argument that is based on a clear definition of what mind wandering actually is. I do not think it is important for the authors to necessarily agree with either the Christoff or Seli approach, but I do think having clarity is important. In some ways the same thing could be said for dreams, where the authors suggest that dreams can take many forms; what form is studied here and why is that representative of “dreams”?

Mind wandering is often defined as task-unrelated or stimulus-independent thought (Smallwood & Schooler, 2015; Murray & Krasich, 2022). However, as Seli et al. (2016, 2018), Christoff et al. (2016) and Irving & Glasser (2019) have proposed, mind wandering can also be intentional, with varying degrees of executive control involved.

In our study, we adopted an approach similar to resting-state functional magnetic resonance imaging studies, where participants are instructed to let their minds wander freely during a set period of time (Gonzalez-Castillo et al., 2021). This approach was designed to align mind-wandering reports with the retrospective reporting of dream experiences, ensuring methodological consistency. Our approach is closer to the definition in Christoff et al., 2016: “Mind-wandering can be defined as a special case of spontaneous thought that tends to be more-deliberately constrained than dreaming, but less-deliberately constrained than creative thinking and goal-directed thought".

Following the reviewer’s comments, in the present study subsection, we summarise the instructions given to the participants to avoid confusion as to its operationalisation.

> We employed a unique and large dataset featuring multiple repeated measures of dreaming and mind-wandering (1619 and 1434 reports, respectively, from a total of 176 individuals). For the mind-wandering reports, we instructed participants to let their mind wander freely and subsequently write down what went through their mind during this period.

We specify the use of the spontaneous thoughts framework earlier in the introduction.

> Researchers have even challenged the notion that mind-wandering is a unitary phenomenon(Seli et al., 2016, 2018), noting variations in attentional neural systems, intentionality(Boulakis et al., 2023; Murray & Krasich, 2022; Seli et al., 2016) and affective outcomes(Kam et al., 2024). In this study we operationalise mind wandering using the spontenous thoughts dynamic framework(Christoff et al., 2016).

We also make the above issues explicit in the newly inserted limitations section. Please see this below.

> Of note is that this study instructed participants to engage in mind-wandering during a set period, providing somewhat of a contrast to definitions that highlight task-unrelated aspects of mind-wandering(Murray & Krasich, 2022). The various degrees and multidimensionality of intentionality and executive control in mind-wandering are variably discussed in the literature(Murray & Krasich, 2022; Seli et al., 2016, 2018), and could not be adequately explored in the present study. However, for this study (Sikka et al., 2023), we employ a definition of mind-wandering that is encompassed by the spontaneous thoughts framework(Christoff et al., 2016), which focuses on the dynamics of how thoughts unfold overtime. This is analogous to operationalisations used in resting state functional magnetic imaging studies where participants are instructed to let their minds wander(Gonzalez-Castillo et al., 2021). Such a definition was deemed to allow a greater comparability between the two conditions of interest in the present study (i.e., dream and mind-wandering reports)(Christoff et al., 2016). Nonetheless, there may plausibly be considerable differences between an unintentional and intentionally initiated form of mind-wandering (e.g., differences in attentional and executive functions and affective content(Boulakis et al., 2023; Seli et al., 2016)).

Regrettably, the naturalistic nature of this data would not allow the high degree of control (including potentially neural sampling) needed to characterise the type of dreams. Whilst some

authors may use a narrow definition of sleep, in this paper we consider any subjective experience occurring during sleep as dreaming. Again, we ensure that in the text we refer to all results as pertaining to the reports of mind wandering and dreams rather than the experiences themselves.

A lack of theoretical clarity is also related to how he authors have discussed and framed their individual differences discussion in the Intro. For example, it is suggested to be related to executive function, yet that intentionality might matter. If going this route, this would need to be unpacked more in terms of how it relates to the current study.

As well, I am a bit nervous by the perpetuation of the narrative that mind wandering is overall "negatively" related to anxiety and mood. In fact, recent studies suggest that it can even be the opposite when considering either content of the self-report or which "dimension" of mind wandering is being measured (for a review, see Kam et al., 2023, Psych Bulletin). This is again where I believe the methods could be influencing the results. Perhaps people were intentionally directing their thoughts during the "mind wandering" period. This is very different based on how they were engaging in the task.

Although we review the literature that cite individual differences in relation to such phenomena, one of our main questions in the present study is to see whether the approach is sensitive to individual differences defined as characteristics that distinguish an individual from another. To ensure the definition is clear we defined this more clearly when we introduce mind wandering.

> An important question is to what extent individual differences (stable characteristics that distinguish individuals from one another) underlie the content and structure of dreams and mind-wandering.

In the discussion we discuss how the individual differences found may relate to various variables.

> In fact, we found consistency between an individual's mind-wandering and dream reports. This may be driven by underlying personality traits, particular affective styles and forms of emotional regulation (Sikka et al., 2018a, 2019b, 2021b; Sikka, Tuominen, Ezquerro Nassar, et al., 2023). However, an aspect to consider is the extent to which part of the individual specific variation captured by this approach is driven by writing style, vocabulary, education, social economic status, engagement, and other such variables (Mota et al., 2023). Although, we do include some of these relevant variables in statistical models, future research may try to elucidate how other affective, trait and demographic factors may differentially relate to the structure of naturalistic texts.

Importantly, in this study, we were interested whether this approach could pick up on individual differences. The underlying factors for such individual differences are likley multifarious, complex, potentially relationg to sociological, as well as psychological and biological factors.

We added the following to the limitations section:

> Although we find that the present method is sensitive to individual differences, effect sizes were small and we could not exhaustively elucidate what might be driving such differences. For example, differences in aspects of executive function (e.g., inhibition vs verbal fluency), ethnicity, and socioeconomic status may have influenced the structure of dream and mind-wandering reports.

We thank the reviewer for their thoughts on mind wandering and for directing us to the very interesting paper by Kam and colleagues (Kam et al., 2024). This fits with our view that mind wandering is not a simple univocal process. The data shows that a feature (i.e., word repetition) of these reports are correlated to self-reported symptoms of depression. That means also that less word repetition in mind wandering reports might correlate with less self-reported symptomatology, rather than the frequency or duration mind wandering experiences themselves. To highlight the reviewer's point, we have added the following point to the paper to highlight the bidirectionality of the linear statistic.

> We found that higher repetition of words in dream reports was consistently related to self-reported symptoms of depression. Conversely, higher diversity of words in dream and mind wandering reports are correlated with reduced negative symptomatology.

We note that Kam and colleagues' analyses suggests that intentionality in mind wandering is associated with negative affect, but less so compared to "unintentional" forms of mind wandering. The, the positivity or negativity of mind wandering will likley be related to the content of mind wandering. In fact, future oriented mind wandering is associated with positive affect (Franklin et al., 2013). Following the reviewer's comments, we have added the following phrase in our introduction.

> In fact, researchers have challenged the notion that mind-wandering is a unitary phenomenon (Seli et al., 2016, 2018) and suggest that differences in intentionality during mind-wandering relate to differences in attentional network function (Seli et al., 2016; Boulakis et al., 2023) as well as differences in affective outcome (Kam et al., 2024). In fact, neuroticism has been related to higher frequency and more negative mind-wandering (Figueiredo et al., 2020; Robison et al., 2017; Welhaf et al., 2020), although not univocally (see Kam et al., 2024).

The hypotheses, in my opinion (and especially related to H3), could be both more strongly motivated with clearer conceptualizations. I also think it would be beneficial to integrate the hypotheses into the actual narrative rather than saving them as a list at the end. This list made it difficult to map to the prior work.

We thank the reviewer for their comment. Clarity in the manuscript is of utmost importance. Hence, we have followed the reviewer's advice and weaved the hypotheses within the review of previous literature. However, we have also kept a final list at the end of the introduction to bring the hypotheses together, provide a summary and a brief description of the study, in the hope this increases clarity for the reader.

After section one "**Dreaming and mind-wandering as continuous and divergent**"

> In this study, we sought to characterise the continuity and divergence of dreams and mind wandering reports by comparing the network structure of written reports of these phenomena.

After the second section "**Individual differences related to dreaming and mind-wandering.**"

> In this study, we investigate the characteristics of mind wandering and dreams reports are consistent within individuals and dissociable between individuals.

After the third section "**Dream and mind-wandering reports reflect mental health**"

> Thus, all the above suggests that reports of dreams and mind-wandering may not only reflect, but possibly also predict, mental health. In this study, we thus investigated whether the structures of mind-wandering and dream reports may relate to self reported anxiety and depression.

Related to one of my points above, one of the major issues with this study is the way that mind wandering reports were recorded as retrospective memories. There are several key issues on this methodology, but one of them being that we have no reassurance that the memory for dreams after waking up and the memory for thoughts immediately are not actually driving the differences in the results— rather than the different states themselves. The claims being made in the paper are ultimately difficult to trust given the methodologies used.
I also understand that this makes it akin to the dream recalls, but I would like to see the authors make a stronger case for why this is a valid way to study mind wandering. I think this includes being able to compare to other actual think aloud or type aloud studies as well.

The reviewer is absolutely correct that this is a major confound factor. This issue was considered by our team during the design of the study, and the comparability between the two conditions was prioritised. The ability to compare dream and mind wandering reports requires them to be as comparable as possible. The present methodology tries to achieve exactly this, whilst at the same time trying to maintain the naturalistic element in the data. In fact, explained in Sikka et al., 2023, reporting log methodologies is an improvement on the use of retrospective questionnaires.

In the text, we acknowledge the issue of memory recall of past memories, and discuss how memory recall may differ between these different experiences. Below is an example, with possible future directions.

> In fact, texts describing dreams imply longer-term recall (from sleep to wakefulness), whilst mind-wandering reports were characterised by a shorter-term "recording" after the mind wandering experience. Thus, this verbosity effect may be due to recency

effects on recall (i.e., recall is easier with more recent events (Vandenbos, 2016). Furthermore, differences in working memory load during delayed dream free recall may have reduced verbal fluency in comparison to immediate mind-wandering recall (although one may wander about the past). In fact, dream reports are characterised by recall of information between starkly different states of consciousness (Carhart-Harris et al., 2014). This may make dream recall more susceptible to interferences (e.g., recalling irrelevant notions, confabulation (Rosen, 2013)). To elucidate the implications of this intriguing characteristic, future studies may look at texts of recall of present and past, and future oriented text, and recall between different states of consciousness (e.g., inebriation and sobriety) and how this may affect the structure of reports (Sanz et al., 2021). Although we intended to keep the instructions and reporting style as similar as possible in these two conditions, participants were instructed to mind wander for 10 minutes. Alterations in these instructions may have resulted in different amounts of verbosity, and it is unclear how one could consistently control verbosity in such naturalistic data. Either way, via the sliding window approach, the principal component analyses, and the inclusion of verbosity as a covariate in statistical models, we ensured that verbosity did not influence other graph theory metrics.

To ensure that any reader is presented with this issue directly, in this latest version, we write about written reports of dreams and mind wandering events rather than actual dreams and mind wandering events. By focusing on the fact that we look at comparing reports, we acknowledge that differences are between these, and that these may be driven by various factors (including recall, cognitive load, recency effects, as mentioned in the discussion).

Following the reviewer's comment, although the experimental design would require careful elaboration, in the limitations section we have repeated the issue of recall and have proposed a future direction in which we propose wake-up and think aloud methodologies.

The qualitative differences in recall may have had an effect on the data (e.g., from different states of consciousness, (Laureys et al., 2008)). These may be considered a relevant difference between dream and mind wandering reports, and perhaps telling on the debates as to extent to which mind wandering and dreams are continuous (Christoff et al., 2016). Of note is that the time of day may have had an effect on the reports. Beyond recency effects on recall, some participants may have been more alert whilst completing the mind wandering tasks, thus perhaps explaining the higher verbosity in such naturalistic data. Furthermore, individual differences in circadian preferences may have had further effects. Rather than the present natural language, big data approach, future studies may consider a more laboratory based approach, where mind wandering experience is sampled via a think aloud paradigm and wake-up paradigms are utlised for dreams. However such an approach would still suffer from a difference in the reporting of an experience (engendering a second "recall" and "verbalization" experience) versus the experience itself.

As noted, the problems of recall across the different states of consciousness, the differences between conditions, the issue of verbal reporting of experiences (being itself a different experience from the reported experience (Block, 2019) and other aforementioned variations

in type of dreams and mind wandering etc., would remain. Other potential differences between such phenomena are explicated in the discussion (e.g., working memory load)

Of note, is that this methodology is derived from (Mota et al., 2014) that analysed the speech graph structure of different psychiatric disorders and found significant differences. Thus, to reiterate, we work on speech reports. Of course, a wake up paradigm would not be feasible with such a large amount of data, during a pandemic, and would perhaps require ethical considerations of a large amount of interruption of sleep during stressful times.

I would really like to see some descriptive statistics that lets the reader see how similar the reports were in terms of word count, sentence count, etc. across the states. The lack of descriptives really makes the paper difficult to evaluate. This is for the key variables but also perhaps a histogram of how many reports and length of report per person (with SDs).

The plots in figure 2C show the distributions for the variables of interest. However, given the appropriate request, we have added a supplementary material to elucidate these data. We invite the reviewer to see the descriptives below.

Supplementary Material 6

Descriptive statistics.

Below are descriptive statistics of the relevant continuous variables that were inserted into the statistical models

Table S15 Descriptive statistics for variables of interest. Presented are mean and standard deviation for dream and mind wandering reports and both of them together.

Variable	Mean			Standard Deviation		
Types of report	Dreams	Mind wandering	Both Types of Reports	Dreams	Mind wandering	Both Types of Reports
Word count	197.52	201.51	199.39	163.30	140.02	152.79
PC1	-0.99	1.10	-0.004	2.80	2.85	3.01
PC2	0.02	-0.02	0.0009	0.60	0.64	0.62
Assortativity	-0.15	-0.13	-0.14	0.06	0.05	0.05

Maximum Betweeness centrality	0.55	0.56	0.55	0.042	0.039	0.037
Clustering	0.13	-0.15	0.0004	1.04	0.92	0.999
Age	48.87	49.59	49.21	15.16	15.26	15.21

Please note that all the variables were z scored before being inserted into the statistical models. For further transformation of the data to ensure normality, see supplementary material 2.

Figure S5 Shows the frequency of word counts of reports.

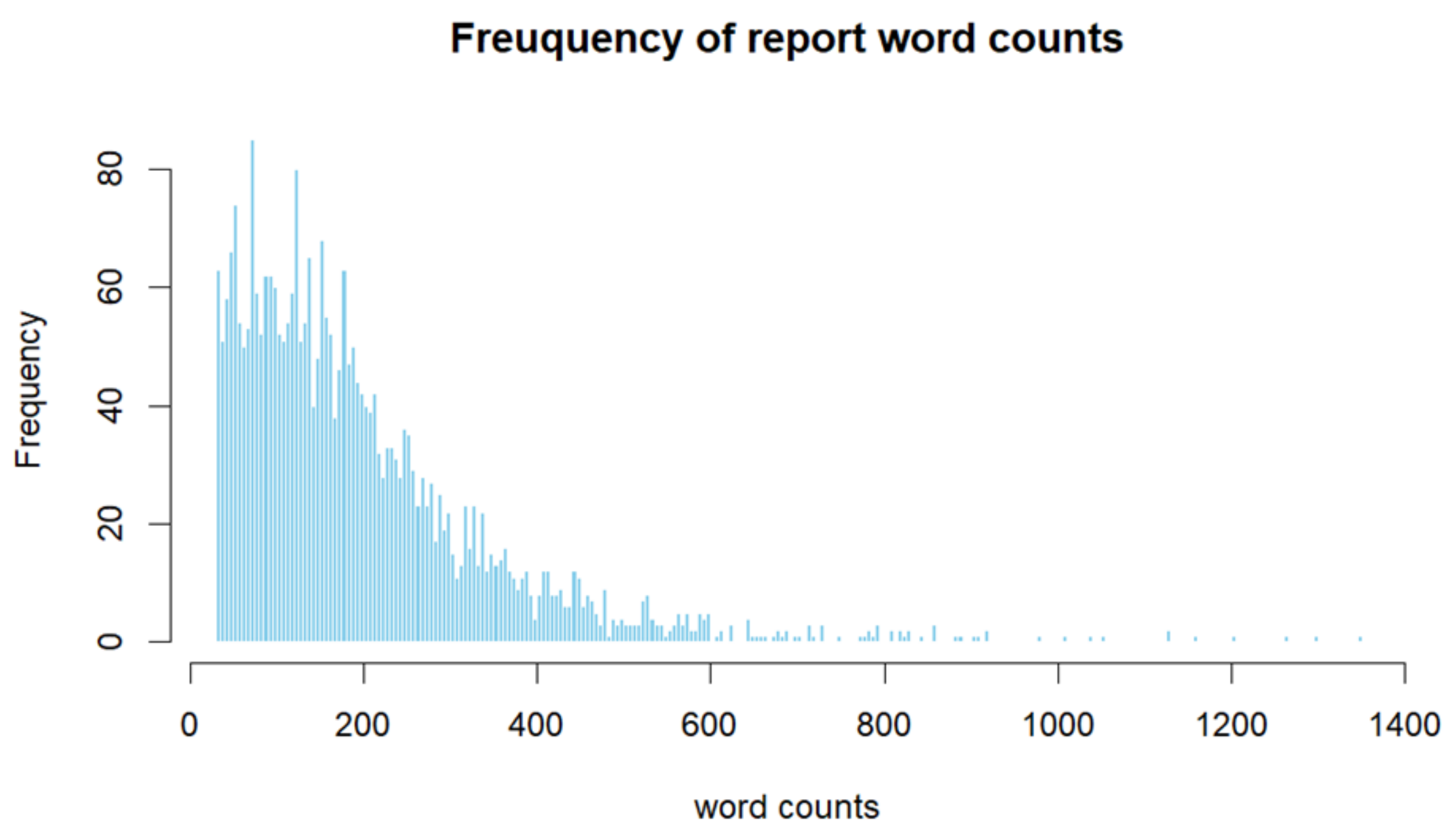

Please note that we excluded all reports with less than 30 words to ensure graph theory metrics were stable. Word counts were log transformed and z scored before being inserted in statistical models (supplementary material 2).

Figure S6 Frequency of reports per person.

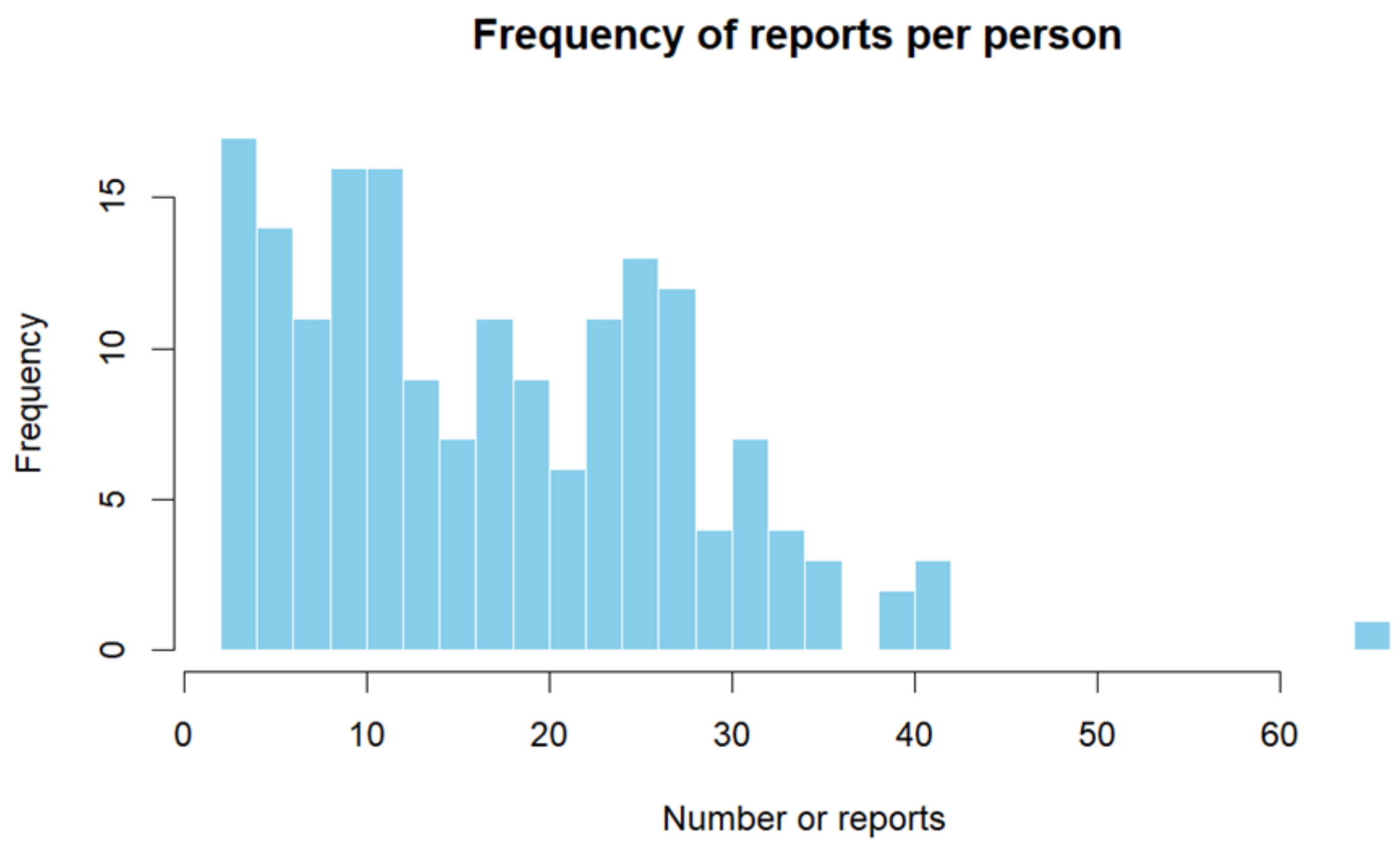

Figure S7 Frequency of dream reports per person

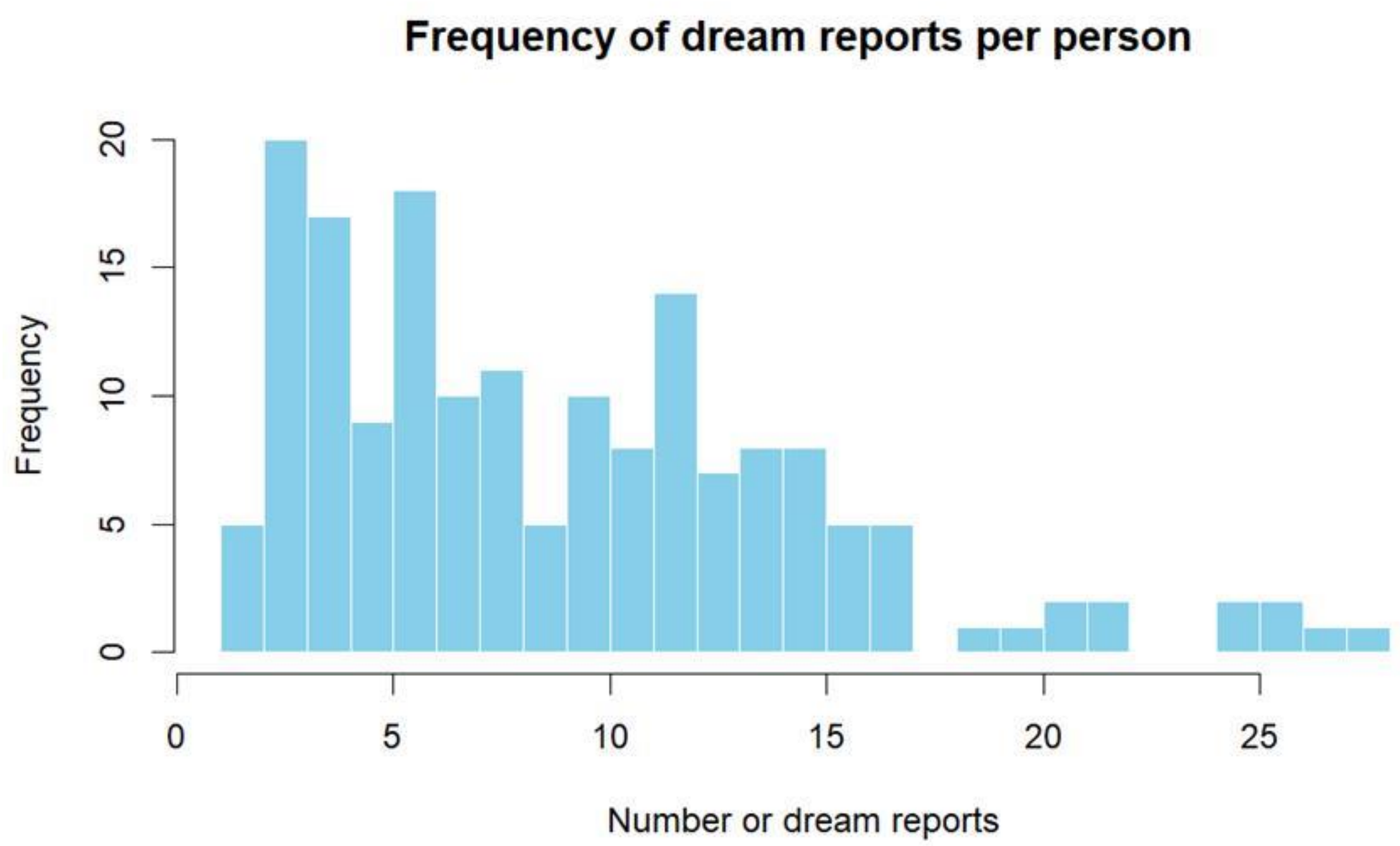

Figure S8 Frequency of mind wandering reports per person

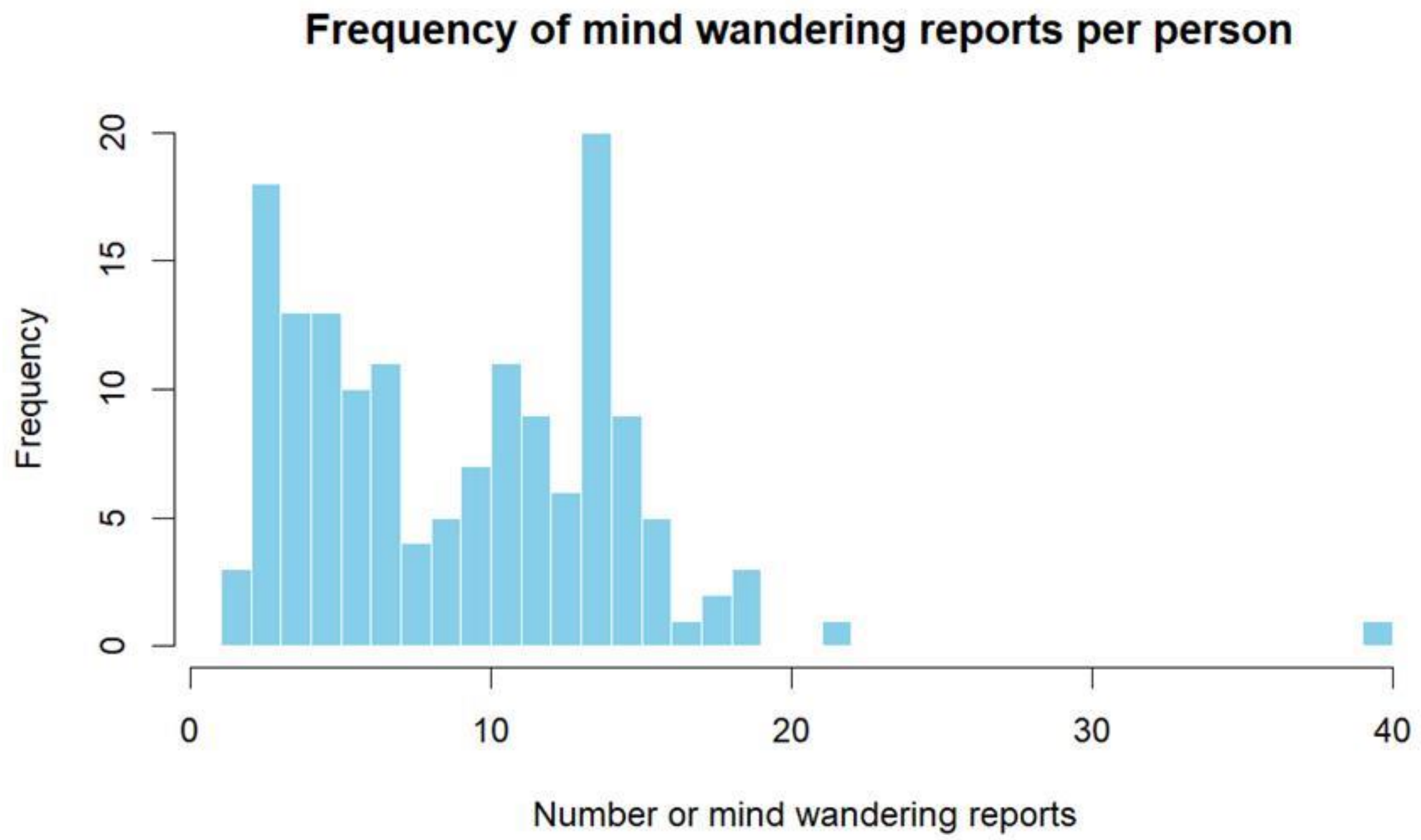

Please note that, as per previous work (Mota et al., 2012, 2014) (see (Mota et al., 2023) for a review of a decade of research with this methodology), we removed full stops at the pre-processing stage and therefore do not include information regarding sentence count. Future studies may look into this, however, our objective was to explore the methodology described in Mota et al., 2014 in relation to the present dataset. As discussed throughout the manuscript, we adopted several control analyses to ensure that word count did not influence the other results.

Why were three components extracted? Explaining 91% of the variance with a the first PC seems extremely high. Did the authors look at and elbow plot, etc. or follow some convention to make these decisions? Please provide more information on this with clear justifications based on prior literature. The loadings of the PCA features are also important to discuss.

Due to the way these particular networks are formed (word recurrence graphs, Mota et al., 2023), we knew that a high percentage of the variance would be explained by word recurrence. Collinearity is an issue for graph theory analyses studies more generally. Due to the expertise of some of our team members we knew this would be a problem and hence why we discussed this in the preregistration (https://osf.io/arx2h).

Of note is that the data reduction and finding differences between the two types of reports, should be considered as different issues altogether. The inclusion of a variable amount of PCs depended on their statistica performance in distinguishing the reports of interest, which was our objective. Such approces are discussed in the litterature (Sclove, 2021), which suggest that small eigenvalues should not be necessarily rejected, as they can still be strongly related to the response variable.

Below we also cite the procedure for using two principal component analyses in the statistical model, which is reported in the methods section. We found that the information criterion of the inferential analyses improved with two principal component analyses but was worsened by including three.

> Following preprocessing described above, we included PC1-density, age, country, gender, word count, assortativity, maximum betweenness centrality, and clustering coefficient in the statistical model. We also constructed two additional models, one adding PC2-Diameter, and another adding the third principal component in addition to the previously mention predictors. Through model comparison using “anova()” we found that the model with PC1-density and PC2-diameter provided the better fit (considering predictive power and parsimony, supplementary material 3). In fact, the Akaike information criterion was 3716.2 for the model without PC2 and 3700.9 for the model with PC2. We thus report the results that has PC2 as a predictor variable

As per the reviewer’s request, we report the pricipal component analysis with loadings in the supplementary materials. For the ease of the editor and reviewers we have inserted this below from supplementary material 2.

Figure S1- Correlational structure of graph theory variables.

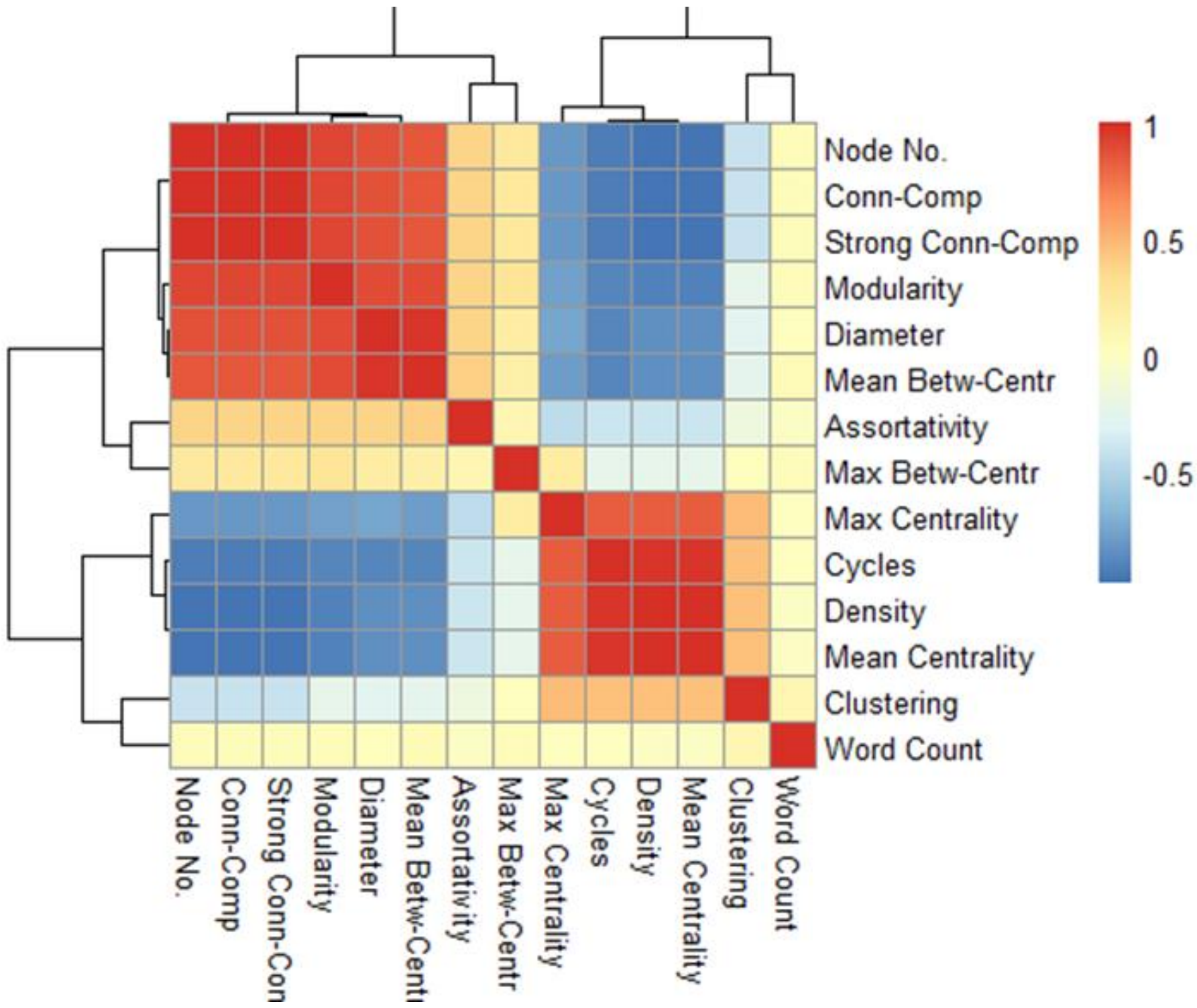

Of these variables, only the most highly correlated were inserted in the PCA, as the object of this was to reduce the highly correlated data into component that had sufficient independent variance to be included as covariates in a statistical model.

Percentage explained variance for PC1 was 90.98. For PC2 it was 3.94 whilst for PC3 it was 2.88

Table S2. Variable inserted in the PCA with loadings onto the principal components (sign also displayed). Highlighted in BOLD are the highest loadings onto PC1 and PC2

	PC1	PC2
Diameter	0.306	**-0.546**
Density	**-0.326**	-0.239
Node Number	0.324	0.264
Mean centrality	**-0.326**	-0.239
Maximum centrality	-0.285	0.045
Modularity	0.313	-0.114
Cycles	-0.320	0.045
Largest Connected component	0.324	0.264
Largest strongly connected component	0.324	0.264
Mean Betweeness centrality	0.305	**-0.600**

As discussed in the main text, all variables loaded more or less equally onto PC1. However, we note that a foundational source of variance for this type of network is word repetition, and thus we decide to interpret this primarily in terms of word-repetition/centrality.

In PC2, mean betweenness centrality has the highest loading whilst diameter is the second. This is due to the fact that both functions rely on calculation of shortest paths. We decided to interpret this primarily in terms of diameter as this is more intuitive and avoids confusion with another metric in the paper which is maximum betweenness centrality.

As well, how did the repeated measures aspect of the PCA come into play? The fact that people had different reports means that they were likely "weighting" the PCA. Please address this issue with more detail or updated analyses.

We thank the reviewer for their discerning comment. The purpose of the principal component analysis was for data reduction only to ensure our statistical models were parsimonious and did not violate statistical assumptions. The PCA permitted us to reduce 10 independent variables to two in our mixed model logistic regression. Then we obtained the participant specific coefficients and inserted these into the mixed model logistic regression. Thus, the repeated measures were controlled for at this stage, where the hypotheses were tested.

We appreciate however that the repeated measures might have affected the data reduction. We therefore reproduced the principal component analysis by averaging the data within participants to investigate whether repeated measures had a major effect on the data reduction.

Indeed, averaging all repeated measures had very little effect on the Principal component analysis. The most notable difference was that PC1 had a minor difference in explained variance (3.09%). Furthermore PC2 and PC3 were swapped due to minor differences in explained variation (1.7% and 0.23%, see table below). Importantly, the loadings were consistent (shown below). This indicates that, for the purposes of data reduction, the repeated measures did not have a significant effect on the summary metrics. We recognise that these small differences may have had an effect on the procedure described above involving the information criterion. However, of note these minor differences may be partially driven by the widely differing sample size (3054 vs 176) and there does not seem to be a straightforward way to tease apart these effects. We are satisfied that the data reduction was performed successfully for our purposes (as can be investigated given the correlations matrix in S2) and, importantly, that the differences between the types of report were adequately investigated. Importantly, the mixed nature of the data were taken into account when analysing data for hypothesis 1 and were further capitalised on for hypothesis 2.

Please see the plots and tables below describing this control analysis.

Table depicting percept explained variance of principal components with and without repeated measures

	PC1 in main text-	PC2 in main text (PC3 in averaged analysis)	PC3 in main text<br><br>(PC2 in averaged analysis)
Driven by	Density/mean centrality/node number	Diameter	Max degree centrality

With repeated measures	90.98%	3.943%	2.881%
Averaged across repeated measures	94.07%	2.243%	2.649%
Difference in explained variance<br><br>(With repeated measured > averaged)	-3.09%	1.7%	0.232%

Explained variance with repeated measures

Explained variance with data averaged across repeated measures.

Table with loadings, with and without repeated measures

	With repeated measures			Without repeated measures		
	PC1	PC2	PC3	PC1	PC2	PC3
Diameter	0.306	-0.546	-0.172	0.306	-0.150	0.539
Density	-0.326	-0.239	0.042	-0.328	-0.177	0.164
Node Number	0.324	0.264	-0.109	0.331	0.229	-0.167
Mean centrality	-0.326	-0.239	0.042	-0.329	-0.177	0.164

Maximum centrality	-0.285	0.045	-0.937	-0.265	0.807	0.510
Modularity	0.313	-0.114	-0.221	0.316	0.031	0.202
Cycles	-0.320	0.045	0.016	-0.313	-0.018	-0.088
Largest Connected component	0.324	0.264	-0.110	0.331	0.227	-0.168
Largest strongly connected component	0.324	0.264	-0.110	0.331	0.227	-0.168
Mean Betweeness centrality	0.305	-0.600	-0.051	0.301	-0.326	0.510

The above results of this control analysis have been briefly noted in the most recent version of supplementary material 2. As discussed, the mixed nature of this data (both repeated and independent measurements) was taken into account at the inferential level.

One issue is that the paper does not connect to prior work on using graph theory in language more generally. This work has been going for quite some time and seems like it would relate to the hypothesis — especially in relation to narratives etc.

We would like to thank the reviewer for their interesting comment.

We utilise a specific instantiation of graph theory analyses as developed by the co-author Natalia Mota and her team throughout the last decade. Natalia proposed over a decade ago (original paper Mota et al 2012, literature reviewed in Mota et al 2023) to model narrative trajectories as a word-reccurrence graphs, and was one of the pioneers in the field of computational psychiatry. She applied the method to understand not the content, but the structure of word-recurrence in dreams narratives of patients (allowing for the study of formal thought disorders related to psychosis). This method was replicated in several languages,

applied transdiagnostically and also to understand cognitive development: the effects of learning written language and influences on oral narratives in first and second language acquisitions (Mota et al 2023). We choose to use this method for being one of the first methods to study dream reports (Mota et al 2012).

But we are aware of different possibilities in the use of graph theory analyses

Different methodologies such as the following (https://knowledge.e.southern.edu/cgi/viewcontent.cgi?article=1215&context=crd(Ermer & Alférez, n.d.)) would imply largely different findings and variation of interest. This would afford and interesting comparative approach. Nonetheless, briefly, the definition of narratives and how graphs are constructed from them would have a large influence on the results and a detailed analysis of such methods is beyond the scope of the present article.

The present analysis has minimal preprocessing steps (e.g., does not require a theme recognition algorithm, or a somewhat arbitrary stop word list (further discussed below)). As it is not related to content but word-reccurrence structure, all the words are represented, as explained in Mota et al., 2023.

We can describe word sequence and its irregularities using psychopathological terms. However, with computational tools, we are now able to understand a narrative as a set of words organized in sequence from which specific meanings emerge. The original idea was to formally study the phenomena of planning a narrative using math tools to characterize these word sequences. Looking at this process (planning a narrative), we describe its final product (the narrative) as a system in which each element is a word, and the sequential information determines the recurrence pattern. It is an indirect way to characterize thought, which is assessed through language. In addition, because the tool does not consider any linguistic rules, theoretically, it is not restricted to any particular language. Moreover, it could be used to study the biological and social factors that affect these word sequences.

I noticed in the graph that some words I would expect to be removed were actually in the graph. In NLP analyses, we often remove "stop words" in order to address non-content words biasing results. I noticed in the Discussion this was pointed out as a limitation, but I would argue it is not a minor limitation that should be address in future work. Stop word removal is a pretty basic part of language processing that is almost always the first step along with lemmatizing, etc. The way this was done essentially values meaningless words and also does not allow for words like run, running, ran to be considered the "same" which is also a problem in my view.

We followed the procedure set out in previous publications by Dr Mota (who is a co-author on this paper) and her team. (Gomes et al., 2023; Martin et al., 2020; Mota et al., 2012, 2014, 2023). This can be an important issue for other methods that studies semantics and content, but this methods foccus on word-reccurrence trajectories, and all the words are important to represent topological organization studied. Again, we kindly ask to read the recent review in the methodology (Mota et al., 2023) to understand this view:

"The original idea was to formally study the phenomena of planning a narrative using math tools to characterize these word sequences. Looking at this process (planning a narrative),

we describe its final product (the narrative) as a system in which each element is a word, and the sequential information determines the recurrence pattern. It is an indirect way to characterize thought, which is assessed through language." (from Mota et al 2023) This method looks at analysing the writing output without changing it substantially.

There is also not a consensus as to which stop words to include. For example, often researchers will exclude words such as "I" and "me". However, plausibly, representing yourself as a subject or an object of a phrase (I and me respectively) may carry implications in various ways, (e.g., being correlated with greater sense of agency, internal locus of control) (e.g., Todd, 2014). Of interest is that these graphs are not sensitive to the content of the dreams and mind wandering reports, but rather the structures, word recurrence being the primary driving factor in these networks (as shown by the principal component analysis, supplementary material 2, Mota et al., 2023). The pattern of interest is given by verbal report itself and for the present method It is important to include all the words. For example, in psychosis (which has been variably and arguably comparable to dream experiences), pronouns act differently (Chaves et al., 2023). The process of removing such words might incurr in loss of information that is relevant to the phenomenon of interest

Future methodological studies may investigate the present method with alternative preprocessing steps. However this was beyond the scope of this study which intended to apply a specific and established technique (Mota et al., 2023) to a novel dataset (Sikka et al., 2023). Of interest is the fact that this method does not rely on substantial preprocessing methods. It also allows for more straightforward cross cultural comparison, not relying on specific language rules (Mota et al., 2023), as it is not based on specific corpora and does not rely on specific linguistics rules, as mentioned on this paragraphs extracted from the review:

Removing stop words would change the phenomenon per se': the narrative production and the choice of specific words in sequence. These topological dynamics develops during cognitive development in specific association with verbal memory (Mota et al., 2020), and social cognition (theory of mind abilities) and learning (specifically reading acquisition in native and a second language as well) (Finger et al., 2025; Malcorra et al., 2024; Mota et al., 2020). While producing oral or written narratives, mneumonic and cognitive processing are quite different, but from them the narrative production is the same: a sequence of words.

I understand the authors are saying that the anxiety scores are ordinal. However, they are very frequently used as continuous measures. I would like to see if the results replicate regardless of how the data is split, and I would also like to see the descriptives per category that was created based on the scoring

We are glad to say that we focus on results in the main text that were consistent across the large number of alternative analyses we ran. In fact, as can be inspected in supplementary material 4, there are likely several other effects that we do not report, as we decided to err on the conservative and robust side of interpretation.
The issue underlying this is that, regrettably, the mixed models did not converge in running these analyses (often the error being that models were "rank deficient", although in linear

models assumptions were also violated to an undeniable degree). To attempt to solve this issue, we ran many variations on this analysis, including using alternative cut offs, and using the self-reported mental health measures as continuous and binary. Despite all of these models largely replicated results, none of these ran without violating statistical assumptions, and consultations with statisticians and mathematicians did not solve the issue. The errors consistently indicated that the models were “rank deficient”, meaning that various models attempted could not estimate the random effect intercepts which were likely too unbalanced. When the criterion variables were treated as linear, the assumptions of the statistical tests were violated.

A substantial portion of reporductions (ten separate tables in total) are reported in supplementary material 4. However, we did not report all the analyses we ran with alternative cut offs and continuous definitions of the dependent variables, as this would require an inordinate number of tables. We do not copy the tables here as they would occupy a notable number of pages, however we are encouraging of anyone who would like to peruse the supplementary materials or attempt alternative analyses (data available from data availability statement)

Of importance to the reviewer’s observations, is that we do report non-parametric correlations in the main text, that effectively treat the criterion variables as continuous in the main text (Kendal’s tau was used rather than spearman’s rho as this allows for ties in the data).

We can confirm that, broadly, the results were convergent across all analyses, often with substantially higher effect sizes that the more conservative approaches adopted in the main text (supplementary material 4). This consistency increases our confidence in the effects. We would be happy to oblige any further requests for specific analyses output, however noting that any mixed models will be rank deficient. The data is shared (see data availability statement), thus anyone may attempt such analyses.

Following the reviewers comments, we have added the category counts in the dedicated supplementary materials as above.

Table S17 Counts for different categories of the PHQ-9 and GAD-7 following averaging across repeated measures.

	None	Mild	Moderate-Severe
GAD-7	88	52	36
PHQ-9	64	66	46

The categories are defined as per the official guidance of scale interpretation(Kroenke et al., 2001; Spitzer et al., 2006). The moderate and severe categories were collapsed to ensure increased balance between the conditions.

I am still not entirely sure how the mental health models were constructed. There is no way to estimate a multi-level model with a random effect when a person has only one value for the predicted value; the authors do acknowledge this. The authors should likely take an "average" of the graph theory metrics and then see how the aggregate measure relates to the scores. I was not clear if this was the approach taken (but I think it was); how the models were exactly constructed was not clear though.

We would like to thank the reviewer for their comment and pointing out a lack of clarity. Indeed, there was only one report per participant for the mental health scales. This may have contributed to the above mentioned issue (mixed models not converging) and, as per the reviewer's intuition, this was indeed solved by averaging all relevant independent variables (e.g., report specific word count, word recurrence, and other graph theory measures) in a participant specific manner. Further details on alternative models are present in supplementary material 4.

We did average across repeated measures to obtain one score per participant. We have modified the text to help with clarity

> We subsequently explored how the architecture of dream and mind-wandering reports predicts mental health symptoms, specifically, self-reported measures of anxiety (GAD-7) and depression (PHQ-9). Attempts to fit multilevel mixed models did not yield trustworthy intercept estimations, likely due to having one self-reported mental health measure per participant (176) rather than per report (3053). Although mixed models showed several significant effects (supplementary material 4), here we decide to adopt a highly conservative approach.
>
> We averaged across repeated measures to yield one value per individual and conducted analyses for dream and mind-wandering reports separately. We adopted a convergence strategy, and here report results showed consistency across several methodologies (see Methods and supplementary material 4), thus suggesting robust findings. Presented are results using ordinal logistic regressions and non parametric correlations. Alternatives are available in supplementary material 4.
>
> Across various analyses, we report the graph theory measure that was particularly stable. Specifically, the centrality of dream reports (i.e., word repetition) was likely to be associated with a higher PHQ-9 score (in ordinal logistic regression coef.=-0.47, [95% CI= -0.84, -0.10], p=0.013, Odds Ratio = 0.52; Kendall's tau 0.14, p=0.005; see Figure 3D). There was another reasonably stable trend that emerged across analyses (see supplementary material 4). The centrality of mind-wandering reports showed a relationship with the GAD-7 score which was non significant using Kendall's tau (in ordinal logistic regression coef.=-0.48, [95% CI= -0.94, -0.02], p=0.038, Odds Ratio = 0.62; Kendall's tau=0.10, p=0.06).

Other than adding confidence ratios and odds ratios, we noted that statistics for the ordinal logistic regression (without repeated measures) between PC1 and GAD-7 were misreported (this was not the case in the supplementary materials).

We have now amended this.

Reviewer #2 (Remarks to the Author):

This study compared and contrasted the reports of dreaming and mind wandering using graph theory applied to natural language. Participants were asked to report their dreams in the morning and report their mind wandering thoughts during the evening every day for up to two weeks. The authors found that dream reports have a more complex structure (i.e., small world architecture), whereas mind wandering reports have less word repetitions and more verbose structure compared to the other condition. These reports are more similar within subjects compared to between subjects. Finally, one of the measures, the word centrality component, in dream reports positively predict depressive symptoms.

This study addressed a longstanding question about similarities and differences between dreaming and mind wandering using sophisticated measures of graph theory. I think the results are novel and will appeal to both fields. The analyses of choice are generally valid and appropriate for addressing the research questions at hand and I appreciate the pre-registered analyses. I am impressed with the size of the dataset and that it was collected across two countries. Most of the results are convincing while others appear less robust (which I elaborate below). I raise some questions about the methods and statistical analyses, and the interpretations about some of the results, with the aim to help improve the clarity and comprehensiveness of the manuscript. Please don’t take the number of comments to reflect my enthusiasm for this paper, as I believe the paper has good potential.

We would like to kindly thank the reviewer for their encouraging comments and their effort in improving the manuscript. We have tried our best to implement their comments and suggestions.

GENERAL

Succinctness of Introduction/Discussion

Although I find the summaries of dreaming and mind wandering interesting, I did ponder several times throughout the introduction, the relevance of some the background literature. To that end, I have a few suggestions.

I think a more focused introduction would help prepare the readers for the research question when they reach the “Present Study” section. As an example, I suggest making the third paragraph (Lines 68-84) more succinct.

Following the reviewers comments we have shortened the paragraph describing literature on differences between mind wandering and dream experiences.

> Yet, the two phenomena occur in different behavioural states and are different states of consciousness(Carhart-Harris et al., 2014; Laureys, 2005). Dreams are characterised by a lack of reality testing(Voss et al., 2014) , greater bizarreness and

> unstructured content(Kahn, 2013; Williams et al., 1992), and are often considered a hallucinatory phenomenon(Occhionero & Cicogna, 2016). In contrast, mind-wandering may involve varying degrees of intentionality, and executive control(Christoff et al., 2016; Domhoff & Fox, 2015; Occhionero & Cicogna, 2016; Seli et al., 2016; Smallwood & Schooler, 2015). Researchers have even challenged the notion that mind-wandering is a unitary phenomenon(Seli et al., 2016, 2018), noting variations in attentional neural systems, intentionality(Boulakis et al., 2023; Murray & Krasich, 2022; Seli et al., 2016) and affective outcomes(Kam et al., 2024).

Having read the Discussion, I see why the authors wish to introduce concepts of reality testing etc here, but without knowing the results, this does not seem directly related to the 'architecture' of dreaming. The same applies to the section on individual differences. This section primarily summarizes findings involving personality traits, even though the manuscript does not implement measures of personality traits. I understand authors use this as a measure of individual differences, but it gives the illusion that personality traits will be examined. I also note it's not clear how some of the findings tap into individual differences so stating the link more explicitly would be helpful.

We understand the reviewer's concern that the cited literature may seem relevant only in hindsight. However, in our pre-registration, we proposed a possible analogy between dreams and schizophrenia (https://osf.io/arx2h). This analogy is not only based on phenomenological similarities, such as imparited reality testing (which only occurs in lucid dreams) and lack of insight, hallmark feature a psychosis, but also in terms of neural correlates (e.g., prefrontal deactivation). That said, we do not intend to equate dreams with schizophrenia, but rather to highlight an analogy that may offer useful insights into both phenomena.

As per the suggestion of another reviewer, we have inserted the hypotheses at the end of each hypothesis-specific paragraph. We hope that this adjustment will help readers better understand the relevance of comparing the repots of dreaming and mind-wandering, and how examining their similarities and differences contributes to the wider interdisciplinary discourse. We have also applied this to the second and third paragraphs, ensuring that our hypotheses are framed within a broader context.

Regarding individual differences, we use the term in its most general sense. To clarify this, we have inserted a definition within the relevant paragraph:

> An important question is to what extent individual differences (stable characteristics that distinguish individuals from one another) underlie the content and structure of dreams and mind-wandering.

Our hypothesis as defined at the end of the paragraph intends to make the objective clearer

We have also introduced an interpretation in the discussion section that echoes the above definition of individual differences:

Importantly, we found that within-individual similarity between dream and mind-wandering reports was higher than between-individual similarity. This suggests that this method is sensitive to stable characteristics that distinguish individuals.

• **Similar to the Introduction, I think there may be some ways to make the Discussion more concise. For example, the more complex structure of dream reports was already discussed in the previous page, so it would make more sense to include the discussion of this finding in the paragraph above, versus interpreting the results twice across separate non-contiguous paragraphs.**

We have now reduced the text and reorganized it to ensure that we discuss complexity in one unified paragraph. The additional descriptions of the small world network were brought on by the reviewer's comments below:

We found that dream reports have a more complex structure, exhibiting the computational elements of a small-world architecture(Barahona et al., 2002; Milgram, 1967; Watts & Strogatz, 1998). This network structure is characterised by clustered, interconnected neighbours (higher clustering coefficient) and a simultaneous ease in long-distance communication (lower path length). Although the effect sizes were small, this suggests that dream reports had more local clusters (neighbouring words also tended to be neighbours amongst themselves), but were, overall, also more interconnected (there were words that created shortcuts, that facilitated efficient transitions across the network).

The multifaceted and somewhat unpredictable nature of experiences that may be had in dreams may somewhat explain their higher structural complexity(Occhionero & Cicogna, 2016; Windt, 2019). In fact, variably, dreams can be rather bizarre(Kirberg, 2022; Rosen, 2018), and may be considered comparable to psychosis in terms of a lack of reality testing and bizarreness(Occhionero & Cicogna, 2016), and analogous alterations in prefrontal cortex functions(Fox et al., 2013; Voss et al., 2014). Furthermore, analogies have been drawn between dreaming and psychosis, as both may relate to a breakdown of higher-order predictions(Carhart-Harris et al., 2014; Carhart-Harris & Friston, 2010; Friston, 2010). In fact, texts produced by schizophrenic individuals(Mota et al., 2014; Noreika et al., 2010) and individuals under the influence of LSD(Sanz et al., 2021) show similar characteristics to dream reports (i.e., a small world strcture, lower diameter and clustering coefficient). In schizophrenia, these elements may be partially explained by the cognitive difficulties and negative symptoms associated with such disorders(Mota et al., 2014, 2023). By analogy, there may be difficulties in accurately recalling experiences from one state of consciousness (dreaming) when in another state of consciousness (wakefulness)(Carhart-Harris et al., 2014). The cognitive divergences between such states may therefore somewhat explain the convoluted and complex structures of these types of text.

We have further removed information in discussion

~~Thus, potentially, themes are less stable, and more numerous, in mind-wandering reports, whilst being more constant in dreams reports. This is in consonance with the~~

~~fact that mind-wandering reports have higher diameter, indicating that the report is less integrated (interconnected over long distances via specific words) and is related to the more linear (rather than recursive), extensive, and less integrated structure.~~

Terminology

This paper appears to use mind wandering throughout the manuscript in a way that may be inconsistent with the general definition of mind wandering in the field. Specifically, the methods indicate that subjects were asked to think about whatever comes to mind without specific goals in a distraction free environment (reminiscent of a resting state instruction) and then to report their thoughts. This would fit better with the definition of spontaneous thought than mind wandering, in particular based on the papers cited in this manuscript (Smallwood & Schooler, 2015; Seli et al., 2016), all of which tend to define mind wandering as "task-unrelated thought". Given the subject's task is to let their thoughts wander, this would not be consistent with this common definition of mind wandering. I suggest the authors choose a different term, or explicitly define mind wandering in their own way that aligns with what the task captures early on in the introduction.

We would like to thank the reviewer for their comment which is reminiscent to those made by the reviewer above.

To ensure clairty as to the operationalisation, in the present study subsection of the introduction, we detail the instructions given to participants.

> For the mind-wandering reports, we instructed participants to let their mind wander freely and subsequently write down what went through their mind during this period.

Earlier in the introduction, we explicitly state how our operationalisation is derived from the spontenous thoughts framework:

> Researchers have even challenged the notion that mind-wandering is a unitary phenomenon(Seli et al., 2016, 2018), noting variations in attentional neural systems, intentionality(Boulakis et al., 2023; Murray & Krasich, 2022; Seli et al., 2016) and affective outcomes(Kam et al., 2024). In this study we operationalise mind wandering using the spontenous thoughts dynamic framework(Christoff et al., 2016).

We have also added an explicit discussion of relevant conceptual variations in the newly added limitations section.

> Of note is that this study instructed participants to engage in mind-wandering during a set period, providing somewhat of a contrast to definitions that highlight task-unrelated aspects of mind-wandering(Murray & Krasich, 2022). The various degrees and multidimensionality of intentionality and executive control in mind-wandering are variably discussed in the literature(Murray & Krasich, 2022; Seli et al., 2016, 2018), and could not be adequately explored in the present study. However, for this study (Sikka et al., 2023), we employ a definition of mind-wandering that is encompassed by the spontaneous thoughts framework(Christoff et al., 2016), which focuses on the

dynamics of how thoughts unfold overtime. This is analogous to operationalisations used in resting state functional magnetic imaging studies where participants are instructed to let their minds wander(Gonzalez-Castillo et al., 2021). Such a definition was deemed to allow a greater comparability between the two conditions of interest in the present study (i.e., dream and mind-wandering reports)(Christoff et al., 2016).

Depth of Result Interpretation in the Discussion

In the interpretation of results, I think the manuscript will benefit greatly from interpreting the results in a concrete way. For example, starting from Line 365, authors found a small world architecture in dream reports. Instead of (or in addition to) summarizing what small world architecture reflects in the context of graph theory, I think it would be much more meaningful to describe what that means in terms of dream experience. For example, what does it mean for a dream to show more clustered, interconnected neighbours? If possible, sharing an example or an actual excerpt from the study would be helpful. The same applies to interpretation of the mind wandering result (Line 376 onwards).

The advantage of network science is that it enables us to extract summary metrics in very complex and naturalistic data such as this. However, beyond a literal interpretation as to what this means for the structure, we cannot provide further interpretation that we can ensure applies to all reports given the variety and complexity of these. Nonetheless, we refer to figure 1 which has a visual explanation of the metrics in the report structure . Furthermore table 1 has non technical explanations and we further refer to this.

As suggested by the reviewer we have now added the following clarifications.

> Although the effect sizes were small, this suggests that dream reports had more local clusters (neighbouring words also tended to be neighbours amongst themselves), but were, overall, also more interconnected (there were words that created shortcuts, that facilitated efficient transitions across the network).
>
> Whilst dream reports were more recursive and complex, mind-wandering reports had a more linear, extended structure with essential transition points in the report structure (higher max betweenness centrality). These are connecting words that create "shortcuts" that connect the network in the more linear and extended structure that characterised mind-wandering reports. Importantly, mind-wandering reports also had less repetition of words (i.e., centrality), which may be of a syntactical or semantic nature (Mota et al., 2023).

Consideration of Alternative Explanations

I'd like to suggest a few points of consideration to be discussed in a limitation section. First, could time of day of the report play a role in some of the differences observed between mind wandering and dreaming? For at least some individuals, they may be more alert while completing the mind wandering task and providing a report on their thoughts in the evening whereas they may be less alert when providing the dream report in the morning. I'm not sure the authors can address this, unless they

have measure of hours and quality of sleep, as well as individuals' work hours (9-5pm vs graveyard shifts). But it would be important to discuss.

Following the reviewer's comment we have inserted the following in the newly added limitations section:

> Of note is that the time of day may have had an effect on the reports. Beyond recency effects on recall, some participants may have been more alert whilst completing the mind wandering tasks, thus perhaps explaining the higher verbosity in such naturalistic data. Furthermore, individual differences in circadian preferences may have had further effects.

A related point concerns whether control measures or post-report question were implemented to ensure that 1) the environment in which participants allowed their minds to wander was in fact free of distraction and 2) the thoughts were not restricted by any specific goal (either through self-report or experimenter coding)? If so, please report and if not, this would be important to discuss as a limitation (as this impacts the 'spontaneity' of their thoughts). Consider an example in which the participant was in a coffee shop and their thoughts were selectively drawn to the conversation between two friends sitting next to the participant.

Even though participants were instructed to carry out the mind wandering task in distraction-free environment and were asked to "allow your mind to wander freely from thought to thought without any specific task or target for a ten-minute period", we did not include any post-report questions to confirm whether they had fully adhered to these instructions. Therefore, we have added the following to the Limitations section:

> Furthermore, although the participants were instructed to find an environment free from distractions, we did not employ post-hoc measures evaluating distractions. Such measures may be a consideration for future studies.

As indicated in the methods section, the instructions to the participants specified mind wandering as "contents that enter our mind when we are not engaged in or focused on any specific activity or task".

Line 214. The decision that additional features of text explained 'sufficient unique variance' based on correlations with other metrics <25% seems somewhat arbitrary. I'm not familiar with past literature that included both principal components and individual features in the same model. Can authors verify that this is appropriate mathematically and there are previous literature suggesting <25% is considered sufficiently unique to include in the same model with principal components derived from the same data? Related to this, can authors provide some past literature (on

statistics and literature adopting this approach) that suggests r < 0.5 indicates independent variance (Line 231)? Some clarification on how these values were decided on would be helpful, as I did not see any information about this in the pre-registration.

The reviewer is right in that this was an arbitrary cut off that erred on the conservative side. Our objective here was to reduce the relevant graph theory metrics and avoid multicollinearity.

The rules of thumb for too much correlation between independent variables found online vary. However, in the past "Some investigators use correlation coefficient cutoffs of 0.5 and above" (P. Vatcheva & Lee, 2016). All these cutoffs are arbitrary and there is no universal agreement. Either way, with the data reduction methodology employed, any independent variance of interest within the variables would have been expressed in principal components.

In view of the correlation between the variables inserted in the PCA, alternative cut offs would not have changed the outcome.

> Subsequently, a correlation matrix revealed that maximum-centrality, number of cycles, density, mean centrality, diameter, mean betweenness centrality, modularity, node number, largest connected component and strongly connected component were all highly correlated (R~0.7-1) (*Fig 1D)*

At the time, the particularly stringent cut off was thought to be a good approach given the use of data reduction methods and the complexity of unbalanced repeated measures in multilevel models.

Regarding the use of both principal components and other "non-reduced" data in multiple regression, this should not constitute a problem unless the assumptions are violated (e.g., multicollinearity). In the pre-registration we predicted this would be an issue. Keeping the original graph theory metrics without including them in the PCA would enable higher interpretability in this study and for future studies using similar methods. Either way, please see a reference that uses an analogous approach to ours, (Liu et al., 2022).

Line 244. The reporting of the logistic regression seems to be inconsistent with the nature of these analyses. Specifically, logistic regression compares the odds of something happening. In this case, if I understand correctly, it is whether a report predicts mind wandering or dreaming. So the precise language I believe is that a certain network architecture feature has a higher probability (or betters odds) of characterizing a mind wandering than a dreaming report. It's a nuanced difference in reporting but I think this more precisely and accurately captures the analyses implemented.

To ensure clarity and precision, we have revised the language throughout the Results section:

> A mixed-effect logistic regression showed that lower density/centrality (i.e., less repetition of words; coef.= 0.83, p<0.001), higher diameter (more difficulty to get from

one side of the text to the other (coef.= -0.19, p<0.001), and more words (coef.=0.14, p=0.002) had a significantly higher probability of characterising mind wandering reports. Maximum betweenness centrality, indicating the presence of words that connect different parts of the text (via shortest paths), was also more likley to characterise mind wandering over dream reports (coef.= 0.42, p<0.001).

Specifically, the centrality of dream reports (i.e., word repetition) was likely to be associated with a higher PHQ-9 score (in ordinal logistic regression coef.=0.47, p=0.012; Kendall's tau 0.14, p=0.005; see Figure 3D).

Figure 2. The group level mean of PC2 for dreaming and mind wandering seem very similar. Yet the result is highly significant (despite a small coefficient). Although a large data set such as this one is valuable, it also does render very small effects significant by nature of the sample size. Can authors discuss the conceptual significance of their results for PC2, taking into consideration this issue? It's not clear the effect would be replicated in a smaller sample. This is particularly concerning given PC2 accounted for <5% of variance in the data.

We agree with the reviewer that the substantial sample size will increase statistical power inordinately.

In view of this we have added in the limitations section

> Whilst the large sample size is a strength, this may have allowed to find small effects that would not be found with smaller sample sizes (e.g., PC2, Clustering coefficient and word count in figure 2).

As discussed above, we included the PC2 in our statistical analyses due to the fact that it improved the model as per AIC, as it retained predictive power in distinguishing dream and mind wandering reports after data reduction. Of note is that the amount of explained variance would have been different if other variables were included in the PCA (this depends on the correlational structure of the variables included and changes with the amount of variables included). Arguably, whether PC2 explains a difference between mind wandering and dream reports and whether it explains covariance in the data included in the PCA are different questions.

Of note is that the clustering coefficient and diameter are the components of the small world network, following the reviewers comment we have added this caveat in the discussion.

> Although the effect sizes were small, this suggests that dream reports had more local clusters (neighbouring words also tended to be neighbours amongst themselves), but were, overall, also more interconnected (there were words that created shortcuts, that facilitated efficient transitions across the network).

Line 277. Can authors report the number of data points of participants with both

dream and mind wandering reports? I'm specifically wondering about whether the data is balanced in the analyses addressing H2.

The reviewer is right that the data used in H2 is highly unbalanced. However, we were aware of the issue. The samples compared are highly unequal due to the method used (e.g., comparing every possibly within person comparison against every possible between-person comparison). This allowed us to utilise all the available data and not have to select arbitrary subsets of the data.

We therefore utilised the Mann-Whiteny U test which is robust to unequal sample sizes. An issue may arise with reduction of statistical power due to unequal sample sizes, however, that is clearly not the case in this instance.

Following the reviewers comments, we have inserted the numbers of each type of comparison in the newly inserted supplementary material for the descriptive statistics.

Table S16 Numbers of comparison within and between persons for dreams and mind wandering reports for Hypothesis 2.

Within and between comparison	Type of report	Number of comparison
Within individual	Dream	9654
Within individual	Mind wandering	8250
Within individual	Between Dream and Mind wandering	16888
Between individuals	Dream	1300117
Between individuals	Mind wandering	1019211
Between individuals	Between Dream and Mind wandering	2304758

The number of participants per type of report can be found in the participants subsection of the methods section. For the ease of the reviewer, we have copied it below.

> After excluding the above, 1781 dream reports from 172 participants (25 men, 139 women, eight “other”; mean age 46.99±14.80 years, range 19–78) and 1496 mind-wandering reports from 152 individuals (23 men, 123 women, 6 “other”, mean age 48.42 ± 14.71 years, range 19-78) remained. In accordance with the graph theory method used (see below), reports that did not result in a minimum of 30 words were excluded. Additionally, an outlier observation was removed to ensure that statistical assumptions were met. As a result, in the present study we analysed 1619 dream reports (172 participants) and 1434 mind-wandering reports (152 participants) from a total of 176 individuals (4 participants provided mind-wandering reports but no dream reports).

As shown above, we have added a new supplementary material with tables and figures describing the data.

We have also added the following to the methods section.

> We then investigated if the reports written by the same person were more similar compared to the reports from other individuals using two-tailed Mann-Whitney U test which is suitable for unequal sample sizes. In total there were 9654 within individual comparison for dreams and 8250 for mind wandering reports. There were 16888 within-individual comparisons between dream and mind wandering reports. As for between individual comparisons there were 1300117 for dreams reports and 1019211 for mind wandering reports and 2304758 between dreams and mind wandering reports (supplementary material 6).

Line 327. That multilevel mixed effects models did not yield robust results but repeated measures did is noteworthy. Typically, in accounting for within subject variance, multilevel mixed effects are more powerful. This contrasts with obtaining a large amount of data and narrowing it down to a single value per individual. Can authors address this in the Discussion, and speculate why this may be the case, and what this means in the context of predicting mental health status?

The issues are not about statistical power, but about model convergence and the fact that the models could not estimate the intercepts successfully (the most common error was that the model was “rank deficient”). In fact, the mixed model results, presented in supplemenatry material 4, show statistical significance and indeed were more powerful, as the reviewer suggested. We consulted with mathematicians and bio statisticians to solve this problem.

We thus decided to err on the side of caution and reduce the data, and adopt a convergence approach to enable robust inferences. Variations in the analyses are presented in supplementary material 4. However, we did not insert all analyses we tried, as this would produce an inordinate amount of tables.

The fact that the intercepts are dropped indicates that the mixed nature of the data (and our attempt to model this in the statistics) was the cause of the problem. Another issue may be that we had one dependent variable per participant, but several reports per participants. Despite various attempts, and alternative analyses, we were not able to even ascertain with absolute certainty the origin of the cause. Either way, we are confident that the results presented are robust. There are in fact several other variables that were significant with the mixed model method, however, as aforementioned, we decided to go with a conservative approach.

We do not copy modifications to the main text here as they are copied above and below.

Line 374. Given the importance of the notion of node centrality (or word repetition), can authors elaborate on the last sentence of this paragraph? This seems crucial for understanding what this means for dreams by linking to other literature. Although the links are reported here, the actual relationship between node centrality and cognitive development/social cognition/etc are not described. This may help the authors more fully interpret and contextualize the findings.

We thank the reviewer for their comment. This is indeed a very important point. There is in fact a recent publication (Mota et al., 2023) that reviews the research using this method and how it relates to social-cognitive development. However, this research is extensive and a full summary of this in the present article may not be advisable to maintain the manuscript focused. In the main text we have modified the wording to explicitly refer the reader to the review so that they are able to read this should they be interested.

> In fact, in a recent review, the relationships between word recurrence structure and cognitive development, social cognition, and verbal memory in a typical population are elucidated (Mota et al., 2023).

The absence of an extended summary of this research field may be further justified by an absence of relevant variables to empirically dissociate various components of individual differences in the present article. We nonetheless acknowledge this in the limitations section

> Although we find that the present method is sensitive to individual differences, effect sizes were small and we could not exhaustively elucidate what might be driving such differences. For example, differences in aspects of executive function (e.g., inhibition vs verbal fluency), ethnicity, and socioeconomic status may have influenced the structure of dream and mind-wandering reports.

Line 405. Have unpredictable or bizarre or unrealistic experiences been linked to more complex network structures? If so, this would be helpful to cite here. Otherwise, it would be helpful if authors elaborated on this speculation as to why more bizarre or unpredictable experiences have more complex structures.

We thank the reviewer for their comment. In the text, we draw analogies between dream experiences and texts produced in various states that can be characterised as relating to bizarre experiences (e.g., psychedelic-induced and psychotic states). Of note, we make this

prediction in the preregistration (https://osf.io/arx2h), although admittedly, this is not as elaborated as the discussion within this article. We have made some small changes to make the analogies more explicit.

> The multifaceted and somewhat unpredictable nature of experiences that may be had in dreams may somewhat explain their higher structural complexity (Occhionero & Cicogna, 2016; Windt, 2019). In fact, variably, dreams can be rather bizarre (Kirberg, 2022; Rosen, 2018), and may be considered comparable to psychosis in terms of a lack of reality testing and bizarreness (Occhionero & Cicogna, 2016), and analogous alterations in prefrontal cortex functions (Fox et al., 2013; Voss et al., 2014). Using the same method, REM dream report structures have been shown to be more complex than non-REM dream reports (Martin et al., 2020).
>
> [...]
>
> In fact, texts produced by schizophrenic individuals (Mota et al., 2014; Noreika et al., 2010) and individuals under the influence of LSD (Sanz et al., 2021) show similar characteristics to dream reports (i.e., a small world structure, lower diameter and clustering coefficient). In schizophrenia, these elements may be partially explained by the cognitive difficulties and negative symptoms associated with such disorders (Mota et al., 2014, 2023). By analogy, there may be some difficulty in accurately recalling experiences had during a particular state of consciousness (e.g., during a dream) into a more “normal” one (Carhart-Harris et al., 2014).

We also interpret these results in the context of the free energy principle/predictive coding/ entropic brain hypothesis.

> Furthermore, analogies have been drawn between dreaming and psychosis, as both may relate to a breakdown of higher-order predictions (Carhart-Harris et al., 2014; Carhart-Harris & Friston, 2010; Friston, 2010).

Again, we cannot access the structure of experiences directly, but the reports, which is a notable problem across any scientific study of experience (Block, 2019).

Line 453. Given the results were not significant using multilevel mixed effects models and the coefficient is not large (or the p-value is not as highly significant as other effects), I would tone down this interpretation. In addition to the relatively unreliable effects, the study also does not address the mechanism by which higher word repetition in dream (but not mind wandering) reports was related to depression. Based on the speculated explanation, why may this be only observed in dream but not mind wandering reports?

Please excuse the lack of clarity.

Thus, the reviewer is perfectly right in that the mixed models have more power, in fact, in our analyses, multilevel models were far more significant, just not reliable due to the issue in estimating intercepts.

Extensive statistical details are not copied below but are available in supplementary material 4 if the reviewer would like to investigate.

Importantly, we decided to be highly cautious and conservative, and report only the minimum common denominator that survived across all variations of analyses. Thus, arguably, our results are stable, as they reproduce across several analysis permutations. Regrettably, several effects that would have been significant in a mixed model, did not reproduce in the analyses with no repeated measures, likely due to a lack of power. Our wording in the paper reflects this caution. However, thanks to the reviewers comments, we now see that this wording came at the expense of clarity. Hence, we have changed the wording in the following manner.

> We subsequently explored how the architecture of dream and mind-wandering reports predicts mental health symptoms, specifically, self-reported measures of anxiety (GAD-7) and depression (PHQ-9). Attempts to fit multilevel mixed models did not yield trustworthy intercept estimations, likely due to having one self-reported mental health measure per participant (176) rather than per report (3053). Although mixed models showed several significant effects, we decided to adopt a highly conservative approach, and report results that are stable across analyses.
>
> We averaged across repeated measures to yield one value per individual and conducted analyses for dream and mind-wandering reports separately. We adopted a convergence strategy, and here report results showed consistency across several methodologies (see Methods and supplementary material 4), thus suggesting robust findings. Presented are results using ordinal logistic regressions and nonparametric correlations. Alternatives are available in supplementary material 4.
>
> Across various analyses, we report the graph theory measure that was particularly stable. Specifically, the centrality of dream reports (i.e., word repetition) was likely to be associated with a higher PHQ-9 score (in ordinal logistic regression coef.=-0.47, [95% CI= -0.84, -0.10], p=0.013, Odds Ratio = 0.52; Kendall's tau 0.14, p=0.005; see Figure 3D). There was another reasonably stable trend that emerged across analyses (see supplementary material 4). The centrality of mind-wandering reports showed a relationship with the GAD-7 score which was non significant using Kendall's tau (in ordinal logistic regression coef.=-0.48, [95% CI= -0.94, -0.02], p=0.038, Odds Ratio = 0.62; Kendall's tau=0.10, p=0.06).

We noted that the statistics describing the relationship between mind wandering and PC1 were misreported in the main text (but not in the supplementary materials). This has not been amended. Please excuse this oversight.

We cannot be completely sure as to why mind wandering might show weakened effects compared to dreams.

This may be related to explanations via continuity theory (Topics of dreaming tend to be related to contents engaged with in waking life). There may be effects with encoding and recall, and even moderating variables of this (e.g., alcohol consumption) which may partially

explain such a difference. Similarly, mind wandering, as stated in the introduction, can be quite variable in its nature, and such a variability may partially explain such an effect.

However, of note, as shown in supplementary material 4, there tended to be stronger effects for mind wandering reports than those reported in the main text across the various analyses performed to assess the robustness of results. We opted to report the most conservative statistics.

Following the reviewer's comment, we have changed the interpretation in the discussion to reflect the above.

> Capitalising on this unique and extensive dataset, we found that graph theory analysis of natural language may relate to mental health symptoms, in accordance with previous literature (Mota et al., 2023). The issues in reliably estimating mixed models likely severely reduced predictive power of several variables (see supplementary material 4 for more details). In the present article we take a particularly conservative approach.

Is the repeated measures analysis implemented post hoc and not in the preregistration? if so, this needs to be explicitly stated.

We explicitly state that we would use mixed level models, in combination with a data reduction technique, and that we would use averages incase the unbalanced nature of the data induced unreliable statistics.

Regrettably, we did not explicitly include that we would attempt to use a mixed model in H3, but rather generally refer to a "multiple regression". This lack of specification seems to be an oversight.

We have added the following

> We performed mixed model analyses that permitted an ordinal outcome (not explicitly stated in the pre-registration). Across different types of models that were suitable to this question (e.g., ordinal logistic regression, cumulative link models), there was an inability to estimate the random effect intercepts in a stable manner.

However, we do state that we would take independent variables from previous hypotheses which can imply that a mixed model would be used.

Line 488. I think it would be important to add a limitations section. For example, how well might these results be generalizable beyond the current sample. The authors reported age, gender and years of education. What about ethnicity?

As per the reviewers' helpful suggestions, we have created a limitations section. Please see it below, copied in its entirety. In this we do discuss various individual differences. Regrettably, we did not collect ethincity data and mention this below.

> **Limitations.**

The writing of a verbal report of an experience (such as mind-wandering and dreaming) poses the problem that it requires the recall and then a verbal description of a qualitative experience. Such an issue relates to any verbal report. As described above, therefore, it becomes difficult to distinguish between effects that are driven by verbal reporting style, type of recall, distance of recall from the experience, various elaborations of the experience, etc. Nonetheless, the study was designed to render the two experiences in question as comparable as possible regarding the reporting of experiences (Sikka et al., 2023). The qualitative differences in recall may have had an effect on the data (e.g., from different states of consciousness, (Laureys et al., 2008)). These may be considered a relevant difference between dream and mind-wandering reports, and perhaps telling on the debates as to extent to which mind-wandering and dreams are continuous (Christoff et al., 2016). Of note is that the time of day may have had an effect on the reports. Beyond recency effects on recall, some participants may have been more alert whilst completing the mind wandering tasks, thus perhaps explaining the higher verbosity in such naturalistic data. Furthermore, individual differences in circadian preferences may have had further effects. Rather than the present natural language, big data approach, future studies may consider a more laboratory based approach, where mind-wandering experience is sampled via a think aloud paradigm and a wake-up paradigm is utilised for dreams. However, such an approach would still suffer from a difference in the reporting of an experience (engendering a second "recall" and "verbalization" experience) versus the experience itself.

Of note is that this study instructed participants to engage in mind-wandering during a set period, providing somewhat of a contrast to definitions that highlight task-unrelated aspects of mind-wandering(Murray & Krasich, 2022). The various degrees and multidimensionality of intentionality and executive control in mind-wandering are variably discussed in the literature (Murray & Krasich, 2022; Seli et al., 2016, 2018), and could not be adequately explored in the present study. However, for this study (Sikka et al., 2023), we employ a definition of mind-wandering that is encompassed by the spontaneous thoughts framework (Christoff et al., 2016), which focuses on the dynamics of how thoughts unfold overtime. This is analogous to operationalisations used in resting state functional magnetic imaging studies where participants are instructed to let their minds wander(Gonzalez-Castillo et al., 2021). Such a definition was deemed to allow a greater comparability between the two conditions of interest in the present study (i.e., dream and mind-wandering reports) (Christoff et al., 2016). Nonetheless, there may plausibly be considerable differences between an unintentional and intentionally initiated form of mind-wandering (e.g., differences in attentional and executive functions and affective content(Boulakis et al., 2023; Seli et al., 2016)).

Although we find that the present method is sensitive to individual differences, effect sizes were small and we could not exhaustively elucidate what might be driving such differences. For example, differences in aspects of executive function (e.g., inhibition vs verbal fluency), ethnicity, and socioeconomic status may have influenced the structure of dream and mind-wandering reports.

> Whilst the large sample size is a strength, this may have allowed to find small effects that would not be found with smaller sample sizes (e.g., PC2, Clustering coefficient and word count in figure 2, individual differences analysis). Furthermore, although the participants were instructed to find an environment free from distractions, we did not employ post-hoc measures evaluating distractions. Such measures may be a consideration for future studies.
>
> The use of sliding window may have highlighted different words in each window, and future research may also look for salient words after having removed “stop words” (i.e., commonly found, semantically unimportant words e.g., “and”) and explore whether other variables may explain sentence word repetition (e.g., education, sentence length).

In the individual differences subsection of the discussion, we also cite various possible drivers of individual differences and have added ethnicity:

> However, an aspect to consider is the extent to which part of the individual specific variation captured by this approach is driven by writing style, vocabulary, education, ethnicity, social economic status, engagement, and other such variables (Mota et al., 2023). Although, we do include some of these relevant variables in statistical models, future research may try to elucidate how other affective, trait and demographic factors may differentially relate to the structure of naturalistic texts.

Line 518. My understanding is that anything that is not included in the pre-registered analysis needs to be made clear. Can authors please ensure that? I recognize the analysis plan was written in a more of a high-level way, but some decisions (for example to include individual graph theory measures in addition to the principal components seems to be a post hoc decision). I think it would be appropriate to explicitly state which aspects were pre-registered (which is a valuable feature of this manuscript) and which aspects were exploratory/post-hoc.

We thank the reviewer for their comment and agree with them. The pre-registration was written in a high level way. We did specify that there would likely be an inherent problem of multicollinearity. However, the specific extent of co-variation was impossible to predict with accuracy before analysing the data. Hence, we also specified the metrics of interest:

> The graph theory measures of interest will be maximum and average centrality, number and strength of connected components, network density, largest connected component, modularity, verbosity (before sliding window), path length, participation coefficient, transitivity/clustering, assortativity (and other such hierarchical measures, flow hierarchy, rich club), number of loops, betweenness centrality. These measures will be obtained from the python “networkX” package (https://networkx.org/)

We also make suggestions as to how the correlations may play out. Although we did imagine that word recurrence would be of primary importance, we did not predict it would explain

most other graph theory metrics included (hence the high explained variance of PC1). As to the inclusion of both individual graph theory metrics and reduced variables, we have included the following:

> We reduced the data via the above described principal component analysis to ensure that predictor variables had sufficient independent variance to avoid multicollinearity issues (NB: inclusion of both individual graph theory metrics and reduced graph theory metrics was not explicitly stated in the pre-registration).

Importantly we elaborate on the included graph theory metrics in supplementary material 1. Following the reviewers comment, we have now modified the relevant table to include which were post hoc and which were pre-registered. We refer the reader to this in the main text should they be interested.

> In the preregistration (https://osf.io/arx2h) we attributed interpretations to such measures (post hoc decisions are noted in supplementary material 1).

For H2, we did specify various ways in which the individual specific information could be tested.

1) Difference between graph theory metrics
2) Portrait divergence (Bagrow & Bollt, 2019)
3) Alignment of nodal scores between networks (e.g., MI-GRAAL in (Tantardini, Ieva, Tajoli, & Piccardi, 2019))
4) Graphlet (subgraph structures) correlation distances.
5)

We opted from the most straightforward version post hoc finding this method valid, simple and effective. Other methods may have not yielded valid results (e.g., can such sparse networks yield valid results with portrait divergence? How would the high level of explained variance in PC1 relate to alignment of nodal scores? etc.,), and we did not explore this further. In the main text we add

> As per preregistered hypotheses (https://osf.io/arx2h, first proposed method used), we tested whether the report structure contained individual specific information.

Although we explicitly state this for H1, for H3, we did not not predict that multi-level models would not be able to resolve intercepts, hence we adopted the conservative convergence approach above.

We have made this explicit in the methods section

> We performed mixed model analyses that permitted an ordinal outcome (not explicitly stated in the pre-registration).

Line 587. Based on my understanding of principal components as well as common practice, it is rare to see principal components that account for less than 5% of the data to be used for subsequent analysis. It's helpful to see the inclusion of PC2 accounts for significant unique variance based on model comparisons with the anova function.

But it would be difficult to justify testing it in the first place. Can authors provide justification for examining this component in the first place? If not, this would seem to be a post-hoc decision – I did not find information in the pre-registration about how to select the number of components. If the authors feel strongly about keeping, please include a rationale and supporting literature in the Methods and report the variation explained in the Results, as this stark contrast between the variance accounted for in PC1 and PC2 is important for interpretation. This also needs to be addressed as a limitation. Line 625.

We thank the reviewer for their comment. We did adopt a bottom up approach and did not know how data reduction would play out. We were not sure whether all graph theory metrics could be reduced to a component, whether several useful components would arise, or even whether several graph theory properties would have relevant explained variance. Interestingly, all of these scenarios turned out to be true to some extent. However, the reviewer is correct in that we could have been more insightful in predicting the various scenarios and explicitly preregistering different processes for each of these.

As above, we would like to note that there are different questions involved in this analysis.

1) What is the shared variance between graph theory properties and how can we reduce highly collinearity for useful summary metrics

2) What graph theory properties predict differences between dream and mind wandering reports

3) What graph theory properties correlate with self reported measures.

Whilst these questions are inter-dependent (specifically, 2 and 3 depend on 1), they are different and may plausibly have different answers(Sclove, 2021).

Beyond the notion that PC2 statistically was valuable for the question as to how mind wandering and dream reports differ, it could be argued that the removal of PC2 would be a shortcoming. The high explained variance of the first component is due to the high number of highly correlated variables inserted. If fewer were inserted then the explained variance would have dropped. Similarly, If we excluded all the highly correlated variables then the first PCs would have had far less explained variance. Including all graph theory variables may have been a viable alternative, but at the cost of interpretability, as the present method permitted us to retain some of the “original” unmodified graph theory metrics, which had sufficient independent variance to be used in the analysis. Furthermore, of note, there are a substantial number of graph theory components. The selection and inclusion of any subset of these is an open question. Many of these variables would be highly correlated, and the final explained variance of the PCs would depend on such a-priori and somewhat arbitrary decisions.

Nonetheless, we agree with the reviewer that predicting and explicating this and the lack of specification at the preregistration level is important and desirable. This has been a valuable experience for us.

We have added the following phrases to make this absence explicit.

In fact, the Akaike information criterion was 3716.2 for the model without PC2 and 3700.9 for the model with PC2 (note the procedure for including variables of interest was not specificed in the preregistration).

We have added the following changes in the results section with information regarding the explained variance as well as an indication as to the fact that data reduction and differences in report structure are different questions.

> The first component primarily captured network density/centrality and explained 91% of the variance of the highly correlated variables included in the PCA, whilst the second component reflected network diameter/mean betweenness centrality (capturing 3.9% of the variance, Fig. 1E). Using the Akaike Information Criterion, these reduced components were subsequently used to answer the separate question as to how dream and mind wandering reports differ without violating statistical assumptions(Sclove, 2021).

Nonetheless, it is true that the effects of PC2 were small. As per a comment above, in the limitations section there is also an intimation that the large sample size may have contributed to the second PC showing significant effects.

> Whilst the large sample size is a strength, this may have allowed to find small effects that would not be found with smaller sample sizes (e.g., PC2, Clustering coefficient and word count in figure 2).

MINOR

Figure 2. Can authors plot out assortativity as well, despite the variable was non-significant?

Please see below the new version of figure 2.

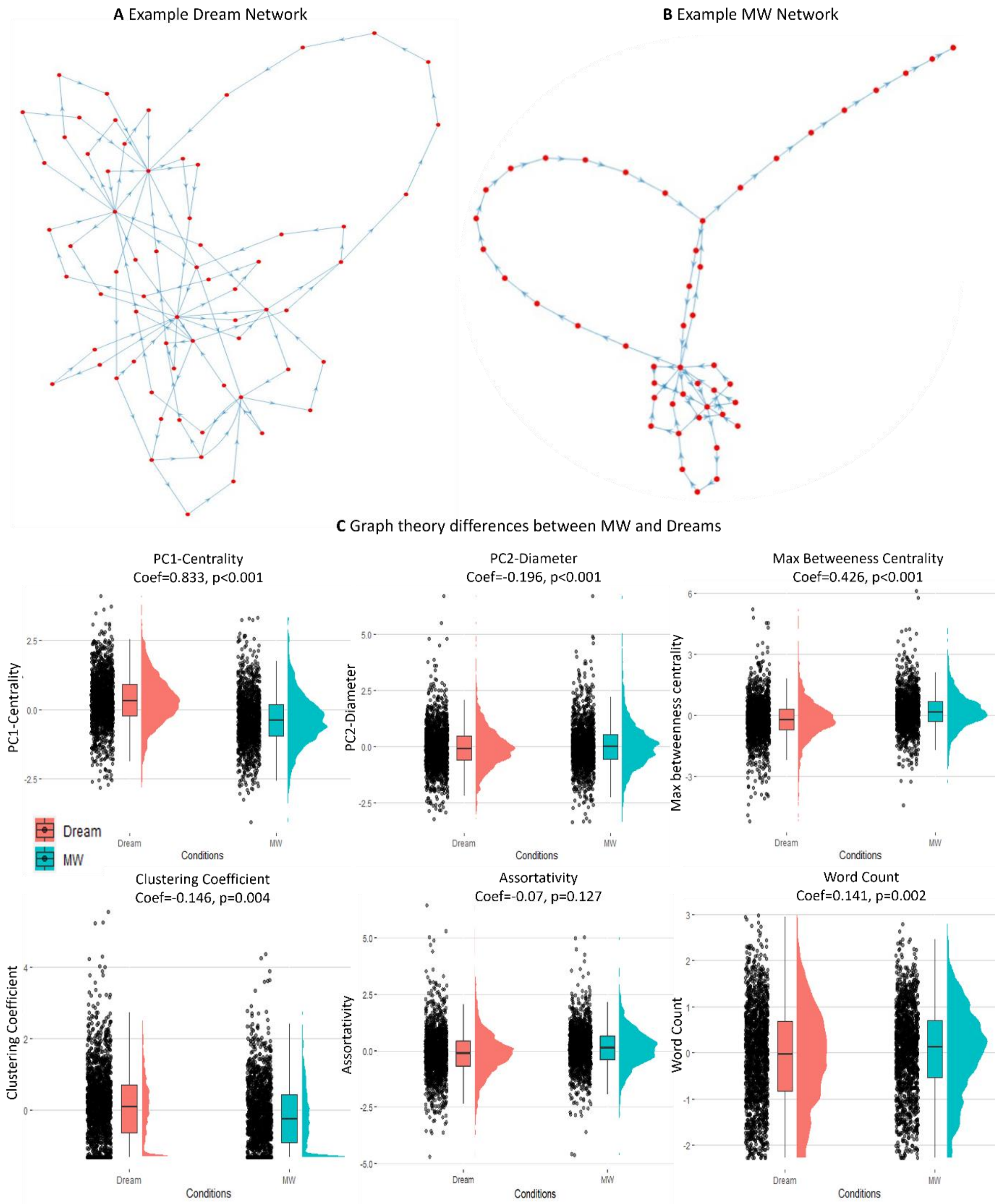

Line 506. The authors state that a participant was excluded if they did not have at least three entries per type of report. Then they report that 1781 dream reports were recorded from 172 participants and 1496 mind wandering reports were recorded from 152 participants. So how is it possible to have a final sample of 176 participants (given that participants must have at least 3 entries of dreaming and mind wandering reports)?

We thank the reviewer for having pointed this out. This was a regrettable oversight on the first author's part. Whilst it is true that participants needed to have three entries for a previous study, this was not the case for this study. We have now amended the methods section.

We ensured that statistical assumptions were not violated using the R package DHARMA as explicated in the methods section.

> We used the DHARMa to test assumptions and found these were not violated (when removing an outlier in PC1).

However, when encountering the issue in H3, whereby models were rank deficient, the removal of participants without repeated measures was one of the attempts to get a statistically sound model. Regrettably this was to no avail.

Line 550. Can authors explain what word sequences are, for those not familiar with this approach?

We thank the reviewer for pointing out the lack of clarity.

Please see below changes to the text with further explanation.

> Where, in the report, there were two or more instances of word sequences (i.e., in the relevant text as per sliding window, the same words were adjacent two or more times), the corresponding cell in the matrix (representing the graph theory network) was filled with the total instances of the sequence, thus creating a weighted network.

Line 560. Can authors clarify what is meant by "several networks" were obtained for each report?

We thank the reviewer for pointing out a lack of clarity. We have added the following information to ensure that the analysis is clear.

> We thus obtained several networks for each report by using the sliding window approach explained above. Accordingly, the number of sliding windows depended on the total number of words for each text and were subsequently averaged to ensure that the word count did not have undue influence on the graph theory metrics. These networks, as obtained via the sliding window methodology, were then transferred into python for graph theory analyses as the package NetworkX (https://networkx.org/) provides exhaustive and robust analyses.

Line 604. When authors stated they reduced the data, are they referring to implementing the principal component analysis? If so this needs to be clarified as it is unclear written as is.

Absolutely. Please see the clarification below.

We reduced the data via the above described principal component analysis to ensure that predictor variables had sufficient independent variance to avoid multicollinearity issues.

Line 617. Please clarify whether variables were scaled within subjects. I think that would be the most appropriate given the repeated measures nature of the data.

We thank the reviewer for their comment. We utilised the R function scale() for all the variables before hand as is standard practice in R to render the statistical output more easily interpretable and different variables comparable. If the variables were scaled in a participant specific manner, then the individual differences information would have been removed and H2 and H3 could not have been tested.

Reviewer #3 (Remarks to the Author):

This is an interesting piece of work, with a creative use of a graph-theoretic measure to look at how words are repeated. I think there's a lot of interesting material here.

However, I also think that there are major gaps in the analysis and presentation that would require at least major revisions and a change in presentation. There are some very basic problems (e.g., material in the abstract that directly contradicts material in the main paper, sentences that seem to contradict the ones just before) that mean that revisions would need to be significant and wholesale.

We thank the reviewer for their comments. They are very insightful, and we will endeavour to clarify any issue to improve the manuscript.

(1) The preregistration that the authors present is rather incoherent. In particular, they claim that "dream reports will be unstructured". However, they also claim that "dreams would have a more modular and hierarchical structure". This doesn't seem to make sense: if I write a paper (or a referee report) with distinct modules in a hierarchical arrangement, this seems to indicate a very structured presentation.

We thank the reviewer for their comment. This is indeed a penetrative point. The vagueness and incoherence is partially due to the impossibility of accurately predicting the amount of shared/independent variance that different graph theory properties may have had a priori, as well as the multidimensionality of networks and the variable interpretation different concepts may have. We concede that the statements may have been incoherent.

Highly complex network may have several properties that are dissociable. Thus plausibly, two networks could exist that has simultaneously have a strong hierarchical structure but a short and high diameter respectively. Similarly, a highly modular structure could be interpreted as being unstructured (different sporadic, unconnected, disorganised components, as was our a-priori prediction), or being highly organised (different “types” that can be clearly classified into different modules). Needless to say, these predictions were conjured prior to analysing the data and were high level.

In illustration of the difficulty of forming predictions apriori, we note that modularity was completely subsumed by the first principal component and thus any such prediction would turn out to be, irrelevant once the correlational structure of the data was empirically investigated.

It is regrettable that we were not insightful enough to be able to make more explicit predictions. We nonetheless take the reviewers point that the different high level predictions could have been better explained and defined and will endeavour to try better in the future.

In response to the reviewers comments below, we modify our interpretations in the main text (detailed below).

(2) It is very unclear if the authors have correctly translated the claim "modular and hierarchical" into graph theoretic language. They find, for example, that dreams have higher "density/centrality". I'm not sure what that means — "network density" is usually something like "edges per node-pair", i.e., (# edges)/(N*(N-1)/2).) So a higher density is just the amount of word repetition — they should find that word repetition and density covary (almost) perfectly. The fact that dream reports have more word repetition does not seem to indicate "lack of structure" or (conversely) "modularity and hierarchy".

The reviewer is absolutely correct. After analysis, it became clear that word repetition is a major source of variation in this type of graphs. The high covariation meant that we refered to them as “density/centrality”.

To make this explicit, in table 1 in describing principal component one (PC1), we put the words “How dense are the graph connections? Are there words with lots of repetitions?”. Of note PC1 subsumes other graph theory metrics such as mean betweenness centrality, strongest connected component, modularity etc., (we have now inserted this information in Table 1 in the main text. Supplementary material 1 and 2 give further details). Importantly, we inserted highly correlated variables for data reduction, preferring to retain “pure”, unadulterated graph theory variables in the statistical models where there was independent variance. The correlation matrix in figure 1D (main text) and figure S1 in the supplementary materials offer a good visual representation.

We nonetheless make this explicit in the main text

> Thus, repetition of words strongly drives density, number of connected components, node centrality, modularity, numbers of cycles etc.,

We also explicit state this notion in other parts:

> Specifically, dream reports were characterised by higher word repetition within the same report (i.e., node centrality, in graph theory terminology). This effect was captured by the first principal component and is an important feature of the data, explaining high amounts of variance (in principal component analysis of highly correlated variables) and displaying the strongest effects (Fig 1C & Fig 3D).

The modularity metric was instead subsumed into the first principal component and was driven by density/node number, as stated above. Although we did suggest that this would be a major source of variation in the data, we were not able to predict this level of detail a-priori.

As to hierarchy, we did investigate this via the flow.hierarchy NetworkX function (supplementary material 2) but gave consistent results of 0 in this type of network.

Arguably, assortativity can be considered a measure of hierarchy (the richer nodes, connect to richer nodes, and "poorer" ones to "poorer" ones, thus forming "tiers" in connectedness of nodes). This was revealed to be non significant.

The reviewer is right in pointing out that the interpretation in relation to our pre-registered high level hypotheses are not a straightforward as we might have suggested in the first version of the manuscript. Thus we have also removed the interpretation of the findings in terms of the pre-registered hypothesis. Although we do interpret the findings in terms of higher complexity in dream graphs (e.g., displaying hallmarks of a small world network), regrettably we did not define "complexity" explicitly in the preregistration and we recognise that the relationship between complexity and structure is not always straightforward (e.g., lack of structure may indicate unpredictability, thus higher Shannon entropy, yet a uniform distribution (i.e., high entropy) may be considered unstructured and not complex). We have **removed** the following phrases:

> ~~"Overall, the findings support our first hypothesis that dream reports are more complex than mind wandering reports."~~
>
> Thus, ~~"in consonance with our predictions",~~ mind-wandering reports had a simpler, more linear (i.e., more extended) structure. They were more verbose (had more words), with essential transition points (higher max between centrality) in the narrative flux (e.g., words connecting different parts of the report).

(3) There's a claim that PC1 is also "centrality"; it's unclear what this means, because centrality is usually a property of nodes, rather than edges. There's no definition of what they mean in the text. There's a great deal left out about the details of the analysis, and quite a bit of material is missing altogether (e.g., I'm referred to a MATLAB package for basic questions like what kinds of measures are being used.)

We thank the reviewer for pointing out the lack of clarity.

The reference to MATLAB refers to a script that is freely available and is used to create the matrices. To avoid confusion we have inserted the following:

> We created a MATLAB script that would reproduce the output of the speech graphs software that was used in previous research (Mota et al., 2014, 2022). To do this, all words were converted to lower case and punctuation (including apostrophes) was

removed. The script returns matrices that represent graphs/networks and is available at https://github.com/Peter6789/graph-theory-analysis-of-text/tree/main.

This is to enable anyone to create these types of networks.

In fact, we use the python toolbox “networkX” which is more exhaustive than the toolbox MATLAB provides. We state this in the following:

> These networks, as obtained via the sliding window methodology, were then transferred into python for graph theory analyses as the package NetworkX (https://networkx.org/) provides exhaustive and robust analyses. Information with all relevant details of graph theory analyses can be viewed in supplementary material 1.

We intended to write this paper for a general audience rather than for experts in graph theory. Thus we do not enter into extreme details in terms of the mathematical technicalities, or an exhaustive description of all the measures used when these are not required to understand the results (e.g., due to data reduction/ high collinearity) and would occupy various pages. However, we offer all information necessary for reproduction (e.g., functions and parameters) in supplementary material 1. Any reader may use the information in S1 to refer to the freely available original toolbox documentation. This will allow the reproduction of any analyses. Of note is that centrality is a measure of how important nodes are. There are various ways to measure centrality, although here we utilize the degree centrality measure, as being the most common and straight forward.

As per the reviewer’s suggestion, we now specify this in the methods in various places, for example:

> Mean degree centrality and density had the highest loadings indicating the repetition of words was the main contributor to this principal component.

In the spirit of giving a strong intuitive understanding to a wide readership we try to explain what we are measuring in a variety of ways (including principal component 1).

We do so visually in figure 1E with practical examples.

In simple language in table 1 which is copied below

Metrics	Description
PC1-Centrality	How dense are the graph connections? Are there words with lots of repetitions? This aspect of networks explains graph theory measures of modularity, strongest connected component, mean betweenness centrality (see S2).
PC2-Diameter	How far away are the two extremes of the graph? Does it take many “steps” to get from one side of the graph to another?

Clustering Coefficient	Are there cliques in the report? The extent to which neighbours of one node are also neighbours amongst themselves.
Maximum betweenness centrality	Are the points in the network through which lots of paths tend to go through? Are there essential “bridges” in the report?
Assortativity	Are highly connected nodes also connected to highly connected nodes? Are minimally connected nodes connected to minimally connected nodes?
report length/verbosity	How many words are in the report? What would be the total number of edges if the sliding window approach was not used?

As well as throughout the text. For example

> A mixed-effect logistic regression showed that lower density/centrality (i.e., less repetition of words; coef.= 0.83, p<0.001),
>
> Both trait and state anxiety have been related to dream recall frequency (Rimsh & Pietrowsky, 2020), which likely correlates with higher word repetition (i.e., node centrality).
>
> Across various analyses, we report the graph theory measure that was particularly stable. Specifically, the centrality of dream reports (i.e., word repetition)
>
> Specifically, dream reports were characterised by higher word repetition within the same report (i.e., node centrality, in graph theory terminology).

As is shown in figure 1D, Density and centrality have a very high correlation, as the reviewer appropriately notes above. In fact, these measures load most highly onto PC1. This is shown in SI 2 in case any reader wanted to inspect these details. Thus within PC1, density and centrality (driven by word repetition) are summarised as they are highly correlated (r>0.9).

We have changed the following to make the text clearer from word centrality to word repetition

… and explore whether other variables may explain sentence word repetition (e.g., education, sentence length).

In the following we write

Thus, repetition of words strongly drives density, number of connected components, node centrality, modularity, numbers of cycles etc.,

The specific functions are fully reported in the supplementary materials, the script to produce the graphs is shared with the audience, and the main text tries to give an intuitive understanding of the measures to a general audience.

(4) It's also claimed that diameter is smaller in the dream graphs; it's not clear if this is max diameter, or average diameter (again, we don't have details). It's unclear if "unstructured" implies "small diameter" — indeed, you generally need quite a bit of structure to make diameter small; it's also unclear if "small diameter" implies "modular" (in general, if you have modules, the diameter will get larger).

We thank the reviewer for their comment. The diameter refers to the maximum path length. Average diameter could be obtained, if the maximum path length was averaged over several networks (although in as sense this is what we do as per the sliding window algorithm).

Although we do not explain path length and computational details, again, we try to give and intuitive definition in figure 1E in table 1D

PC2-Diameter	How far away are the two extremes of the graph? Does it take many “steps” to get from one side of the graph to another?

As well as throughout the text, as for example.

higher diameter (more difficulty to get from one side of the text to the other (coef.= -0.19, p<0.001),

SI1 enables to find the specific algorithm used and any additional preprocessing steps so as to enable to find the formal mathematical definition and reproduction with the appropriate function.

The reviewer makes an interesting point in relation to the modularity and its relationship with path length. Empirically we found that modularity is highly colinear with density and centrality (hence these are all summarised by PC1) and thus we cannot investigate the claim in an appropriate manner. Suffice it to say that modularity and diameter were sufficiently independent as to be described by different principal components, but not so much as to not warrant the use of data reduction. It may very well be the case that the answer changes with

different types of networks and even samples of different types of networks (e.g., friendly vs professional emails, brain white matter etc.,). We now realise that there is an issue with the lack of precision in the term “structure”. As above, following the suggestion of the reviewer, in the latest version of the manuscript we have removed notions as to interpreting results in these terms of a lack of structure.

(5) It would be good to show coefficients on the PCs, and how they decompose into the different basic measures.

Absolutely, we copy below the information in supplementary material 2.

Figure S1- Correlational structure of graph theory variables.

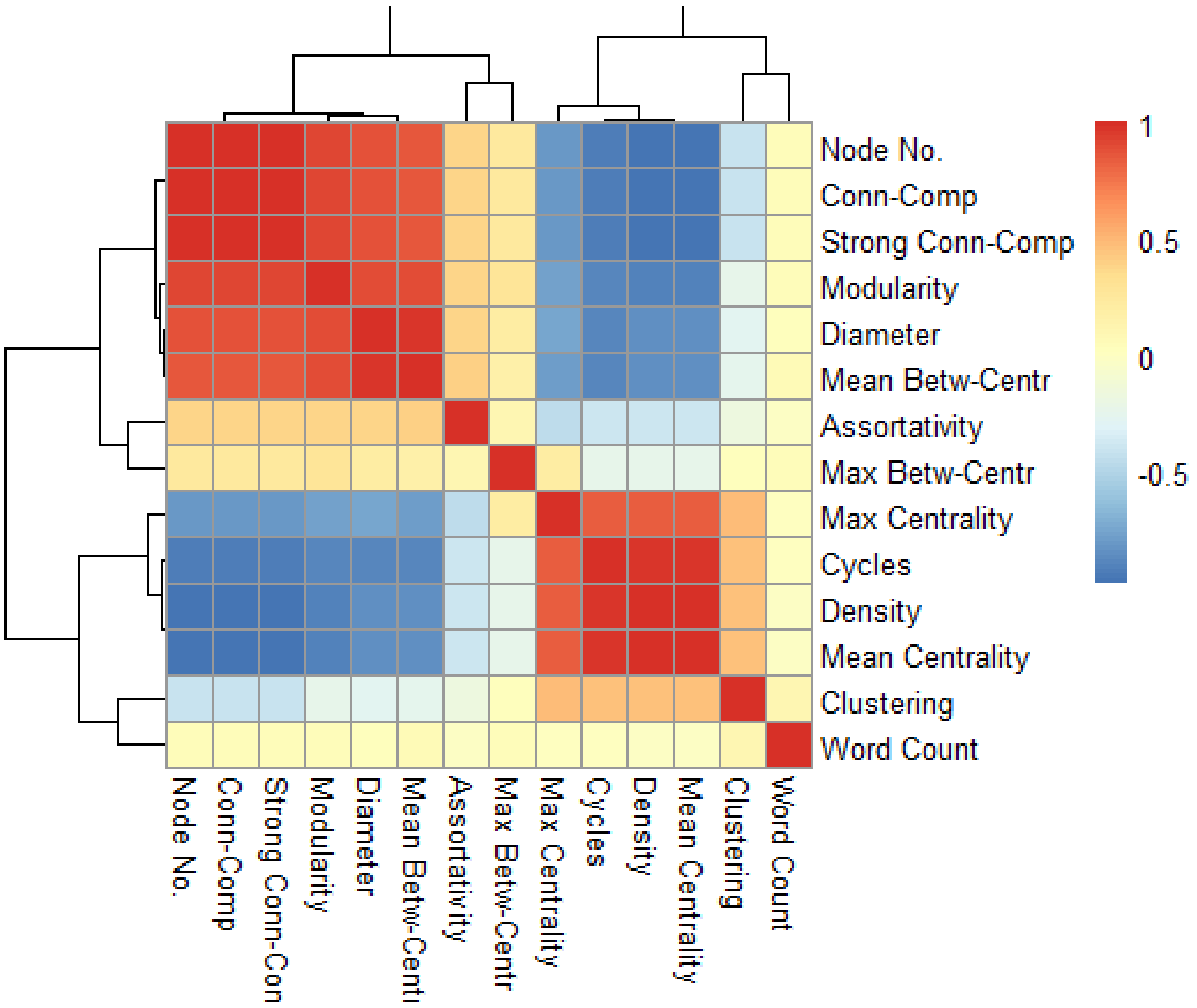

Table S2. Variable inserted in the PCA with loadings onto the principal components (sign also displayed). Highlighted in BOLD are the highest loadings onto PC1 and PC2

	PC1	PC2
Diameter	0.306	**-0.546**
Density	**-0.326**	-0.239

Node Number	0.324	0.264
Mean centrality	**-0.326**	-0.239
Maximum centrality	-0.285	0.045
Modularity	0.313	-0.114
Cycles	-0.320	0.045
Largest Connected component	0.324	0.264
Largest strongly connected component	0.324	0.264
Mean Betweeness centrality	0.305	**-0.600**

Of the variables we chose to investigate, only the most highly correlated were inserted in the PCA, as the object of this was to reduce the highly correlated data into components that had sufficient independent variance to be included as covariates in a statistical model.

Percentage explained variance for PC1 was 90.98. For PC2 it was 3.94 whilst for PC3 it was 2.88

As discussed in the main text, all variables loaded more or less equally onto PC1. However, we note that a foundational source of variance for this type of network is word repetition, and thus we decide to interpret this primarily in terms of word-repetition, as noted by the reviewer above. We make this explicit in the
In PC2, mean betweenness centrality has the highest loading whilst diameter is the second. This is due to the fact that both functions rely on calculation of shortest paths. We decided to interpret this primarily in terms of diameter as this is more intuitive and avoids confusion with another metric in the paper which is maximum betweenness centrality.

(6) There seems to be an error in either the table or the text — it's claimed that dream reports have more clustering, but the coefficient is negative. (The negative coefficient for Diameter implies that mind wandering has larger diameter, so why doesn't a negative coefficient for Clustering imply mind wandering has larger clustering?)

We thank the reviewer for their comment and their attention to detail.

They are absolutely right. However, as can be seen above, the diameter is loaded negative onto the principal component, thus the interpretation needs to be reversed for this (as well as, in fact, PC1). We inverted this on the figures to make the interpretation more straight forward. This is shown on the loading copied above and displayed in SI 2. However, we thank the reviewer for pointing out the difficulty.

We have added the following to the figure caption to avoid confusion.

The values for PC-1 and PC-2 are inverted for ease of interpretation (e.g., density and diameter had negative loadings onto PC1 and 2 respectively)

We noted that the negative sign was not included in the figure. We have added it in the latest version.

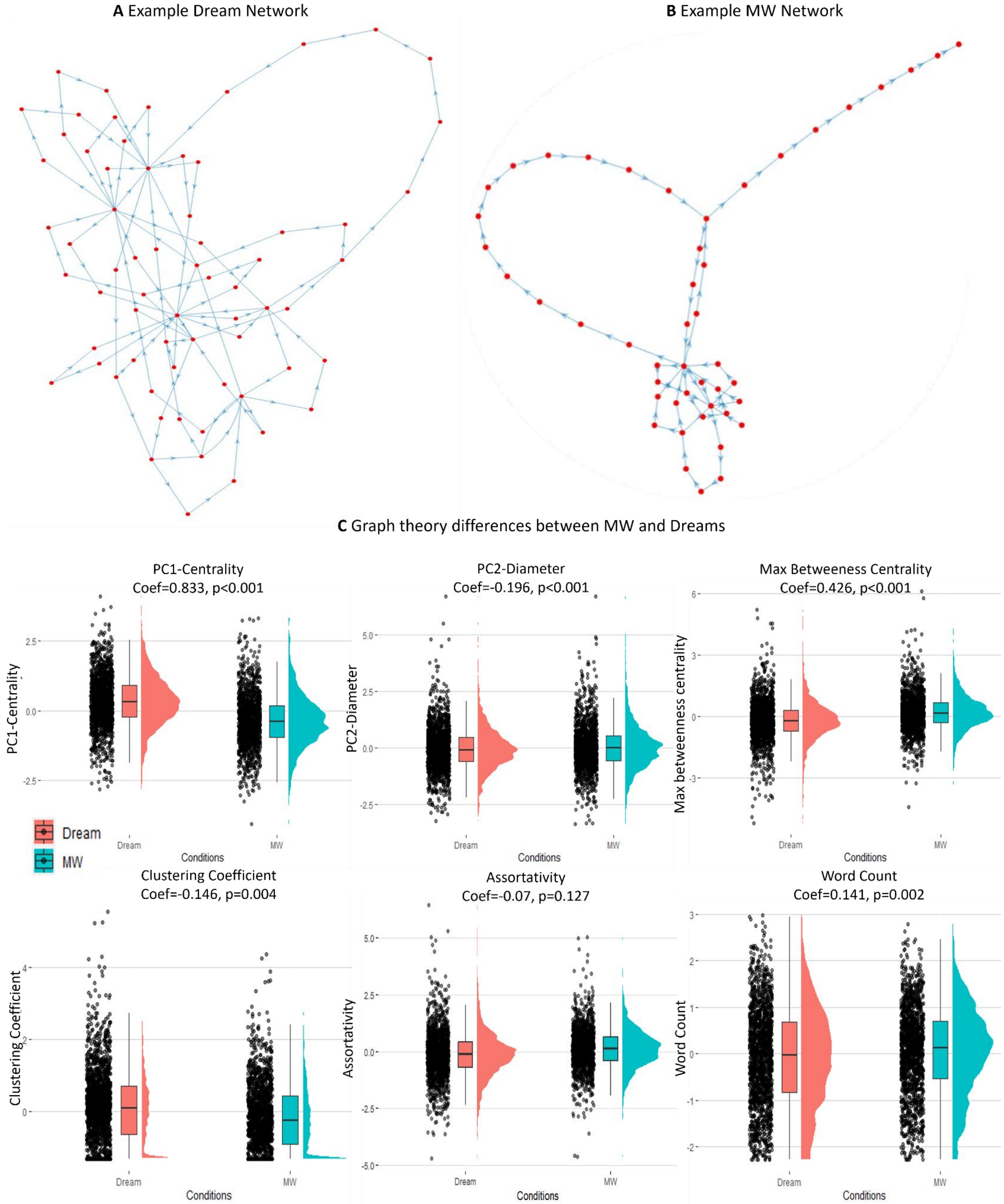

We have inserted the following phrase in the methods section to ensure that this is clear.

> Loadings of the relevant variables were negative onto PC1 and PC2. In the main text figures values have inverted these for ease of interpretation.

(7) There are some strange claims that I don't quite understand — e.g., the claim that mindwandering has "essential nodal points". What does this mean? How is this determined by graph metrics? (I can think of ways it might be done, but I'm left to guess, and I'm left without any significance or effect size for this claim).

We thank the reviewer for making the lack of clarity apparent.

This refers to the maximum betweenness centrality. (i.e., nodes through which a lot of short paths go through). We phrased this result in such a manner in an attempt to make the results understandable to readers without graph theory expertise but the reviewer has made us realise that the opposite of the desired effect may be induced if the reader has relevant technical knowledge.

We have thus inserted the measure name when we make this claim in the main text. We have also changed nodal points to transition points as this was deemed to be more interpretable/understandable.

> Thus, in consonance with our predictions, mind-wandering reports had a simpler, more linear structure. They were more verbose (had more words), with essential transition points (higher max betweeness centrality) in the narrative flux (e.g., words connecting different parts of the report).
>
> Whilst dream reports were more recursive and complex, mind-wandering reports had a more linear, extended structure with essential transition points in the report structure (higher max betweenness centrality).

(8) I'm unclear why a larger diameter means a more "linear" narrative. Here are two narratives:
(A) I went to the store. At the store, I bought some oranges. I paid the cashier, who complimented me, and I left the store.
(B) I went to the store. A giant giraffe attacked me. Then some monkeys came out. My spaceship landed safely.

It seems clear that (A) is a very linear narrative, and (B) very random and non-linear. But (B) has a much larger diameter than (A), if I understand the method correctly — (A) repeats "I" and "store" a lot, but (B) has no repetitions. If by "linear" they mean something more specific, it needs to be made clear what this is.

The reviewer is absolutely right in that our method does not explicitly target narrative content, but rather more the structure of the text, where word repetition is a primary factor.

Throughout the text we refer to a linear “structure”. Please see below examples of this. To increase clarity, we specify that we mean a more extended structure (as per the graph theory methodology employed).

Following the reviewers appropriate comment, we have modified the places where we cite “narrative”, as this is more accurate.

Thus, in consonance with our predictions, mind-wandering reports had a simpler, more linear (i.e., more extended) structure

Whilst dream reports were more recursive and complex, mind-wandering reports had a more linear, extended structure with essential transition points in the report structure (higher max betweenness centrality).

These are essential connector points in the more linear and extended structure that characterised mind-wandering reports.

This is in consonance with the fact that mind-wandering reports have higher diameter, indicating that the report is less integrated (interconnected over long distances via specific words) and is related to the more linear (rather than recursive), extensive, and less integrated structure.

They were more verbose (had more words), with essential transition points in the structure of the texts (e.g., words connecting different parts of the report).

We find that dream reports have a more complex structure, while mind-wandering reports have fewer word repetitions and more verbose structure, with essential transition points in the structure of the texts.

(9) The results on mental health and texts seem so vague as to be basically null results. I couldn't understand, for example, why they couldn't do very simple logistic regressions, in the same way they did for the graph properties. This section needs to be either cut, or completely redone.

We thank the reviewer for their comment and pointing out our lack of clairty. We did in fact use logistic regression, however, we had to forgoe the use of repeated measures.

As explained above in this document, we ran various versions of mixed logistic regressions; however these could not estimate intercepts correctly. The most common error across models was that it was rank deficient. However, across many different permutations of the analyses, PC1 showed convergent effects. We decided to be highly conservative in our reporting of this result, (we report results from averaged data accross repeated measures). However we now realise that this has caused more confusion rather than confidence. If the reviewer were interested, various versions of this analysis are presented in S4. A reader inspecting this would notice that in fact, there are likely to be more than one graph theory measures that have significant effects in predicting self-reported mental health scales. Regrettably none of these analyses, that attempted to capitalise on the repeated measures design of the study, showed unviolated assumptions. Consultations with biostatisticians and mathematicians did not yield a satisfactory solution. Thus, we resolved this by showing convergence with robust methods that however significantly reduced our statistical power. (namely non parametric correlation and averaging all repeated measures and running an ordinal logistic regression).

Please see the changes to the text below that better reflect our conservative convergence approach and our confidence in the effects.

> We subsequently explored how the architecture of dream and mind-wandering reports predicts mental health symptoms, specifically, self-reported measures of anxiety (GAD-7) and depression (PHQ-9). Attempts to fit multilevel mixed models did not yield trustworthy intercept estimations, likely due to having one self-reported mental health measure per participant (176) rather than per report (3053). Although mixed models showed several significant effects (SI 4), here we decide to adopt a highly conservative approach.
>
> We averaged across repeated measures to yield one value per individual and conducted analyses for dream and mind-wandering reports separately. We adopted a convergence strategy, and here report results showed consistency across several methodologies (see Methods and supplementary material 4), thus suggesting robust findings. Presented are results using ordinal logistic regressions and non parametric correlations. Alternatives are available in supplementary material 4.
>
> Across various analyses, we report the graph theory measure that was particularly stable. Specifically, the centrality of dream reports (i.e., word repetition) was likely to be associated with a higher PHQ-9 score (in ordinal logistic regression coef.=-0.47, [95% CI= -0.84, -0.10], p=0.013, Odds Ratio = 0.52; Kendall's tau 0.14, p=0.005; see Figure 3D). There was another reasonably stable trend that emerged across analyses (see supplementary material 4). The centrality of mind-wandering reports showed a relationship with the GAD-7 score which was non significant using Kendall's tau (in ordinal logistic regression coef.=-0.48, [95% CI= -0.94, -0.02], p=0.038, Odds Ratio = 0.62; Kendall's tau=0.10, p=0.06).

(10) The result that "people are more similar to themselves than to others" doesn't seem to make sense to me. How could the opposite be true?

That the opposite could be true would be relatively unlikely (e.g., accross contexts). However, the notion that "people are statistically no more similar to themselves than to others" would indicate that the method employed is marred by so much noise or randomness that it is not sensitive to individual differences.

This was a hypothesis that intended to find whether this method is indeed sensitive to individual differences.

(11) I don't see how the results claimed in the abstract are supported by the analysis. For example: thematic repetition in dreams? But how do we know it's thematic repetition, rather than just repeating function words like "I"? Above I've mentioned other issues: the claim that dream reports are "less structured" (this is H1, that they claim is consistent with their data) seems contrary to the claim in the abstract that dream reports have "more complex structure".

We thank the reviewer for their comment.

They are absolutely right that we did not find thematic repetitions. We agree with the reviewer that this is an overstatement. We have thus changed the abstract in accordance with the reviewer's suggestions

> Dream reports tend to have more word repetitions, local cliques, and global integration.

Again, as above, the relationship between complexity and structure is very interesting and complex, and may depend on the specific definitions and operationalisations employed (e.g., high structure might indicate high predictability, and thus low complexity, or moreover, a highly detailed dynamic structure may be defined as complex). Nonetheless, we realise that this needed to be better defined a priori. As stated above we have removed statements that the data supports hypothesis one in relation to this aspect.

Summary: I don't want to seem too negative here. The basic story is that the authors came up with a cool method, and are clearly getting distinctions between the dreams and mind-wandering. There is great data, and the basic methods are strong. But there are big gaps: in both the framing of the analysis, the operationalization of the concepts, the construct validity of the claims, and the analysis of the the mental health material. I think that something approaching a complete re-write would be necessary here to fix things.

We thank the reviewer for their comments. We have tried to amend the various issues they pointed out and we believe that the manuscript has been substantially improved as a result.

References

Barahona, M., Barahona, M., & Pecora, L. M. (2002). Synchronization in small-world systems. *Physical Review Letters*, *89*(5), 054101/1-054101/4. https://doi.org/10.1103/PhysRevLett.89.054101

Block, N. (2019). What Is Wrong with the No-Report Paradigm and How to Fix It. *Trends in Cognitive Sciences*, *23*(12), 1003–1013. https://doi.org/10.1016/j.tics.2019.10.001

Boulakis, P. A., Mortaheb, S., van Calster, L., Majerus, S., & Demertzi, A. (2023). Whole-Brain Deactivations Precede Uninduced Mind-Blanking Reports. *Journal of Neuroscience*, *43*(40), 6807–6815. https://doi.org/10.1523/JNEUROSCI.0696-23.2023

Carhart-Harris, R. L., & Friston, K. J. (2010). The default-mode, ego-functions and free-energy: A neurobiological account of Freudian ideas. *Brain*, *133*(4), 1265–1283. https://doi.org/10.1093/brain/awq010

Carhart-Harris, R. L., Leech, R., Hellyer, P. J., Shanahan, M., Feilding, A., Tagliazucchi, E., Chialvo, D. R., & Nutt, D. (2014). The entropic brain: A theory of conscious states informed by neuroimaging research with psychedelic drugs. *Frontiers in Human Neuroscience*, *8*(1 FEB), 1–22. https://doi.org/10.3389/fnhum.2014.00020

Chaves, M. F., Rodrigues, C., Ribeiro, S., Mota, N. B., & Copelli, M. (2023). Grammatical impairment in schizophrenia: An exploratory study of the pronominal and sentential domains. *PLoS ONE*, *18*(9 September). https://doi.org/10.1371/journal.pone.0291446

Christoff, K., Irving, Z. C., Fox, K., Spreng, R. N., & Andrews-Hanna, Jessica, R. (2016). Mind-wandering as spontaneous thought: a dynamic framework. *Nature Reviews Neuroscience*, *17*(11), 1–44. https://doi.org/10.1038/nrn.2016.113

Domhoff, G. W., & Fox, K. C. R. (2015). Dreaming and the default network: A review, synthesis, and counterintuitive research proposal. *Consciousness and Cognition*, *33*, 342–353. https://doi.org/10.1016/j.concog.2015.01.019

Ermer, M., & Alférez, G. H. (n.d.). *Visualizing literary narratives with a graph-centered approach.*

Finger, I., Lemke, C. E., Cury, L. da S., Mota, N. B., & Weissheimer, J. (2025). When kids juggle it all: Biliteracy instruction and the development of discourse connectedness in L1 and L2 writing. *Cognitive Development*, *73*, 101535. https://doi.org/10.1016/j.cogdev.2024.101535

Fox, K. C. R., Nijeboer, S., Solomonova, E., Domhoff, G. W., & Christoff, K. (2013). Dreaming as mind wandering: Evidence from functional neuroimaging and first-person content reports. *Frontiers in Human Neuroscience*, *7*(JUL), 1–18. https://doi.org/10.3389/fnhum.2013.00412

Franklin, M. S., Mrazek, M. D., Anderson, C. L., Smallwood, J., Kingstone, A., & Schooler, J. W. (2013). The silver lining of a mind in the clouds: Interesting musings are associated with positive mood while mind-wandering. *Frontiers in Psychology*, *4*(AUG). https://doi.org/10.3389/fpsyg.2013.00583

Friston, K. (2010). The free-energy principle: a unified brain theory? *Nature Reviews. Neuroscience*, *11*(2), 127–138. https://doi.org/10.1038/nrn2787

Gomes, M., Picó Pérez, M., Castro, I., Moreira, P., Ribeiro, S., Mota, N. B., & Morgado, P. (2023). Speech graph analysis in obsessive-compulsive disorder: The relevance of dream reports. *Journal of Psychiatric Research*, *161*(March), 358–363. https://doi.org/10.1016/j.jpsychires.2023.03.035

Gonzalez-Castillo, J., Kam, J. W. Y., Hoy, C. W., & Bandettini, P. A. (2021). How to interpret resting-state fMRI: Ask your participants. In *Journal of Neuroscience* (Vol. 41, Issue 6, pp. 1130–1141). Society for Neuroscience. https://doi.org/10.1523/JNEUROSCI.1786-20.2020

James, W. (1890). *The Principles of Psychology*. 4004.

Kahn, D. (2013). Brain basis of self: Self-organization and lessons from dreaming. *Frontiers in Psychology*, *4*(JUL), 1–11. https://doi.org/10.3389/fpsyg.2013.00408

Kam, J. W. Y., Wong, A. Y., Thiemann, R. F., Hasan, F., Andrews-Hanna, J. R., & Mills, C. (2024). On the relationship between unprompted thought and affective well-being: A systematic review and meta-analysis. *Psychological Bulletin*, *150*(5), 621–641. https://doi.org/10.1037/bul0000428

Kirberg, M. (2022). Neurocognitive dynamics of spontaneous offline simulations: Re-conceptualizing (dream)bizarreness. *Philosophical Psychology*, *35*(7), 1072–1101. https://doi.org/10.1080/09515089.2022.2042231

Kroenke, K., Spitzer, R. L., & Williams, J. B. W. (2001). The PHQ-9: Validity of a brief depression severity measure. *Journal of General Internal Medicine*, *16*(9), 606–613. https://doi.org/10.1046/j.1525-1497.2001.016009606.x

Laureys, S. (2005). The neural correlate of (un)awareness: lessons from the vegetative state. *TiCS*, *9*. www.sciencedirect.com

Liu, C., Zhang, X., Nguyen, T. T., Liu, J., Wu, T., Lee, E., & Tu, X. M. (2022). Partial least squares regression and principal component analysis: Similarity and differences between two popular variable reduction approaches. In *General Psychiatry* (Vol. 35, Issue 1). BMJ Publishing Group. https://doi.org/10.1136/gpsych-2021-100662

Malcorra, B., Ribeiro, M., Jensen, L., Gomes, G., Meletti, T., & Mota, N. B. (2024). Speech connectedness predicts reading performance three months in advance: a longitudinal experiment. *Npj Science of Learning*, *9*(1), 35. https://doi.org/10.1038/s41539-024-00248-4

Martin, J. M., Andriano, D. W., Mota, N. B., Mota-Rolim, S. A., Araujo, J. F., Solms, M., & Ribeiro, S. (2020). Structural differences between REM and non- REM dream reports assessed by graph analysis. *PLoS ONE*, *15*(7), 1–20. https://doi.org/10.1371/journal.pone.0228903

Milgram, S. (1967). The Small World Problem. *Psychology Today*, *1*(1), 61–67.

Mota, N. B., Callipo, R., Leite, L., Torres, A. R., Weissheimer, J., Bunge, S. A., Copelli, M., & Ribeiro, S. (2020). Verbal Short-Term Memory Underlies Typical Development of “Thought Organization” Measured as Speech Connectedness. *Mind, Brain, and Education*, *14*(1), 51–60. https://doi.org/10.1111/mbe.12208

Mota, N. B., Furtado, R., Maia, P. P. C., Copelli, M., & Ribeiro, S. (2014). Graph analysis of dream reports is especially informative about psychosis. *Scientific Reports*, *4*, 1–7. https://doi.org/10.1038/srep03691

Mota, N. B., Vasconcelos, N. A. P., Lemos, N., Pieretti, A. C., Kinouchi, O., Cecchi, G. A., Copelli, M., & Ribeiro, S. (2012). Speech graphs provide a quantitative measure of thought disorder in psychosis. *PLoS ONE*, *7*(4), 1–9. https://doi.org/10.1371/journal.pone.0034928

Mota, N. B., Weissheimer, J., Finger, I., Ribeiro, M., Malcorra, B., & Hübner, L. (2023). Speech as a Graph: Developmental Perspectives on the Organization of Spoken Language. *Biological Psychiatry: Cognitive Neuroscience and Neuroimaging*, *8*(10), 985–993. https://doi.org/10.1016/j.bpsc.2023.04.004

Murray, S., & Krasich, K. (2022). Can the mind wander intentionally? *Mind and Language*, *37*(3), 432–443. https://doi.org/10.1111/mila.12332

Noreika, V., Valli, K., Markkula, J., Seppälä, K., & Revonsuo, A. (2010). Dream bizarreness and waking thought in schizophrenia. *Psychiatry Research*, *178*(3), 562–564. https://doi.org/10.1016/j.psychres.2010.04.034

Occhionero, M., & Cicogna, P. (2016). Phenomenal consciousness in dreams and in mind wandering. *Philosophical Psychology*, *29*(7), 958–966. https://doi.org/10.1080/09515089.2016.1213800

P. Vatcheva, K., & Lee, M. (2016). Multicollinearity in Regression Analyses Conducted in Epidemiologic Studies. *Epidemiology: Open Access*, *06*(02). https://doi.org/10.4172/2161-1165.1000227

Rosen, M. G. (2018). How bizarre? A pluralist approach to dream content. *Consciousness and Cognition*, *62*(January), 148–162. https://doi.org/10.1016/j.concog.2018.03.009

Sanz, C., Pallavicini, C., Carrillo, F., Zamberlan, F., Sigman, M., Mota, N., Copelli, M., Ribeiro, S., Nutt, D., Carhart-Harris, R., & Tagliazucchi, E. (2021). The entropic tongue:

Disorganization of natural language under LSD. *Consciousness and Cognition*, *87*(April 2020), 103070. https://doi.org/10.1016/j.concog.2020.103070

Sclove, S. L. (2021). Using Model Selection Criteria to Choose the Number of Principal Components. *Journal of Statistical Theory and Applications*, *20*(3), 450–461. https://doi.org/10.1007/s44199-021-00002-4

Seli, P., Risko, E. F., Smilek, D., & Schacter, D. L. (2016). Mind-Wandering With and Without Intention. *Trends in Cognitive Sciences*, *20*(8), 605–617. https://doi.org/10.1016/j.tics.2016.05.010

Seli, P., Smallwood, J., Schacter, D. L., Maillet, D., Schooler, J. W., & Smilek, D. (2018). Mind-Wandering As a Natural Kind. *Trends in Cognitive Sciences*, *22*(6), 479–490. https://doi.org/10.1016/j.tics.2018.03.010.Mind-wandering

Sikka, P., Tuominen, J., Ezquerro Nassar, A., Kirberg, M., Loukola, V., Revonsuo, A., Valli, K., Windt, J., Bekinschtein, T. A., & Noreika, V. (2023). COVID-19 on mind: Daily worry about the coronavirus is linked to negative affect experienced during mind-wandering and dreaming. *Emotion*. https://doi.org/10.1037/emo0001255

Smallwood, J., & Schooler, J. W. (2015). *The Science of Mind Wandering : Empirically Navigating the Stream of Consciousness*. https://doi.org/10.1146/annurev-psych-010814-015331

Spitzer, R. L., Kroenke, K., Williams, J. B. W., & Löwe, B. (2006). A brief measure for assessing generalized anxiety disorder: The GAD-7. *Archives of Internal Medicine*, *166*(10), 1092–1097. https://doi.org/10.1001/archinte.166.10.1092

Todd, N. (2014). Between subject and object: Using the grammar of verbs to enhance the therapeutic construction of personal agency. *Journal of Constructivist Psychology*, *27*(4), 289–302. https://doi.org/10.1080/10720537.2013.843479

Voss, U., Holzmann, R., Hobson, A., Paulus, W., Koppehele-Gossel, J., Klimke, A., & Nitsche, M. A. (2014). Induction of self awareness in dreams through frontal low current stimulation of gamma activity. *Nature Neuroscience*, *17*(6), 810–812. https://doi.org/10.1038/nn.3719

Watts, D., & Strogatz, S. (1998). Collective dynamics of networks. *Nature*, *393*(6684), 440–442.

Williams, J., Merritt, J., Rittenhouse, C., & Hobson, J. A. (1992). Bizarreness in dreams and fantasies: Implications for the activation-synthesis hypothesis. *Consciousness and Cognition*, *1*(2), 172–185. https://doi.org/10.1016/1053-8100(92)90059-J

Windt, J. M. (2019). *Dreams and Dreaming*. The Stanford Encyclopedia of Philosophy. https://plato.stanford.edu/cgi-bin/encyclopedia/archinfo.cgi?entry=dreams-dreaming

Responses to second round of reviews

We would like to thank the reviewers for their continued effort and dedication. We have made numerous changes to the article. Among these, we have reframed the paper, highlighting the methodological approach, which focuses on unveiling features of thought organisation. We have also added more interpretations and definitions of the measures.

We have also changed the way we analyse individual differences, using a permutation method. We have also removed all mentions of attempting to obtain convergent results via different statistical methods for the analysis using self-reported mental health symptoms, thus increasing readability and clarity. We have also added supplementary materials and figures with examples of the texts.

We have further specified the outcome of the pre-registered hypotheses and research questions. Following this re-work, we have asked the editor to make Dr. Mota a senior co-author.
To facilitate the review process, we have highlighted the reviewer comments in BOLD. We have indented quotations from the paper and highlighted changes in red.

REVIEWER REPORTS:

Reviewer #1 (Remarks to the Author):

In general, I found that the revisions did improve the paper. At the same time, I do not think some of the deeper issues were substantially attended to — mostly the comments by myself and Reviewer 3, which point out the submission tends to focus on their methods without linking them in very deep ways to the literature and existing theories. I also think some of the methods were applied in ways that make it difficult to understand the mechanistic contribution.

We thank the reviewer for their time and commitment in helping us improve the paper. Below we have endeavoured to answer each point in an exhaustive manner, including new analyses and a reframing of the paper.

Looking at the changes made to the submission, the authors have added some additional explanations and clarifications - but that is not exactly what the reviewers were requesting in the major revisions (in my opinion). I think the comments were asking for substantial changes to the framing, hypotheses, and connections to the literature - both in spontaneous thought and graph theory. Some of the responses instead simply pointed to previous publications to justify choices — which in my view is not sufficient. In some cases, the responses themselves were more sufficient compared to what actually made it into the paper.

We have endeavoured to engage in a major reframing of the paper. We now start the paper highlighting the methodological approach as a way to characterise organisation of thoughts expressed by individuals[1]. We subsequently frame hypotheses and interpretations in this manner. We also highlight the unrestricted forms of mind wandering[2] used that allow for greater comparability between the types of reports. We maintain the literature review as this remains relevant to the study. We copy the changes below

Lines 50-73:

The way we organize our thoughts is reflected in how we structure words, themes, and arguments when we speak and write. By examining the spontaneous associations that emerge in narratives, we can observe the ways in which individuals construct their interpretations of specific situations, thereby revealing aspects of their memories and life experiences. This notion underpins the concept of mental organization, which refers to the arrangement of declarative information into complex combinations that convey broader concepts beyond isolated parts.

Classic ideas such as William James's stream of consciousness[3] or the word-association methods explored in psychoanalysis have been given an objective methodological framework through natural language processing (NLP) approaches developed over the past 13 years, notably word recurrence graphs[1,4]. In these graphs, each spoken word is treated as a node, and the sequence of words in spontaneous speech is represented by directed edges. This method allows us to visualize a narrative as a trajectory of associations and recurrences, uncovering complex topological structures that are linked to the development of cognitive abilities such as verbal memory and social cognition[1,5,6].

Conversely, in the context of psychopathological conditions that impact memory and social cognition—such as dementia or psychosis—there is evidence of a loss of these topological properties in word recurrence graphs[1,4,7–11]. This finding suggests that such graph-based analyses can provide an objective means to access features of mental organization.

Investigating mental organization and event memory involves considering different physiological states of consciousness, such as dreaming and mind-wandering. Both states involve introspective narratives that are uniquely experienced and reported by the individual. Here we investigate the required mental organization to convey these highly personal, witness-free memories in written form.

Lines 106-108

Thus, we investigated the organisation of narratives when recalling introspective and personal memories from these different physiological states of consciousness, thus characterising the continuity and divergence of dreams and mind-wandering reports.

Lines 128-130:

In fact, studies of dream narratives in healthy participants, using the present method, have shown that dreams reported upon awakening from REM sleep are more complex and connected than those reported after slow-wave sleep (N2)[12].

Lines 156-167:

An important consideration in the development of the word recurrence graph methodology used in this study is the observation that dream reports reveal differences between the narratives of individuals diagnosed with schizophrenia compared to those without such symptoms, while everyday narratives do not show these differences[7,8]. Individuals without psychotic symptoms, or with affective psychotic symptoms, tend to produce more complex and connected narratives when describing dreams compared with daily reports, unlike those with schizophrenia diagnoses[7].

Interestingly, acute psychological distress—such as that caused by social isolation during the COVID-19 pandemic—can profoundly alter dream content (e.g., increased expressions of sadness and anger, contamination and cleaning themes) compared to pre-pandemic dreams by the same individuals, yet does not change the topological structure of dream narratives[13] . This suggests that such structural features may reflect stable individual characteristics.

Mind-wandering, on the other hand, has been conceptually related to rumination and empirically to negative mood, and as such is thought to be related to anxiety and depression[14–18].

Lines 142-144

In this study, we investigate whether the organisations of narratives of mind wandering and dream are consistent within individuals and dissociable between individuals, via the use of word recurrence graphs[1,4].

Lines 179-184

In this study, we thus investigated whether the organisation of mind-wandering and dream report architecture[1] may relate to self-reported symptoms of anxiety and depression.

The Present Study

In the present study, we applied graph theory to investigate the thought organisation structure of dream and mind-wandering reports.

Lines 189-191

We used an unrestricted form of mind wandering[2] allowing longer and naturalistic reports and greater comparability with the dream reports.

At the beginning of the results section. Lines 227-241

To analyse the flow of thoughts during the narration of a dream or mind-wandering within a word recurrence graph, we move beyond simply considering verbosity or the frequency of repetitions. Instead, we investigate the complex features of the trajectories used to connect words that collectively express these experiences. Drawing an analogy to a map in which each point represents a city, we can quantify the number of cities in each map—analogous to verbosity—and the number of visits to each city—comparable to word repetitions. However, when applying word recurrence graphs, we characterize the complexity of the routes taken to travel from one city to another, revealing whether the paths are more linear or more interconnected. Certain measures describe how well-connected specific cities are to others, the density of routes linking all cities, and the distances between the most distant cities given the possible pathways. These topological metrics capture the complexity of different ways in which memories of dreams or mind-wandering are expressed, organizing the sequence or flow of words to reflect underlying cognitive processes. Using this method, we tested whether dream and mind-wandering reports differed in network structure[7].

In the discussion. Lines 432-436:

In this study we sought to investigate the thought organization pattern while reporting inner experiences encoded during waking or sleep. Using word-recurrence graphs[1] we

characterised network structure differences between dream and mind-wandering written reports, explore individual differences in these, and examine whether such features predict mental health symptoms.

Lines 439-445:

Using these metrics, we found that that this method can distinguish between individual differences, that both types of report present different and complex patterns of mental organization in which dream reports tend to have a more “small-word” structure and higher intra-individual variation, probably related with diverse mechanisms to express memories encoded during waking or sleep. To conclude, we showed that dream and mind wandering reports could be used to predict clinically relevant variables.

Lines 447-448:

There seems to be two diverse patterns of mental organization expressed in the narrative when the encoded memory during waking or sleep stages.

Lines 453-459:

Specifically, dream reports were characterised by a more densely connected architecture and higher word repetition (i.e., with smaller lexical diversity, node centrality, in graph theory terminology). This effect was captured by the first principal component and is an important feature of the data, explaining high amounts of variance (in principal component analysis of highly correlated variables) and displaying the strongest effects (Fig 1C & Fig 3D). On the other hand, mind-wandering reports engaged higher lexical diversity and developed into longer structures.

Lines 470-476:

This pattern may reflect difficulties in organizing the narrative of a memory encoded during sleep, potentially due to reduced engagement of the default mode network (DMN) compared to wakefulness. In a recent study that presented affective images prior to sleep to investigate how emotional and mnemonic information is encoded during sleep onset, approximately 50% of the DMN structures were identified as sources of electrophysiological activity associated with the content and emotional residues of memories in dream reports collected during a nap[19].

Lines 560-561

Furthermore, in a 2 year follow-up sample of individuals expressing attenuated symptoms, graph theory features were predictive of a conversion to anxiety and depression[20].

Lines 568-581

In fact, tendentially, anxiety is particularly characterised by worry-driven repetitive thoughts about future situations[21], which may have been preferentially expressed in mind wandering reports. On the other hand, potential cognitive symptoms in depression may be more prominent during dream recall, which is cognitively complex (recall across states of consciousness), as noted above. Furthermore, higher word

recurrence in dream reports may reflect self-centred processing (e.g., repetition of "I", see in figure 4A for an example) or a ruminative focus on specific themes.

Other relevant changes are reported below, in particular in relation to the interpretation of the results and metrics.

Some of the updated language is useful but raises some additional questions. For example, the authors have updated the text to say they attempted to fit multilevel models for the mental health analyses. I guess I would argue that acknowledging that attempt is likely (and the several significant effects) are potentially misleading given that it is simply not an accurate modeling strategy (which is seemingly acknowledged). Saying that there were any significant effects is not really a good idea in my opinion, unless there is a way to suggest this is in fact the correct way to analyze the data.

Related, I guess I am a bit uncomfortable with some of the responses, such as "across many different permutations of the analyses, PC1 showed convergent effects." In a hypothesis driven, null hypothesis testing framework, why are there multiple permutations? This seems to be directly at odds with other places saying the study was pre-registered. Some of this was updated, but I think the issues raised by the other reviewers (confusing preregistration hypotheses and changes made) makes me wonder how valuable the preregistration actually was.

Following the reviewers comment we have now removed all mentions of attempts of modelling the data using mixed models and attempts at finding convergence.

In the face of complex analyses, with many possible analysis choices, checking the robustness and consistency of results may be considered appropriate (if it is testing the same hypothesis, e.g., in complex fMRI papers, this can be quite common by changing preprocessing methods, parcellation etc.,[22]). We have removed any mention of testing convergence, and now report one analysis per hypothesis, thus hopefully reducing confusion. We have also removed mentions of using correlations as an alternative analysis attempting to show convergence. We now only report multivariate statistics for the models where we averaged all data in a participant specific manner (following the reviewer's comment immediately below). Due to this less stringent approach (i.e., not searching for consistency across multiple statistical approaches), we now report one statistic for mind wandering and for dreams (we discuss this further in the related response below). Therefore, we report mind wandering reports as being related to self reported anxiety symptoms as this is significant according to this method. Please see the latest version of the results below:

Lines 393-408:

> We subsequently explored how the architecture of dream and mind-wandering reports predicts mental health symptoms, specifically, self-reported measures of anxiety (GAD-7) and depression (PHQ-9). We averaged across repeated measures to yield one value per individual and conducted analyses for dream and mind-wandering reports separately.
>
> The centrality of dream reports (i.e., word repetition) was likely to be associated with a higher PHQ-9 score (in ordinal logistic regression coef.=-0.47, [95% CI= -0.84, -0.10], p=0.013, Odds Ratio = 0.62; see Figure 3D). ~~There was another reasonably stable trend that emerged across analyses (see supplementary material 4).~~ The centrality of mind-wandering reports also showed a relationship with the GAD-7 score (in ordinal logistic regression coef.=-0.48, [95% CI= -0.94, -0.02], p=0.038, Odds Ratio = 0.62).

> There was a negative relationship between age and PHQ-9 (ordinal logistic regression, coef= -0.031, [95% CI= -0.05, -0.009], Odds Ratio =0.96, p=0.005), as well as with GAD-7 (iordinal logistic regression, coef= -0.031, [95% CI= -0.06, -0.005], Odds Ratio =0.97, p=0.016). This result might be related to greater psychological burden of pandemic-induced isolation to younger individuals or education level.

Following this we have removed the discussion of the multi-level model fitting from the discussion as well.

In relation to the pre-registration

We acknowledge that the pre-registration was underspecified, and as noted in the reviews, written at a high level. Part of this underspecification was indeed acknowledged in the preregistration itself (especially in reference to the data reduction). Following discussions amongst the co-authors, given the pre-registration is available online and had a role in shaping analyses, we feel that removing any mention of it may not be advisable.

We recognise that the pre-registration might have been much more precise in pre-defining analyses, and various contingencies might have been better considered and discussed.

Beyond the modifications precipitated by the first round of reviews, following the reviewers comment and the editors explicit encouragement, we now add further specifications in regards to the pre-registration and divergences from this.

In relation to Reviewer 3's previous comments that our predictions that dream reports would be unstructured could not be tested in an univocal manner (mentioned in verbal description of hypothesis 1).

Lines 814-817:

> Of note is that we predicted that graph theory analyses would show that dreams are more unstructured. However, due to a lack of a straightforward operationalisation, beyond the high level of covariance found between the metrics, this hypothesis could not be tested in a way that was conducive to univocal interpretations.

In relation to the modularity/segmented structures predictions (hypotheses 1.3 and 1.4 in preregistration):

Lines: 817-820

> Due to high collinearity, we could not test differences in modularity and hierarchy (these having high collinearity with other measures and being reduced in the principal component analysis. This possibility was specified in the pre-registration).

In relation to H.3.1. and 3.1.1 and 3.3.2 we add the following:

Lines: 409-411

> Contrary to our predictions (https://osf.io/arx2h), we did not find evidence of verbosity, path length and betweenness centrality predicting the relevant outcome variables.

In regards to modularity (h3.3 and h3.3.1)

Lines 881-884:

> Modularity, similarly to above, was found to have collinearity to other independent variables (Figure 1D, Table 3, Supplementary Material 2) and thus we could not test whether this would be related to self reported mental health symptoms.

Please also see Lines 568-570:

> Although cognitive symptoms are documented in anxiety and depression, we control for the verbosity of the reports in our analyses, and this variable did not show significant effects in contrast to our predictions.

I am still not entirely convinced by the argument that no stop words should be removed. The recurrent/repetitive nature that is mentioned in the manuscript could simply be just that (which would not require graph theory to determine) and may be related to the issues raised in my previous review — which is that it is product of the method not the phenomenon. At the same time, I understand the authors' argument that these words may be important to include. What I would suggest is that they remove a stop word list that contains highly commonly removed words, such as articles, prepositions, and pronouns — if the results are completely different after this removal, I would think that is an important point to note and would be useful for interpretation. This is related to the change that says the results indicate word level repetitions - but it'd be nice to know which words or categories of words, e.g.?

We thank the reviewer for their comment and note that they have an important point to make. We implemented a methodology that has numerous published and peer reviewed papers[1,4–8,11,19,23–27]. The method is designed to abstract quantifiable features out of complex verbal data. Repeating the entire paper with an alternative methodology would essentially produce another paper which would be relegated to the extensive supplementary materials and be only represented by one or more paragraphs in the limitations section. Importantly, this would be the case if the results were sufficiently amenable to synthesis). In fact, beyond the significant computational intensity and time, there is the additional issue that the results may not be comparable, potentially in non-predictable ways (e.g., different normalisation required, different results for the principal component analysis, statistical assumptions violated and different statistical methods needed etc.,).

Nonetheless, we appreciate the point that removing stop words might help interpretation. The reviewer notes important future directions to further develop this. We have added the following in the limitations section:

Lines 639-648

> We implement a method with several precedents (e.g., [1,4–6,8,20]). An interested reader may consult Mota et al., 2023[1] for an overview of the methodology. In this approach, stop words (i.e., common, grammatically functional words such as "I", "and", "in", "the") are not removed. These words may contain important information (e.g., the use of "I" as a subject rather than "me" as an object of a verb, the repetition of "and" in speech/thought organisation or narrative planning). This method is sensitive to the structure of verbal data rather than its content. Nonetheless, future studies may focus on exploring the importance of stop words in this method, across different conditions. This may be united with an analysis of the grammatical category of important words in these speech graphs.

Can the authors also justify further why age, country, and gender were included in the analyses? If these are removed, how much variance does PC1 explain?

These demographic variables were not included in PC1 as the object of this was to reduce only the graph theory variables that showed an inordinate amount of collinearity, thus rendering them amenable to valid multivariate statistics.

Lines 764-767

> Subsequently, a correlation matrix revealed that maximum-centrality, number of cycles, density, mean degree centrality, diameter, mean betweenness centrality, modularity, node number, largest connected component and strongly connected component were all highly correlated (R~0.7-1) (*Fig 1D)*. Thus, these measures were inserted in a principal component analysis and three principal components extracted.

Age can have an effect on reporting style[7,28]. The peculiar relationship between age and some verbal components of IQ is well known (tends to increase slightly rather than decline like processing speed).

Furthermore, age may have interacted with the effect that the pandemic may have had on subjects.

The use of culture may be justified by the fact that there may be differences in reporting style and manner of expressing themselves. Thus controlling for this might have been considered appropriate.

Finally, there may be some gender differences of interest. For example, beyond reported effects of sex differences in verbal fluency[29], differences in dreams have been reported in the literature[30].

I am also still confused how the Manny Whitney U test were conducted. The Man Whitney test allows for within subjects data when compared as a paired comparison but it does NOT allow for multiple observations per person in a single group. It seems like the authors are still taking multiple reports per person, rather than averaging to participant level. Further, there are uneven samples collected from people, so not including some sort of random effect is also problematic because it is effectively weighted — but the more important point is that it is likely not the appropriate test here. This may be an issue with clarity but I'm struggling to follow how this analyses was not done at the report level.

The Man Whitney U tests is for independent samples (Matlab and R functions for paired samples cannot accept unequal sample sizes, and thus would give an error).

To test our hypotheses), we used it to compare how the <u>similarities between reports</u> differ rather than a measurement of a person. We are not taking multiple measurements per person, but measurements of the similarity/difference between reports (e.g., similarity of reports within the same person vs similarity of reports between different people). Mann Whitney U tests should be robust to unequal samples sizes.

Addressing the most important point, we considered similarities within people and between people as independent (as well as between report type), as these are not repeated

measures (the similarity between two reports was never repeated). However, we understand that this analysis can be considered quite unorthodox.

In response to the reviewers comment we implemented a permutation method, which is notoriously robust and light in terms of assumptions. However, the unequal sample sizes may become a problem during the creation of the permuted null distribution. We solved this using a downsampling approach, whereby the larger sample size was downsampled to the same length as the condition with the lowest sample (this was repeated for each permutation). Please see the following changes in the method section:

Lines: 845-855

> We investigated if the reports written by the same person were more similar compared to the reports from other individuals using a non-parametric permutation approach. This involved randomly shuffling condition labels (e.g., within vs between person similarity) 1000 times to create a null distribution from the data. This null distribution was then compared to the test statistic (difference between medians). Due to the highly unbalanced sample size, each permutation involved a random sampling of the condition with the highest number of observations so as to match to the number of observations in the lowest condition. This ensured that the null distribution was appropriately defined. We present the median differences of robust z-scored values (using median absolute deviation and median) to enable ready interpretation of the effect size. We also present the robust Cohen's d; the Cohen's d and 95% confidence intervals statistics calculated using the Matlab meanEffectSize function.

The results reproduce which is reassuring. Please see the following changes to the results section.

Lines 342-363

> We found that the structure of dream (median difference=-0.62, p<0.001, standardized median difference=-0.33; robust Cohen's d=-21; Cohen's d=-21, C.I.=-0.23:-0.19) and mind-wandering (median difference=-1.10, p<0.001, standardized median difference =-0.65; robust Cohen's D= -0.42; Cohen's d=-0.38, C.I.=-0.41:-0.36) reports were more similar within individuals than between them. Thus, the network structure of dream and mind-wandering reports does contain individual specific information (*Fig 3C*). Similarly, we found that dream reports were more similar to mind-wandering reports when written by the same individual than when written by different individuals (median difference=-0.31, p<0.001, standardized median difference =-0.17; robust Cohen's d=-0.11, Cohen's d=-0.10, C.I.=-0.11:-0.08).
>
> Finally, within-individual similarities of mind-wandering reports were higher than within-individual similarities of dream reports (median difference=-0.82, p<0.001, standardized median difference =-0.51; robust Cohen's d= -0.34; Cohen's d=-0.30, C.I.=-0.33:-0.27). This indicates higher within-individual variation in dream reports (i.e., higher complexity) (*Fig 3C*) and indicates a diverse pattern of mental organization to narrate memories encoded during sleep. Although not specified in the preregistration, we also investigated whether the similarity between an individual's dream and mind-wandering reports was higher than similarities between dream reports from different people. We indeed found that the within-person similarity across dream and mind-wandering reports was higher than between-person dream similarity (median difference=-0.20, p<0.001,standardized median difference =-0.11; robust Cohen's d= -0.08, Cohen's d=-0.07, C.I.=-0.09:-0.06), further indicating an individual specific

stability of mental organization pattern expressed in writing style and phenomenology compared to that of between person dreams.

The addition of the r values is useful, but also highly concerning in terms of practical effects. I realize this is mentioned, but again it seems like the sample size of reports might be “revealing” significant p values but extremely small effects overall.

This is indeed the case. A concern is that the high number of observations will have severely affected the r value. This is because the total number of observations is inserted in the denominator in calculating the r value (in some cases going into the millions, given all possible combinations). Following the reviewer’s comment above, we have removed the r values which relate to the Mann Whitney U test. We present a median difference on robust Z scored values, which enables an easy interpretation in terms of the median difference. We also present robust Cohen’s d.

This shows that there was between small and medium effect sizes. This is due to the effect size being standardized by measures of variance rather than the number of observations. We have therefore modified the following.

Lines 528-530

> ~~Although effect sizes were small,~~ These findings may imply that writing style and the way people express their experiences is similar within individuals.

Lines 630-632

> Although we find that the present method is sensitive to individual differences~~, effect sizes were small and~~ we could not exhaustively elucidate what might be driving such differences.

Some of the graph theory metrics that are showing differences are still not well explained. I think it would be useful to explain these theoretically more, as well as perhaps comparing them against more simply measures that theoretically do not capture the same as what the graph theoretic measures do - i.e. verbosity, proportion of word repetition, etc.

Beyond the responses above, reframining the paper in relation to the methodological paradigm, we have made several modifications to further explain these metrics theoretically more:

In particular we copy table 1, with explanations of each metric.

Lines 292-293

Table 1. Descriptions of graph theory metrics that are relatively independent from one another.

Metrics	Description

PC1-Centrality	This is sensitive to lexical diversity. How dense are the graph connections? Are there more nodes (words) connected with fewer possible pathways, i.e. higher lexical diversity connected through fewer possibilities of pathways, fewer repetitions of central words? (note: this aspect of networks explains other graph theory measures (see methods section and S2 for further details)).
PC2-Diameter	How far away are the two extremes of the network? Does it take many "steps" to get from one side of the graph to another? How linear are word trajectories? Reveals lower connectedness between distant words, fewer repetitions that do not connect large amounts of words (probably repeating locally in the sentences).
Clustering Coefficient	Are there "cliques" or clusters in the report? The extent to which neighbours of one node are also neighbours amongst themselves. How the words connected to another word, are also connected to each other.
Maximum betweenness centrality	Are the points in the network through which lots of paths tend to go through? Are there essential "bridges" in the report? Are there important words that connect a variety of other words in the narrative?
Assortativity	Are highly connected words also connected to highly connected words? Are minimally connected nodes connected to minimally connected nodes? Are repetitive words linked in sequence to each other?
Report length/verbosity	How many words are in the report? What would be the total number of edges if the sliding window approach was not used?

At the start of the results section we have added the following, which uses explanations that are typical with graph theory and network science:

Lines 227-240

> To analyze the flow of thoughts during the narration of a dream or mind-wandering within a word recurrence graph, we move beyond simply considering verbosity or the frequency of repetitions. Instead, we investigate the complex features of the trajectories used to connect words that collectively express these experiences. Drawing an analogy to a map in which each point represents a city, we can quantify the number of cities in each map—analogous to verbosity—and the number of visits to each city—comparable to word repetitions. However, when applying word recurrence graphs, we characterize the complexity of the routes taken to travel from one city to another, revealing whether the paths are more linear or more interconnected. Certain measures describe how well-connected specific cities are to others, the density of routes linking all cities, and the distances between the most distant cities given the possible pathways. These topological metrics capture the complexity of different ways in which memories of dreams or mind-wandering are expressed, organizing the sequence or flow of words to reflect underlying cognitive processes.

Furthermore,

Lines 296-299:

> A mixed-effect logistic regression showed that higher PC1 or lower density/centrality (i.e., higher lexical diversity); coef.= 0.83, p<0.001), lower PC2 or higher diameter (more difficult to get from one side of the text to the other, or longer structures) (coef.= -0.20, p<0.001), and higher verbosity (coef.=0.14, p=0.002)

Lines 306-307

> Thus, mind-wandering reports were less dense with higher lexical diversity and presented longer trajectories/structures.

Lines 312-315

> It seems that in dream recall, a higher number of repeated words connects the reports over long and short distances, whilst the more verbose mind-wandering reports develop into longer structures. There seems to be two diverse patterns of mental organization expressed in the narrative when memory encoding occurred during waking or sleep stages.

Lines 323-324

> *Note that the dream report network (panel A), as compared to the mind-wandering report network (panel B), tends to have lower lexical diversity (PC1),*

Lines 355-356:

> This indicates higher within-individual variation in dream reports (i.e., higher complexity) (*Fig 3C*) and indicates a diverse pattern of mental organization to narrate memories encoded during sleep.

Lines: 358-363

We indeed found that the within-person similarity across dream and mind-wandering reports was higher than between-person dream similarity (median difference=-0.20, p<0.001,standardized median difference =-0.11; robust Cohen's d= -0.08), further indicating an individual specific stability of mental organization pattern expressed in writing style and phenomenology compared to that of between person dreams.

Lines 397-398

Lower PC1 (i.e., lower lexical diversity, higher density/centrality) was likely to be associated with a higher PHQ-9 score

Lines: 401-403

(please note figure 4 is copied below in response to a comment by reviewer #2)

This means that lower lexical diversity was associated with more severe depressive and anxiety symptoms scored by the participant (see figure 4 for examples).

Lines 439-445

Using these metrics, we found that that this method can distinguish between individual differences, that both types of report present different and complex patterns of mental organization in which dream reports tend to have a more "small-word" structure and higher intra-individual variation, probably related with diverse mechanisms to express memories encoded during waking or sleep. To conclude, we showed that dream and mind wandering reports could be used to predict clinically relevant variables.

Lines 453-459

Specifically, dream reports were characterised by a more densely connected architecture and higher word repetition (i.e., with smaller lexical diversity, node centrality, in graph theory terminology). This effect was captured by the first principal component and is an important feature of the data, explaining high amounts of variance (in principal component analysis of highly correlated variables) and displaying the strongest effects (Fig 1C & Fig 3D). On the other hand, mind-wandering reports engaged higher lexical diversity and developed into longer structures.

Lines 496-502

The encoding and recall during wakeful consciousness may facilitate the mental organization of inner experiences such as mind-wandering, enabling participants to construct narratives with greater lexical diversity and a more linear sequence of events. This typically involves the repetition of linking words that connect the beginning and end of narrative topics. In contrast, dream reports often exhibit repetitions within narrower thematic segments, possibly reflecting an effort to recall fragmented details as well as in structuring the experience into a coherent and logical account.

Lines 549-551

Capitalising on this unique and extensive dataset, we found that graph theory analysis of natural language may relate to mental health symptoms, in accordance with previous literature using this method[1,7,8].

Lines 554-557

We found that patterns of word repetition in dream and mind-wandering reports were related to self-reported symptoms of depression and anxiety respectively. Lower density/centrality and, thus, a higher diversity of words in dream and mind-wandering reports was correlated with reduced negative symptomatology.

Lines. 560-562:

Furthermore, in a 2 year follow-up sample of individuals expressing attenuated symptoms, graph theory features were predictive of a conversion to anxiety and depression[20]

Lines 564-567:

Importantly, higher word repetition may be driven by symptoms that characterise depression. Lower cognitive flexibility, focus on the self, and higher rumination (which has been related to mind-wandering and negative affect[40]) may be driving higher word recurrence that impacts lexical diversity.

Please also note explanations in figure 1;

Lines 261-265

“clustering coefficient (indicating the cliqueness of the network, i.e., the extent to which neighbours of a node are also neighbours), maximum betweenness centrality (the maximum amount of shortest paths that pass through a node), assortativity (similarity between the connectivity/degree of neighbouring nodes), and report length or verbosity (Fig. 1E; Table 1)”

Lines 461-463

This network structure is characterised by clustered, interconnected neighbours (higher clustering coefficient) and a simultaneous ease in long-distance communication (lower path length).

Lines 465-466

but were, overall, also more interconnected (there were words that created shortcuts, that facilitated efficient transitions across the network).

Lines 491-496

mind-wandering reports had a more linear, extended structure with essential transition points in the report structure (higher max betweenness centrality). These are connecting words that create “shortcuts” that connect the network in the more linear and extended structure that characterised mind-wandering reports. Importantly, mind-wandering reports also had less repetition of words (i.e., centrality), which may be of a syntactical or semantic nature[59].

Lines 559-560

which would translate to higher node centrality (lower lexical diversity) in a graph theory context

Lines 732-735

> Thus, repetition of words strongly drives density, number of connected components, centrality, modularity, numbers of cycles etc., (Fig 1D), whilst the second principal component tends to represent how extended, overall, the network is (how easy is it to get from one side of the network to the other), somewhat indicating the “integration” of the report with itself rather than a non-recurrent linearity.

Please note, for brevity, here we do not insert all available explanations of the metrics in the paper, of which some use a graph theory language (sometimes considered part of pure mathematics, despite extensive empirical applications), whilst other give intuitions of the method to non-experts.

As explained throughout the paper, please note that verbosity was measured and analysed as a independent variable (e.g., table 1 and 2).

As shown in figure 1D (as well as being discussed in the methods), this was statistically independent from the other metrics following the sliding window approach. However, if the sliding window approach was not taken, this would not be the case (verbosity correlates with number of repeated words). We have added the following:

Lines 503-505

> Participants were also more verbose in reporting mind-wandering experiences (a feature which is independent from other graph theory metrics).

We appreciate that other natural language processing methods may provide further characterisation of the measures used in this study in an additive manner in relation to the explanations inserted above, as suggested by the reviewer, and the previous publications (e.g., [6,8,9,23,26]). Although interesting, this may amount to an entirely different paper altogether, which is methodological in nature in that it elucidates and develops the method (Mota et al.,), rather than focused on characterising mind wandering and dream reports with this method with several peer reviewed publications, as was the intent of this work. As such, this might require other methodological implementations and considerations and, possibly, further data.

In function of the reviewer’s comment, we have inserted the following as a future direction:

Lines 651-652:

> Furthermore, this method may be united with other natural language processing methods to further characterise the features this approach is sensitive to.

Following the re-framing of the paper we have also inserted the following:

Lines: 227-230

> To analyse the flow of thoughts during the narration of a dream or mind-wandering within a word recurrence graph, we move beyond simply considering verbosity or the frequency of repetitions. Instead, we investigate the complex features of the trajectories used to connect words that collectively express these experiences.

How is each sliding window represented in the data? (i.e. does that create more instances)

Each statistic used in the paper was calculated from the mean of all windows of a given report. Each window had 30 words and was slided by one word. Thus the windows are overlapping. This is typical in a sliding window technique. In response to the reviewer's question, please see the further clarification in the main text.

Lines 719-727

> Importantly, to ensure that the word count (report length) would not influence graph theory properties[1,7,31], we applied a sliding window approach where 30 words (as sequentially found in the report) were selected and a network created out of those. The window was slided by one step (i.e., one word) and the process repeated until that instance of text was exhausted. Thus, no windows are perfectly the same but are overlapping with all adjacent windows. The averaging ensures the ensuring metric is representative of the report whilst controlling overall verbosity. Given the lack of correlation between the total word count of the report and all network properties (*average r=0.025*), this approach was successful in controlling for network size (which has a substantial effect on most graph theory measures).

I'm still not sure what to make of the 91% variance. I understand the authors response logically, but I guess I'm trying to understand how to make sense of this theoretically still and what the contribution of this is then. For example, the authors say the first component captured density/centrality in their summary of the PC, but it actually had way more variables in it and the loadings are not clearly presented in the manuscript.

We thank the reviewer for pointing out the lack of clarity. We have now moved the table with loadings from the supplementary material to the main text and have inserted more information in the methods section.

Lines 770-780

> In the first PC, the variables loaded more-or-less equally, as implied by the high explained variation. Mean degree centrality and density had the highest loadings indicating the repetition of words was the main contributor to this principal component (table 3). In the second component, diameter and mean betweenness centrality (also based on path length, i.e., distance between two nodes) had the highest loadings. We decided to interpret this primarily in terms of diameter as this is more intuitive and avoids confusion with another metric which is maximum betweenness centrality. The final PC was primarily driven by max centrality, however, as explained below, this component could not improve inferential statistical models and therefore was not included in the final analyses. This analysis was repeated averaging all within participant repeated measures. No major differences in the data reduction results were found.

Lines 781-784

Table 3. Variable inserted in the PCA with loadings onto the principal components (sign also displayed). Highlighted in BOLD are the highest loadings onto PC1 and PC2

	PC1	PC2

Diameter	0.306	**-0.546**
Density	**-0.326**	-0.239
Node Number	0.324	0.264
Mean centrality	**-0.326**	-0.239
Maximum centrality	-0.285	0.045
Modularity	0.313	-0.114
Cycles	-0.320	0.045
Largest Connected component	0.324	0.264
Largest strongly connected component	0.324	0.264
Mean Betweeness centrality	0.305	**-0.600**

I suggest using three significant digits throughout the paper.

We have now modified the text so that p-values have three digits (decimals).

Finally, the authors have added a limitations section which is great. However, in my first review, I pointed out some serious concerns with the the methods and if they are really representative of the phenomenon of interest. The issue is that the methods are also a bit of the "lead" here, which may be a problem without really grappling deeply with how this relates to the theories.

Following the reviewers comments above we have reframed the paper highlighting the theoretical foundations of the method as well as the phenomena in question (for brevity, we do not repeat changes that we have inserted above). It is true that this data was not collected with this technique in mind (See Sikka et al., for the first paper published with this dataset). Nonetheless, in designing the experiment we prioritised comparability between the phenomena of interest (dreams and mind wandering reports).

For this we have inserted this in the introduction.

Lines 189-191

> We used an unrestricted form of mind wandering[2] allowing longer and naturalistic reports and greater comparability with the dream reports.

Reviewer 2 below asked us to provide more practical examples of the phenomenon. We resolved actual reports in a network structure and present them in a new figure (we do not copy this here to avoid repetition as it is below). In function of this we have also inserted a new supplementary material … which has complete examples of reports, a pictorial representation of their graph theory structure, and all the metrics that are relevant to the paper.

Reviewer #2 (Remarks to the Author):

I appreciate the authors' efforts in addressing my comments, which have certainly helped clarify some aspects of the manuscript. However, some of my original questions remain and I raised a couple of new comments based on the newly added sections.

We thank the reviewer for their renewed engagement and significant assistance in helping us improve this paper. We have tried to answer their concerns in a comprehensive manner.

Depth of Result Interpretation in the Discussion
I understand the authors believe that they cannot provide interpretation beyond a literal interpretation of the structure, because they cannot be sure that interpretation applies to all reports. Though I'm not entirely sure why an example cannot be used to explain the findings, even if this example doesn't apply to all the reports. Although I

appreciate the data is variable and complex, I found this rather dissatisfying, especially for a psychology journal. If we apply sophisticated analysis tools on a dataset but cannot make sense of the data in terms of the phenomenology (which I believe is core to this type of naturalistic data), then it raises the question of how useful is that tool. I think it's important for the authors to interpret the results in a concrete way. Please refer to my original review comment for more details.

We thank the reviewer for their interest in rendering our analyses interpretable. We appreciate that a lack of examples may give the impression of a lack of concrete interpretability. Although graph theory analyses are used for largely uninterpretable data (e.g., large and highly complex brain networks), in the present case it is possible to relate the graph theory metrics to the original report in a relatively interpretable manner.

There are figures 1E and 3A which should give intuitions as to how metrics represent different aspects of the network structure. For further examples to be inserted in the discussion and results, the reports can be rather long, and the insertion of the report in the discussion would induce a significant lengthening of the discussion (approx 900-1000 words). The features captured by the metrics capture may not be immediately obvious from the pure text. Furthermore, a longer example would be necessary to show repetition of words. Therefore, following the reviewer's advice, we have decided to insert a new figure 4 with the words represented and resolved in a network structure. This both allows the reader to see the words of the text and the network structure at the same time. This also has a description of their self reported mental health symptoms. Then, an interested reader can review the original full text from which these figures, in the newly inserted supplementary material … . This has the relevant values of the graph theory metrics used in the paper, as well as a plotted network structure of the relevant text (this uses the whole text, and thus words could not be laid over without obliterating readability).

Please see the new additions below:

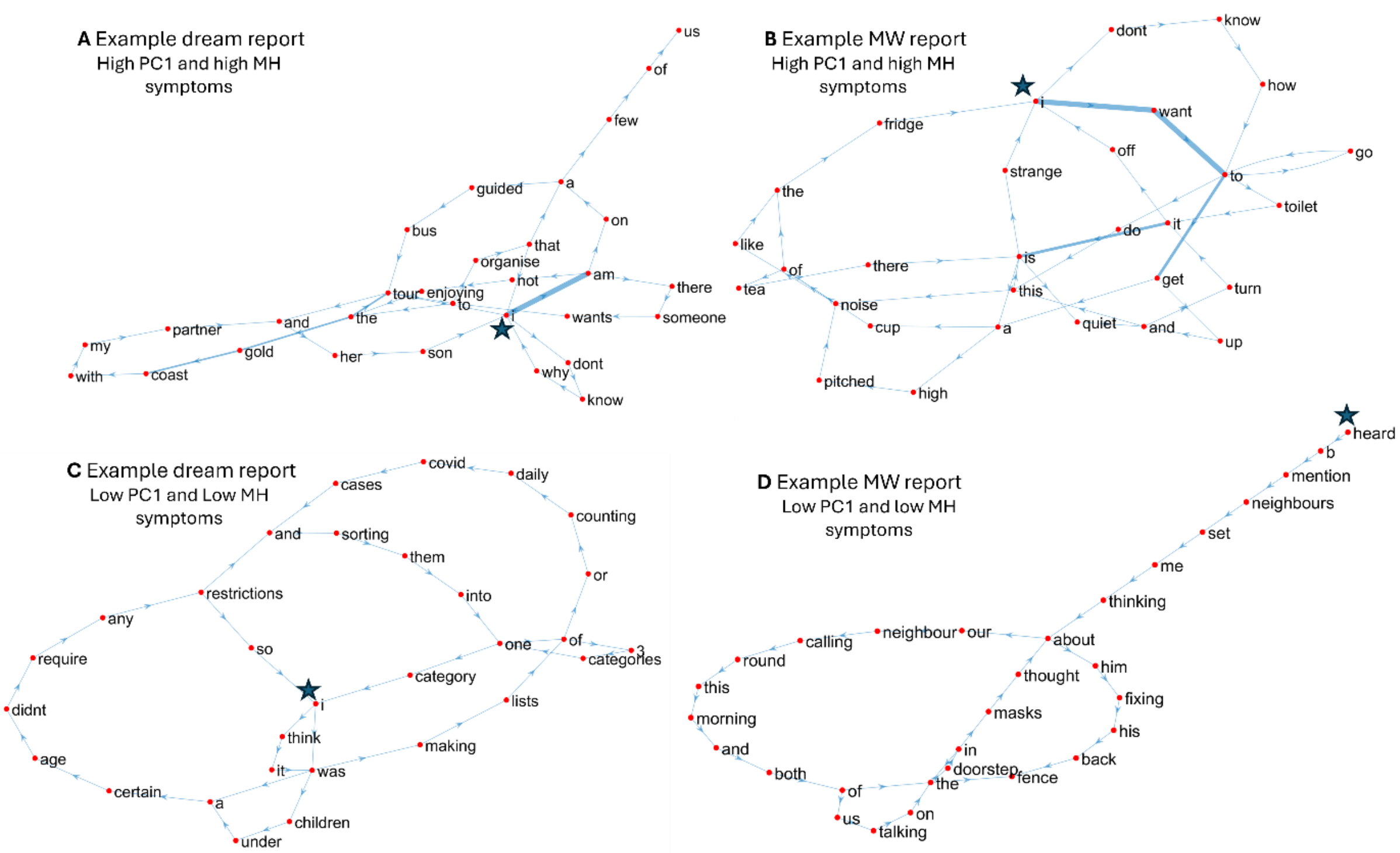

Lines 415-429

Figure 4. Examples of dream and mind wandering graphs with high and low PC1 and self reported symptoms.

The top left quadrant (A) shows an example of a dream report with high PC1 and high self-reported symptoms (PC1=-3.01, PHQ-9=17, GAD-7=11). From the same participant, the top right quadrant (B) shows an example of a mind-wandering report with high PC1 and high self-reported symptoms (PC1=-2.47, PHQ-9=17, GAD-7=11). From a different participant, (C) the bottom left quadrant shows an example of a dream report with low PC1 and low self-reported symptoms (PC1=2.58, PHQ-9=4, GAD-7=2), whilst the bottom right quadrant (D) shows an example of a mind wandering report with low PC1 and low self-reported symptoms (PC1=3.07, PHQ-9=4, GAD-7=2; same participant as C). Please note this is for illustration purposes only. In this figure, all networks here have 30 nodes plotted. Please see supplementary material 5 for the full text used in this figure with all the relevant graph theory metrics that are specific to those dream and mind wandering reports, as well as a pictorial representation of the graph. Thicker lines indicate repeated sequences of words.The star indicates the beginning of the report. MH symptoms = self-reported mental health symptoms. PC1= PC1-centrality.

Below is the newly inserted supplementary material with the full and original texts, a picturally represented graph theory structure, and the relevant metrics:

Supplementary Material 5

Example full dream and mind wandering reports with relevant graph theory metrics. Figure 4 in the main text was derived from these examples below.

Also below each example is the plotted network structure.

Example of dream report with high PC1 and high self-reported symptoms (PC1=-3.01; PHQ = 17, GAD =11, PC2=0.36, Clustering Coefficient=0.81; Word Count=-0.24; Max Between Centrality= 0.51; Assortativity =-0.22)

I am on a guided bus tour to the Gold Coast with my partner and her son. I am not enjoying the tour, I don't know why I am there. Someone wants to organise that a few of us break away from the tour and go drinking that night, and they are trying to involve me in the plan, but I am trying to excuse myself. i explain that I used to live near the Gold Coast, so I am familiar with it and it is not new. I say that I used to always drive through the town to cross over the border, but I would never stop there. I am trying to say this while being polite and not put them off their excitement for their night out.

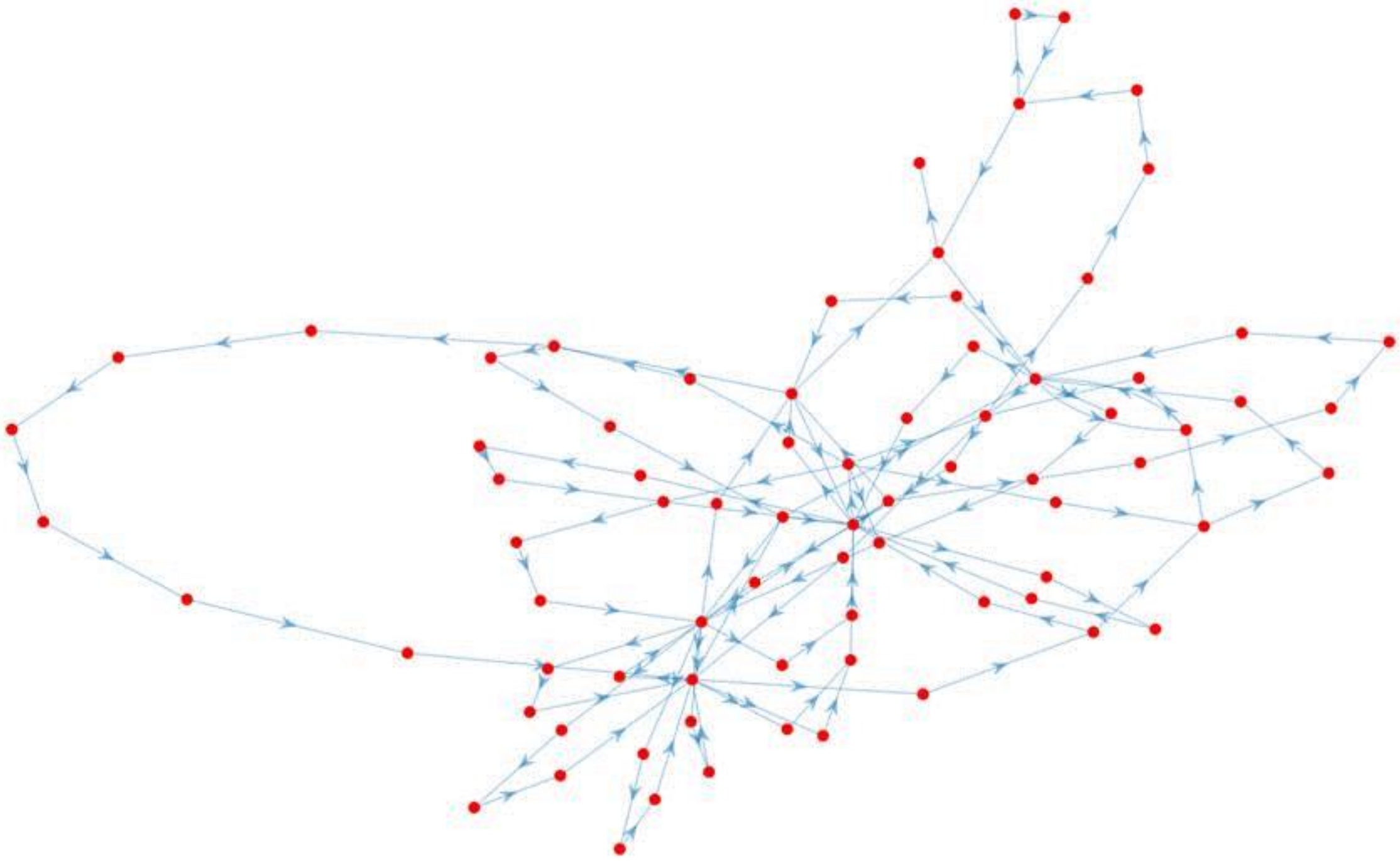

Example of mind wandering with high PC1 and high self-reported symptoms (PC1=-2.47, PHQ=17, GAD = 11, PC2=-0.91, Clustering Coefficient=0.96; Word Count=1.12; Max Between Centrality=0.55; Assortativity =-0.14)

I don't know how to do this noise of the fridge: I want to get up and turn it off I want to go to toilet it is quiet, and this is strange I want to get a cup of tea There is a high pitched noise, like the sound of crickets; I can't tell if it is in my head or outside. This happened the other night, when I thought heard a faulty alarm all night, when I went to check, it turned out it was inside my head, Then I was afraid that this might be the beginning of hearing voices. I want to do/act/move/distract myself. I think of maintenance tasks around my home a sense of 'I should' I should have a to-do list next to me to write these things down I'm sad it's more than sad, a sense of tragedy, an all-pervading tragedy of life, that is at the same time very sweet. I could easily be close to tears. at the same time, a very physical sense of 'letting go' into myself. something in me just stops I am very aware of the front of my body there is a tingling at my genitals this reminds me of a time when I used to practice and teach yoga a sense of a time when I could just stop doing things, when time was my own I anticipate cooking dinner, or going for a run I should go to the office to get some work done I should turn on my phone sadness/ I think of a time I left a partner, and her daughter's horse died shortly afterwards. The dying horse is connected to a time I wrote poetry, it is a line in a poem I wrote after a beloved aunt's death. lost innocence/grieving the feeling has moved to my chest now the feeling has moved to my brow I think of friends who I should recommend doing this study to I think I should post something on facebook about the study, to encourage people to try it.

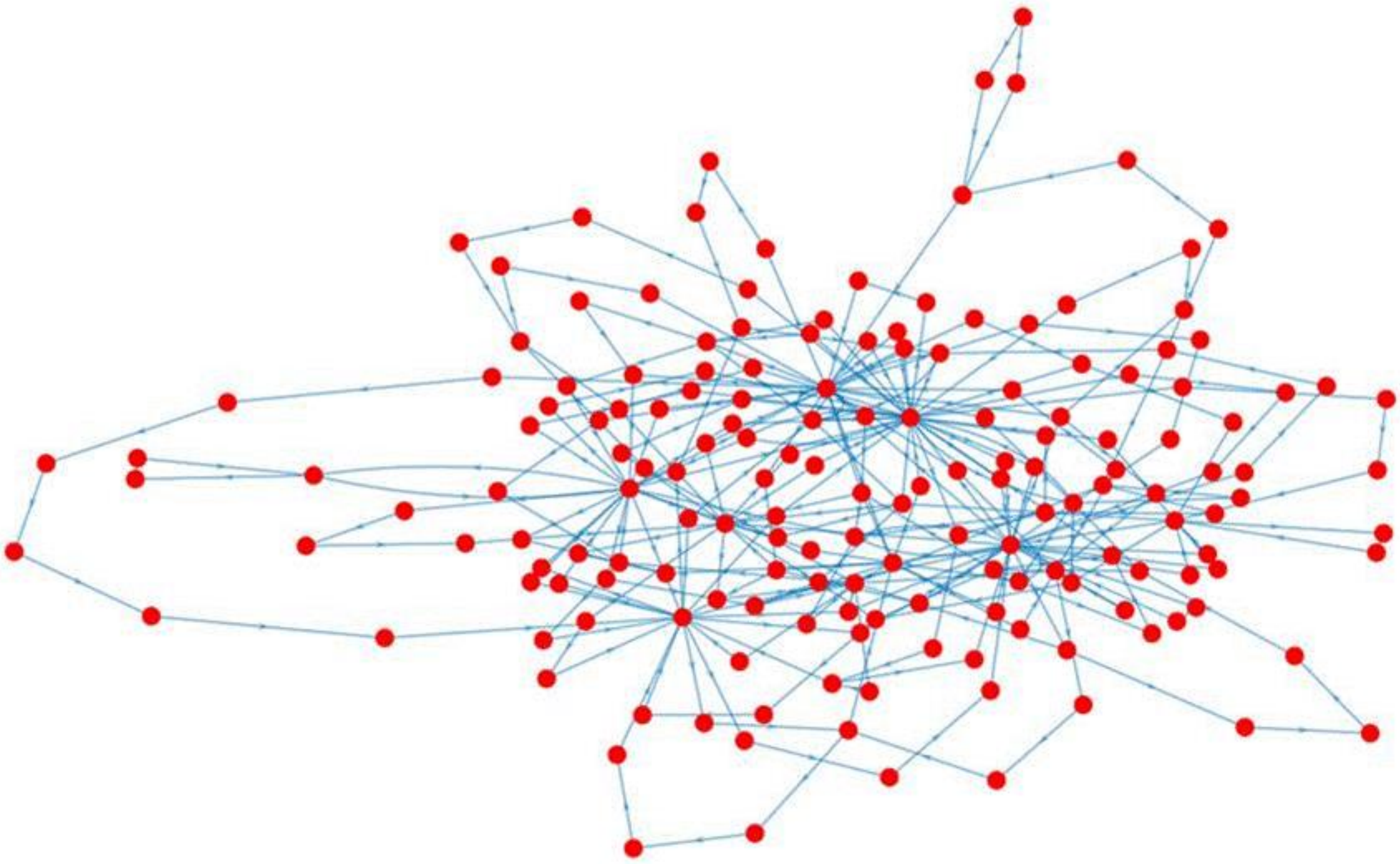

Example of dream report with low PC1 and low self-reported symptoms (PC1=2.58, PHQ = 4, GAD= 2, PC2=0.16, Clustering Coefficient=-1.31; Word Count=-0.81; Max Between Centrality=0.63; Assortativity =0.13).

I was making lists of, or counting, daily Covid cases, and sorting them into one of 3 categories. One category, I think it was children under a certain age, didn't require any restrictions, so I felt pleased if I could include a name in that list, but also anxious about getting it right. A second group was a bit ambiguous - maybe with limited restrictions, and the third group was fully restricted. The lists were piles of pieces of lined brown/manila paper, written on in pencil.

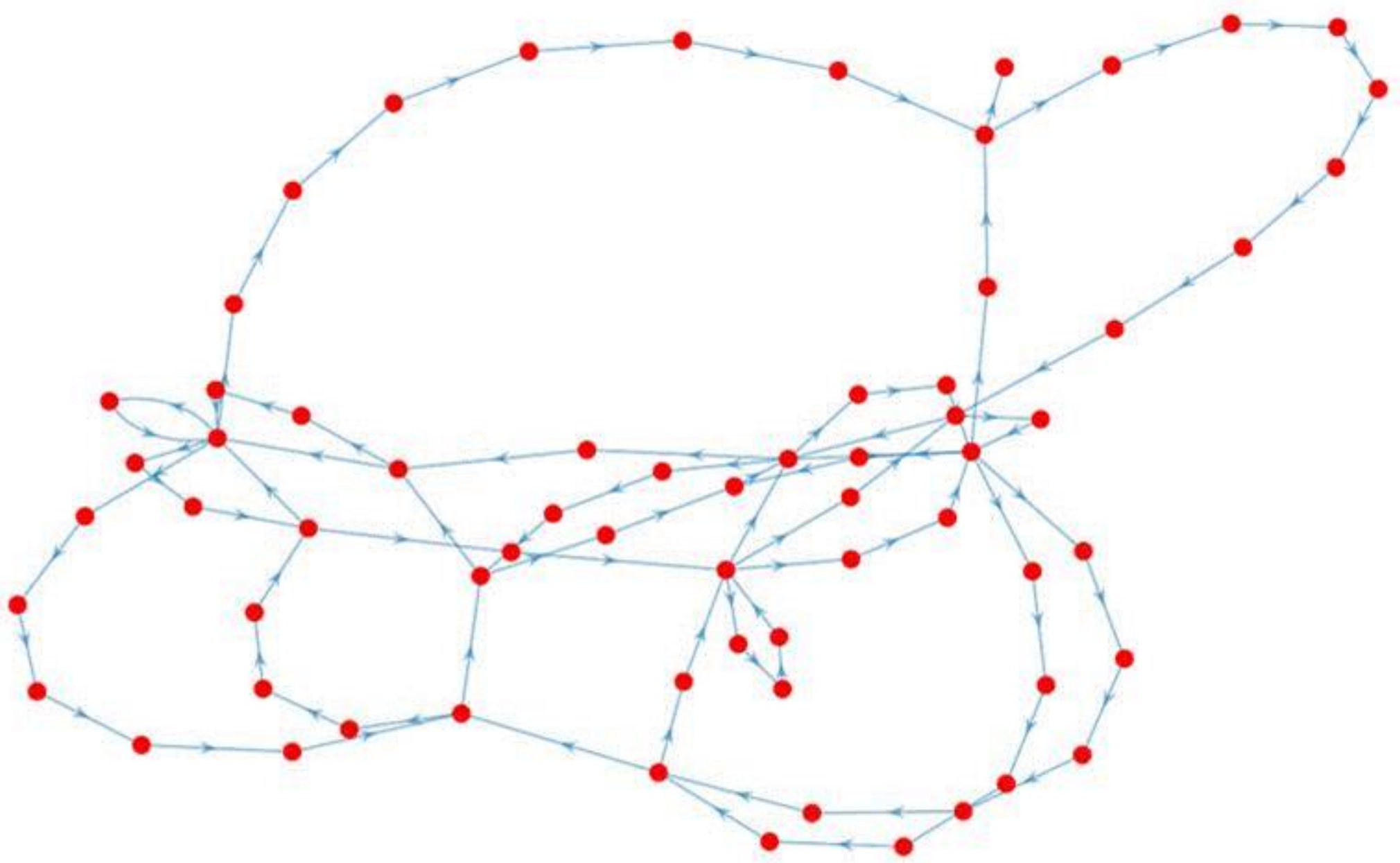

Example of mind wandering with low PC1 and low self-reported symptoms (PC1=3.07, PHQ=4, GAD=2, PC2=0.29, Clustering Coefficient=-0.128; Word Count=0.32; Max Between Centrality=0.58; Assortativity =-0.006).

Heard B mention neighbours. Set me thinking about our neighbour calling round this morning and both of us talking on the doorstep in masks. Thought about him fixing his back fence that blew down in the wind & how the wind pulled the nails out of the fence posts. It's windy again tonight. I din't like wind. I'm getting better with my clarinet lessons. Got to play high notes today. Nice to see Ann, the music teacher online. I wish Al would play his saxophone more. Will he move in with T next year. If he does who will look after our house if we travel. Thinking about the old dolls house upstairs where I am & how we moved it to make room for filming our pan pipe tune this afternoon, and when will we be able to play as a group again and wondering how Ga is going with his Parkinsons - Parkinsons, not Alzheimers, I was getting them mixed up. And Shane Jacobson at the Parkinsons walk every year & people trolling him for doing the Covid ads. Steve Moneghetti is always at the walks too. So thin. It's still windy. The wind's rattling the windows.

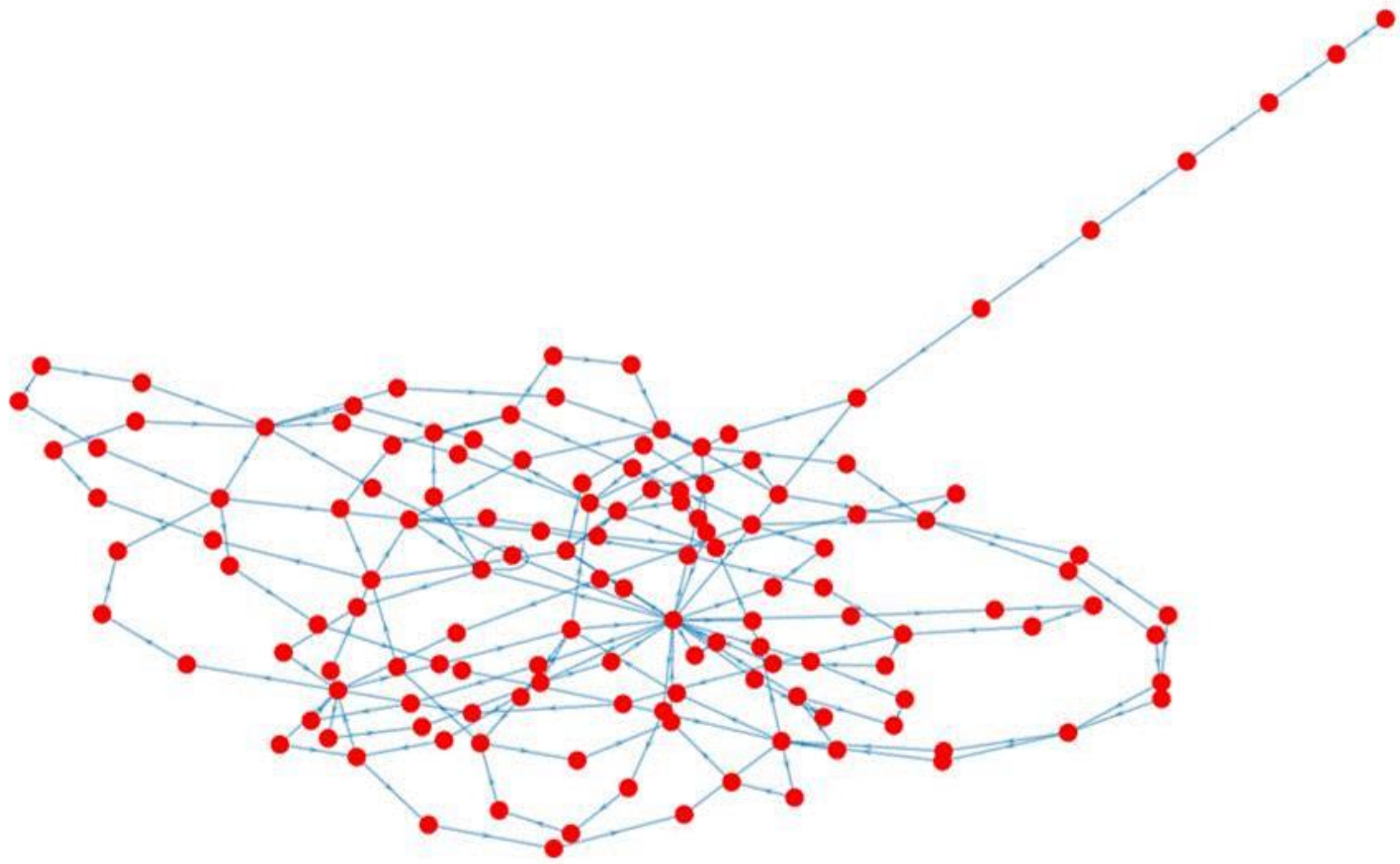

Alternative Explanations

I understand that the authors have instructed participants to report contents that enter their mind when they are not engaged in or focused on any specific activity or task, which was clearly stated in the Methods section. My original question pertains to whether authors verified that. Since the entire study is grounded in this definition of mind wandering (which the authors now specified is based on dynamic thoughts (Christoff et al., 2016)), it would be important to verify that participants actually did not “engage in or focused on any specific activity or task”. In behavioral tasks, we can use performance to determine whether participants were performing the task as instructed. In these types of tasks, it’s more difficult as there are no clear ‘behavioral measures’, but that shouldn’t prevent one from implementing post-hoc measures, such as questions to verify. A more concrete way to address this is to actually look at their reports to see if the content was focused on a specific thought. As participants are instructed to “allow your mind to wander freely”, if the thoughts were mostly focused on a topic, then this would mean the participant did not follow the instructions and the state that was captured likely does not qualify as “mind wandering”.

We thank the reviewer for their comment. In relation to this, as part of the data curation process members of our team reviewed the reports and removed anything that was “meta-narrative” (an example might be “now I shall start writing”) or meta-cognitive components so only the relevant content was retained.

Lines 696-698

> Before analysis, reports were pruned, removing all commentaries on the experiences that were not part of the actual experience.

Outside of this we did not further modify or exclude data retaining the naturalistic element of the data as much as possible.

Importantly in this paper, we decided to focus on unrestricted forms of mindwandering https://pubmed.ncbi.nlm.nih.gov/38767718/ as this allowed for greater comparability between dreams and mind wandering reports. This objective did not allow us to use thought sampling, as we required longer reports, which are necessary for the use of graph theory (minimum number of nodes = 30).

It may be arguable that one may mind wander but fixate on a topic. This would be relevant to forms of rumination, may be considered naturalistic and is highly relevant to the self-reported symptom data used in this paper.

In function of the above, we have inserted the following in the paper:

Lines 189-191

> We used an unrestricted form of mind wandering[2] allowing longer and naturalistic reports and greater comparability with the dream reports.

PCA and Individual Features

Regarding the inclusion of principal components and individual features in the same model, the authors' response did not actually address my concern. The authors pointed to the pre-registration where they anticipated multi collinearity would be an issue, which based on the manuscript, is the rationale for implementing PCA. However, it does not address the concern that there are essentially overlapping data in the same model. As the authors pointed out, it may not be an issue "unless the assumptions are violated (e.g., multicollinearity)". I can't easily find anywhere in the manuscript whether this assumption was tested. This would need to be tested and reported.

We thank the reviewer for their comment. As reported we looked at the correlations between independent variables, and set a was highly stringent bench mark (r<.5). Correlation matrices are available in the main text and supplementary materials.

Of course, if a variable was inserted in the principal component analysis, then this variable was not reinserted in the multivariate statistical methods. In the latest version of the paper we inserted the principal component loading which allows for easier verification of this (table 3, copies above in responses to reviewer #1).

Following the reviewers comments, we have run the VIF() R function on the models reported in the main text. This indicated that the variance inflation factor was low (all below 2.1, when cut offs are usually placed at the values of 5 and 10). We have added the following to the main text.

The following refers to the analysis regarding the differences between mind wandering and dream reports.

Lines 812-813

> We found that variance inflation factors (using the R function VIF()) of the independent variables were all below 1.5 indicating low correlation between these.

We found similar results for the analyses referring to self reported depression and anxiety.

Lines 878-879

Variance inflation factors indicated low collinearity (all below the value of 2.1)

New Analyses
Since the authors are reporting the relationship between word repetition in dream reports and the PHQ9 to be 'particularly stable', I once again suggest the authors try to explain this result in the Discussion; this seems to have been missed in the response document. Specifically, I think it would be important to explain, or at least attempt at an explanation for, why higher word repetition in dream (but not mind wandering) was related to depression.

We would like to apologise for missing this in the previous round of reviews. Following reviewer #1's comment above, we have removed any discussion in relation to our attempt to elucidate particularly stable and consistent results, and exclusively report the ordinal logistic regressions with repeated measures averaged. Here we see that PC1 of mind wandering reports were significantly related to GAD symptoms. Please see the responses above for more details. Following this we have changed the abstract in the following manner:

Lines 37-39

Finally, we find that word centrality (i.e., lower lexical diversity) in dream and mind wandering reports are predictive of depression and anxiety symptoms respectively.

In relation to the reviewers comment, we have also modified the discussion regarding these results, trying to offer plausible interpretation for the different relationships mind wandering and dream reports show with self reported mental health symptoms.

Lines 568-581

Although cognitive symptoms are documented in anxiety and depression, we control for the verbosity of the reports in our analyses, and this variable did not show significant effects in contrast to our predictions. Of interest is the difference between dream and mind wandering reports in that they relate to depression and anxiety symptoms respectively. Having reduced repeated measures by averaging metrics within participants, this discrepancy may be partly due to reduced statistical power. On the other hand, similarly lexical diversity in mind wandering may be related to repetitive thoughts and worries, which may be particularly prominent during mind wandering and less noticeable in dream recall. In fact, tendentially, anxiety is particularly characterised by worry-driven repetitive thoughts about future situations (Brewin et al., 2010), which may have been preferentially expressed in mind wandering reports. Beyond cognitive symptoms noted above (e.g., shorted phrases and lower lexical diversity) during dream recall, higher word recurrence in dream reports may reflect self-centred processing (e.g., repetition of "I") or a ruminative focus on specific themes. Recurring dreams, in fact, are often characterised by negative content[88]. Post-traumatic clinical presentations may offer a stark example of this phenomena. In future research, parsing out the influence that different symptoms of depression may have on naturalistic reports may provide a thought-provoking elaboration on our findings.

Limitation Section
Minor point: "The writing of a verbal report of an experience (such as mind-

wandering and dreaming) poses the problem that it requires the recall and then a verbal description of a qualitative experience. Such an issue relates to any verbal report." I believe this last statement is not correct. Verbal report in the context of a think aloud would circumvent the issue of recall, which may not be accurate.

We have removed the phrase "Such an issue relates to any verbal report".

REFERENCES

1. Mota, N. B. *et al.* Speech as a Graph: Developmental Perspectives on the Organization of Spoken Language. *Biol. Psychiatry Cogn. Neurosci. Neuroimaging* **8**, 985–993 (2023).

2. Li, H. & Hills, T. Time, valence, and imagination: a comparative study of thoughts in restricted and unrestricted mind wandering. *Psychol. Res.* **88**, 1510–1521 (2024).

3. James, W. The Principles of Psychology. 4004 (1890).

4. Mota, N. B. *et al.* Speech graphs provide a quantitative measure of thought disorder in psychosis. *PLoS One* **7**, 1–9 (2012).

5. Mota, N. B. *et al.* A Naturalistic Assessment of the Organization of Children's Memories Predicts Cognitive Functioning and Reading Ability. *Mind, Brain, Educ.* **10**, 184–195 (2016).

6. Mota, N. B. *et al.* Verbal Short-Term Memory Underlies Typical Development of "Thought Organization" Measured as Speech Connectedness. *Mind, Brain, Educ.* **14**, 51–60 (2020).

7. Mota, N. B., Furtado, R., Maia, P. P. C., Copelli, M. & Ribeiro, S. Graph analysis of dream reports is especially informative about psychosis. *Sci. Rep.* **4**, 1–7 (2014).

8. Mota, N. B., Copelli, M. & Ribeiro, S. Thought disorder measured as random speech structure classifies negative symptoms and schizophrenia diagnosis 6 months in advance. *npj Schizophr.* **3**, 1–10 (2017).

9. Malcorra, B. *et al.* Speech connectedness predicts reading performance three months in advance: a longitudinal experiment. *npj Sci. Learn.* **9**, 35 (2024).

10. Malcorra, B. L. C. *et al.* Low speech connectedness in alzheimer's disease is associated with poorer semantic memory performance. *J. Alzheimer's Dis.* **82**, 905–912 (2021).

11. Bertola, L. *et al.* Graph analysis of verbal fluency test discriminate between patients with Alzheimer's disease, Mild Cognitive Impairment and normal elderly controls. *Front. Aging Neurosci.* **6**, 1–10 (2014).

12. Martin, J. M. *et al.* Structural differences between REM and non- REM dream reports assessed by graph analysis. *PLoS One* **15**, 1–20 (2020).

13. Mota, N. B. *et al.* Dreaming during the Covid-19 pandemic: Computational assessment of dream reports reveals mental suffering related to fear of contagion. *PLoS One* **15**, 1–19 (2020).

14. Christoff, K., Irving, Z. C., Fox, K., Spreng, R. N. & Andrews-Hanna, Jessica, R. Mind-wandering as spontaneous thought: a dynamic framework. *Nat. Rev. Neurosci.* **17**, 1–44 (2016).

15. Fox, K. C. R., Nijeboer, S., Solomonova, E., Domhoff, G. W. & Christoff, K. Dreaming as mind wandering: Evidence from functional neuroimaging and first-person content reports. *Front. Hum. Neurosci.* **7**, 1–18 (2013).

16. Sikka, P. *et al.* COVID-19 on Mind: DailyWorry About the Coronavirus Is Linked to Negative Affect Experienced During Mind-Wandering and Dreaming. *Emotion* (2023) doi:10.1037/emo0001255.

17. Killingsworth, M. A. & Gilbert, D. T. A wandering mind is an unhappy mind. *Science (80-.).* **330**, 932 (2010).

18. Poerio, G. L., Totterdell, P. & Miles, E. Mind-wandering and negative mood: Does one thing really lead to another? *Conscious. Cogn.* **22**, 1412–1421 (2013).

19. Souza, G. V., Mota, N. B., Barros, A. K. & Ribeiro, S. Visuoaffective day residue in hypnagogia involves sequential bihemispheric interactions between cortical, subcortical, and cerebellar structures. *Commun. Biol.* **8**, 1–9 (2025).

20. Mota, N. B. *et al.* Attenuated symptoms are associated with connectedness and emotional expression in narratives based on emotional pictures in a Brazilian clinical high-risk cohort. *Psychiatry Res.* **348**, (2025).

21. Brewin, C. R., Gregory, J. D., Lipton, M. & Burgess, N. Intrusive Images in Psychological Disorders: Characteristics, Neural Mechanisms, and Treatment Implications. *Psychol. Rev.* **117**, 210–232 (2010).

22. Coppola, P. *et al.* The complexity of the stream of consciousness. *Commun. Biol.* **5**, 1–15 (2022).

23. Mota, N. B. *et al.* Speech graphs provide a quantitative measure of thought disorder in psychosis. *PLoS One* **7**, 1–9 (2012).

24. Mota, N. B. *et al.* Imagetic and affective measures of memory reverberation diverge at sleep onset in association with theta rhythm. *Neuroimage* **264**, (2022).

25. Gomes, M. *et al.* Speech graph analysis in obsessive-compulsive disorder: The relevance of dream reports. *J. Psychiatr. Res.* **161**, 358–363 (2023).

26. Chaves, M. F., Rodrigues, C., Ribeiro, S., Mota, N. B. & Copelli, M. Grammatical impairment in schizophrenia: An exploratory study of the pronominal and sentential domains. *PLoS One* **18**, (2023).

27. Marti, J. M. *et al.* Structural differences between REM and non- REM dream reports assessed by graph analysis. *PLoS One* **15**, (2020).

28. Lee, U. *et al.* Brain Networks Maintain a Scale-free Organization across Consciousness, Anesthesia, and Recovery. *Anesthesiology* **113**, 1081–1091 (2010).

29. Hirnstein, M., Stuebs, J., Moè, A. & Hausmann, M. Sex/Gender Differences in Verbal Fluency and Verbal-Episodic Memory: A Meta-Analysis. *Perspect. Psychol. Sci.* **18**, 67–90 (2023).

30. Cappadona, R. *et al.* Sleep, dreams, nightmares, and sex-related differences: A narrative review. *Eur. Rev. Med. Pharmacol. Sci.* **25**, 3054–3065 (2021).

31. Li, C., Wang, H., De Haan, W., Stam, C. J. & Van Mieghem, P. The correlation of metrics in complex networks with applications in functional brain networks. *J. Stat. Mech. Theory Exp.* **2011**, (2011).

We would like to thank the reviewers for their extended effort in improving this article.

Following a mandatory editorial revision request, we have moved the methods section to be between the introduction and the results. Please note we were also requested to remove methdological detail from the results section.

To facilitate the review process, we have highlighted the reviewer comments in BOLD. We have indented quotations from the paper and highlighted changes in red.
Line numbers are from the tracked version of the manuscript.

Reviewer #1 (Remarks to the Author):

I appreciate the chance to re-review this paper. The authors have once again made substantial changes that have improve the manuscript overall. I personally still have some concerns about the manuscript being published in its current form - partly due to some of the issues not being addressed in a deep and thoughtful way that I have already pointed out a few times. That said, there is still a lot of positive about the paper and so I will defer to the Editor and other reviewer's on the publication readiness of this manuscript.

1.1 In both of my previous reviews, I pointed out that the authors have not done a theoretical deep dive to formulate their key research questions and contributions. This is still problematic to me in a few ways. First, the authors would have a much stronger paper if they made the theoretical and/or methodological contributions very clear. There is some good interpretation in the discussion section, but it focuses on explaining individual findings. In my last review, I asked the authors to more deeply connect their work to the previous literature and theory. They authors indeed changed the framing of their Intro, but in once again a relatively "light touch way."

1.1.1. There are several example of this, some are pretty more shallow ones and others are deeper. I asked for a deeper examination without necessarily pointing out issues in great detail, but here I will point a few specifics to hopefully provide more clarity. One that I (and other reviewers) have already mentioned is the treatment and construct definition of mind wandering and spontaneous thought, as well as whether the task actually elicits this phenomenon. The authors still do not define this clearly and do not handle it in a nuanced way. There is an assumption that all thought that arises in this paradigm is mind wandering, which is not at all what the theory they cite (Christoff et al.) would suggest to be consistent with mind wandering. The Dynamic Framework would suggest it *could* arise in this context but by no means will. It seems to me the authors are comparing waking cognition and dreaming — this is actually a fair and interesting comparison, but to extrapolate this to "mind wandering" or spontaneity is

a confusing leap regardless of what theory is implicated (even Seli et al's family resemblances theory would not agree, I don't think).

We thank the reviewer for their continued effort in improving the paper. We appreciate that the spontaneous thoughts framework might not adequately capture our operationalisation. We have therefore removed this as the theoretical definition of mind wandering throughout the paper.
In accordance with Seli et al., 2018, clear and conclusive conceptual boundaries for mind wandering are lacking. We cite directly from their paper.

> "Indeed, absent a classical definition, there are no necessary and sufficient defining features with which to include or exclude exemplars from the category."

> "As multiple definitions of mind-wandering already exist—some of which are mutually exclusive—this seems a fruitless endeavor:"

Given that no universally acceptable definition for mind wandering, and a thorough review of the conceptual debate is beyond the scope of this manuscript, we have opted to follow the reviewer's suggestion to clearly define mind wandering in a way that fits our research paradigm. As Seli and others note mind wandering can be intentional and unguided.
In response to the reviewers comments, we implement Seli et al.'s advice.
Below we quoote their suggestion from thier 2018 paper.

> "Here, we conceptualized mind-wandering as ________, and operationally defined it for our participants as ________." Critically, this approach allows researchers the freedom to study whatever features of mind-wandering they wish, while providing needed specificity about aspects of the experience being explored"

Here is what we insert in the introduction
[Lines 112-118]

> We define mind wandering as self-generated, intentional or unintentional unguided thought that can be either stimulus-dependent or stimulus-independent, and comprises all inner experiences (e.g., thoughts, images, memories, plans). Participants were instructed to find a distraction-free environment, let their minds wander and write a report of the content of their mind wandering immediately afterwards.

The distraction free environment reduced the possibility of stimulus driven thought but we cannot guarantee this. Either way, mind wandering in some definitions may occur following a stimulus (Seli et al., 2018).
We again quote from this paper:

> "Consider, for example, the scenario in which you are eating dinner with your family and your thoughts drift to an argument you just had with your sibling, who is seated next to you. Such stimulus-associated thoughts would qualify as "mind-wandering" to many people. Yet, because these thoughts were triggered by (and continue to feature) an environmental stimulus, the SIT definition rejects them as mind-wandering."

We also note the following quote from the paper, which describes our study quite well.

> “Collecting large corpuses of open-ended reports would allow text-mining techniques to reveal novel and potentially unknown dimensions of the mind-wandering state.”

1.1.2 I am not an expert in graph theory, but the deep contribution is still not clear in relation to this; what do we get by combining that literature with this question that we do not already know? The authors seems to still base their investigation on the idea that we need to be able to understand “the organisation of narratives when recalling introspective and personal memories from these different physiological states of consciousness, thus characterising the continuity and divergence of dreams and mind-wandering reports.” Yet I am still struggling to see how the organization of these narratives will tell us more than we know from other NLP and phenomenological investigations — that they are different in key ways. Why does the organization tell us something specific and how does that relate to theory? I continue to believe this is an answer here, but I also think a stronger case should be made.

We note that this specific implementation of G.T.A., with over a decade of research, is a specific instance of the wide spectrum of NLP, which highlights specific aspects. As reported by the reviewers, there are strong difference with other NLP approaches (e.g., stop words). For example the NLP paper cited below uses a completely different technique (semantic embeddings), with different sensitivity and focuses.
Of note, despite the preregistration, this is a data driven approach, which can be considered part of “complexity science”. It is appropriate for large and complex datasets such as this one. We refer the reader to extensive writing in the paper and previous responses.

Nonetheless, we add [lines 207-210].

> With this technique we aim to explore word recurrence patterns in natural writing with little preprocessing of the data, in accordance with the framework described above (see Mota et al., 2023 for further info.)

Our purpose was not to test any particular flasifiable statement of a theory with an appropriate experimental design. Nonetheless, we have related our analyses to the statements in the literature that MW and dreaming are continuous (H1), can reflect Individual differences (H2), and can be related to mental health (H3). Our intent was to provide rigorous empirical evidence for or against these debates. (Why we have not particularly been explicit with the dream and MW debate is discussed below. We now do discuss it in the text, with rigorously stated caveats that are required.)

Our contribution relates to these debates. As the editor notes, we do not report any revolutionary results. However, we report results that are rigorous and will be useful as incremental evidence in the multidisciplinary long term direction of the relevant academic fields.

We cite specific sections of the paper explaining how we contribute to the litterature.
[Lines 692-695]:

> Dreams have been described as an intensified form of mind-wandering[22–24]. On one hand, dreaming and mind-wandering are considered continuous[16–19]. On the other hand, there seem to be consistent differences between these experiences[24,30,38,63,79]. We observed differences in the structure of the written reports of these experiences[30].

After this section there is a method specific summary of the results and after that, further interpretations.

Please also see in relation to H2:
[Lines 772-773]

> This method is sensitive to stable characteristics that distinguish individuals in writing dream and mind-wandering reports

Again, after this section, we interpret how and why we find such stable characteristics.

In relation to H3:
[Lines 794-796]

> We found that graph theory analysis of written reports of mind wandering and dreams may relate to mental health symptoms, in accordance with previous literature using this method[7,10,11].

Following this we summarise the results and interpret their meaning.

1.2 Another pretty clear issue is that the authors motivate parts of their individual differences section with the idea that "studies show that mind-wandering is often related to negative affect." This is, once again, relatively shallow treatment of the literature. The two papers cited on this claim do not even seem relate to the mind wandering literature directly, despite a rich literature that exists. The idea that mind wandering is related to negative affect is actually quite complex and nuanced (there is a recent review on this, as well as other work in this field). Some measures of mind wandering actually positively relate to affect. Again, the core theory cited here would suggest that mind wandering is actually different from rumination and anxiety, so this where the background and framing seems a bit off.

This is an important point that the reviewer has made. We have modified the relevant paragraphs in the following manner to reflect that mind wandering can relate to positive affect:

[Lines 143-144]

Similarly, studies show that mind-wandering is can be related to both[1,2] as well as negative affect[49,50]. There have been suggestions that mind wandering may be related to rumination[51,52].

[Lines 181-182]

Mind wandering, although sometimes associated with positive affect[1,2] has also been related to negative mood, and has been proposed to be potentially be related to anxiety and depression[3–7].

The title of the papers cited in the reviewer's comment are as follows:
Killingsworth, M. A. & Gilbert, D. T. A wandering mind is an unhappy mind. *Science* (2010).

Mind-wandering and negative mood: Does one thing really lead to another? *Conscious. Cogn.* (2013).

1.3. As for the shallow ones: One is that the paper opens talking about tracking psychological states on social media, which seems quite distant from the literature/constructs of interest here.

We have removed the relevant section.

1.4 The authors have attempted to clarify the fact that one of the PCs accounted for over 90% of the variance. I guess this is a critical point where I'm not sure how to interpret the PC and how it relates to the core research questions and predictions. I am also not sure why: "the variables included were word count, max betweenness centrality, assortativity, PC-1 Centrality, PC2-Diameter, clustering coefficient, country, gender and age." I asked about why certain variables were included last time, but it's still a bit confusing to me. Why did the authors do the PC if the ultimate approach was to pick particular variables — why not just include those variables to begin with instead of their loadings? This is especially true since this part was not pre-registered.

There are several pages in previous responses, supplementary materials, as well as various sections in the paper explaining this. Nonetheless, we highlight some points succinctly to clarify the concerns the reviewer raises.

- The use of PCA was necessary to avoid collinearity and follow statistical assumptions.
-This was also necessary to unique predictive value of specific measures. Admitting we could ignore statistical assumptions, If different metrics are correlated, one cannot interpret results in an appropriately accurate manner (i.e., how one specific independent variable uniquely relates to the dependent variable).
-The other variables that were not included in the PCA, but when they are statistically independent from other variables. Hence issues of colinearity and interpretability are not applicable. We only inserted highly correlated variables in the PCA.

- As noted, although results of the principal component analysis are used in the other analyses for the above stated reasons, these are distinct analyses.
-This was in fact specified in the preregistration. We quote directly:

> "Of note is that different network properties (as measured by graph theory analyses) can be highly correlated (Li, Wang, De Haan, Stam, & Van Mieghem,2011). Such a correlational structure needs to be resolved to provide strong interpretations. The large amount of data in this dataset provides the ideal platform onto which explore the relationship between different metrics in these types of networks (i.e., built from text, see
> (Mota et al., 2014)
> Thus, although we have predictions as to the hypotheses (see section 1), we need to resolve the correlational structure of different network metrics to increase interpretability, power of statistical models, parsimony of results and subsequent clinically-relevant implementations.
> These measures will be reduced to fewer variables via principal component analysis. In the following paragraph, see apriori preliminary suggestions of the correlational structure. The question to be resolved is the extent of independent variance between measures"

1.5 I would have expected the authors to integrate their core hypothesis into their literature review. I agree with them that the review is useful, but it is still very descriptive in nature without being integrated with theory. For example, what does hypothesis 2 telling us?

We phrased the paper in terms of mind wandering and dream reports being related to affect and individual differences.
As stated above, our analyses are not designed to experimentally test specific statements within a theory, and hence we cannot make any statements to this effect. Furthermore, H2, beyond potentially being interesting to a researcher, might be considered a methodological quality check. It is difficult to imagine a theory of mind wandering that might propose that the relevant phenomena are not at all related to individual differences. Hypothesis two tests if there is consistency within individuals, thus revealing that the results are in consonance with what we might expect.

Importantly, we framed the paper in terms of the dreams and MW being continuous, a notion which is proposed in the spontaneous thoughts framework.

We do not make a final pronouncement on this issue as
1) There are some significant differences.
2) Distributions of variables are overlapping between MW and dreams.
3) Even in the unlikely scenario that distributions are not overlapping, the present design could not make any final claims.

Following the reviewers comments we note this explicitly.

[Lines 692-695]

Although we found differences, we do not outright reject the proposal that dreams and mind wandering are continuous[8], given that distributions were overlapping (Fig. 2), the method and design of this study are not appropriate to make such claims, and only some variables differed between the conditions.

We interpret and relate our results (H1, H2 and H3) to the literature extensively in the discussion section. Please also see a statement of our hypotheses at the end of all the relevant introduction sections
[Lines 108, 155, 193]

1.6 I am still concerned that the authors don't account for the methods and how they contribute to the findings themselves. This is a retrospective account, which is essentially a recall. The mind wandering reports happened immediately after a 10 minute period, whereas the dreams are not necessarily immediate and may have more decay and less specificity as a result.

We have discussed this and made this explicit since the first version of the article, and have added more in this respect in this over the various versions despite the consideration not been explicitly mentioned by the reviewers. This is indeed important for interpretation and is disucssed throughout the article. Following the reviewer's comment, we also now include further information still in the abstract, introduction and discussion.

[Lines 36-38]

Dream reports, which rely on recall over longer periods of time, tend to have more word repetitions, local cliques, and global integration, comparatively indicating a small world structure.

[Lines 81-83]

They both tend to arise spontaneously and often reflect one's current concerns[9] and mentation, and rely on mnemonic information (although to different degrees) or provide a simulation of reality[3,4,10,11].

[Lines 756-757]

Neuroimaging studies describe differences between dreams and mind wandering[12] in regions that are fundamental to consciousness[13]. In fact, dream reports, compared to mind-wandering reports, are characterised by recall of information between starkly different states of consciousness[25]

We note this in the limitations section.

[Lines 877-881]

Although it is possible (although not in such a large and naturalistic study) in principle to wake participants up and obtain an immediate recall of dreams as well as obtain live mind wandering reports, this would hardly make for naturalistic conditions, and beyond this, immediate reporting is likely to change the nature of the experience itself (a well known, difficult and arguably unresolved issue in consciousness science[14–16]).

1.7. The authors mention this in the verbosity section of the discussion, but I'm not entirely convinced that this is not actually a major factor. For example, if an only a subset of the mind wandering results were compared — specifically ones that are less verbose — were compared, do results totally change? Or perhaps after controlling for length and episodic specificity in the other models? I would love to see some action to address this rather than simply saying it is an issue that can't be addressed given that the claims from the paper rest on the methodological soundness.

We do not argue this is not a major factor. Verbosity is in fact a major factor, and we have isolated in a methodologically sound manner, as demonstrated by the various sanity checks we employ.

We avoid copying our extensive previous responses and the various secitons of the paper for brevity, although these can be found in previous responses. Nonetheless, for the reviewer's benefit, we highlight:

- Verbosity is related to G.T.A. properties, unless you control for this appropriately. This is well known and the intention to use the sliding window approach for this is noted in the pre-registration.
- We control for this purposefully and successfully using the sliding window approach.
- The effectiveness of this is demonstrated with the very low correlation with other metrics (if not word recurrence will always correlate with the length of the text).
- We also insert verbosity as a covariate in the statistics. The lack of violation of statistical assumptions (explicitly tested) also shows that this was corrected effectively.
- Verbosity is different between MW and Dreams as discussed in the main text (and, notably, independently so from other features we analyse, see the response in relation to the PCA above).

1.8. I would think the authors might have also found some other relevant literature in their attempt to review related work, particularly comparisons of sleep and wake condition (or specifically mind wandering). For example, Kirberg et al. (2025), Mallet et al. (2025), and Gross et al. (2021), to name a few (some even including NLP, such as Mallet).

We thank the reviewer for pointing out these relevant references and have added them. We note that most of the studies here cited were not published when we submitted this article (in the summer of 2024).

Kirberg et al. (2025): of note is that this has similar issues to our design, however, they do not discuss the issues with recall (which may be more substantial in their design) in any depth.

Mallet et al., (2025): This uses a completely unrelated computational methodology to ours (as they primarily use semantic embeddings which do not feature at all in our analysis).
We have inserted it into our previously mentioned future directions, as it would be possible in principal and interesting to undertake work to integrate these two approaches.

Gross et al., (2021): Similarly to our results, these also highlight differences between dream and MW. This has been also added.

I appreciate some of the additional details on analyses. I'm still a bit confused about why sometimes a logistic regression was used when I assume that the PCs and PHq9 are not binary variables?

This was not done using binary variables. We used ordinally ordered categorical variables (in an ordinal logistic regression). We used the official cut offs that are ubiquitously used in everyday clinical practice in many different countries.
We originally did, in fact, report different ways of treating this variable to show that results reproduce (e.g., non parametric, continuous). But following the reviewers' previous comments we have removed this.

Reviewer #2 (Remarks to the Author):

2 I commend the authors for implementing sophisticated analyses on a complex dataset with the aim to address an interesting question. Unfortunately, I still see that they have not quite fully addressed some of the core issues raised by myself and the other reviewer, which mostly concerns the methods and theoretical/conceptual aspects.

2.1 For example, I still struggle to interpret some of the results involving graph theory metrics in the context of the data. To address this comment, the authors provided examples of mind wandering and dream reports in Figure 4 and the actual verbalized data, which is helpful. However, it does not directly help with interpreting the results. Given that I have already made this comment twice, I'm not sure that an additional round of comment would lead to a different outcome.

We appreciate the reviewers continuous effort in helping us improve the manuscript.
We also direct the reviewer to the supplementary materials that we created following their comment.
Graph theory analysis is considered part of the so called "complexity science". As such it is used to tease out features that are not apparent from a human perspective. Nonetheless, we have attempted to give numerous alternative pictorial and conceptual descriptions of the variables. We hope that these will help any reader form their own interpretations. If ever, any particularly motivated researcher may study the mathematics underlying this method directly. Either way, the analyses were conducted rigorously, and we confidently show that this method is sensitive to individual differences, and can show variation that is related to affect.

2.2. I recognize the authors spent a huge amount of effort to addressing comments during both rounds and I appreciate those efforts. Perhaps this second submission is reflective of the most that can be offered in terms of providing theoretical grounds and offering meaningful interpretations.

We thank the reviewer for recognising the work.
We have added additional theoretical interpretations in response to the reviews above. We hope that this has improved the manuscript further in their view.

* TRANSPARENT PEER REVIEW: Communications Psychology uses a transparent peer review system. This means that we publish the editorial decision letters including Reviewers' comments to the authors and the author rebuttal letters online as a supplementary peer review file. However, on author request, confidential information and data can be removed from the published reviewer reports and rebuttal letters prior to publication. If your manuscript has been previously reviewed at another journal, those Reviewers' comments would not form part of the published peer review file.

** Visit Nature Research's author and referees' website at www.nature.com/authors for information about policies, services and author benefits**

References

1. Welz, A., Reinhard, I., Alpers, G. W. & Kuehner, C. Happy Thoughts: Mind Wandering Affects Mood in Daily Life. *Mindfulness (N Y).* **9**, 332–343 (2018).
2. Gross, M. E. *et al.* Comparing the phenomenological qualities of stimulus-independent thought, stimulus-dependent thought and dreams using experience sampling: Phenomenology of SIT, SDT, and dreams. *Philosophical Transactions of the Royal Society B: Biological Sciences* **376**, (2021).
3. Christoff, K., Irving, Z. C., Fox, K., Spreng, R. N. & Andrews-Hanna, Jessica, R. Mind-wandering as spontaneous thought: a dynamic framework. *Nat. Rev. Neurosci.* **17**, 1–44 (2016).
4. Fox, K. C. R., Nijeboer, S., Solomonova, E., Domhoff, G. W. & Christoff, K. Dreaming as mind wandering: Evidence from functional neuroimaging and first-person content reports. *Front. Hum. Neurosci.* **7**, 1–18 (2013).
5. Sikka, P. *et al.* COVID-19 on Mind: DailyWorry About the Coronavirus Is Linked to Negative Affect Experienced During Mind-Wandering and Dreaming. *Emotion* https://doi.org/10.1037/emo0001255 (2023) doi:10.1037/emo0001255.
6. Killingsworth, M. A. & Gilbert, D. T. A wandering mind is an unhappy mind. *Science (1979).* **330**, 932 (2010).

7. Poerio, G. L., Totterdell, P. & Miles, E. Mind-wandering and negative mood: Does one thing really lead to another? *Conscious. Cogn.* **22**, 1412–1421 (2013).
8. Christoff, K., Irving, Z. C., Fox, K., Spreng, R. N. & Andrews-Hanna, Jessica, R. Mind-wandering as spontaneous thought: a dynamic framework. *Nat. Rev. Neurosci.* **17**, 1–44 (2016).
9. Domhoff, G. W. *The Emergence of Dreaming: Mind-Wandering, Embodied Simulation, and the Default Network. The emergence of dreaming: Mind-wandering, embodied simulation, and the default network.* (Oxford University Press, New York, NY, US, 2018).
10. Windt, J. M. Dreams and Dreaming. *The Stanford Encyclopedia of Philosophy* https://plato.stanford.edu/cgi-bin/encyclopedia/archinfo.cgi?entry=dreams-dreaming (2019).
11. Windt, J. M. How deep is the rift between conscious states in sleep and wakefulness? Spontaneous experience over the sleep-wake cycle: Spontaneous experience in sleep/waking. *Philosophical Transactions of the Royal Society B: Biological Sciences* vol. 376 Preprint at https://doi.org/10.1098/rstb.2019.0696 (2021).
12. Fox, K. C. R., Nijeboer, S., Solomonova, E., Domhoff, G. W. & Christoff, K. Dreaming as mind wandering: Evidence from functional neuroimaging and first-person content reports. *Front. Hum. Neurosci.* **7**, 1–18 (2013).
13. Coppola, P. A review of the sufficient conditions for consciousness. *Neurosci. Biobehav. Rev.* **177**, (2025).
14. Duman, I. *et al.* The No-Report Paradigm: A Revolution in Consciousness Research? *Front. Hum. Neurosci.* **16**, (2022).
15. Tsuchiya, N., Wilke, M., Frässle, S. & Lamme, V. A. F. No-Report Paradigms: Extracting the True Neural Correlates of Consciousness. *Trends in Cognitive Sciences* vol. 19 757–770 Preprint at https://doi.org/10.1016/j.tics.2015.10.002 (2015).
16. Block, N. What Is Wrong with the No-Report Paradigm and How to Fix It. *Trends Cogn. Sci.* **23**, 1003–1013 (2019).
