# Supplementary material for: A graph theory approach to chart the architecture of dream and mind-wandering reports
Source: Commun Psychol. 2026 Oct 8;4:135. doi: 10.1038/s44271-026-00509-5 (PMC13650135; doi:10.1038/s44271-026-00509-5)
Supplement: Supplementary file 2 [file 44271_2026_509_MOESM2_ESM.pdf]

Supplementary Information

The architecture of spontaneous thoughts and experiences: a graph theory approach.

Supplementary Material 1

List of the NetworkX functions used.

Detailed documentation is available for each of these at https://networkx.org/ (code created on 05-2023).

Where possible a directed weighted network was used. When the specific function required undirected nertwork we symmetrised the matrix by using nx.Graph.to_undirected(). The matrices were transformed into the right format by using nx.from_numpy_matrix().

Table S1. Python and Network X functions used to calculate graph theory metrics with notes as to additional processing as well as whether it was included after (post-hoc) the registration

Graph theory metric	Python NetworkX function	Notes
**Diameter**	nx.diameter()	Note, this was not calculated if the graph was not fully connected (evaluated via nx.is_strongly_connected()) – post-hoc inclusion
**Density**	nx.density()	
**Number of nodes**	nx.number_of_nodes()	post-hoc inclusion
**Number of loops**	nx.number_of_selfloops()	Note, undirected graph used here.
**Assortativity**	nx.degree_assortativity_coefficient()	
**Clustering Coefficient**	nx.average_clustering()	
**Mean centrality**	stat.mean(nx.degree_centrality().values())	
**Maximum centrality**	max(nx.degree_centrality().values())	

**Hierarchy**	nx.flow_hierarchy()	
**Modularity**	x.community.modularity(, nx.community.greedy_modularity _communities())	
**Number of Connected components**	nx.number_connected_componen ts()	Undirected graph used
**Number of cycles**	len(nx.cycle_basis())	Undirected graph used, post-hoc inclusion
**Number of Bridges**	len(list(nx.bridges()))	Undirected graph used, post-hoc inclusion
**Largest connected component**	max(max(nx.connected_compone nts(), key=len))	Undirected graph used. Note this is not the case in the Speechgraphs software (Mota et al., 2012). This algorithm requires undirected inputs.
**Largest strongly connected component**	max(max(nx.strongly_connected_ components(), key=len))	Note, in comparison to the speech graphs software (Mota et al., 2012, 2014), this used the Nuutila's modification. https://networkx.org/.
**Mean betweenness centrality**	stat.mean(nx.betweenness_centra lity().values())	
**Maximum betweenness centrality**	max(nx.betweenness_centrality(). values())	post-hoc inclusion
**Average shortest path length**	nx.average_shortest_path_length( )	Note, calculated when network was fully connected. This was not inserted in the principal component analysis as highly related to diameter and mean betweenness centrality

We engaged in pre-study interpretation of graph theory metrics to guide selection. This, was partially elaborated in the preregistration (https://osf.io/arx2h), as well as in previous studies ((Mota, Furtado,

Maia, Copelli, & Ribeiro, 2014)). Together, these texts guided the selection of graph theory properties that was calculated in network and then reduced via principle component analyses (S2).

Of note is that the network X toolbox did not have an implementation of participation coefficient.

Supplementary Material 2

Before inserting the chosen variables into the principal component analysis (PCA), we ensured that the variables had normal distributions.

We found that average clustering coefficient, Diameter, Mean betweenness centrality, number of bridges, and word count all had high levels of skewness (as evaluated with the skewness function in R). These were successfully transformed with a log transformation (natural logarithm, default in R) and made to have a skewness value below 1. Of note, given the presence of zeros in the clustering coefficient variable, and the subsequent impossibility to perform a log transform, 0.1 was added to all scores.

GAD-7 and PHQ-9 had high skewness, but other than being ordinal scales, transformations did not yield good results.

We then standardized all variables to be inputted in the PCA.

Figure S1- Correlational structure of graph theory variables.

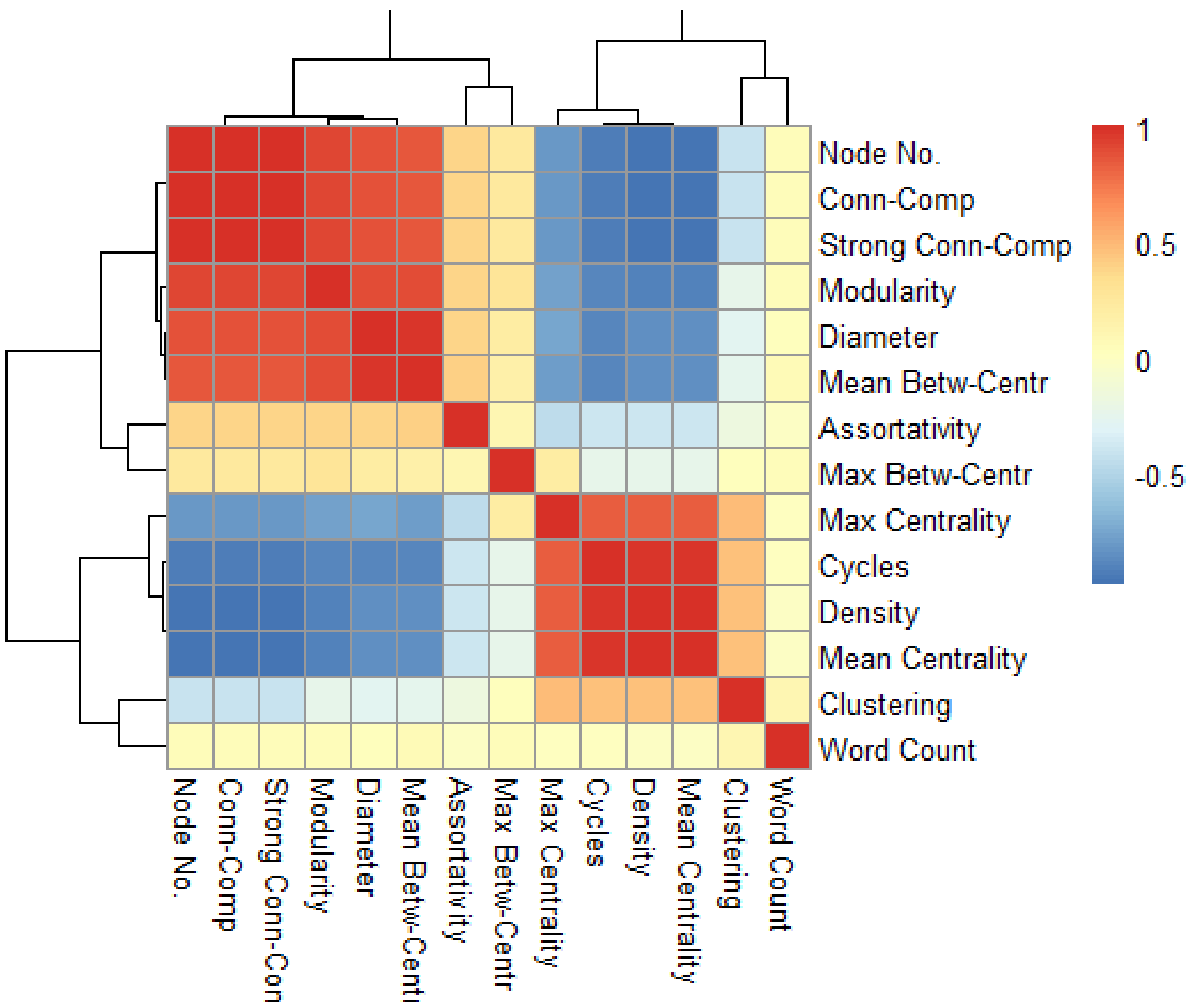

Of these variables, only the most highly correlated were inserted in the PCA, as the object of this was to reduce the highly correlated data into component that had sufficient independent variance to be included as covariates in a statistical model.

Percentage explained variance for PC1 was 90.98. For PC2 it was 3.94 whilst for PC3 it was 2.88

Supplementary Material 3

Supplementary material for hypothesis one. Logistic regression discerning the network structure differences between dreams and mind wandering.

Outcome measure was a dummy variable indicating whether the graph theory metrics were derived from mind wandering or dreams. We used the glmer function (family= binomial("logit")) from the lme4 package in R, with participant ID was a random effects factor.

Table S3. Logistic regression with fixed effects, estimated coefficient, standard errors and p-values.

	Coeff	Std. Error	z value	P-value
Intercept	-0.12	0.17	-0.70	0.483
PC1-Centrality	0.83	0.06	13.98	2.18e-44
PC2-Diameter	-0.20	0.05	-4.15	3.39e-05
Age	-0.05	0.07	-0.74	0.460
Country	-0.15	0.13	-1.19	0.233
Gender	0.03	0.15	0.17	0.861
Word Count	0.14	0.05	3.08	0.002
Assortativity	-0.07	0.05	-1.53	0.127
Maximum betweeness centrality	0.43	0.05	9.06	1.32e-19
Clustering coefficient	-0.15	0.05	-2.91	0.0036

Note, for ease of interpretation, figures relating to PC1 were inverted (as centrality metrics had a negative loading onto this).

Below is the anova test comparing the logistic regression with and without PC2-diameter

Table S4. Model selection for H1.

	Number parameters	AIC	BIC	CHI square	P-value
Without PC2	10	3716.2	3776.5		
With PC2	11	3700.9	3767.2	17.3	3700.9

BIC= Bayesian information criterion

AIC= Akaike information criterion

Supplementary Material 4

This shows some of the analyses performed to try to elucidate hypothesis 3 (i.e., do dream and mind wandering report structures predict GAD-7 and PHQ-9 scores).
We start with the PHQ scores, the models were without the random effects. We also present kendall's Tau correlations.
We then do the same for the GAD-7 scores. We highlight in BOLD the relevant statistics (i.e., the results deemed sufficiently robust and that are reported in the main text.)

PHQ-9 related results

Table S5. Ordinal logistic regression of dream architecture with no repeated measures (metrics averaged in a participant specific manner) and with PHQ-9 scores as the criterion variable.

	Estimate	Std. Error	z value	P-value
Word Count	-0.02	0.16	-0.14	0.88
Max betweeness centrality	0.40	0.17	2.30	0.021
Assortativity	-0.12	0.18	-0.67	0.50
**PC1-Centrality**	**-0.47**	**0.19**	**-2.48**	**0.013**
PC2-Diameter	-0.19	0.16	-1.20	0.22
Clustering	0.01	0.17	0.08	0.93
Country	-0.35	0.32	-1.11	0.26
Gender	-0.02	0.36	-0.07	0.94
**Age**	**-0.47**	**0.17**	**-2.80**	**0.005**
None|Mild	-0.88	0.43	-2.03	0.04
Mild|Moderate-Severe	0.92	0.43	2.13	0.03

Table S6. Ordinal logistic regression of MW architecture with no repeated measures (metrics averaged in a participant specific manner) and with PHQ-9 scores as the criterion variable.

	Estimate	Std. Error	z value	P-value

Word Count	-0.09	0.17	-0.54	0.58
Max betweeness centrality	-0.23	0.17	-1.37	0.17
Assortativity	0.33	0.19	1.76	0.07
PC1-Centrality	-0.22	0.23	-0.97	0.33
PC2-Diameter	-0.07	0.18	-0.42	0.67
Clustering	0.04	0.21	0.17	0.86
Country	-0.54	0.33	-1.62	0.10
Gender	-0.14	0.38	-0.37	0.71
**Age**	**-0.43**	**0.18**	**-2.43**	**0.015**
None|Mild	-1.11	0.45	-2.48	0.012
Mild|Moderate-Severe	0.70	0.44	1.58	0.11

Table S7. Kendall’s Tau Correlations between graph theory structures and PHQ-9 with repeated measures

	Dreams		Mind wandering	
	Kendalls’s Tau	P-Value	Kendalls’s Tau	P-Value
**PC1-centrality**	**-0.09**	**1.591e-07**	-0.0498	**0.0064**
PC2-Diameter	-0.04	0.018	-0.011	0.55
Max Betweeness centrality	-0.072	2.273e-05	-0.038	0.036
Assortativity	-0.044	0.009	0.030	0.097
Word Count	0.025	0.14	-0.027	0.129
Clustering Coefficient	0.071	3.52e-05	0.001	0.936

Table S8. Kendall’s Tau Correlations between graph theory structures and PHQ-9 without repeated measures (averaged in a participant specific manner)

	Dreams		Mind wandering	
	Kendalls’s Tau	P-Value	Kendalls’s Tau	P-Value
**PC1-centrality**	**-0.147**	**0.005**	-0.089	0.11
PC2-Diameter	-0.077	0.14	-0.038	0.49
Max Betweeness centrality	-0.131	0.013	-0.052	0.34
Assortativity	-0.097	0.066	0.082	0.14
Word Count	0.091	0.085	-0.010	0.84
Clustering Coefficient	0.162	0.002	-0.005	0.927

GAD-7 related results

Table S9. Ordinal logistic regression of dream architecture with no repeated measures (metrics averaged in a participant specific manner) and with GAD-7 scores as the criterion variable.

	Estimate	Std. Error	z value	P-value
Word Count	-0.02	0.17	-0.10	0.92
Max betweeness centrality	0.34	0.18	1.84	0.06
Assortativity	-0.25	0.19	-1.31	0.19
PC1-Centrality	-0.31	0.20	-1.56	0.11
PC2-Diameter	-0.04	0.17	-0.22	0.82
Clustering	0.14	0.18	0.77	0.44
Country	-0.07	0.33	-0.22	0.82
Gender	0.46	0.39	1.18	0.23
Age	-0.59	0.18	-3.30	0.0009
None|Mild	0.40	0.45	0.87	0.38
Mild|Moderate-Severe	1.94	0.48	4.08	4.56e-05

Table S10. Ordinal logistic regression of MW architecture with no repeated measures (metrics averaged in a participant specific manner) and with GAD-7 scores as the criterion variable.

	Estimate	Std. Error	z value	P-value
Word Count	-0.06	0.17	-0.37	0.71
Max betweeness centrality	0.10	0.18	0.54	0.58
Assortativity	0.30	0.20	1.51	0.13
**PC1-Centrality**	**-0.48**	**0.23**	**-2.07**	**0.038**
PC2-Diameter	-0.24	0.18	-1.34	0.18
Clustering	-0.29	0.21	-1.41	0.15
Country	-0.59	0.34	-1.75	0.08
Gender	0.33	0.41	0.80	0.42
**Age**	**-0.46**	**0.19**	**-2.40**	**0.016**
None|Mild	-0.11	0.47	-0.24	0.81
Mild|Moderate-Severe	1.44	0.48	2.97	0.002

Table S11. Kendall's Tau correlations between graph theory structures and GAD-7 with repeated measures

	Dreams		Mind wandering	
	Kendalls's Tau	P-Value	Kendalls's Tau	P-Value
PC1-centrality	**-0.078**	**5.404e-06**	**-0.063**	**0.0005**
PC2-Diameter	-0.037	0.027	-0.0019	0.914
Max Betweeness centrality	**-0.060**	**0.0004**	**-0.059**	**0.001**
Assortativity	**-0.065**	**0.0001**	-0.002	0.896
Word Count	0.0046	0.786	-0.0099	0.58
Clustering Coefficient	**0.0466**	**0.007**	-0.004	0.79

Table S12. Kendall's Tau correlations between graph theory structures and GAD-7 without repeated measures (averaged in individual specific manner)

	Dreams		Mind wandering	
	Kendalls's Tau	P-Value	Kendalls's Tau	P-Value
PC1-centrality	-0.152	0.004	-0.106	0.06
PC2-Diameter	-0.083	0.11	0.028	0.61
Max Betweeness centrality	-0.133	0.012	-0.11	0.06
Assortativity	-0.119	0.024	0.009	0.86
Word Count	0.059	0.26	0.008	0.87
Clustering Coefficient	0.16	0.003	-0.033	0.55

Supplementary Material 5

Example dream and mind wandering reports with relevant graph theory metrics as in fig 4 in the main text.

Also below each example is the plotted network structure.

Example of dream report with high PC1 and high self-reported symptoms (PC1=-3.01; PHQ = 17, GAD =11, PC2=0.36, Clustering Coefficient=0.81; Word Count=-0.24; Max Between Centrality= 0.51; Assortativity =-0.22)

I am on a guided bus tour to the Gold Coast with my partner and her son. I am not enjoying the tour, I don't know why I am there. Someone wants to organise that a few of us break away from the tour and go drinking that night, and they are trying to involve me in the plan, but I am trying to excuse myself. i explain that I used to live near the Gold Coast, so I am familiar with it and it is not new. I say that I used to always drive through the town to cross over the border, but I would never stop there. I am trying to say this while being polite and not put them off their excitement for their night out.

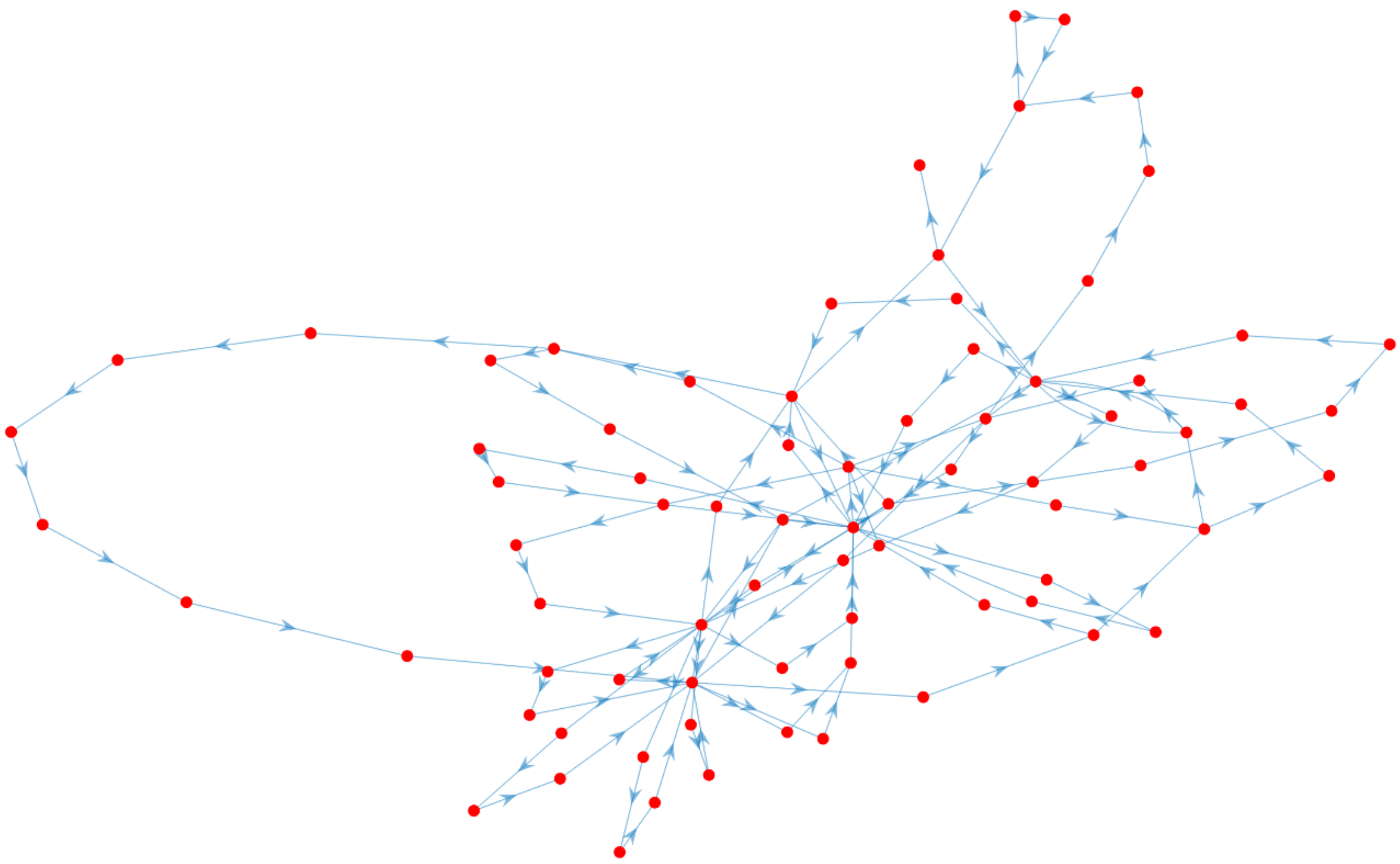

Example of mind wandering with high PC1 and high self-reported symptoms (PC1=-2.47, PHQ=17, GAD = 11, PC2=-0.91, Clustering Coefficient=0.96; Word Count=1.12; Max Between Centrality=0.55; Assortativity =-0.14)

I don't know how to do this noise of the fridge: I want to get up and turn it off I want to go to toilet it is quiet, and this is strange I want to get a cup of tea There is a high pitched noise, like the sound of crickets; I can't tell if it is in my head or outside. This happened the other night, when I thought heard a faulty alarm all night, when I went to check, it turned out it was inside my head, Then I was afraid that this might be the beginning of hearing voices. I want to do/act/move/distract myself. I think of maintenance tasks around my home a sense of 'I should' I should have a to-do list next to me to write these things down I'm sad it's more than sad, a sense of tragedy, an all-pervading tragedy of life, that is at the same time very sweet. I could easily be close to tears. at the same time, a very physical sense of 'letting go' into myself. something in me just stops I am very aware of the front of my body there is a tingling at my genitals this reminds me of a time when I used to practice and teach yoga a sense of a time when I could just stop doing things, when time was my own I anticipate cooking dinner, or going for a run I should go to the office to get some work done I should turn on my phone sadness/ I think of a time I left a partner, and her daughter's horse died shortly afterwards.

The dying horse is connected to a time I wrote poetry, it is a line in a poem I wrote after a beloved aunt's death. lost innocence/grieving the feeling has moved to my chest now the feeling has moved to my brow I think of friends who I should recommend doing this study to I think I should post something on facebook about the study, to encourage people to try it.

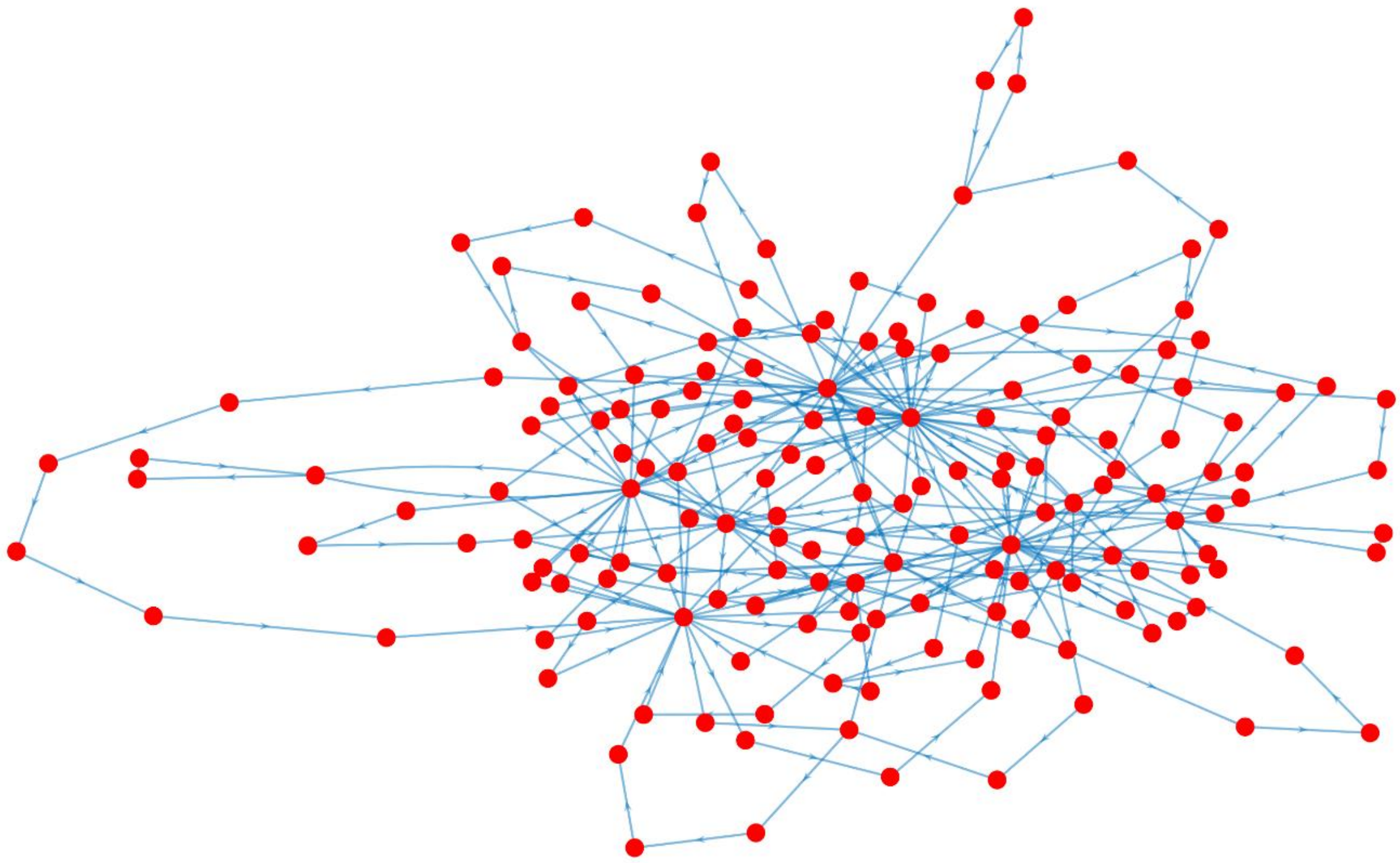

Example of dream report with low PC1 and low self-reported symptoms (PC1=2.58, PHQ = 4, GAD= 2, PC2=0.16, Clustering Coefficient=-1.31; Word Count=-0.81; Max Between Centrality=0.63; Assortativity =0.13).

I was making lists of, or counting, daily Covid cases, and sorting them into one of 3 categories. One category, I think it was children under a certain age, didn't require any restrictions, so I felt pleased if I could include a name in that list, but also anxious about getting it right. A second group was a bit ambiguous - maybe with limited restrictions, and the third group was fully restricted. The lists were piles of pieces of lined brown/manila paper, written on in pencil.

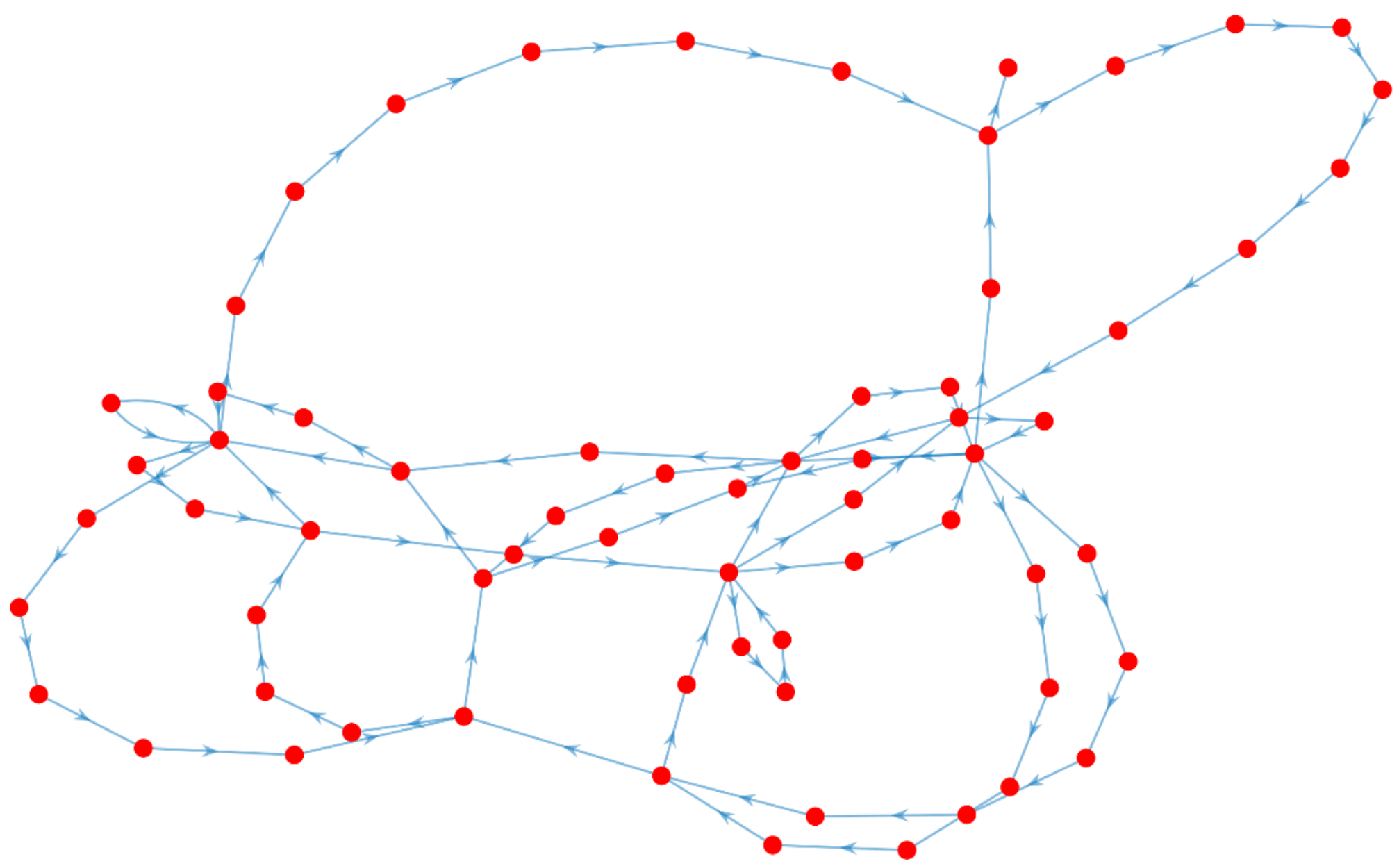

Example of mind wandering with low PC1 and low self-reported symptoms (PC1=3.07, PHQ=4, GAD=2, PC2=0.29, Clustering Coefficient=-0.128; Word Count=0.32; Max Between Centrality=0.58; Assortativity =-0.006).

Heard B mention neighbours. Set me thinking about our neighbour calling round this morning and both of us talking on the doorstep in masks. Thought about him fixing his back fence that blew down in the wind & how the wind pulled the nails out of the fence posts. It's windy again tonight. I din't like wind. I'm getting better with my clarinet lessons. Got to play high notes today. Nice to see Ann, the music teacher online. I wish Al would play his saxophone more. Will he move in with T next year. If he does who will look after our house if we travel. Thinking about the old dolls house upstairs where I am & how we moved it to make room for filming our pan pipe tune this afternoon, and when will we be able to play as a group again and wondering how Ga is going with his Parkinsons - Parkinsons, not Alzheimers, I was getting them mixed up. And Shane Jacobson at the Parkinsons walk every year & people trolling him for doing the Covid ads. Steve Moneghetti is always at the walks too. So thin. It's still windy. The wind's rattling the windows.

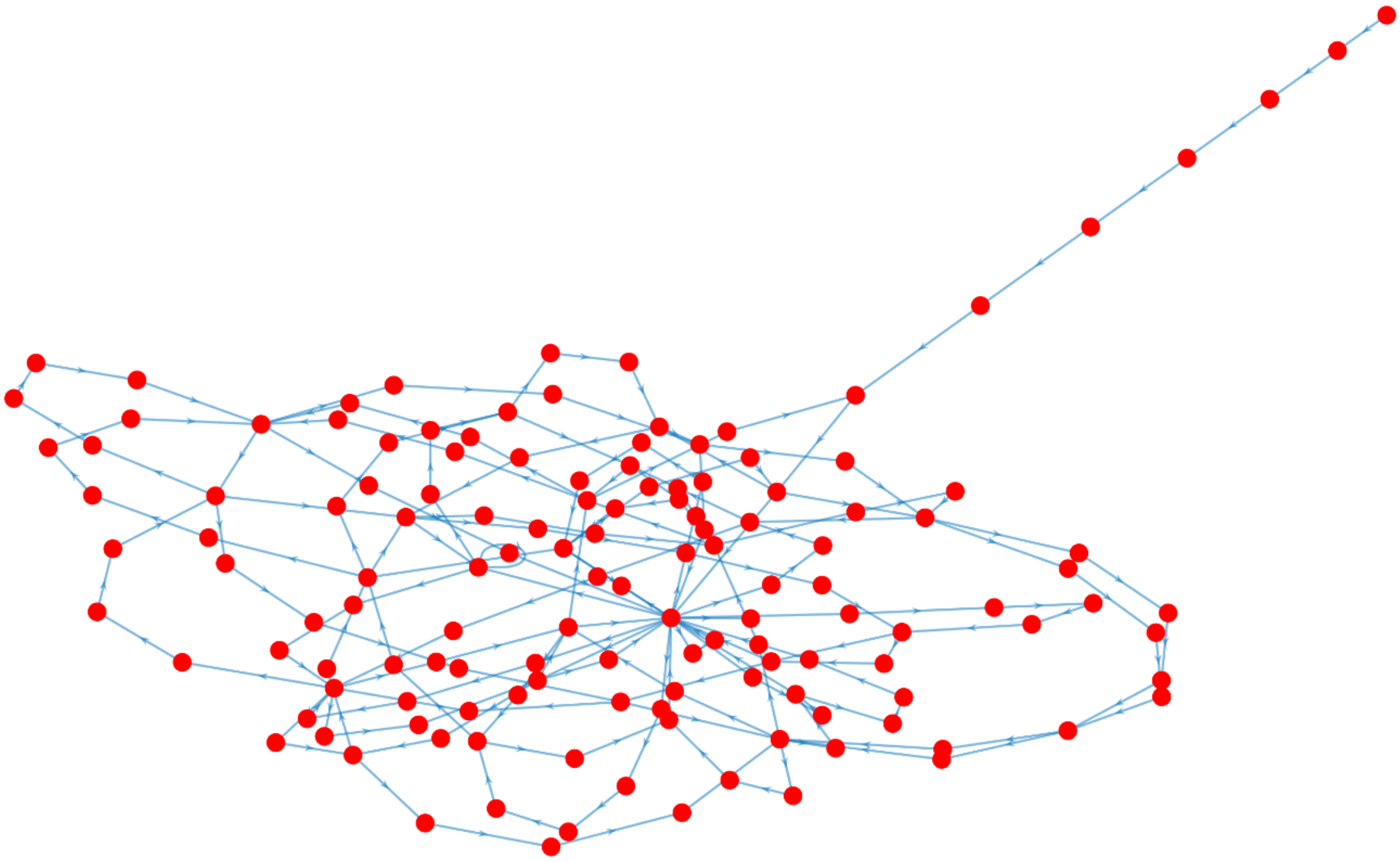

Supplementary Material 6

As shown above and in the main text, the word centrality of dream reports was the most consistent predictor for self-reported depression scores (PHQ-9).

We therefore split the participants according to the relevant ordinal categories (none., mild, moderate-severe, split this way to obtain higher frequency similarity between groups(Kroenke, Spitzer, & Williams, 2001)), and plotted the words with the highest frequency (scaling with word size) using the MATLAB word cloud function.

Figure S2. High frequency words in no depression (PHQ-9>5) group' dream reports

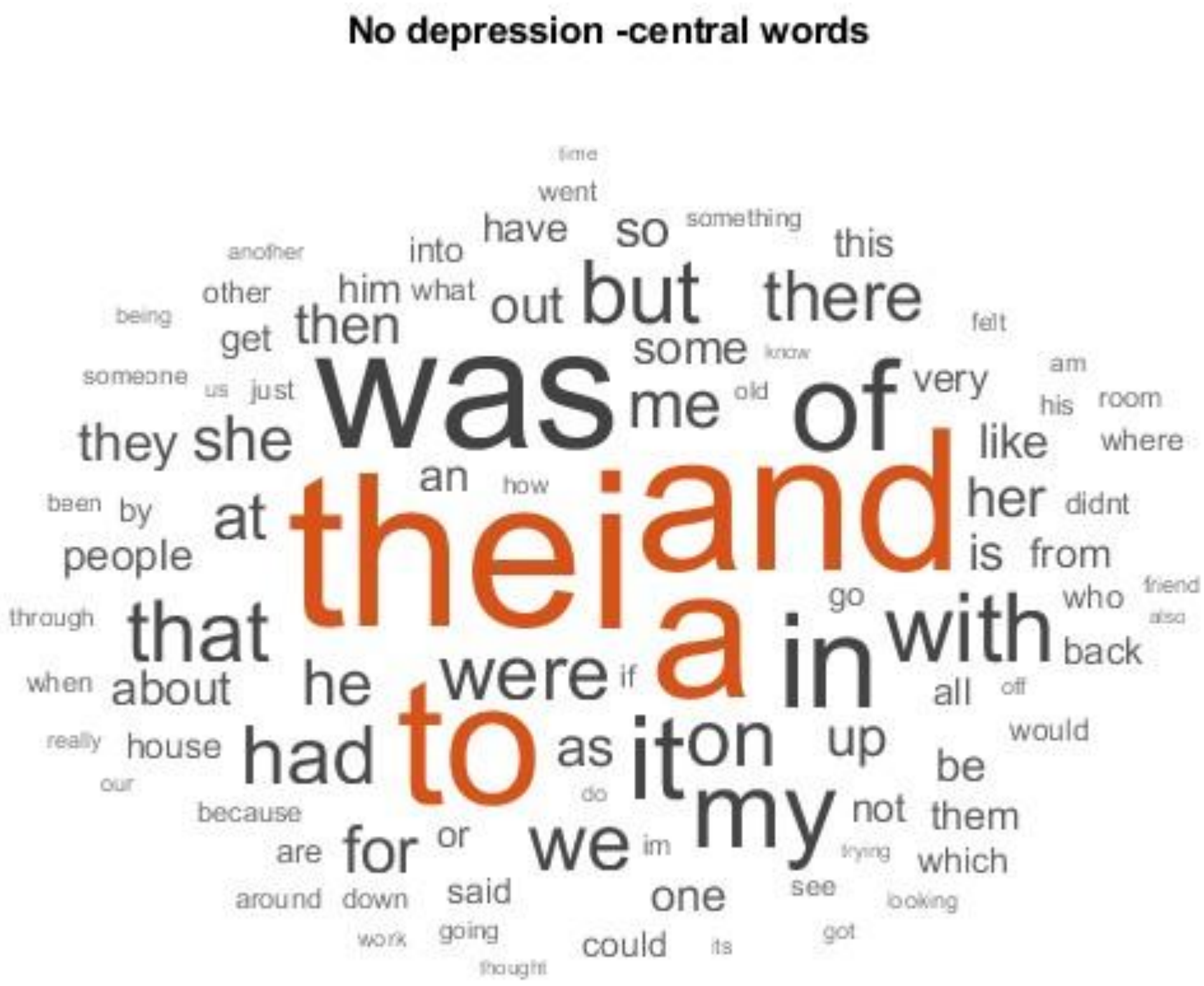

Figure S3. High frequency words in mild depression (4>PHQ-9<10) group's dream reports

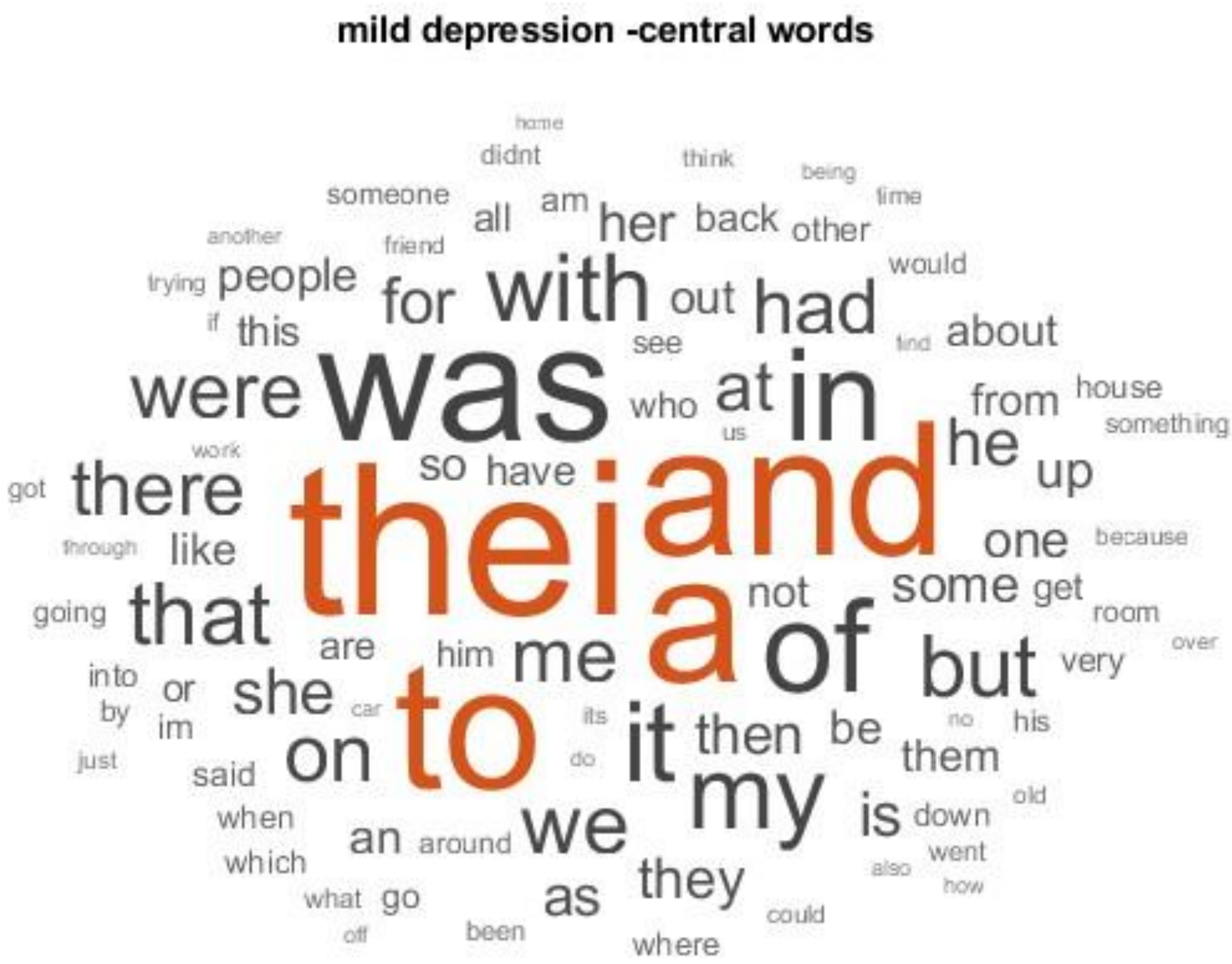

Figure S4. High frequency words in moderate/severe depression (PHQ-9>9) group's dream reports

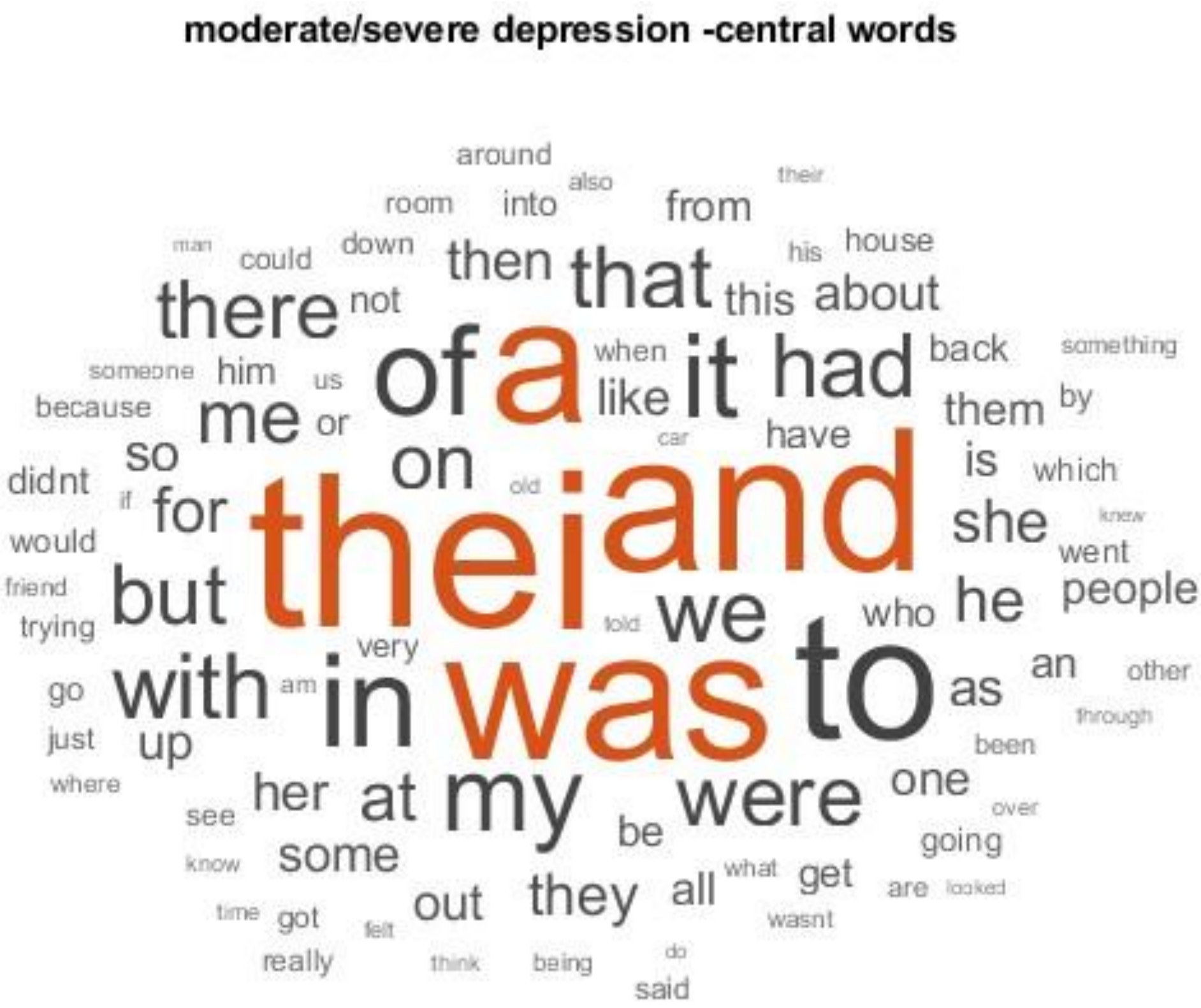

No substantial differences are obvious from these plots.

Supplementary Material 7

Below are descriptive statistics of the relevant continuous variables that were inserted into the statistical models

Table S13 Descriptive statistics for variables of interest. Presented are mean and standard deviation for dream and mind wandering reports and both of them together.

Variable	Mean			Standard Deviation		
Types of report	Dreams	Mind wandering	Both Types of Reports	Dreams	Mind wandering	Both Types of Reports
Word count	197.52	201.51	199.39	163.30	140.02	152.79
PC1	-0.99	1.10	-0.004	2.80	2.85	3.01
PC2	0.02	-0.02	0.0009	0.60	0.64	0.62
Assortativity	-0.15	-0.13	-0.14	0.06	0.05	0.05
Maximum Betweeness centrality	0.55	0.56	0.55	0.042	0.039	0.037
Clustering	0.13	-0.15	0.0004	1.04	0.92	0.999
Age	48.87	49.59	49.21	15.16	15.26	15.21

Please note that all the variables were z scored before being inserted into the statistical models. For further transformation of the data to ensure normality, see supplementary material 2.

Figure S5 Shows the frequency of word counts of reports.

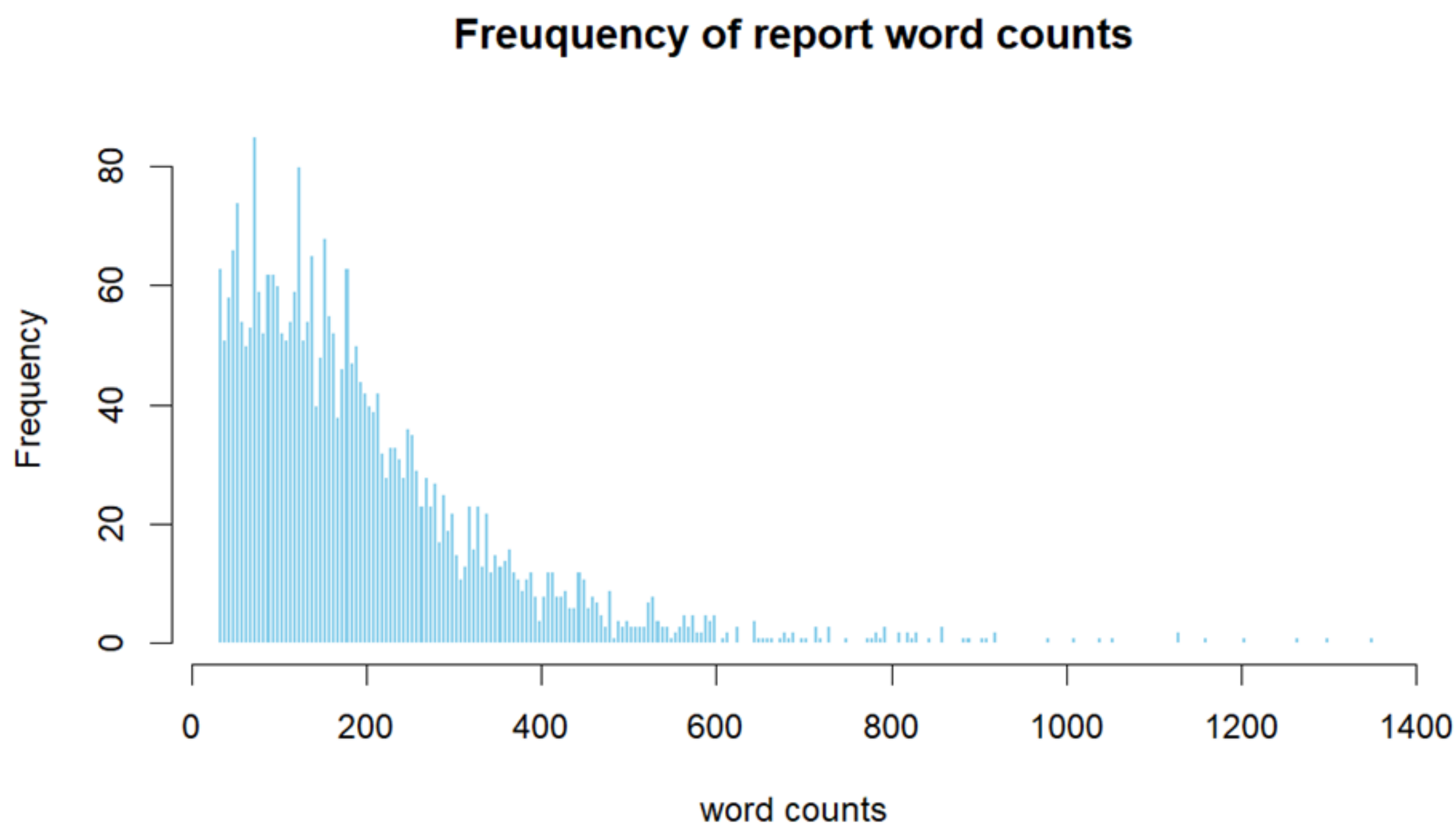

Please note that we excluded all reports with less than 30 words to ensure graph theory metrics were stable. Word counts were log transformed and z scored before being inserted in statistical models (supplementary material 2).

Figure S6 Frequency of reports per person.

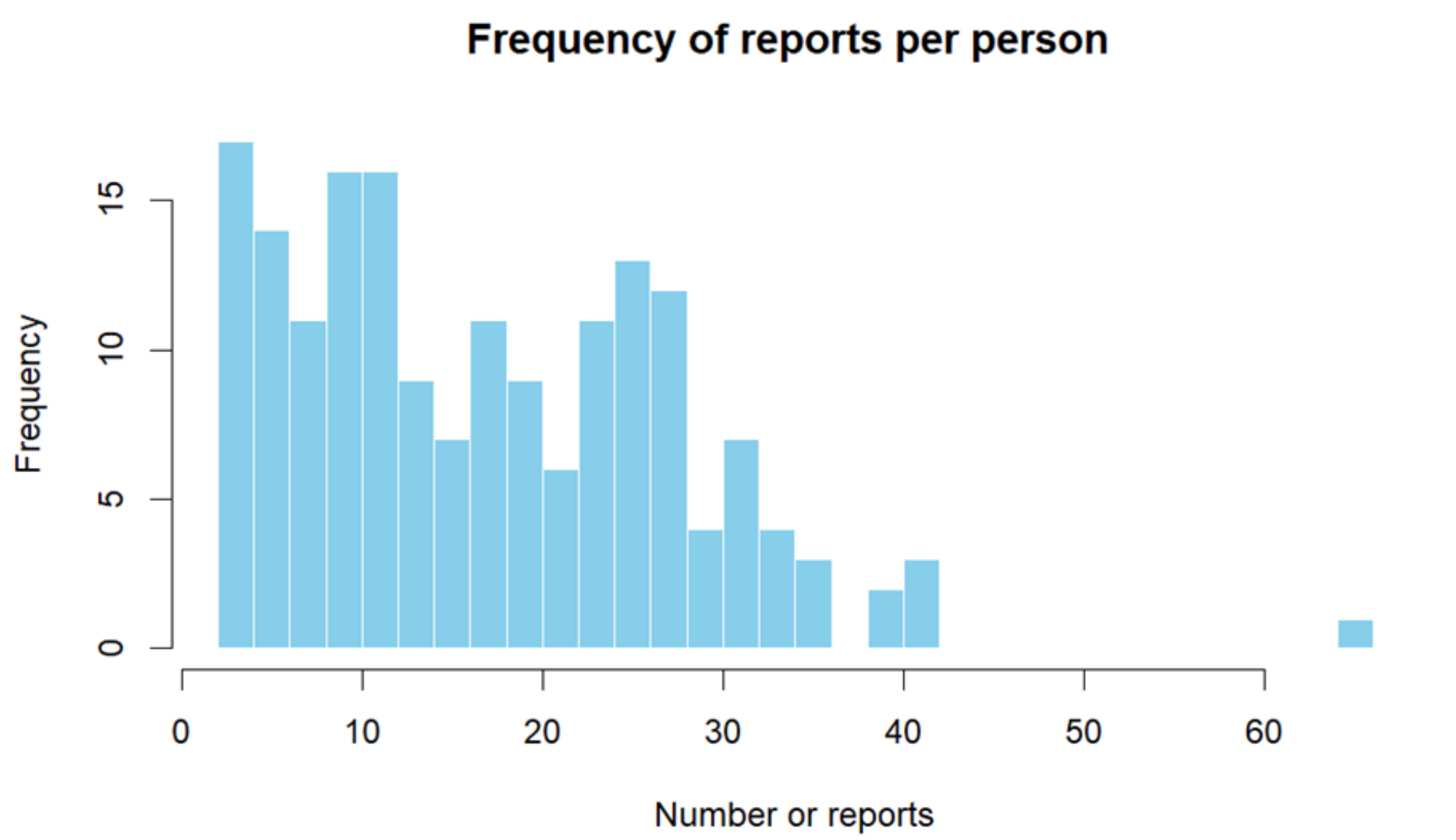

Figure S7 Frequency of dream reports per person

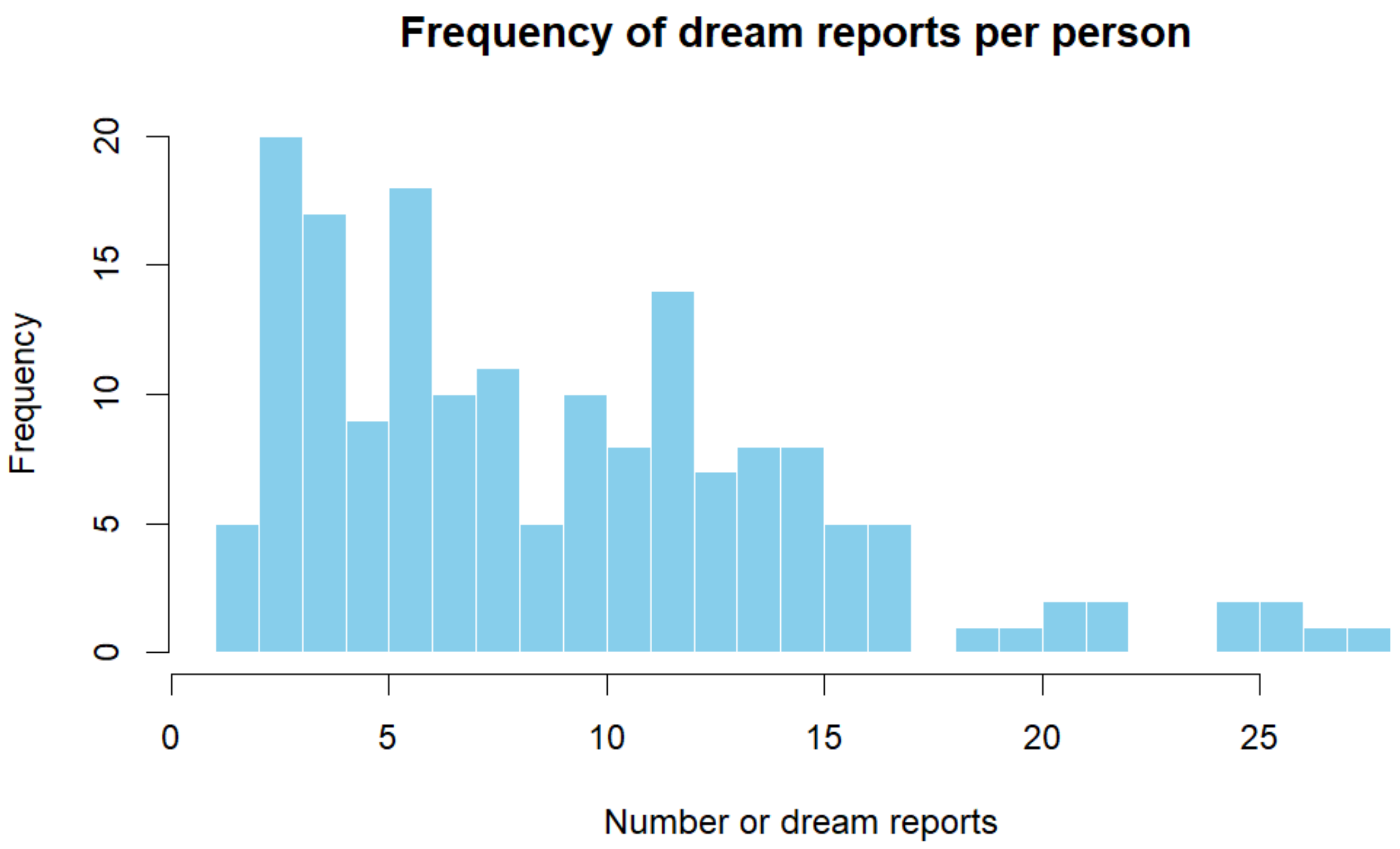

Figure S8 Frequency of mind wandering reports per person

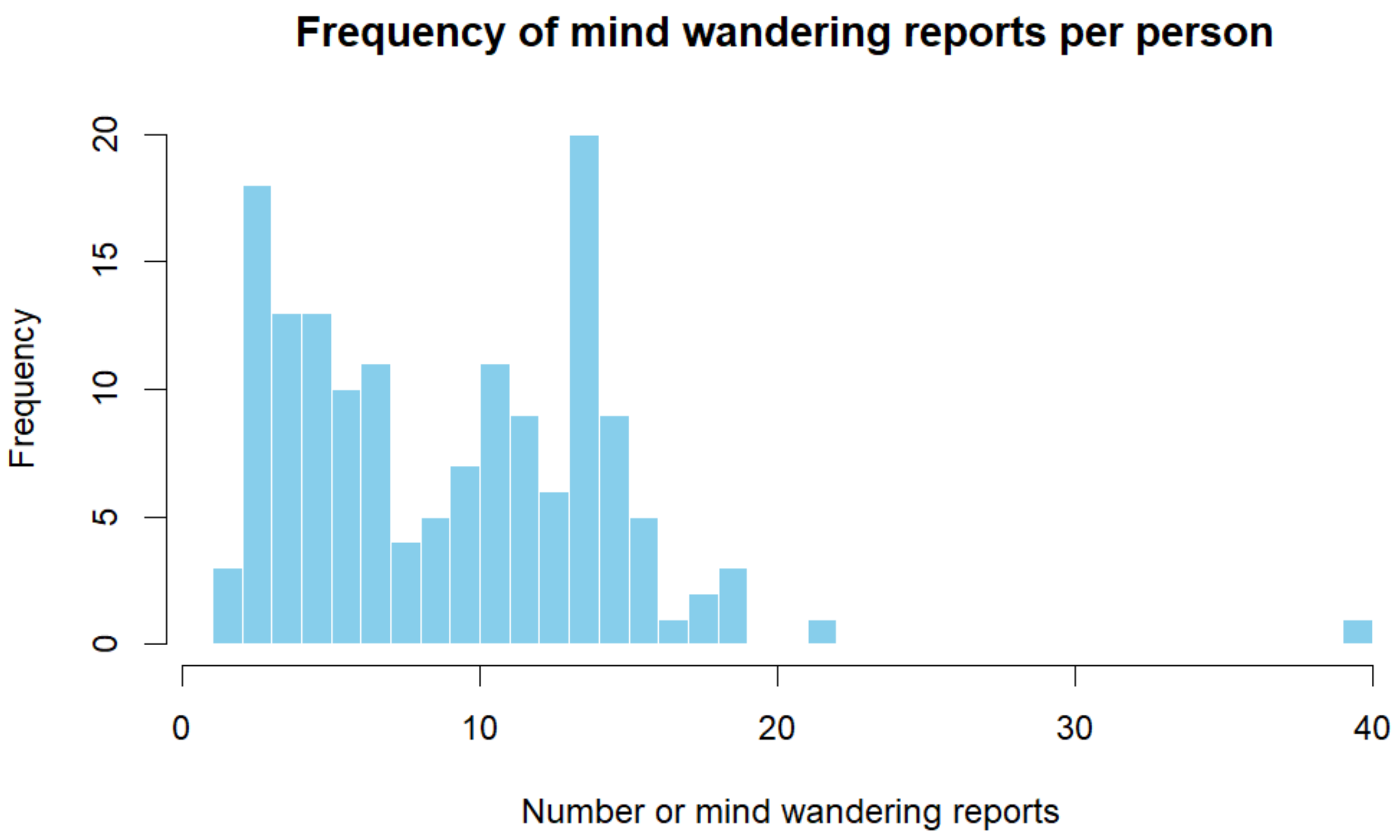

Table S13 Numbers of comparison within and between persons for dreams and mind wandering reports for Hypothesis 2.

Within and between comparison	Type of report	Number of comparison
Within individual	Dream	9654
Within individual	Mind wandering	8250
Within individual	Between Dream and Mind wandering	16888
Between individuals	Dream	1300117
Between individuals	Mind wandering	1019211
Between individuals	Between Dream and Mind wandering	2304758

Table S14 Counts for different categories of the PHQ-9 and GAD-7 following averaging across repeated measures.

	None	Mild	Moderate-Severe
GAD-7	88	52	36
PHQ-9	64	66	46
